# An individual participant data meta-analysis on metabolomics profiles for obesity and insulin resistance in European children

**DOI:** 10.1038/s41598-019-41449-x

**Published:** 2019-03-25

**Authors:** Christian Hellmuth, Franca F. Kirchberg, Stephanie Brandt, Anja Moß, Viola Walter, Dietrich Rothenbacher, Hermann Brenner, Veit Grote, Dariusz Gruszfeld, Piotr Socha, Ricardo Closa-Monasterolo, Joaquin Escribano, Veronica Luque, Elvira Verduci, Benedetta Mariani, Jean-Paul Langhendries, Pascale Poncelet, Joachim Heinrich, Irina Lehmann, Marie Standl, Olaf Uhl, Berthold Koletzko, Elisabeth Thiering, Martin Wabitsch

**Affiliations:** 1 LMU - Ludwig-Maximilians-Universität München, Dr. von Hauner Children’s Hospital, Div. Metabolic and Nutritional Medicine, 80336 Munich, Germany; 2 Division of Pediatric Endocrinology and Diabetes, Interdisciplinary Obesity Unit, Department of Pediatrics and Adolescent Medicine, University of Ulm, 89081 Ulm, Germany; 3 Division of Clinical Epidemiology and Aging Research, German Cancer Reasearch Center (DKFZ), 69120 Heidelberg, Germany; 4 Institute of Epidemiology and Medical Biometry, Ulm University, 89081 Ulm, Germany; 5 Neonatal Intensive Care Unit, Children’s Memorial Health Institute, 04-736 Warsaw, Poland; 6 Pediatric Nutrition and Development Research Unit, Universitat Rovira I Virgili, IISPV, 43201 Reus, Spain; 7 Department of Paediatrics, San Paolo Hospital, University of Milan, 20142 Milano, Italy; 8 Centre Hospitalier Chrétien St Vincent, 4000 Liège-Rocourt, Belgium; 9 Hôpital Universitaire des enfants Reine Fabila, 1020 Bruxelles, Belgium; 10 Institute of Epidemiology I, Helmholtz Zentrum München- German Research Center for Environmental Health, 85764 Neuherberg, Germany; 11 Ludwig-Maximilians-Universität München, Institute and Outpatient Clinic for Occupational, Social and Environmental Medicine, 80336 Munich, Germany; 12 Department of Environmental Immunology/Core Facility Studies, Helmholtz Centre for Environmental Research – UFZ, 04318 Leipzig, Germany; 13 Berlin Institute of Health and Charité- Universitätsmedizin Berlin, Molecular Epidemiology Unit, Berlin, Germany

## Abstract

Childhood obesity prevalence is rising in countries worldwide. A variety of etiologic factors contribute to childhood obesity but little is known about underlying biochemical mechanisms. We performed an individual participant meta-analysis including 1,020 pre-pubertal children from three European studies and investigated the associations of 285 metabolites measured by LC/MS-MS with BMI z-score, height, weight, HOMA, and lipoprotein concentrations. Seventeen metabolites were significantly associated with BMI z-score. Sphingomyelin (SM) 32:2 showed the strongest association with BMI z-score (P = 4.68 × 10^−23^) and was also closely related to weight, and less strongly to height and LDL, but not to HOMA. Mass spectrometric analyses identified SM 32:2 as myristic acid containing SM d18:2/14:0. Thirty-five metabolites were significantly associated to HOMA index. Alanine showed the strongest positive association with HOMA (P = 9.77 × 10^−16^), while acylcarnitines and non-esterified fatty acids were negatively associated with HOMA. SM d18:2/14:0 is a powerful marker for molecular changes in childhood obesity. Tracing back the origin of SM 32:2 to dietary source in combination with genetic predisposition will path the way for early intervention programs. Metabolic profiling might facilitate risk prediction and personalized interventions in overweight children.

## Introduction

Obesity and its associated co-morbidities such as diabetes or cardiovascular diseases are a major challenge not only in high but also in medium and low income countries^1^. The rising numbers of obesity in children are getting closer to the number of underweight children globally: In 2016, 192 million children were moderately or severely underweight while 24 million children were obese^1^. The detrimental effect of excess weight on the expected years of life is greatest in obesity and already present in young adults^2^. While most of the adverse effects of adiposity become apparent only in adulthood, the underlying pathogenic mechanisms are believed to originate in early life^3^. Most overweight children remain in the same or higher BMI category in their adult life, resulting in significantly increased lifetime health costs compared to normal weight children^4^. Current approaches for obesity treatment and prevention in adulthood and childhood are less than satisfactory^1,5–7^. Studies to elucidate the mechanisms of development of childhood obesity on a molecular level may contribute to identifying potential targeted intervention approaches in childhood.

The study of small molecules (<1500 kDa), the so-called “Metabolomics” science, offers the possibility to understand biological responses and alterations due to changes on the genetic, epigenetic or protein level, but also due to environmental exposure like diet, physical activity, microbiome and toxins. Thus, metabolomics can help to define molecular phenotypes and may elucidate mechanisms for obesity and diabetes^8^. Several cross-sectional studies have screened for associations between metabolites and obesity as well as insulin resistance (IR) in adults, but only very few studies exist in childhood obesity and diabetes^9–15^ which were often conducted with small sample sizes of less than 100 children^16^ resulting in limited statistical power and a high margin of error. This is especially limiting in the case of metabolomics data that require statistical adjustment for multiple testing. Another challenge in current metabolomics research is the lack of standardization. Results of different research groups are not easily comparable because different analytical approaches were used for determination of metabolites and data analysis^8,9,17^.

In the present study, we combined metabolomics data from three large European cohorts: the 5.5 and 8 year follow-ups of the European Childhood Obesity Project study (CHOP)^18^, the 8 year follow-up of the Ulm birth cohort study (UBCS)^19^, and the 10 year follow-up of the GINIplus/LISA study^20^. We performed an individual participant meta-analysis of all studies for BMI z-scores and homeostasis model assessment (HOMA) levels to explore the molecular basis of obesity and IR development in childhood. All four metabolomics datasets were measured using the same analytical platform comprising metabolites of the lipid- and energy metabolism. The follow-up visits at 5.5 and 8 years of age of the CHOP children allowed for a longitudinal and a predictive analysis of our targeted metabolome.

## Methods

### Study design, anthropometric assessment, and insulin and glucose measurement

In this meta-analysis, we included data of three European studies: The 5.5 and 8 year follow-up visits of CHOP, the 8 year follow-up visit of the Ulm Birth Cohort Study (UBCS), and the combined 10 year follow-up visits of the “German Infant study on the influence of Nutrition Intervention plus environmental and genetic influences on allergy development” (GINIplus) and the “Influence of Life style factors on the development of the Immune System and Allergies in East and West Germany” (LISA) birth cohort studies. All research was performed in accordance with the Declaration of Helsinki. We included only data and bio-samples from fasted children. Anthropometric measurements were used to calculate the age- and sex-specific z-scores for BMI during childhood using a German reference dataset^21^.

#### CHOP study

The CHOP study is a double-blind, randomized, multicenter intervention trial conducted in five countries: Germany, Belgium, Italy, Poland, and Spain^18^. At a mean age of 2 weeks but no later than the age of 8 weeks of life, infants were randomly assigned to a higher or lower protein content infant formula (HP and LP groups, respectively) provided through the first year of life. Additionally, an observational group of breastfed infants was included. This study was approved by the ethics committees of all study centers, and written informed parental consent was obtained for each infant (trial registration: ClinicalTrials.gov; identifier: NCT00338689). Anthropometry data at 5.5 and 8 years were obtained at visits at the study centers. Blood samples were collected during both follow-up visits. Blood samples were collected and centrifuged and the serum samples were frozen at −70 °C. Glucose, HDL cholesterol and LDL cholesterol were analyzed in the respective laboratories of the local study centers with an enzymatic method^22^. An enzymatic reference method with hexokinase or indirect potentiometry was used for glucose measurement. Serum insulin levels were quantified using immunoradiometric assays (DiaSource, Nivelles, Belgium) at the Children’s Memorial Health Institute Warsaw (Poland). HOMA index was calculated: insulin (mU/l) x glucose (mg/dl)/405 (Matthews 1985). For metabolomics measurements, samples were sent on dry ice to Munich and re-stored at −80 °C until final analysis. We only use data from children who were fasted for at least 6 hours prior to blood withdrawal. In total, 396 children from the 5.5 year follow-up and 355 of the 8 year follow-up visit were included.

#### UBCS

The UBCS is a longitudinal prospective birth cohort study. Women that gave birth at the Department of Gynaecology and Obstetrics at the University of Ulm between November 2000 and November 2001 were recruited^23^. Participation was voluntary and written informed consent was obtained in each case. The study was approved by the ethics committees of the Universities of Ulm and Heidelberg and of the physicians’ boards of the states of Baden-Wuerttemberg and Bavaria. There have been regular follow-up examinations up to 8 years, when anthropometry data was obtained by trained staff at the Ulm University hospital and blood samples from 413 children were withdrawn between 8 and 9 a.m. after an overnight fast of at least 10 h. Samples were processed immediately and plasma aliquots were frozen at −80 °C^19^. For metabolomics measurements, samples were sent on dry ice to Munich and re-stored at −80 °C until final analysis. Fasting plasma concentrations of insulin were measured as a batch using a commercially available ELISA (Mercodia). Concentrations of fasting plasma glucose were measured by using HemoCue B-Glucose Analyzer (Quest Diagnostics, Spain). HOMA was calculated accordingly. Fasting lipoproteins ApoAI, ApoB were determined by immunonephelometric methods, using the Health Care Diagnostic Product (Siemens GmbH, Germany) on a Dade Behring nephelometer BNII System.

#### GINIplus/LISA study

The study population consists of a subsample of children living in Munich, Leipzig and Bad Honnef who participated in the 10-year follow-up. GINIplus is a prospective birth cohort of 5,991 children born at full-term and normal weight recruited between 1995 and 1998. Children with at least one atopic parent or sibling were allocated to an intervention study arm which investigated the effect of different hydrolyzed formulas consumed during the first year of life on allergy development (N = 2,252)^24^. All children whose parents did not give consent for the randomized clinical trial or who did not have a family history of allergic diseases were allocated to the observation study arm (N = 3,739). LISAplus is a population-based prospective birth cohort of 3,097 children born at full-term and normal weight recruited between 1997 and 1999. Detailed descriptions of the recruitment and follow-up strategy for both cohorts are available^24,25^. Both studies were approved by the local Ethics Committees (the Bavarian Board of Physicians (reference numbers: 01212 and 07098), University of Leipzig (reference number: 345/2007), and Board of Physicians of North-Rhine-Westphalia (reference numbers: 2003355 and 2008153)) and written consent was obtained from all parents of the participants.

There have been regular follow-up examinations up to 15 years in study centers with anthropometric measurements and blood withdrawal. After blood withdrawal, the samples were processed immediately and plasma aliquots were frozen at −80 °C. For metabolomics measurements at Ludwigs-Maximilians-Universität (LMU), samples were sent on dry ice to Munich and re-stored at −80 °C until final analysis. Overnight fasted samples were available for 252 children. Glucose measurements in blood were performed by standard laboratory methods by the individual hospitals. Fasting insulin in serum was measured centrally by the fully mechanized system, LIAISON (DiaSorin). HOMA-IR was calculated accordingly. The measurement of serum lipoproteins was performed using homogenous enzymatic colorimetric methods according to the manufactures instructions on a Modular Analytics System from Roche Diagnostics GmbH Mannheim.

### Metabolomics analyses

Metabolomics analyses were performed at LMU Munich. Amino acids (AA), non-esterified fatty acids (NEFA), carboxylic acids (CA), acylcarnitines (acyl-Carn) and phospholipids (PL) were measured. We report all metabolite concentrations in µmol/L. As a point to note, the analytical technique applied here is not capable of determining the position of the double bonds and the distribution of carbon atoms between fatty acid (FA) side chains. The acyl-Carn, PL and NEFA are mentioned as X:Y. In this nomenclature, X is the length of the carbon chain, Y is the number of double bonds.

50 µL of plasma were thawed and diluted with 450 µl methanol, containing internal standards representing different groups of metabolites (AA- Labeled amino acid standards set A (NSK-A-1, Cambridge Isotope Laboratories – CIL, USA), ^15^N2-L-Asparagine (NLM-3286-0.25, CIL, USA) and Indole-D5-L-Tryptophan (DLM-1092-0.5, CIL, USA); NEFA- ^13^C16-palmitic acid (CLM-409-MPT-PK, CIL, USA); acyl-CARN D3-acetyl-carnitine (DLM-754-PK, CIL, USA), D3-octanoyl-carnitine (DLM-755-0.01, CIL, USA) and D3-palmitoyl-carnitine (DLM-1263-0.01, CIL, USA); PL- Tridecanoyl-2-hydroxy-sn-glycero-3-phosphocholine (855476, Avanti Polar Lipids, USA) and 1,2-dimyristoyl-sn-glycero-3-phosphocholine (850345, Avanti Polar Lipids, USA)); TCA D3-Methylmalonic Acid (DLM-387-PK, CIL, USA).

After centrifugation (4000 rpm, 10 min, room temperature), supernatants were divided according to the following methods:

#### Amino acids

100 µL of the supernatant were prepared using derivatization as previously reported^26^. The supernatant was evaporated with nitrogen to dryness and the free amino acids were derivatized with 50 µl butanolic HCl for 15 min at 600 rpm at 60 °C. After evaporation, the residue was dissolved in 50 ml flow solution. AA butylesters were determined by ion-pair liquid chromatography coupled to mass spectrometry detection (LC-MS/MS). 10 µL of the prepared sample were injected into the HPLC system (HPLC 1100, Agilent, Waldbronn, Germany) and chromatographic separation was performed with a XBridge C18 column (Waters GmbH, Eschborn, Germany). MS detection was performed with an API 2000 triple quadrupole instrument (Sciex, Darmstadt, Germany) with an APCI source operating in positive ion ionization mode. Data acquisition on the mass spectrometer was controlled by Analyst 1.6.2 software (AB Sciex, Darmstadt, Germany). Data handling and quantification were also performed with Analyst 1.6.2 software.

#### Nonesterified fatty acids

100 µL of the supernatant were analyzed as previously reported^27^. The supernatant was mixed for 20 min at 600 rpm at room temperature and transferred for LC-MS/MS analysis. An UPLC diphenyl column (Pursuit UPS Diphenyl, Varian, Darmstadt, Germany) was used for chromatographic separation with an Agilent 1200 SL series HPLC system (Waldbronn, Germany). The injection volume was set to 10 µL with an eluent flow rate of 700 mL/min. A hybrid triple quadrupole mass spectrometer (QTRAP4000, Sciex, Darmstadt, Germany) operating in negative ESI mode was coupled to the HPLC system for identification of NEFA.

#### Carboxylic acids

Carboxylic acids (CA) were measured by a modified LC-MS/MS method based on previously published methods^28^. 100 μL of the supernatant were evaporated to dryness and re-suspended in 50 μL water. Five μL of the extracted samples were injected to an Agilent 1200 HPLC and molecular species were separated on a Kinetex F5 core-shell HPLC column, 150 × 2.1 mm, 2.6 μm particle size (Phenomenex, Aschaffenburg, Germany). The mobile phase A was water with 1% formic acid and mobile phase B was composed of methanol/isopropanol (50:50) with 1% formic acid. The gradient elution at a flow rate of 200 μL/min was from 1% B to 85% B within 9 minutes and turned back to initial conditions of 1% B within 1 minute. Re-equilibration was held for 5 minutes at 1% B. The triple quadrupole mass spectrometer (QTRAP4000) was operated in negative scheduled multiple reaction monitoring mode using electrospray ionization (ESI).

#### Phospholipids

Flow-injection mass spectrometry (FIA-MS/MS) was used to analyze PL. 30 μL of the centrifuged supernatant was mixed with 500 µL methanol (containing 1 µM ammonium acetate) for 20 min at 600 rpm and then used for FIA-MS/MS analysis. Samples were analyzed with a triple quadrupole mass spectrometer (QTRAP4000) with an electrospray ionization (ESI) source which was used in both positive and negative mode. The MS was coupled to an Agilent 1200 SL series HPLC system. MS/MS analysis was run in Multiple Reaction Monitoring (MRM) mode with 184 Da (choline head group) as product ion for the PL. Analyst 1.6.2 software and the statistical program R (R Project for Statistical Computing, http://www.r-project.org/) were used to post-process the entire analytical process.

The analysis comprised diacyl-phosphatidylcholines (PCaa), acyl-alkyl-phosphatidylcholines (plasmalogens, PCae), sphingomyelins (SM), lyso-phosphatidylcholines (lysoPC), and sum of hexoses. “a” indicates that the acyl chain is bound via an ester bond to the backbone, while “e” means binding by an ether bond.

Using FIA-MS/MS, it is not possible to determine which single FA is bound to the SM backbone and of which configuration this backbone is. To identify the exact configuration of SM 32:2, a chromatographic separation was used which was previously described for individual glycerophospholipid species^29^, combined with mass spectrometric fragmentation of lithium adducts^30^. Identification was achieved by retention time and fatty acid specific fragmentation and quantification of SM 32:2 was achieved by comparison to commercially available standard SM (d18:1/18:0). A calibration curve from 0.1 µM to 5.0 µM standards were prepared equal to 6 quality control samples, 9 plasma samples containing low levels of SM 32:2 and 10 plasma samples containing high levels of SM 32:2. 20 µL of samples were prepared by adding 200 µL methanol including SM (d18:1/6:0) as internal standard. After mixing, freezing at −20 °C for 20 minutes, and centrifugation (4000 U/Min for 10 min), the supernatant was used for the analyses of SM by LC-MS/MS analyses with 1200 SL HPLC system coupled to a 4000QTRAP tandem mass spectrometer. Chromatographic separation was achieved with Kinetex C18, 2.6 µm, 100 × 2.1 mm HPLC column (Phenomenex, Aschaffenburg, Germany) and gradient elution^29^. Mass spectrometric detection was conducted in positive MRM with 184 Da as fragment as well as fragments of lithium induced fragmentation^30^. The two potential combinations of SM 32:2 shared the same precursor ion mass (679.5 Da), but had different lithium-induced fragments (SM (d18:1/14:1): 250.2, 264.2; SM (d18:2/14:0): 252.2, 262.2). For the MRM transition, we focused on 679.5/250.2 and 679.5/252.2.

#### Acylcarnitines

FIA-MS/MS was used to analyze acyl-CARN. 100 μL of the centrifuged supernatant was used for FIA-MS/MS analysis. Samples were analyzed with a QTRAP4000 mass spectrometer with an electrospray ionization (ESI) source which was used in positive ionization mode. The MS was coupled to an Agilent 1200 SL series HPLC system. MS/MS analysis was run in MRM mode. Analyst 1.6.2 software and the statistical program R (R Project for Statistical Computing, http://www.r-project.org/) were used to post-process the entire analytical process.

#### Quality Control

The quality control (QC) procedure was applied to the measurement of each follow-up visit separately.

For the CHOP study and GINIplus/LISA study, we assessed the measurement quality using 6 QC samples per batch. A batch was included in the analysis if the intra-batch coefficient of variation after outlier elimination (exclusion of the most extreme measurement lying more than 1.5 IQR apart from the nearest measurement) was < 0.2. If less than five QC sample measurements were available in a batch, the batch was not included either. A metabolite was included if it passed quality control in at least 50% of the batches. Inter-batch variation was removed by calculating the median ratios of the quality controls and using these to align the medians of the samples.

To quantify measurement accuracy of the UBCS, six plasma quality control (QC) samples were consistently measured twice along with the samples per batch. We calculated the coefficient of variation (CV) for each QC sample across the batches and excluded metabolites whose CV was > 35%.

### Statistical analyses

Statistical analyses were performed using the statistical program R (version 3.3.3). Initially, we screened the data for extreme and potentially influential observations in BMI, HOMA levels or concentrations of metabolites and removed the measurement if it was >1.5 times the SD away from the second highest value. The batch effect was removed by dividing each measurement of a batch with the corresponding ratio of the batch QC median to the overall QC median. In doing so, the correction necessary to align all batch medians of the QC samples was applied to the samples in the respective batch. The procedure was performed for each study and follow-up separately.

To assess the associations of outcomes and metabolites, we firstly analyzed each study separately using linear models regressing the outcomes BMI z-score, weight, height, LDL, HDL, and HOMA levels on the metabolites. We ran bivariate linear models and, with exception of BMI z-score, multiple linear models adjusted for child age and sex. In a second step, we compiled the data from the three studies to perform an individual participant data meta-analysis, without the data of the CHOP 5.5 year follow-up. The association between outcome and metabolite was assumed to be the same across all studies (fixed effects model). LDL and HDL were not included in the meta-analysis due to the heterogeneity in the analytical methods to determine the lipoproteins. We furthermore included sex as a potential confounder. Additionally, the meta-analysis models for the HOMA level and metabolites were adjusted for BMI z-score. To quantify the variance explanation capacity of the metabolite in the respective outcome, we calculated the partial R^2^. Heterogeneity of the effect sizes was assessed using Cochran’s Q^31^. We furthermore repeated all analyses stratifying according to the child’s sex. We used the same models as described above without adjustment for sex.

Predictive analyses were based on the 5.5 and 8 years data from the CHOP study. For prediction, z-BMI and HOMA levels at 8 years were regressed on the metabolite concentrations of the 5.5 year follow-up in bivariate linear regression models and adjusted for either BMI z-score or HOMA at 5.5 years.

All p-values were adjusted for multiple testing by using the Bonferroni method. A Bonferroni corrected p-value < 0.05 was considered statistically significant.

## Results

Some 286 metabolites passed the QC in at least one study/follow-up, while 108 metabolites could be adequately determined in all studies and follow-ups (Supplemental Table 1). Among these, we measured 21 AA, H1 (sum parameter for hexoses), free carnitine, 10 acyl-Carn, 11 lysoPC, 18 PCaa, 16 PCae, 8 SM, and 22 NEFA in all studies. Seventeen different carboxylic acids were measured only in the GINIplus/LISA study and both follow-ups of the CHOP study. Hereof, seven intermediates of the tricarboxylic acid cycle, two ketone acids (products of BCAA metabolism), alpha-aminoadipic acid, and taurine passed the QC criteria at all three time-points.

Characteristics of the studied children are described in Table 1. The mean BMI z-score was 0.09, and only 4% of the children had a BMI z-score > 2. Insulin and glucose levels were similar across the studies. In total, 109 children (8%) had a HOMA > 2.5.

**Table 1:** Characteristics of the studied populations. Values are presented as mean +/− SD or n (%).

	CHOP 5.5 years (n = 396)	*Included in the meta-analysis*		
		CHOP 8 years (n = 355)	UBCS 8 years (n = 413)	GINIplus/LISA 10 years (n = 252)
*Age (years)*	5.5 ± 0.07	8.1 ± 0.10	8.2 ± 0.16	10.2 ± 0.25
*Sex (female)*	198 (50%)	180 (51%)	207 (51%)^a^	126 (50%)
*BMI (kg/cm²)*	15.9 ± 2.00	16.9 ± 2.76	16.1 ± 2.05	18.6 ± 2.96
*zBMI*	0.2 ± 1.00	0.2 ± 1.08	−0.2 ± 0.96	0.3 ± 1.01
*Height (cm)*	113.7 ± 4.51	129.5 ± 5.63	131.3 ± 5.35	144.9 ± 6.75
*Weight (kg)*	20.7 ± 3.49	28.5 ± 6.21	27.9 ± 4.91	39.2 ± 8.39
*HOMA*	1.3 ± 0.68	1.8 ± 0.70	0.6 ± 0.36	1.9 ± 1.20
*Insulin(µU/ml)*	6.4 ± 3.08	8.8 ± 3.16	2.9 ± 1.52	8.5 ± 5.19
*Glucose (mg/dl)*	82.8 ± 6.86	83.5 ± 7.66	88.6 ± 11.90	87.3 ± 6.19
*LDL (mg/dl)*	99.3 ± 26.22	94.5 ± 24.52		95.3 ± 27.43
*HDL (mg/dl)*	54.3 ± 14.43	60.3 ± 15.45		51.4 ± 12.01
*LDL/HDL*	2.4 ± 8.19	1.7 ± 0.69		1.9 ± 0.70
*Triglycerides (mg/dl)*	61.0 ± 36.56	59.2 ± 26.48		87.5 ± 37.71
*Cholesterol (mg/dl)*	166.4 ± 30.51	166.9 ± 26.77		196.4 ± 36.52
*ApoB (g/l)*			0.69 ± 0.1	
*ApoAI (g/l)*			1.47 ± 0.2	
*ApoB/ApoAI*			0.48 ± 0.11	

^a^Eleven subjects had no information on sex.

### Associations with BMI z-score, weight and height

**Figure 1:**
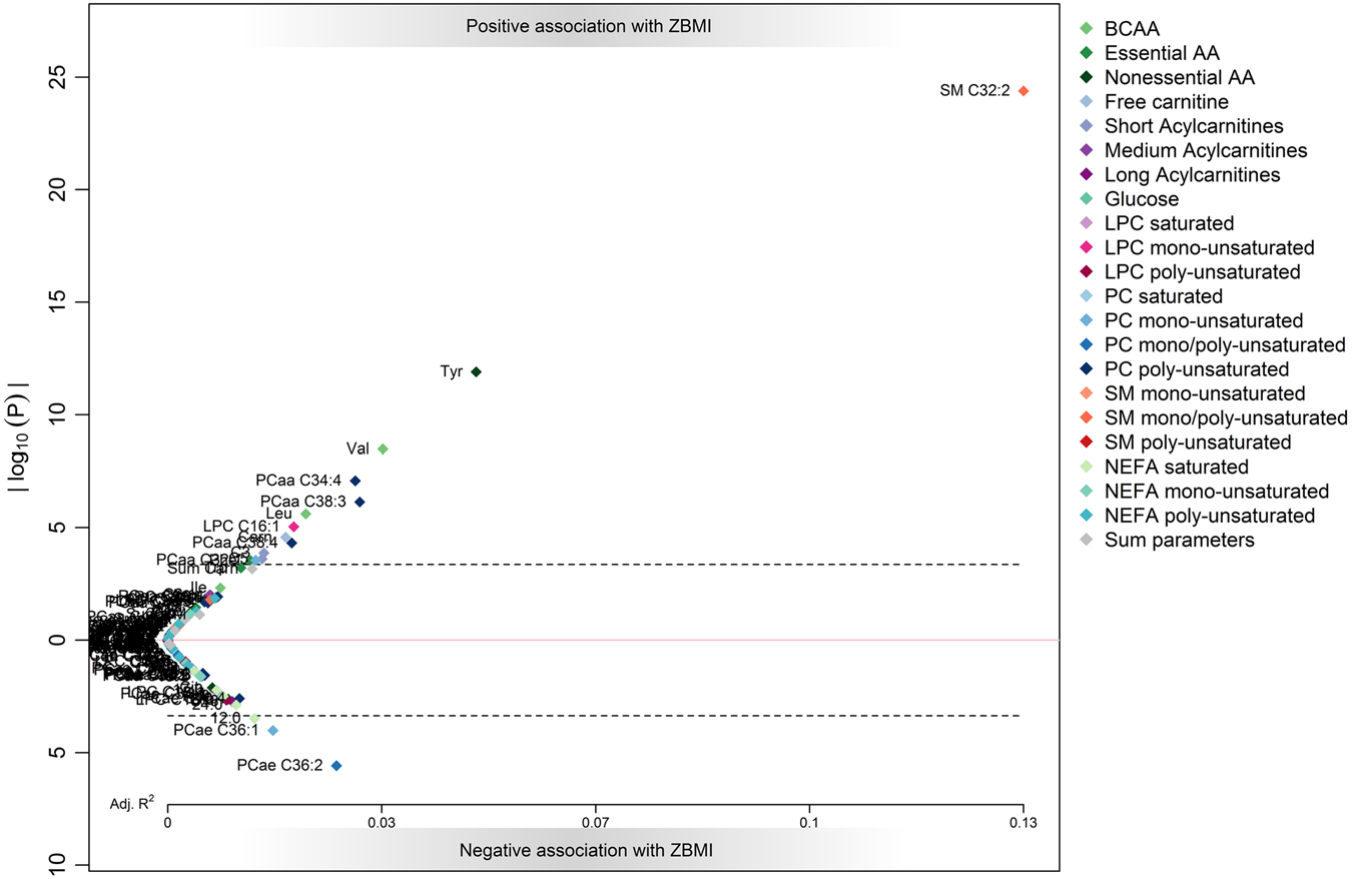
Results of the individual participant meta-analysis of the association of metabolites to BMI z-score. Log transformed p-values (y-axis) are plotted against adjusted R² (x-axis), with positive associations in the upper part (above 0) and negative associations in the lower part (below 0) of the figure. The dashed line indicates the Bonferroni-corrected significance level of 0.0004. Abbreviations: AA, amino acids; BCAA, branched-chain amino acids; LPC, lyso-phosphatidylcholine; NEFA, nonesterified fatty acid; PC, phosphatidylcholine; SM, sphingomyelin.

Results of the meta-analysis are displayed in Fig. 1. In total, 16 metabolites were significantly linked to BMI z-score in the meta-analysis.

**Figure 2:**
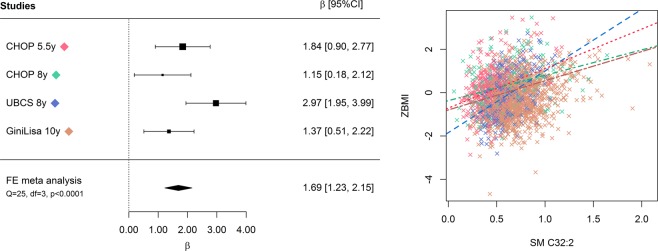
Forest plot and scatterplot on the association of SM 32:2 with BMI z-score. The forest plot (left) shows the study-specific regression estimates (and 95% confidence intervals [95%CI] corrected for multiple testing) of the age and sex adjusted regression models quantifying the effect of an increase of 1 µM in SM 32:2 on BMI z-score. The regression estimate and 95%CI for the individual fixed-effects (FE) participant data meta-analysis adjusted for sex is shown as a diamond. Heterogeneity of study estimates was assessed using Cochran’s Q-test. The scatterplot (right) shows the results of study-specific bivariate fits.

SM 32:2 was the metabolite that showed the strongest association with BMI z-score (R^2^ = 0.13, Bonferroni corrected *P* = 4.68 × 10^−23^, Fig. 2) followed by tyrosine (R^2^ = 0.05, *P* = 1.45 × 10^−10^), valine (R^2^ = 0.03, *P* = 3.48 × 10^−7^), PCaa 34:4 (R^2^ = 0.03, *P* = 9.54 × 10^−6^), and PCaa 38:3 (R^2^ = 0.02, *P* = 8.41 × 10^−5^). Fewer metabolites were negatively associated with BMI z-score. Only three metabolites, namely PCae 36:2 (R^2^ = 0.03, *P* = 3.00 × 10^−4^), PCae 36:1 (R^2^ = 0.02, *P* = 0.011), and NEFA 12:0 (R^2^ = 0.01, *P* = 0.038) were significantly and negatively associated with BMI z-score at the Bonferroni corrected significance threshold.

Results of the study-specific regression models are shown in Table 2 (significant associations only) and Supplemental Table 2. Except for tyrosine, valine, free carnitine and SM 32:2, all metabolites were associated with BMI z-score in one population only.

**Table 2:** Significant associations with BMI z-score in the three studies/four follow-ups.

Metabolite	CHOP 5.5 years			CHOP 8 years			UBCS 8 years			GINI/LISA 10 years		
	N	Estimate	p-value	N	Estimate	p-value	N	Estimate	p-value	N	Estimate	p-value
Tyr	386	8.14E − 03	1.00E + 00	353	2.73E − 02	7.90E − 07	401	1.46E − 02	4.73E − 04	231	6.90E − 03	1.00E + 00
Val	386	1.08E − 03	1.00E + 00	354	5.86E − 03	3.47E − 02	402	4.01E − 03	2.92E − 02	231	3.29E − 03	1.00E + 00
free Carnitine	391	2.24E − 02	5.90E − 03	354	7.41E − 03	1.00E + 00	401	2.90E − 02	5.07E − 03	173	1.84E − 02	1.00E + 00
Carn C20	389	2.50E + 00	1.00E + 00	348	−4.47E + 01	1.45E − 02						
LPC C14	245	5.50E − 02	1.00E + 00				402	5.08E − 01	6.54E − 05	232	1.35E − 01	1.00E + 00
LPC C16:1	391	2.19E − 03	1.00E + 00	354	7.10E − 02	1.00E + 00	401	3.91E − 01	5.54E − 03	232	2.78E − 01	5.12E − 02
PCaa C34:4	391	2.43E − 01	8.74E − 01	304	2.63E − 01	6.21E − 01	402	2.62E − 01	4.54E − 03	232	1.81E − 01	1.00E + 00
PCaa C38:3	391	1.08E − 02	1.00E + 00	167	6.74E − 03	1.00E + 00	401	2.05E − 02	2.71E − 02	232	1.72E − 02	9.95E − 02
PCae C36:2	390	8.17E − 03	1.00E + 00	155	−7.68E − 02	3.21E − 02	402	−3.61E − 02	1.00E + 00	232	−3.86E − 02	1.00E + 00
SM C32:2	351	1.83E + 00	4.80E − 09	171	1.17E + 00	6.22E − 03	326	2.85E + 00	3.05E − 18	232	1.35E + 00	1.60E − 05
SM C34:2	390	7.46E − 02	1.58E − 04	354	4.61E − 02	7.67E − 01				232	3.99E − 02	1.00E + 00
SM C36:2	390	8.07E − 02	8.21E − 03	221	6.17E − 02	1.00E + 00	402	4.63E − 03	1.00E + 00	232	6.84E − 02	1.00E + 00
NEFA 19:0				283	−1.32E + 00	4.33E − 02				216	−8.23E − 01	1.00E + 00
NEFA 20:0				338	−6.61E − 01	3.97E − 03	360	−1.23E − 01	1.00E + 00	123	−2.70E − 01	1.00E + 00
Citrate	388	4.06E − 04	1.00E + 00	296	−9.09E − 03	2.83E − 03				232	−1.54E − 02	1

Results are from linear models regressing the BMI z-score on the metabolite. P-values were Bonferroni corrected. Abbreviations: Carn, acylcarnitine; LPC, lyso-phosphatidylcholine; NEFA, nonesterified fatty acids; PCaa, diacyl-phosphatidylcholine; PCae, alkl-acyl-phosphatidylcholine; SM, sphingomyelin.

SM 32:2 was the only metabolite which was significantly associated with BMI z-score in all four populations (Fig. 2). The regression estimates ranged from 1.15 to 2.97. The Q-test on heterogeneity across the three populations included in the meta-analysis was significant (p < 0.0001). SM 32:2 furthermore explained the highest proportion of variance in BMI z-score (R^2^ ranged from 0.10–0.23). Tyrosine, valine, and free carnitine were positively associated with BMI z-score in two studies, but R^2^ was lower.

For the meta-analysis of height and weight, we found 21 significant associations for metabolites with weight and seven significant associations with height (Table 3, Supplemental Tables 3 and 4). SM 32:2 was strongly and positively associated with weight (*P* = 1.60 × 10^−18^), but non-significantly with height (*P* = 0.061).

**Table 3:** Significant associations of metabolites with weight and height in the meta-analysis and the corresponding results from the linear regression models in each study/follow-up.

				Inlcuded in Meta-Analysis											
Metabolite	N	Estimate	p-value	N	Estimate	p-value	N	Estimate	p-value	N	Estimate	p-value	N	Estimate	p-value
***WEIGHT***	**CHOP 5.5 years**			**CHOP 8 years**			**UBCS 8 years**			**GINI/LISA 10 years**			**Meta-Analysis**		
SM C32:2	353	6.51E + 00	1.95E − 09	171	5.82E + 00	2.06E − 01	326	1.50E + 01	1.91E − 18	251	1.01E + 01	1.25E − 04	748	9.75E + 00	1.60E − 18
Tyr	388	2.08E − 02	1.00E + 00	354	1.42E − 01	3.10E − 05	401	8.84E − 02	3.26E − 06	249	5.88E − 02	1.00E + 00	1004	9.05E − 02	9.43E − 10
NEFA 12:0	180	−5.46E − 02	1.00E + 00	338	−4.67E − 01	1.00E + 00	398	−1.70E − 01	5.20E − 01	207	−2.51E − 01	1.00E + 00	943	−2.39E − 01	4.38E − 05
Val	388	4.82E − 03	1.00E + 00	355	3.07E − 02	1.31E − 01	402	1.94E − 02	6.31E − 02	249	1.68E − 02	1.00E + 00	1006	2.18E − 02	8.53E − 05
PCae C36:2	392	5.92E − 03	1.00E + 00	155	−4.11E − 01	2.25E − 01	402	−2.17E − 01	1.00E + 00	251	−3.18E − 01	1.00E + 00	808	−3.28E − 01	1.13E − 04
Carn	393	7.11E − 02	3.35E − 02	355	5.44E − 02	1.82E − 01	401	1.54E − 01	2.07E − 03	190	2.24E − 01	1.00E + 00	946	6.80E − 02	8.70E − 04
LPC C18:2	393	3.98E − 03	1.00E + 00	355	−8.27E − 02	3.32E − 01	402	2.25E − 02	1.00E + 00	251	−2.06E − 01	6.59E − 02	1008	−9.02E − 02	1.00E − 03
LPC C16:1	393	4.13E − 01	1.00E + 00	355	4.04E − 01	1.00E + 00	401	2.44E + 00	5.11E − 05	251	1.62E + 00	1.00E + 00	1007	9.65E − 01	1.32E − 03
Leu	388	6.84E − 03	1.00E + 00	355	5.94E − 02	3.78E − 01	402	3.16E − 02	7.37E − 01	249	3.06E − 02	1.00E + 00	1006	3.77E − 02	1.98E − 03
Asp	386	1.20E − 01	1.00E + 00	355	9.80E − 02	1.00E + 00	364	1.53E − 01	5.56E − 01	248	1.10E − 01	1.00E + 00	967	1.18E − 01	3.06E − 03
Cit	388	−2.97E − 02	1.00E + 00	355	−1.63E − 01	1.60E − 01	402	−6.11E − 02	1.00E + 00	249	−8.99E − 02	1.00E + 00	1006	−9.78E − 02	3.15E − 03
PCaa C34:4	393	1.09E + 00	4.23E − 02	305	1.11E + 00	1.00E + 00	402	1.52E + 00	2.44E − 04	251	6.27E − 01	1.00E + 00	958	1.14E + 00	5.28E − 03
PCaa C38:3	393	2.93E − 02	1.00E + 00	167	2.72E − 02	1.00E + 00	401	1.10E − 01	1.19E − 02	251	9.45E − 02	1.00E + 00	819	8.07E − 02	9.11E − 03
PCae C34:1	393	2.47E − 02	1.00E + 00	355	−2.96E − 01	1.00E + 00	402	−1.11E − 01	1.00E + 00	251	−4.86E − 01	1.00E + 00	1008	−2.89E − 01	1.67E − 02
Gln	388	−2.78E − 03	1.00E + 00	354	−7.03E − 03	3.23E − 01	401	−6.16E − 04	1.00E + 00	220	−8.78E − 03	1.00E + 00	975	−5.97E − 03	2.07E − 02
NEFA 24:0	168	−3.14E + 00	1.00E + 00	339	−7.75E + 00	1.00E + 00	399	−8.84E − 01	1.00E + 00	206	−1.69E + 01	7.44E − 01	944	−8.12E + 00	2.43E − 02
Phe	388	9.45E − 03	1.00E + 00	354	7.03E − 02	1.00E + 00	402	4.76E − 02	1.00E + 00	249	5.62E − 02	1.00E + 00	1005	5.72E − 02	2.51E − 02
PCae C34:3	393	6.77E − 02	1.00E + 00	355	−2.53E − 01	1.00E + 00	402	3.25E − 02	1.00E + 00	251	−6.20E − 01	2.61E − 01	1008	−2.38E − 01	3.23E − 02
PCaa C38:4	393	1.02E − 02	1.00E + 00	184	2.45E − 02	1.00E + 00	400	−1.95E − 02	1.00E + 00	251	3.39E − 02	1.00E + 00	837	2.77E − 02	3.24E − 02
***HEIGHT***	**CHOP 5.5 years**			**CHOP 8 years**			**Ulm 8 years**			**GINI/LISA 10 years**			**Meta-Analysis**		
NEFA 12:0	178	−9.02E − 02	1.00E + 00	337	−3.13E − 01	1.00E + 00	398	−2.17E − 01	6.54E − 02	208	−1.34E − 01	1.00E + 00	943	−2.00E − 01	4.97E − 04
Tyr	387	−6.45E − 03	1.00E + 00	353	6.26E − 02	1.00E + 00	401	6.99E − 02	7.49E − 03	250	1.92E − 03	1.00E + 00	1004	4.49E − 02	2.63E − 02
Thr	387	1.19E − 02	1.00E + 00	354	1.95E − 02	1.00E + 00	402	2.91E − 02	4.17E − 01	250	1.24E − 02	1.00E + 00	1006	2.36E − 02	3.76E − 02

Results are from multivariate linear models regressing the weight and height on the metabolite, adjusted for sex and age. The meta-analysis is based on the CHOP 8 years, UBCS and GINIplus/LISA infants. P-values were Bonferroni corrected. Abbreviations: Carn, acylcarnitine; LPC, lyso-phosphatidylcholine; NEFA, nonesterified fatty acids; PCaa, diacyl-phosphatidylcholine; PCae, alkl-acyl-phosphatidylcholine; SM, sphingomyelin.

### Associations with HOMA-index

**Figure 3:**
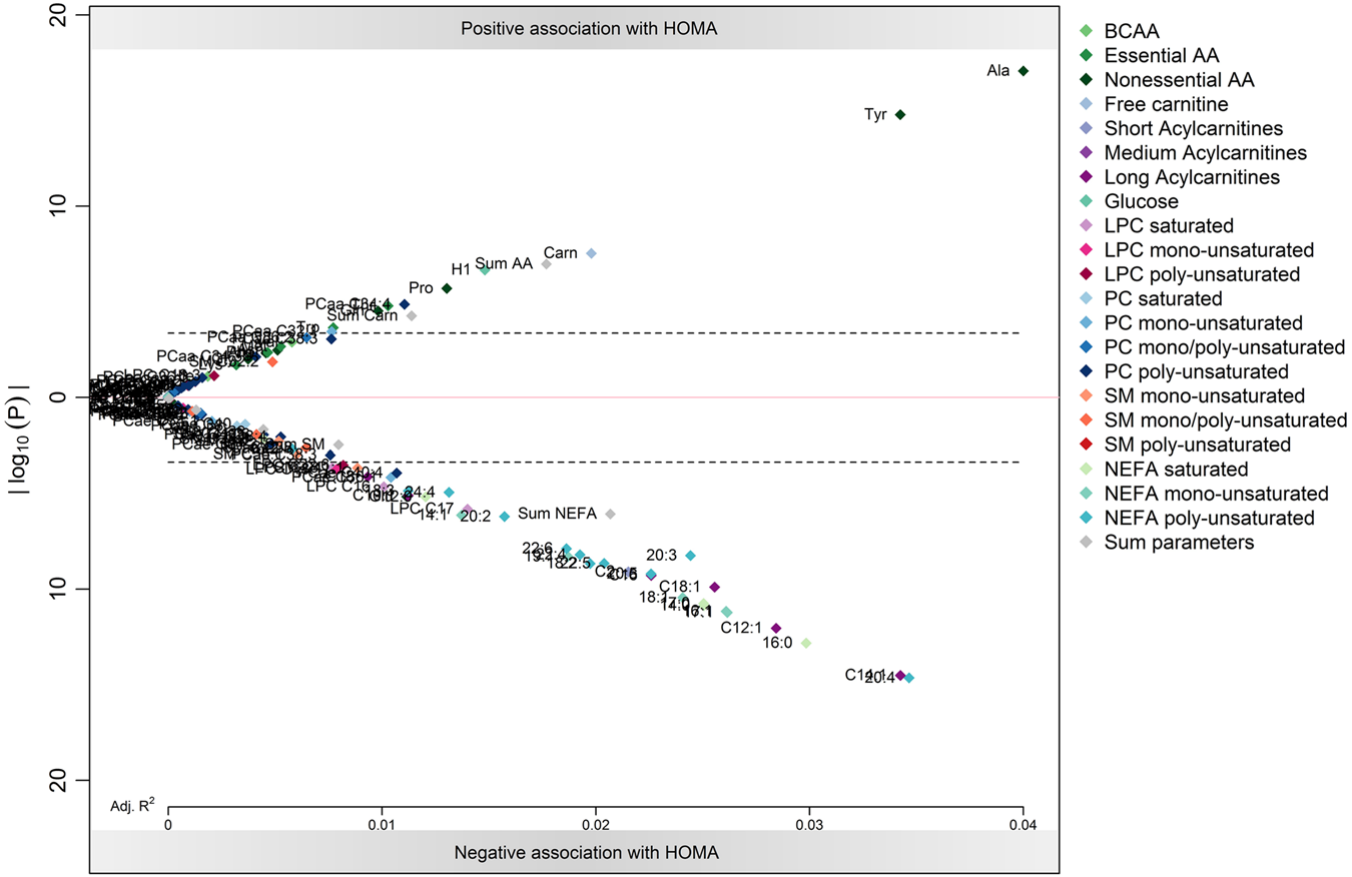
Results of the individual participant meta-analysis of the association of metabolites to HOMA levels. Log transformed p-values (y-axis) are plotted against adjusted R² (x-axis), with positive associations in the upper part of the figure (above 0) and negative associations in the lower part (below 0). Regression models were adjusted for sex. The dashed line indicates the Bonferroni-corrected significance level of 0.0004. Abbreviations: AA, amino acids; BCAA, branched-chain amino acids; LPC, lyso-phosphatidylcholine; NEFA, non-esterified fatty acid; PC, phosphatidylcholine; SM, sphingomyelin.

In the meta-analysis, 35 metabolites were found to be significantly related to HOMA-levels, 10 positively and 25 negatively (Fig. 3).

Alanine was the metabolite most strongly associated with HOMA levels (R^2^ = 0.04, *P* = 9.77 × 10^−16^), followed by tyrosine (R^2^ = 0.04, *P* = 1.88 × 10^−13^). Several Carn and NEFA were negatively associated with HOMA, with Carn 12:1 (R^2^ = 0.03, *P* = 1.94 × 10^−10^) and 14:1 (R^2^ = 0.04, *P* = 3.53 × 10^−13^) as well as NEFA 16:0 (R^2^ = 0.03, *P* = 1.73 × 10^−11^) and 20:4 (R^2^ = 0.04, *P* = 2.62 × 10^−13^) showing the strongest associations. Adjustment for BMI z-score did not alter the associations between HOMA levels and metabolites in the meta-analysis (Supplemental Fig. 1).

In the separate analysis of the four populations, we identified 68 metabolites as significantly associated with HOMA index in one or more populations, 13 of them positively and 55 negatively related to HOMA index (Supplemental Table 5). Carn 14:1 was negatively associated with HOMA-index in all four populations. Among the metabolites negatively related to HOMA in two studies were eleven NEFA species, acetylcarnitine, Carn 4:0.OH, 12:0, 12:1, 16:0, and 18:1. Among the metabolites positively related to HOMA in two studies were alanine, tyrosine, H1 and free carnitine. All three associations were detected in CHOP 8 years and UBCS 8 years.

### Associations with Lipoproteins

Several phospholipid species were positively associated with plasma LDL concentrations (Supplemental Table 6, lowest *P* = 9.96 × 10^−19^ SM 41:1 in GINIplus/LISA 10 years) and HDL (Supplemental Table 7, lowest *P* = 3.03 × 10^−14^ PCae 34:3 in CHOP 8 years) in both CHOP follow-ups and GINIplus/LISA. SM 32:2 was significantly positively associated with LDL (*P* = 3.86 × 10^−7^/1.28 × 10^−7^) in CHOP 5.5 years and GINIplus/LISA 10 years, but not with HDL.

In the UBCS, data on ApoAI and ApoB but not on LDL and HDL were available. Several phospholipid species were also positively associated with ApoAI (Supplemental Table 7, lowest *P* = 6.17 × 10^−19^ PCae 36:3) and ApoB (Supplemental Table 6, lowest *P* = 5.79 × 10^−9^ SM 32:2) in the UBCS. SM 32:2 was also significantly and positively associated with ApoAI (*P* = 3.74 × 10^−4^) in the UBCS.

LDL, HDL, ApoB, and ApoAI were not included in the meta-analysis due to the heterogeneity in the analytical methods used to determine these lipoproteins.

### Sex-stratified analysis

Our findings were similar in girls and boys (Supplemental Fig. 2). The only relevant difference regards the associations between NEFA species and HOMA which were stronger in girls compared to boys, while the associations of alanine and tyrosine with HOMA levels were stronger in boys.

### Predictive and longitudinal analysis in the CHOP study

We used the metabolite concentrations at 5.5years to predict the BMI z-score at 8 years of age in the CHOP study. Plasma levels of free carnitine (*P* = 6.17 × 10^−6^), SM 32:2 (*P* = 2.16 × 10^−4^), SM 34:2 (*P* = 3.09 × 10^−4^), and Carn 3:0 (*P* = 4.09 × 10^−2^) were significantly associated with BMI z-score at 8 years of age. However, after adjusting for BMI z-score at 5.5 years, no metabolite reached the significance level. Regarding HOMA, we found that glutamine (*P* = 0.013/0.003) as well as NEFA 26:1 (*P* = 0.012/0.015), 26:2 (*P* = 0.002/0.01), 26:3 (*P* = 0.009/0.015) at age 5.5 years were significantly associated with HOMA indices at 8 years in both the unadjusted and adjusted linear model. Only serine was significant in the adjusted model only (*P* = 0.032).

### SM 32:2 molecular identification

With the LC-MS/MS method using mass transition of SM 32:2 (673.5 → 184), only one peak at 8.17 minutes was detected for the molecular mass of SM 32:2 (673.5 Da), excluding the possibility of co-eluents. In the MRM scan of lithium induced SM fragmentation for the two potential SM species d18:1/14:1 and d18:2/14:0, we could identify SM 32:2 containing the fatty acid 14:0 (679.5 → 252.2) and the d18:2 backbone (679.2 → 262.2). Both mass transitions were detected at 8.17 minutes. No signal could be detected for the mass transitions of SM d18:1/14:1 (679.5 → 250.2 and 679.5 → 264.2).

## Discussion

In this study, we combined metabolomics data from three large studies in European children to investigate the metabolic fingerprint of childhood obesity and insulin resistance. All metabolite measurements were quantified with the same targeted, LC-MS/MS based metabolomics platform, which allowed for a comparison of the absolute concentrations across all studies (n = 1,416) and an individual participant data meta-analysis on 1,020 children aged 8 to 10 years.

The most noticeable result is a strong positive association of SM 32:2 with BMI z-score. None of the other metabolites measured showed such a close and consistent relation to BMI z-score or HOMA levels. SM 32:2 is not an unknown metabolite in obesity research, but has been “flying under the radar” – its relation attenuated by other metabolites which got the focus in metabolic research, so far, like NEFA or BCAA^10,11,15,32^. Furthermore, SM 32:2 was not quantified in most studies as it is usually not contained in analytical methods when applying common commercial analytical kits^33^.

In previous studies, SM 32:2 was found to be associated with BMI: When measured in early pregnancy, SM 32:2 showed the strongest positive association with pre-pregnancy BMI^34^. It was furthermore the metabolite with the strongest positive association with BMI in young Australian adults^35^ and in Mexican American adults^36^. In these studies, SM 32:2 was accompanied by other SM with two double bonds. In the present study 34:2 and 36:2 showed much weaker associations with obesity, compared to SM 32:2. Our analytical method provided the total number of carbon atoms and double bonds in the side chains but did not identify the exact molecular species - a known pitfall of most screening methods. In order to examine the structure of SM 32:2, we separated this lipid species by LC and identified myristic acid (14:0) at the amid bond and the sphingadienine (18:2) as the long chain base backbone of the SM.

The SM long chain base backbone originates from the biosynthesis from a fatty acid, with palmitic acid (16:0) being the most prominently used fatty acid and yielding a 18:1 long chain base backbone^37^. This structure is called sphingosine and it is the dominant structure in SM. The backbone structure with 18:2 is called sphingadienine^37^. However, it has to be elucidated if this backbone structure in the human metabolism and circulation arises from endogenous biosynthesis in the subjects themselves or from dietary sources. In case of an endogenous production, higher levels of 16:1 (palmitoleic acid) are a potential reason for higher levels of SM 32:2 in obese subjects. Palmitoleic acid is an endogenous fatty acid and it has been discussed to be an adipose tissue-derived lipid hormone^38^. Cao *et al*. linked NEFA 16:1 administration to suppression of hepatic SCD-1 expression, lower hepatosteatosis and improved insulin action in the muscle. Thus, 16:1 seems to be secreted by adipose tissue and to regulate metabolic processes. The authors speculate about a self-protection mechanism of the human metabolism, preventing the adverse effects by adipose fat depots. SM 32:2 could be involved in this pathway by mediating the effect of 16:1 in the cell. However, NEFA 16:1 was not associated with BMI z-score. We propose the following potential mechanism: palmitoleic acid, more secreted by extensive adipose tissue, is taken up by liver or muscle cells and metabolized into intra-cellular lipid signal molecules like SM 32:2, exhibiting specific effects on the insulin signaling pathways. Intriguingly, other metabolite species containing 16:1 or 18:1 were not affected. Thus, the suggested mechanism was based on 16:1 availability and its incorporation into SM 32:2. Single SM species were previously shown to affect membrane proteins, and thus metabolic pathways^39,40^. The d18:2 is also an agonist of peroxisome proliferator-activated receptor gamma^41^. The potential protective mechanisms of SM 32:2 might be intact in the children studied, since most of them appear to be metabolically healthy. Additionally, NEFA 16:1 is negatively associated with HOMA in three follow-ups and in the meta-analysis. The likely reason is that, given the overall healthy state of our collective, NEFA release from adipose tissue still equals the uptake by peripheral tissue and, thus, no change occurs in NEFA plasma levels with higher BMI; but only in their metabolic products such as SM 32:2. Besides 16:1 as potential precursor, the d18:2 backbone may also arise from a synthesis initiated with 16:0. In this case, one double bond may be introduced in the d18:1 backbone (sphingosine) after biosynthesis. An enzyme facilitating this reaction is the delta8 sphingolipid desaturase^42^. In the case of an enzymatic regulation of SM 32:2, also genetic and epigenetic regulations seem possible. However, delta8 sphingolipid desaturase and the d18:2 backbone (4,8-sphingadienine) are mainly found in bacterial strains, yeast, and plants^41,43^.

The other possible origin of the SM 32:2 backbone is from dietary sources. Dietary sphingolipids are digested in the intestine to the long-chain base backbone, sphingosine-1-phosphate or ceramides; long-chain base plus fatty acid^44^. These components are subsequently absorbed and thus elevated after supplementation with sphingolipids. Dietary SM have previously been related to myelination in the central nervous system^45^. Dietary intake is furthermore found to be highly associated with BMI z-score^46^. Thus, we are aware that the associations between SM 32.2 and BMI z-score can be confounded by dietary intake, but this does not explain the unique role of SM 32.2, because other SM should have been affected as well.

A less likely option is that the 18:2 backbone may arise from a higher 18:2n-6 intake, which is linked to weight gain in mice^47^ and affects obesity-related traits^48^. Since we do not have detailed dietary data, we could not evaluate this hypothesis.

Anyhow, we have no information about the position of the two double bonds in the long chain base and could only speculate about a dietary source of 18:2n-6 (plus endogenous hydroxylation), a dietary source of sphingadienine, the endogenous synthesis of sphingadienine from fatty acid 16:1 or an insertion of a double bond at position 8 to sphingosine, equal to n-10.

Given that the backbone consists of the fatty acid residue 18:2, 14:0 is the other fatty acid incorporated into SM 32:2. In particular, when regarding the outstanding role for SM 32.2, compared to other potential sphingadienine SM like 34:2 or 36:2. Phospholipids containing myristic acid have previously been related to obesity. LPC 14:0 in the serum of 6-month-old infants was predictive for obesity risk at 6 years of age^49^ and percentages of FA 14:0 were also elevated in phospholipids of 15-year-old obese children^50^. Also in the present study, LPC 14:0 was found to be positively related to BMI z-score. Thus, lipids with 14:0, with exception of NEFA 14:0, seem to be higher concentrated in children with high BMI and may subsequently be used more often for the synthesis of SM. 14:0 synthesis may also be promoted by high energy intakes and high dietary glycaemic load (carbohydrate intakes)^51^, known risk factors for obesity development^52^.

One may also state that plasma SM 32:2 is just a marker for cell mass, as is BMI, or for altered lipoprotein levels in obese subjects, since lipoprotein profile changes with elevated BMI^53,54^. Indeed, SM 32:2 was associated with higher LDL levels, but we could not identify an outstanding role compared to the other phospholipid species in our study. Thus, in our study the lipoprotein profile seemed not to be the driving factor. Another topic that should be addressed are the sex-differences in SM 32:2 levels: Previous studies have shown that SM 32:2 was higher in adult women^36,55^, but the association with BMI and other metabolic factors was not different between the sexes^55^. Higher fat depots and higher subcutaneous fat depots in women may be the origin for the higher SM 32:2 levels, supporting the outlined hypothesis. However, our stratified analysis showed no difference between boys and girls for the association of SM 32:2 with BMI z-score. And also regarding the other metabolites, we found no relevant differences between boys and girls which might be due to the young age of the pre-pubertal children included in this analysis.

However, the Q-test on heterogeneity was statistically significant: While the effect estimate in the UBCS was 2.97, estimates were 1.15 and 1.37 for the two other studies included in the meta-analysis. We can only speculate on the reasons leading to these different effect estimates, but most likely the children’s age, country of residence, the time of blood sampling, and different standard operating procedures may play a role. Further studies will be needed in order to further corroborate these estimates, but the key message underlying the importance of that metabolite remains unchanged. Next to the associations between BMI and metabolites, especially SM 32:2, we found some other interesting associations with HOMA levels that have to be discussed. The amino acids alanine and tyrosine were positively related to HOMA in our analysis. Alanine was previously found to be positively associated with fasting insulin and leptin levels, pointing towards its role in gluconeogenesis and insulin resistance onset^56^. Tyrosine has previously been related to lower BMI z-score but also to insulin resistance in obese children^33^. It was furthermore identified as the most important metabolite in obese children^15^. Tyrosine is biosynthesized from the non-dispensable AA phenylalanine, but phenylalanine was not associated with HOMA in our study. Thus, it appears likely that elevated tyrosine concentrations are not primarily resulting from dietary intake but from increased insulin secretion inducing an increase in tyrosine aminotransferase activity^57,58^. Given that the present meta-analysis was conducted with healthy children, alanine and tyrosine have the potential to be early markers for the onset of IR. The negative relation of the Carn 12:1 and Carn 14:1 to HOMA may also point towards an altered fatty acid oxidation in early states of insulin resistance^59^. Next to these two acylcarnitines, we found several NEFA species to be also negatively related to HOMA. Since acylcarnitines are formed from NEFA species in several steps, the lower acylcarnitine levels may be just related to the lower NEFA levels. Again, we have to highlight that we conducted the analyses in presumably healthy children with a low incidence of insulin resistance. Thus, higher insulin levels, giving a higher HOMA value, still have an effect in the adipose tissue, suppressing the hormone-sensitive lipase and resulting in a lower NEFA level^60^ and lower Carn levels.

In addition to the individual participant data meta-analysis, we also performed longitudinal analyses in children of the CHOP study. We calculated predictive models using the metabolite concentration at 5.5 years to model the BMI z-score and HOMA at 8 years. As the sample size was much lower in this analysis including children of the CHOP study only, caution should be exercised when comparing the p-values to the meta-analysis. The only remarkable result was the positive association of the NEFA 26:1, 26:2, and 26:6 levels at 5.5 years with the HOMA levels 2.5 years later. While very long-chain FA (VLCFA) with 20, 22 or 24 carbon atoms have been found to be related to lower diabetes risk and other beneficial effects on metabolic outcomes^61^, VLCFA with 26 carbon atoms were found to have positive associations with coronary artery disease and metabolic syndrome^62^. However, the biological role of these NEFA species is poorly investigated and further analyses replicating our findings in insulin-resistant patients would be important.

The major strength of this meta-analysis is the measurement of the metabolomics data from three studies using one and the same analytical platform. As the characteristics of the children were similar, it is reasonable to assume a one true effect across all studies – which is one assumption underlying the application of a fixed effects meta-analysis. On the other hand, this similarity might be considered a limitation because our conclusions are based on studying generally healthy children only. Further studies on subjects with different clinical characteristics are desirable, which may also increase the effect size, which is small in our analysis.

However, our study presents a unique design and effort for metabolomics analysis, since studies usually focus on one trial. Although some research groups used the same platform to analyze different trials, these trials are presented separately and to not share the same results. Another advantage is the number of metabolites compared to studies only focusing on one group of metabolites, in particular amino acids^32^, which should not be regarded as metabolomics studies. We could asses such a big number, since we used a targeted mass spectrometry platform, as performed with available kits^13,33^, which also present the opportunity to perform the same metabolomics analysis for different trials. The targeted approach also limits our study. Using our targeted metabolomics platform, we may also have missed other metabolites of importance which could have been identified in an untargeted approach, which have to possibility to cover a wider range of metabolites but face the challenge to assig metabolite names to the obtained signals^11,12,14,15^. Thus, the results include well known metabolites, like AA^11,12,14,15^, acylcarnitines^13,15^, hormones^11,14^, acylglycerols^12^ or sugars^12^ associated with childhood obesity. In our approach, we determined, among others, a wide number of potential polar lipids with different FA compositions, giving us the possibility also to quantify uncommon metabolites like SM 32:2, not covered by any of the mentioned approaches. Without SM 32:2, our results would have been comparable to the other metabolomic studies of childhood obesity, but SM 32:2 definitely outmatched the other metabolites.

However, we can not conclude on the direction of the association and give more mechanistic insights about what comes first, SM 32:2 or obesity. To answer this question, large cohorts with repeated and frequent blood sampling during childhood are needed. Therefore our proposed suggestions to interpret the findings about SM 32:2 include both possible “directions”. Using only European studies, our population is relatively homogenous, and along with the large sample number this facilitated identification of molecular markers of obesity and insulin resistance in childhood.

## Conclusion

In our meta-analysis, we identified SM 32:2 as a potential molecular marker for mechanistic alterations involved in the pathogenesis of obesity in healthy children. SM 32:2 was identified as molecular species containing myristic acid and sphingadienine (SM d18:2/14:0). SM 32:2 seems to play an outstanding role in the pathogenesis of obesity or related metabolic disorders compared to amino acids, acylcarnitines, fatty acids or other phospholipids. Thus, based on our findings we strongly encourage an implementation of SM 32:2 in other metabolomics protocols in this area. Future studies investigating the role of SM 32:2 will contribute to the understanding and the underlying pathways of childhood obesity and paving the way towards early intervention and personalized treatments. SM 32:2 may be a potential biochemical marker for the combined effect of genetic predisposition, high dietary intake of total energy, glycemic load, and linoleic acid. Thus, our finding opens up new perspectives on early interventions tackling the obesity pandemic.

## Supplementary information

Supplementary Information

## Data Availability

We declare our adherence to the journal policys on sharing data and materials and are open to sharing data for research purposes upon request, under conditions respecting the EU General Data Protection Regulation and the protection of personal rights of participating study subjects. Anonymized data can be made available by the authors upon request provided that accountable assurance is provided on strict compliance with protection of personal data of the human subjects included. The ethical committee of the Medical Faculty of LMU (Contact Dr. Beate Henrikus, Beate.Henrikus@med.uni-muenchen.de) and the Data Protection Officer of the LMU Medical Center (Gerhard Meyer, datenschutz@med.uni-muenchen.de) require that strict personal data protection of study participants is ensured in line with the European General Data Protection Regulation, and that the study database shall not be put in the public domain because of the risk that potential identification of study participants might occur. Rather, upon request by credible researchers and after signature of a written agreement ensuring personal data right protection, those data can be shared that are required for the proposed specific secondary research project. Request for data use should be sent Ms. Annina Herrmann, head of the Interdisciplinary Paediatric Study Center (Hauner iPSC), Dr. von Hauner Children’s Hospital, University of Munich Medicl Center, Annina.Herrmann@med.uni-muenchen.de.
