# Supplementary material for: An individual participant data meta-analysis on metabolomics profiles for obesity and insulin resistance in European children
Source: Sci Rep. 2019 Mar 25;9:5053. doi: 10.1038/s41598-019-41449-x (PMC6433919; doi:10.1038/s41598-019-41449-x)
Supplement: Supplementary file 1 — Supplementary Information [file 41598_2019_41449_MOESM1_ESM.pdf]

An individual participant data meta-analysis on metabolomics profiles for obesity and insulin resistance in European children. Supplementary Info.

Christian Hellmuth[1,a], Franca F Kirchberg[1,a], Stephanie Brandt[2], Anja Moß[2], Viola Walter[3], Dietrich Rothenbacher[4], Hermann Brenner[3], Veit Grote[1], Dariusz Gruszfeld[5], Piotr Socha[5], Ricardo Closa-Monasterolo[6], Joaquin Escribano[6], Veronica Luque[6], Elvira Verduci[7], Benedetta Mariani[7], Jean-Paul Langhendries[8], Pascale Poncelet[9], Joachim Heinrich[10,11], Irina Lehmann[12,13], Marie Standl[10], Olaf Uhl[1], Berthold Koletzko[1,b], Elisabeth Thiering[1,10,b], Martin Wabitsch[2,b]

[1] LMU - Ludwig-Maximilians-Universität München, Dr. von Hauner Children's Hospital, Div. Metabolic and Nutritional Medicine, 80336 Munich, Germany

[2] Division of Pediatric Endocrinology and Diabetes, Interdisciplinary Obesity Unit, Department of Pediatrics and Adolescent Medicine , University of Ulm , 89081 Ulm, Germany

[3] Division of Clinical Epidemiology and Aging Research, German Cancer Reasearch Center (DKFZ), 69120 Heidelberg, Germany

[4] Institute of Epidemiology and Medical Biometry, Ulm University, 89081 Ulm, Germany

[5] Neonatal Intensive Care Unit, Children's Memorial Health Institute, 04-736 Warsaw, Poland

[6] Pediatric Nutrition and Development Research Unit, Universitat Rovira I Virgili, IISPV, 43201 Reus, Spain

[7] Department of Paediatrics, San Paolo Hospital, University of Milan, 20142 Milano, Italy

[8] Centre Hospitalier Chrétien St Vincent, 4000 Liège-Rocourt, Belgium

[9] Hôpital Universitaire des enfants Reine Fabila, 1020 Bruxelles, Belgium

[10] Institute of Epidemiology I, Helmholtz Zentrum München- German Research Center for Environmental Health, 85764 Neuherberg, Germany

[11] Ludwig-Maximilians-Universität München, Institute and Outpatient Clinic for Occupational, Social and Environmental Medicine, 80336 Munich, Germany

[12] Department of Environmental Immunology/Core Facility Studies, Helmholtz Centre for Environmental Research – UFZ, 04318 Leipzig, Germany

[13] Berlin Institute of Health and Charité- Universitätsmedizin Berlin, Molecular Epidemiology Unit, Berlin, Germany

[a,b] contributed equally

Supplemental Figure 1

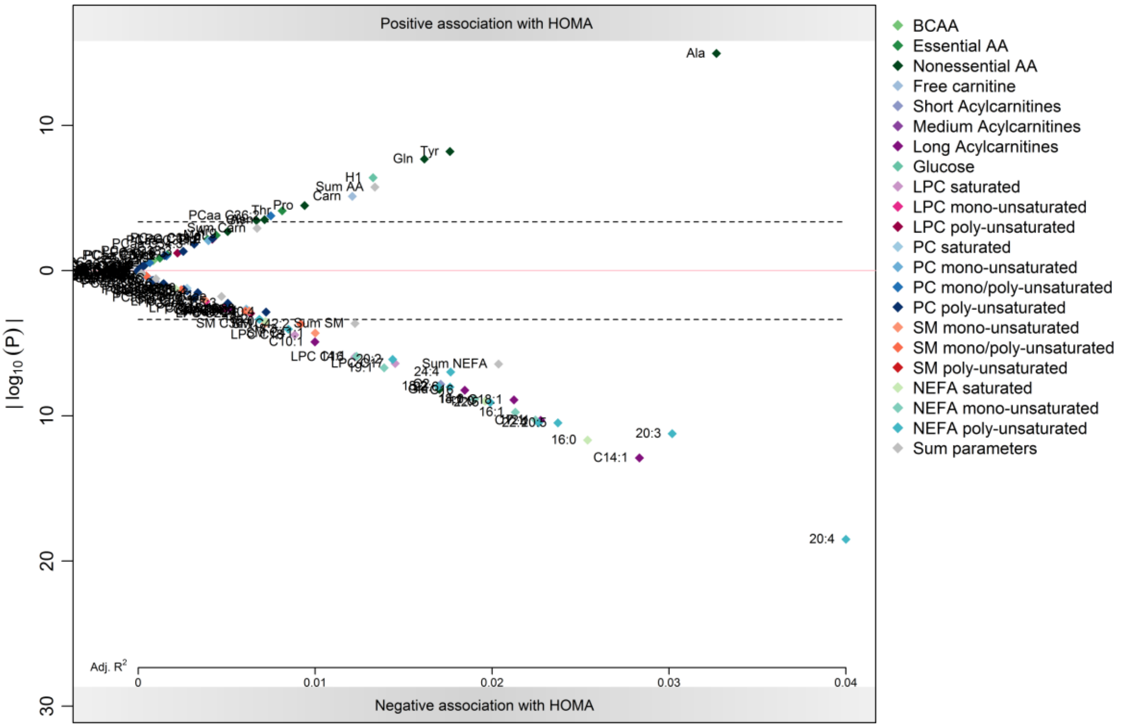

Supplemental Figure 1 Results of the individual participant meta-analysis of the association of metabolites with HOMA levels adjusted for BMI z-score. Log transformed p-values (y-axis) are plotted against adjusted R (x-axis), with positive associations in the upper part of the figure (above 0) and negative associations in the lower part (below 0). The dashed line indicates the Bonferroni-corrected significance level of 0.0004. Abbreviations: AA, amino acids; BCAA, branched-chain amino acids; LPC, lyso-phosphatidylcholine; NEFA, non-esterified fatty acid; PC, phosphatidylcholine; SM, sphingomyelin.

Supplemental Figure 2

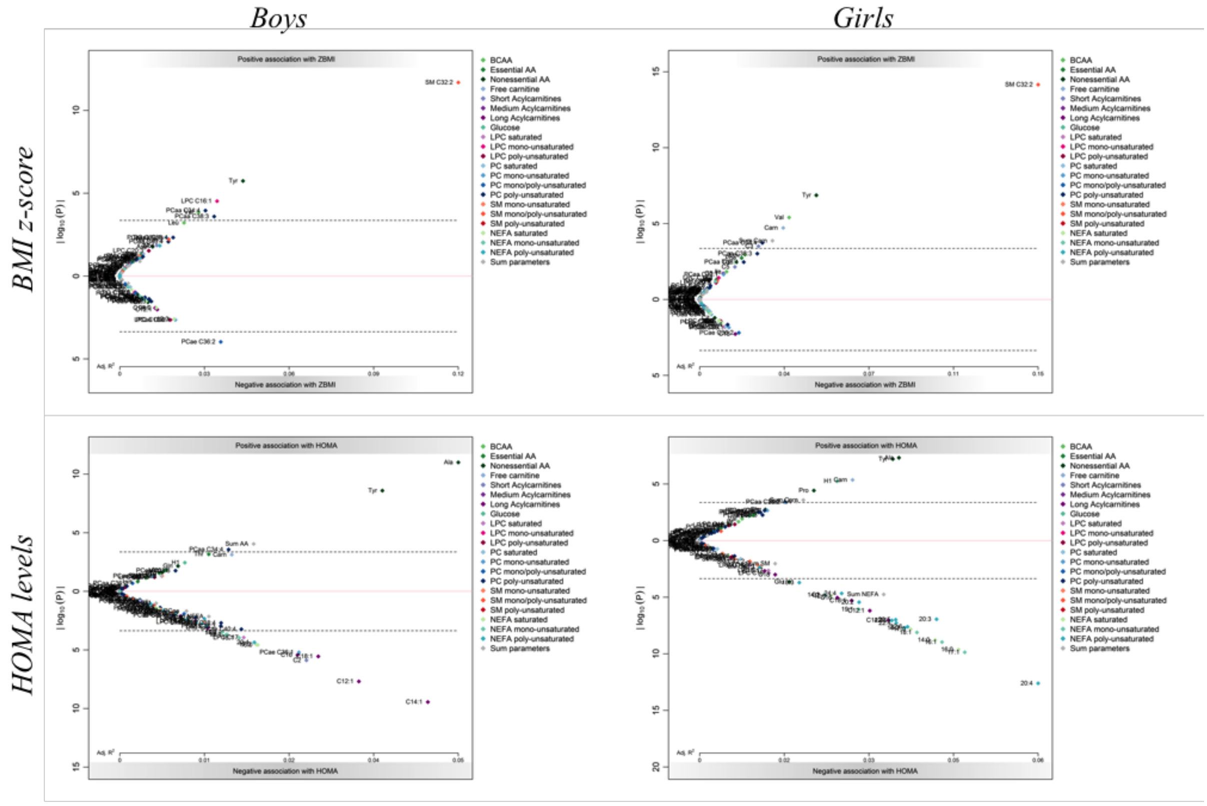

Supplemental Figure 2 *Results of the individual participant sex-stratified meta-analyses of metabolites associated with BMI z-score and HOMA-index. Log transformed p-values (y-axis) are plotted against adjusted R (x-axis), with positive associations in the upper part of the figure (above 0) and negative associations in the lower part (below 0). The dashed line indicates the Bonferroni-corrected significance level of 0.0004. Abbreviations: AA, amino acids; BCAA, branched-chain amino acids; LPC, lyso-phosphatidylcholine; NEFA, non-esterified fatty acid; PC, phosphatidylcholine; SM, sphingomyelin*

Supplemental Table 1 **Metabolites measured in each study and follow up, and used for meta-analyses**. AA, amino acids; Carn, Acylcarnitines; lyso.PC, lyso-phosphatidylcholine; NEFA, nonesterified fatty acid; PCaa, Diacyl-phosphatidylcholine; PCae, Alkyl-acyl-phosphatidylcholine; SM, sphingomyelin.

Metabolite			Inlcuded in meta-analysis							
	CHOP 5.5 years		CHOP 8 years		Ulm 8 years		GINIplus/LISA 10 years		Meta-Analysis	
	N	Mean (SD)	N	Mean (SD)	N	Mean (SD)	N	Mean (SD)	N	Mean (SD)
**Ala**	391	282 (87.2)	355	286 (68.9)	412	347 (81.5)	250	452 (113)	1408	332 (107)
**Arg**	389	64 (19.1)	355	70 (15.5)	411	83 (27.9)	250	124 (24.1)	1405	82 (30.6)
**Asn**	391	34 (11.1)	355	40 (9.45)	374	50 (9.67)	250	61 (13.2)	1370	45 (14.4)
**Asp**	389	7.8 (3.44)	355	7.9 (3.05)	374	17 (4.98)	249	37 (12.6)	1367	16 (12.6)
**Cit**	391	28 (8.2)	355	33 (6.75)	413	34 (9.45)	250	44 (10.1)	1409	34 (10.1)
**Gln**	391	261 (134)	354	497 (151)	412	598 (97.7)	221	592 (139)	1378	475 (192)
**Glu**	391	154 (87.5)	355	103 (92.8)	413	85 (19.1)	250	129 (61.1)	1409	117 (76.2)
**Gly**	391	204 (50.8)	355	208 (43)	411	265 (50.7)	250	350 (71.9)	1407	249 (75.7)
**His**	391	81 (20.5)	355	101 (25.2)	413	112 (20.1)	250	137 (24.5)	1409	105 (29.2)
**Hpro**					411	24 (10.3)				
**Ile**	391	59 (17.4)	355	56 (9.88)	372	64 (12.5)	250	79 (16.2)	1368	63 (16.4)
**Leu**	391	113 (28.2)	355	112 (17.1)	413	131 (22.3)	250	148 (29.8)	1409	124 (28.1)
**Lys**	391	155 (35.6)	355	74 (15.4)	412	170 (33.4)	250	203 (43.7)	1408	147 (56.2)
**Met**	391	19 (4.96)	355	23 (4.22)	412	26 (5.44)	250	27 (5.56)	1408	23 (5.83)
**Orn**	391	58 (20.3)	355	57 (14.5)	412	65 (16.2)	250	111 (32.6)	1408	69 (28.5)
**Phe**	391	51 (11.7)	354	57 (8.76)	413	63 (11.6)	250	87 (18.8)	1408	62 (17.7)
**Pro**	391	161 (58)	355	117 (37.8)	409	226 (74.4)	250	200 (71.1)	1405	176 (74.6)
**Ser**	390	87 (23.5)	354	133 (29)	412	151 (35.5)	250	146 (34.8)	1406	128 (40.4)
**Thr**	391	102 (31.3)	355	94 (22.5)	413	120 (27.5)	250	145 (34.6)	1409	113 (33.9)
**Trp**	390	43 (11.8)	355	56 (9.33)	412	69 (13.4)	250	66 (13.9)	1407	58 (16)
**Tyr**	391	61 (16.8)	354	65 (12.1)	412	75 (15.4)	250	84 (18.4)	1407	70 (17.7)
**Val**	391	222 (57.4)	355	212 (36.3)	413	250 (45)	250	270 (56.6)	1409	236 (53.5)
**H1**	390	5571 (1000)	355	5162 (1000)	411	5047 (1000)	252	4894 (1000)	1408	5194 (1000)
**Carn C2**	396	8.2 (3.68)	355	7.5 (2.52)	412	7 (2.72)	195	5.2 (2.13)	1358	7.2 (3.07)
**Carn C3**	396	0.34 (0.1)	352	0.61 (0.3)	413	0.39 (0.1)	207	0.28 (0.09)	1368	0.41 (0.2)
**Carn C3:1**	278	0.021 (0.007)	287	0.036 (0.02)						
**Carn C4**	396	0.22 (0.1)			413	0.2 (0.09)	163	0.18 (0.09)		
**Carn C4:0-OH**	278	0.15 (0.1)	238	0.081 (0.09)						
**Carn C4:1**	354	0.071 (0.03)								
**Carn C5**	396	0.11 (0.04)	355	0.18 (0.08)	406	0.11 (0.05)	153	0.1 (0.04)	1310	0.13 (0.06)
**Carn C5:0-OH**	319	0.0014 (0.0005)	355	0.0021 (0.001)						
**Carn C6**	320	0.066 (0.03)	355	0.1 (0.05)			251	0.11 (0.05)		
**Carn C6:1**	210	0.011 (0.005)	355	0.027 (0.04)						
**Carn C8**	328	0.41 (0.2)	355	0.33 (0.2)			252	0.54 (0.3)		
**Carn C8:1**	396	0.075 (0.06)	355	0.072 (0.07)	408	0.22 (0.2)	251	0.12 (0.07)	1410	0.12 (0.1)
**Carn C9**	396	0.033 (0.01)	355	0.029 (0.02)			230	0.063 (0.03)		
**Carn C10**	395	0.43 (0.2)	355	0.55 (0.3)			251	0.7 (0.4)		
**Carn C10:1**	396	0.44 (0.2)	354	0.36 (0.2)	411	0.29 (0.2)	252	0.59 (0.3)	1413	0.4 (0.2)
**Carn C10:2**	396	0.059 (0.02)	355	0.044 (0.02)			153	0.15 (0.06)		
**Carn C12::DCarn C**	265	0.14 (0.09)								
**Carn C12**	395	0.21 (0.09)	355	0.22 (0.1)			251	0.35 (0.2)		
**Carn C12:1**	396	0.58 (0.2)	355	0.5 (0.3)	413	0.26 (0.2)	223	0.84 (0.4)	1387	0.51 (0.3)
**Carn C14**	396	0.074 (0.03)	355	0.062 (0.03)			162	0.069 (0.03)		
**Carn C14:1**	394	0.26 (0.1)	354	0.31 (0.2)	412	0.3 (0.1)	251	0.23 (0.1)	1411	0.28 (0.1)
**Carn C14:2**	396	0.051 (0.03)	355	0.063 (0.04)			163	0.093 (0.04)		
**Carn C14:2-OH**	253	0.025 (0.01)	353	0.018 (0.01)						
**Carn C15**	396	0.026 (0.01)	355	0.011 (0.005)						
**Carn C16**	395	0.095 (0.03)	355	0.087 (0.02)	411	0.1 (0.04)	230	0.1 (0.03)	1391	0.096 (0.03)
**Carn C16:0-OH**	258	0.016 (0.008)	289	0.0055 (0.002)						
**Carn C16:1**	395	0.065 (0.03)	355	0.056 (0.03)			200	0.051 (0.02)		
**Carn C16:2**	293	0.016 (0.006)	355	0.019 (0.01)						
**Carn C16:2-OH**	278	0.025 (0.009)	305	0.012 (0.005)						
**Carn C18**	396	0.043 (0.01)	355	0.029 (0.01)	408	0.046 (0.02)	189	0.058 (0.03)	1348	0.042 (0.02)
**Carn C18:1**	396	0.12 (0.05)	355	0.092 (0.03)	413	0.14 (0.05)	252	0.14 (0.05)	1416	0.12 (0.05)
**Carn C18:1-OH**	327	0.016 (0.008)	355	0.013 (0.007)						
**Carn C18:2**	396	0.065 (0.03)	353	0.058 (0.02)			178	0.068 (0.02)		

**Carn C18:2-OH**	252	0.0077 (0.003)	233	0.0057 (0.003)						
**Carn C20**	394	0.015 (0.005)	349	0.0085 (0.005)						
**Carn C20:1**	318	0.00045 (1.9e-04)	354	0.00033 (7.1e-05)						
**Carn C20:3**	396	0.015 (0.007)	355	0.0064 (0.002)						
**Carn C20:4**	396	0.00098 (0.0006)	305	0.00042 (1.5e-04)						
**Carn C22**	294	0.00094 (4.9e-04)	222	0.00044 (2.0e-04)						
**Carn C22:5**	279	0.0076 (0.003)	254	0.0024 (0.001)						
**Carn C22:6**	257	0.0022 (0.0009)	184	0.0031 (0.002)						
**Carn**	396	40 (9.46)	355	66 (20.2)	412	33 (6.87)	191	28 (6.48)	1354	43 (18.9)
**LPC C14**	247	1.7 (0.8)			413	1.2 (0.5)	252	2.2 (1.01)		
**LPC C15**	249	0.79 (0.4)			413	0.7 (0.2)	234	1.1 (0.4)		
**LPC C16**	395	77 (29)	355	82 (44.6)	413	57 (12.3)	252	81 (27.4)	1415	73 (32.1)
**LPC C16:1**	396	2.3 (1.04)	355	2.6 (1.22)	412	1.9 (0.5)	252	2.7 (0.9)	1415	2.3 (1)
**LPC C17**	394	1.4 (0.7)	289	1.7 (0.9)	413	1 (0.3)	252	1.6 (0.6)	1348	1.4 (0.7)
**LPC C18**	396	27 (11.7)	355	29 (16.2)	413	20 (4.68)	252	27 (9.49)	1416	25 (11.8)
**LPC C18:1**	396	18 (5.35)	355	20 (6.9)	413	15 (3.57)	252	17 (4.88)	1416	17 (5.58)
**LPC C18:2**	396	29 (10.2)	355	36 (12.5)	413	25 (7.63)	252	29 (9.21)	1416	30 (10.9)
**LPC C18:3**	355	0.54 (0.3)	221	0.56 (0.3)	412	0.51 (0.2)	234	0.6 (0.3)	1222	0.54 (0.3)
**LPC C20:1**	203	0.41 (0.3)			413	0.27 (0.08)				
**LPC C20:3**	394	2.4 (1)	288	2.8 (0.9)	411	1.8 (0.4)	252	2.2 (0.7)	1345	2.3 (0.9)
**LPC C20:4**	396	6.8 (2.24)	355	7.9 (2.43)	411	4.4 (1.06)	252	6.4 (2.01)	1414	6.3 (2.38)
**LPC C20:5**	324	0.43 (0.2)	170	0.71 (0.3)	411	0.41 (0.2)	249	0.42 (0.2)	1154	0.46 (0.2)
**LPC C22:6**	320	1.8 (0.8)	253	1.9 (0.7)	412	1.3 (0.4)	234	1.6 (0.6)	1219	1.6 (0.7)
**LPC C18:6**			181	0.39 (0.2)	413	0.23 (0.07)	152	0.35 (0.1)		
**LPC C22:5**			154	0.82 (0.3)	413	0.83 (0.2)	187	0.56 (0.2)		
**LPC C15:1**					413	0.058 (0.02)				
**LPC C17:1**					413	0.26 (0.07)	234	0.35 (0.1)		
**LPC C20**					413	0.2 (0.07)				
**LPC C20:2**					413	0.26 (0.08)	135	0.32 (0.2)		
**LPC C22:4**					413	0.36 (0.1)				
**PCaa C18**					413	0.15 (0.04)				
**PCaa C18:1**					413	0.1 (0.04)				
**PCaa C20:4**	216	0.12 (0.06)								
**PCaa C28:2**	324	0.1 (0.05)								
**PCaa C30**	396	3.4 (1.62)			413	3.7 (1.57)	252	3.9 (1.39)		
**PCaa C30:2**	395	0.82 (0.2)			413	1.4 (0.5)	178	0.82 (0.3)		
**PCaa C30:3**	395	0.22 (0.08)					173	0.26 (0.09)		
**PCaa C30:4**	168	0.092 (0.03)					126	0.1 (0.06)		
**PCaa C30:5**			180	0.0043 (0.003)						
**PCaa C32**	396	13 (3.9)	355	14 (3.59)	413	19 (7.1)	252	14 (3.11)	1416	15 (5.55)
**PCaa C32:1**	396	12 (5.42)	289	13 (6.33)	413	18 (6.51)	252	16 (5.91)	1350	15 (6.47)
**PCaa C32:2**	396	2.9 (1.07)			413	3.9 (1.64)	252	3.6 (1.3)		
**PCaa C32:3**					413	0.51 (0.2)	197	0.78 (0.2)		
**PCaa C34**					411	5.3 (2.45)				
**PCaa C34:1**	396	191 (53.3)	355	206 (55.5)	412	224 (49.6)	252	197 (47)	1415	206 (53.2)
**PCaa C34:2**	396	308 (78)	289	339 (88)	412	404 (91.7)	252	337 (81.1)	1349	350 (93.2)
**PCaa C34:3**	394	13 (4.79)	355	15 (5.23)	413	14 (4.38)	252	17 (4.93)	1414	14 (4.96)
**PCaa C34:4**	396	1.5 (0.6)	305	1.7 (0.7)	413	1.8 (0.8)	252	1.9 (0.7)	1366	1.7 (0.7)
**PCaa C34:5**					413	0.22 (0.1)	167	0.21 (0.1)		
**PCaa C34:6**					398	0.11 (0.05)				
**PCaa C36**	277	3.3 (2.61)			412	3.1 (1)	192	3.9 (2.17)		
**PCaa C36:1**	396	43 (15.1)			413	58 (17.8)	252	46 (12.7)		
**PCaa C36:2**	396	234 (58.4)	355	252 (80)	412	237 (44.7)	252	239 (59.1)	1415	240 (61.6)
**PCaa C36:3**	396	116 (29.9)	355	115 (27.2)	413	117 (21.8)	252	119 (30.1)	1416	117 (27.1)
**PCaa C36:4**	396	127 (39.5)	289	136 (38)	413	164 (33.5)	252	147 (38.4)	1350	144 (40)
**PCaa C36:5**	394	9.4 (3.72)	286	11 (5.85)	413	13 (5.78)	251	13 (5.53)	1344	12 (5.48)
**PCaa C36:6**	325	1 (0.7)			413	0.65 (0.3)	231	1.1 (0.5)		
**PCaa C38**	318	3.2 (1.29)	166	3.7 (1.71)	413	2.4 (0.7)	252	3.2 (1.2)	1149	3 (1.25)
**PCaa C38:1**					409	1.8 (0.8)				
**PCaa C38:2**					413	5.7 (1.72)	117	6.9 (2)		
**PCaa C38:3**	396	37 (11.3)	167	37 (10.5)	412	36 (8.81)	252	44 (13.6)	1227	38 (11.3)
**PCaa C38:4**	396	93 (29.2)	184	100 (31.2)	413	113 (27.3)	252	102 (29.3)	1245	103 (30)
**PCaa C38:5**	396	37 (10.6)	239	34 (10.3)	413	48 (13)	252	45 (13.5)	1300	41 (13.2)
**PCaa C38:6**	396	52 (17.6)	355	54 (22)	413	56 (16.1)	252	60 (20.8)	1416	55 (19.2)

Supplemental Table 2 Associations of metabolites with BMI z-score in all studies and follow-ups, and in the meta-analysis. Associations between BMI z-score and metabolites for each study follow-up visit were calculated using linear regression without further covariate adjustment and individual participant meta-analysis. Statistical significance was evaluated using Bonferroni corrected p-values (p.adjust) < 0.05 (with dark grey background). AA, amino acids; Carn, Acylcarnitines; lyso.PC, lyso-phosphatidylcholine; NEFA, nonesterified fatty acid; PCaa, Diacyl-phosphatidylcholine; PCae, Alkyl-acyl-phosphatidylcholine; SM, sphingomyelin

CHOP 5.5 years

Metabolite	N	Estimate	Std..Error	P	P.adjusted	PartialRsquared
Ala	386	0.000	0.001	5.29E-01	1.00E+00	0.001
Arg	384	0.001	0.003	7.79E-01	1.00E+00	0.000
Asn	386	-0.008	0.005	6.88E-02	1.00E+00	0.009
Asp	384	0.026	0.015	8.15E-02	1.00E+00	0.008
Cit	386	-0.006	0.006	3.14E-01	1.00E+00	0.003
Gln	386	-0.001	0.000	1.49E-02	1.00E+00	0.015
Glu	386	0.000	0.001	9.03E-01	1.00E+00	0.000
Gly	386	-0.002	0.001	1.06E-01	1.00E+00	0.007
His	386	-0.001	0.002	6.43E-01	1.00E+00	0.001
Ile	386	0.004	0.003	1.35E-01	1.00E+00	0.006
Leu	386	0.002	0.002	4.08E-01	1.00E+00	0.002
Lys	386	0.000	0.001	9.02E-01	1.00E+00	0.000
Met	386	0.013	0.010	2.21E-01	1.00E+00	0.004
Orn	386	0.000	0.003	8.89E-01	1.00E+00	0.000
Phe	386	0.002	0.004	6.71E-01	1.00E+00	0.000
Pro	386	-0.001	0.001	3.43E-01	1.00E+00	0.002
Ser	385	-0.001	0.002	7.59E-01	1.00E+00	0.000
Thr	386	0.004	0.002	2.96E-02	1.00E+00	0.012
Trp	385	0.004	0.004	3.22E-01	1.00E+00	0.003
Tyr	386	0.008	0.003	7.34E-03	1.00E+00	0.019
Val	386	0.001	0.001	2.27E-01	1.00E+00	0.004
H1	385	0.000	0.000	7.00E-02	1.00E+00	0.009
Carn	391	0.022	0.005	2.53E-05	5.90E-03	0.045
Carn C2	391	-0.017	0.014	2.14E-01	1.00E+00	0.004
Carn C3	391	1.519	0.506	2.87E-03	6.69E-01	0.023
Carn C3:1	274	13.944	8.695	1.10E-01	1.00E+00	0.009
Carn C4	391	0.292	0.466	5.31E-01	1.00E+00	0.001
Carn C4:0:OH	274	-1.316	0.623	3.55E-02	1.00E+00	0.016
Carn C4:1	349	-0.517	1.549	7.38E-01	1.00E+00	0.000
Carn C5	391	3.789	1.336	4.80E-03	1.00E+00	0.020
Carn C5:0:OH	315	203.492	106.725	5.75E-02	1.00E+00	0.011
Carn C6	316	-1.257	1.699	4.60E-01	1.00E+00	0.002
Carn C6:1	206	-4.627	15.613	7.67E-01	1.00E+00	0.000
Carn C8	323	-0.423	0.338	2.13E-01	1.00E+00	0.005

Carn C8:1	391	0.025	0.910	9.78E-01	1.00E+00	0.000
Carn C9	391	-0.626	3.458	8.57E-01	1.00E+00	0.000
Carn C10	390	-0.302	0.215	1.62E-01	1.00E+00	0.005
Carn C10:1	391	0.440	0.288	1.28E-01	1.00E+00	0.006
Carn C10:2	391	1.154	2.589	6.56E-01	1.00E+00	0.001
Carn C12::DC	260	-0.371	0.684	5.88E-01	1.00E+00	0.001
Carn C12	390	-0.554	0.586	3.45E-01	1.00E+00	0.002
Carn C12:1	391	-0.024	0.215	9.10E-01	1.00E+00	0.000
Carn C14	391	-2.002	1.653	2.26E-01	1.00E+00	0.004
Carn C14:1	389	-0.374	0.419	3.73E-01	1.00E+00	0.002
Carn C14:2	391	0.324	1.911	8.66E-01	1.00E+00	0.000
Carn C14:2:OH	249	6.356	5.678	2.64E-01	1.00E+00	0.005
Carn C15	391	0.825	4.643	8.59E-01	1.00E+00	0.000
Carn C16	390	0.952	1.783	5.94E-01	1.00E+00	0.001
Carn C16:OH	254	-2.662	7.920	7.37E-01	1.00E+00	0.000
Carn C16:1	390	-0.840	1.932	6.64E-01	1.00E+00	0.000
Carn C16:2	288	8.499	10.397	4.14E-01	1.00E+00	0.002
Carn C16:2:OH	274	10.836	6.467	9.50E-02	1.00E+00	0.010
Carn C18	391	-4.053	3.472	2.44E-01	1.00E+00	0.003
Carn C18:1	391	0.528	1.121	6.38E-01	1.00E+00	0.001
Carn C18:1:OH	322	-2.686	7.337	7.15E-01	1.00E+00	0.000
Carn C18:2	391	3.106	1.562	4.75E-02	1.00E+00	0.010
Carn C18:2:OH	248	20.565	21.647	3.43E-01	1.00E+00	0.004
Carn C20	389	2.501	11.174	8.23E-01	1.00E+00	0.000
Carn C20:1	314	219.757	290.383	4.50E-01	1.00E+00	0.002
Carn C20:3	391	12.720	7.330	8.35E-02	1.00E+00	0.008
Carn C20:4	391	90.283	83.205	2.79E-01	1.00E+00	0.003
Carn C22	289	-17.306	119.032	8.85E-01	1.00E+00	0.000
Carn C22:5	275	17.568	17.326	3.11E-01	1.00E+00	0.004
Carn C22:6	253	73.036	68.969	2.91E-01	1.00E+00	0.004
LPC C14	245	0.055	0.080	4.93E-01	1.00E+00	0.002
LPC C15	246	-0.407	0.168	1.61E-02	1.00E+00	0.024
LPC C16	390	0.000	0.002	9.65E-01	1.00E+00	0.000
LPC C16:1	391	0.002	0.049	9.64E-01	1.00E+00	0.000
LPC C17	389	-0.102	0.073	1.64E-01	1.00E+00	0.005
LPC C18	391	0.001	0.004	8.23E-01	1.00E+00	0.000
LPC C18:1	391	-0.010	0.010	2.91E-01	1.00E+00	0.003
LPC C18:2	391	0.000	0.005	9.44E-01	1.00E+00	0.000
LPC C18:3	350	-0.137	0.163	3.99E-01	1.00E+00	0.002
LPC C20:1	202	-0.729	0.269	7.20E-03	1.00E+00	0.036
LPC C20:3	389	0.050	0.051	3.27E-01	1.00E+00	0.002
LPC C20:4	391	0.012	0.023	6.05E-01	1.00E+00	0.001
LPC C20:5	320	0.018	0.242	9.41E-01	1.00E+00	0.000

LPC C22:6	316	-0.082	0.074	2.69E-01	1.00E+00	0.004
PCaa C20:4	212	0.966	1.152	4.03E-01	1.00E+00	0.003
PCaa C28:2	320	2.872	1.128	1.13E-02	1.00E+00	0.020
PCaa C30	391	0.055	0.031	7.77E-02	1.00E+00	0.008
PCaa C30:2	390	0.405	0.210	5.49E-02	1.00E+00	0.009
PCaa C30:3	390	2.070	0.666	2.02E-03	4.70E-01	0.024
PCaa C30:4	168	5.701	2.247	1.21E-02	1.00E+00	0.037
PCaa C32	391	0.035	0.013	7.46E-03	1.00E+00	0.018
PCaa C32:1	391	0.030	0.009	1.23E-03	2.86E-01	0.027
PCaa C32:2	391	0.142	0.047	2.64E-03	6.14E-01	0.023
PCaa C34:1	391	0.001	0.001	1.66E-01	1.00E+00	0.005
PCaa C34:2	391	0.001	0.001	8.24E-02	1.00E+00	0.008
PCaa C34:3	389	0.025	0.011	1.83E-02	1.00E+00	0.014
PCaa C34:4	391	0.243	0.083	3.75E-03	8.74E-01	0.021
PCaa C36	273	0.069	0.023	2.79E-03	6.51E-01	0.033
PCaa C36:1	391	0.006	0.003	6.16E-02	1.00E+00	0.009
PCaa C36:2	391	0.001	0.001	1.74E-01	1.00E+00	0.005
PCaa C36:3	391	0.003	0.002	1.29E-01	1.00E+00	0.006
PCaa C36:4	391	0.002	0.001	1.30E-01	1.00E+00	0.006
PCaa C36:5	389	0.019	0.014	1.75E-01	1.00E+00	0.005
PCaa C36:6	321	0.042	0.075	5.80E-01	1.00E+00	0.001
PCaa C38	314	0.094	0.043	2.98E-02	1.00E+00	0.015
PCaa C38:3	391	0.011	0.004	1.61E-02	1.00E+00	0.015
PCaa C38:4	391	0.004	0.002	2.17E-02	1.00E+00	0.013
PCaa C38:5	391	0.000	0.005	9.36E-01	1.00E+00	0.000
PCaa C38:6	391	-0.002	0.003	4.83E-01	1.00E+00	0.001
PCaa C40	280	0.143	0.088	1.06E-01	1.00E+00	0.009

PCaa C40:4	390	0.097	0.040	1.53E-02	1.00E+00	0.015
PCaa C40:5	391	0.023	0.014	1.20E-01	1.00E+00	0.006
PCaa C40:6	391	-0.001	0.007	9.24E-01	1.00E+00	0.000
PCaa C42	245	0.213	0.157	1.76E-01	1.00E+00	0.008
PCaa C42:1	209	0.628	0.326	5.53E-02	1.00E+00	0.018
PCaa C42:6	202	0.206	0.202	3.10E-01	1.00E+00	0.005
PCaa C43:6	249	0.072	0.116	5.37E-01	1.00E+00	0.002
PCaa C44:12	391	0.127	0.085	1.35E-01	1.00E+00	0.006
PCae C30:2	280	0.317	0.486	5.15E-01	1.00E+00	0.002
PCae C32	391	0.131	0.062	3.45E-02	1.00E+00	0.011
PCae C32:1	391	0.109	0.050	3.10E-02	1.00E+00	0.012
PCae C32:2	206	0.383	0.190	4.53E-02	1.00E+00	0.020
PCae C34	391	0.040	0.085	6.39E-01	1.00E+00	0.001
PCae C34:1	391	0.031	0.018	8.34E-02	1.00E+00	0.008
PCae C34:2	391	0.026	0.016	1.01E-01	1.00E+00	0.007
PCae C34:3	391	0.044	0.018	1.64E-02	1.00E+00	0.015
PCae C34:4	246	1.122	0.462	1.58E-02	1.00E+00	0.024
PCae C36	253	0.061	0.100	5.41E-01	1.00E+00	0.001
PCae C36:1	390	0.013	0.008	1.19E-01	1.00E+00	0.006
PCae C36:2	390	0.008	0.010	4.31E-01	1.00E+00	0.002
PCae C36:3	391	0.040	0.018	3.07E-02	1.00E+00	0.012
PCae C36:4	390	0.014	0.009	1.32E-01	1.00E+00	0.006
PCae C36:5	391	0.019	0.012	1.10E-01	1.00E+00	0.007
PCae C36:6	178	-0.075	0.179	6.75E-01	1.00E+00	0.001
PCae C38	315	0.046	0.037	2.21E-01	1.00E+00	0.005
PCae C38:2	312	0.026	0.012	3.52E-02	1.00E+00	0.014
PCae C38:3	389	0.030	0.012	9.55E-03	1.00E+00	0.017
PCae C38:4	391	0.008	0.012	5.11E-01	1.00E+00	0.001
PCae C38:5	391	0.013	0.010	1.74E-01	1.00E+00	0.005

PCae C38:6	391	0.038	0.021	6.95E-02	1.00E+00	0.008
PCae C40	391	0.008	0.017	6.46E-01	1.00E+00	0.001
PCae C40:1	237	0.189	0.064	3.33E-03	7.76E-01	0.036
PCae C40:3	320	0.030	0.016	6.27E-02	1.00E+00	0.011
PCae C40:4	351	0.075	0.026	3.94E-03	9.17E-01	0.024
PCae C40:5	391	0.027	0.028	3.29E-01	1.00E+00	0.002
PCae C40:6	391	-0.014	0.036	6.90E-01	1.00E+00	0.000
PCae C42:2	323	0.165	0.091	7.27E-02	1.00E+00	0.010
PCae C42:3	280	0.037	0.076	6.28E-01	1.00E+00	0.001
PCae C42:4	198	0.411	0.157	9.44E-03	1.00E+00	0.034
PCae C42:5	316	0.052	0.059	3.79E-01	1.00E+00	0.002
PCae C42:6	390	0.091	0.095	3.39E-01	1.00E+00	0.002
SM C21:2	351	1.084	2.117	6.09E-01	1.00E+00	0.001
SM C32:1	391	0.068	0.021	1.17E-03	2.73E-01	0.027
SM C32:2	351	1.825	0.263	2.06E-11	4.80E-09	0.121
SM C33:1	391	0.045	0.037	2.30E-01	1.00E+00	0.004
SM C33:3	185	-0.427	0.915	6.41E-01	1.00E+00	0.001
SM C34:1	390	0.004	0.002	1.00E-01	1.00E+00	0.007
SM C34:2	390	0.075	0.015	6.77E-07	1.58E-04	0.062
SM C34:4	215	-0.900	3.296	7.85E-01	1.00E+00	0.000
SM C35:1	390	0.118	0.062	5.79E-02	1.00E+00	0.009
SM C35:2	203	-0.348	0.501	4.88E-01	1.00E+00	0.002
SM C36:1	389	0.023	0.011	2.76E-02	1.00E+00	0.012
SM C36:2	390	0.081	0.019	3.52E-05	8.21E-03	0.043
SM C36:3	185	0.678	0.211	1.55E-03	3.62E-01	0.053
SM C37:1	203	0.074	0.059	2.13E-01	1.00E+00	0.008
SM C37:3	212	0.007	0.395	9.87E-01	1.00E+00	0.000
SM C38:2	391	0.029	0.013	2.44E-02	1.00E+00	0.013
SM C39:1	316	0.078	0.032	1.57E-02	1.00E+00	0.018
SM C40:2	243	0.011	0.008	1.85E-01	1.00E+00	0.007
SM C40:4	313	0.160	0.086	6.42E-02	1.00E+00	0.011

SM C40:5	351	0.017	0.157	9.16E-01	1.00E+00	0.000
SM C41:1	390	0.025	0.011	3.16E-02	1.00E+00	0.012
SM C41:2	390	0.039	0.017	1.78E-02	1.00E+00	0.014
SM C42:1	391	0.019	0.008	1.53E-02	1.00E+00	0.015
SM C42:2	390	0.010	0.005	2.25E-02	1.00E+00	0.013
SM C42:3	321	0.013	0.009	1.66E-01	1.00E+00	0.006
SM C42:6	391	0.016	0.056	7.73E-01	1.00E+00	0.000
SM C43	176	-0.700	0.316	2.80E-02	1.00E+00	0.027
SM C43:1	205	0.310	0.117	8.54E-03	1.00E+00	0.034
SM C43:2	320	0.047	0.065	4.70E-01	1.00E+00	0.002
SM C44:6	238	0.086	0.152	5.73E-01	1.00E+00	0.001
SM C47:6	323	11.810	9.305	2.05E-01	1.00E+00	0.005
NEFA 12:0	178	-0.004	0.019	8.39E-01	1.00E+00	0.000
NEFA 14:0	321	-0.017	0.006	5.31E-03	1.00E+00	0.024
NEFA 14:1	388	0.004	0.010	6.45E-01	1.00E+00	0.001
NEFA 15:0	178	-0.183	0.075	1.58E-02	1.00E+00	0.033
NEFA 16:0	245	-0.002	0.001	2.23E-02	1.00E+00	0.021
NEFA 16:1	388	-0.006	0.003	4.17E-02	1.00E+00	0.011
NEFA 16:2	310	0.017	0.103	8.68E-01	1.00E+00	0.000
NEFA 17:0	168	0.014	0.032	6.71E-01	1.00E+00	0.001
NEFA 17:1	388	-0.012	0.025	6.28E-01	1.00E+00	0.001
NEFA 17:2	168	-1.210	1.198	3.14E-01	1.00E+00	0.006
NEFA 18:1	388	-0.001	0.000	4.98E-03	1.00E+00	0.020
NEFA 18:2	388	-0.002	0.001	8.73E-02	1.00E+00	0.008
NEFA 18:3	388	-0.046	0.015	1.92E-03	4.48E-01	0.025
NEFA 18:4	168	-1.982	0.627	1.86E-03	4.35E-01	0.057
NEFA 19:1	308	-0.224	0.135	9.82E-02	1.00E+00	0.009
NEFA 20:1	388	-0.090	0.031	4.20E-03	9.78E-01	0.021
NEFA 20:2	388	0.001	0.017	9.42E-01	1.00E+00	0.000
NEFA 20:3	388	0.028	0.034	4.07E-01	1.00E+00	0.002
NEFA 20:4	387	-0.014	0.011	2.05E-01	1.00E+00	0.004
NEFA 20:5	307	-0.068	0.068	3.20E-01	1.00E+00	0.003

NEFA 22:3	235	-1.175	2.008	5.59E-01	1.00E+00	0.001
NEFA 22:4	388	-0.190	0.145	1.91E-01	1.00E+00	0.004
NEFA 22:5	385	-0.118	0.074	1.12E-01	1.00E+00	0.007
NEFA 22:6	388	-0.005	0.030	8.62E-01	1.00E+00	0.000
NEFA 24:0	168	-0.446	0.515	3.87E-01	1.00E+00	0.005
NEFA 24:1	388	-0.195	0.205	3.42E-01	1.00E+00	0.002
NEFA 24:2	168	8.304	4.056	4.22E-02	1.00E+00	0.025
NEFA 24:3	242	2.050	2.946	4.87E-01	1.00E+00	0.002
NEFA 24:4	388	0.595	2.557	8.16E-01	1.00E+00	0.000
NEFA 24:6	167	-14.705	7.739	5.92E-02	1.00E+00	0.021
NEFA 26:0	335	0.711	0.309	2.21E-02	1.00E+00	0.016
NEFA 26:1	313	0.636	0.341	6.32E-02	1.00E+00	0.011
NEFA 26:2	388	0.899	0.424	3.46E-02	1.00E+00	0.012
NEFA 26:3	309	2.714	1.496	7.06E-02	1.00E+00	0.011
NEFA 26:4	388	1.362	5.483	8.04E-01	1.00E+00	0.000
Pyruvate	383	0.000	0.000	5.02E-01	1.00E+00	0.001
Lactic_acid	388	0.000	0.000	7.36E-01	1.00E+00	0.000
Fumarate	381	-0.245	0.172	1.56E-01	1.00E+00	0.005
3-Methyl-2-oxobutanoate	383	0.000	0.004	9.42E-01	1.00E+00	0.000
Succininate	386	0.003	0.015	8.52E-01	1.00E+00	0.000
Methylmalonate	388	0.932	0.712	1.91E-01	1.00E+00	0.004
Taurine	386	0.001	0.001	4.76E-01	1.00E+00	0.001
3-Methyl-2-oxovalerate	387	-0.002	0.004	6.81E-01	1.00E+00	0.000
4-Methyl-2-oxovalerate	382	0.001	0.002	6.97E-01	1.00E+00	0.000
Malate	384	0.000	0.029	9.92E-01	1.00E+00	0.000
Alpha-ketoglutarate	377	-0.002	0.009	8.00E-01	1.00E+00	0.000
Alpha-aminoadipate	386	-0.012	0.313	9.69E-01	1.00E+00	0.000
Isocitrate	388	-0.004	0.009	6.48E-01	1.00E+00	0.001
Citrate	388	0.000	0.001	5.52E-01	1.00E+00	0.001
Beta-hydroxybutyrate	150	-0.002	0.002	3.50E-01	1.00E+00	0.006
Sum AA	383	0.000	0.000	8.02E-01	1.00E+00	0.000
Sum Carn	388	0.017	0.005	5.63E-04	1.31E-01	0.030
Sum LPC	206	-0.005	0.001	1.41E-03	3.27E-01	0.049
Sum PCaa	311	0.000	0.000	1.02E-01	1.00E+00	0.009
Sum PCae	274	0.004	0.002	8.66E-03	1.00E+00	0.025
Sum SM	349	0.006	0.002	2.53E-03	5.89E-01	0.026
Sum NEFA	99	-0.001	0.000	2.17E-02	1.00E+00	0.053

CHOP 8 years

Metabolite	N	Estimate	Std..Error	P	P.adjusted	PartialRsquared
Ala	354	0.001	0.001	1.17E-01	1.00E+00	0.007
Arg	354	-0.005	0.004	1.44E-01	1.00E+00	0.006
Asn	354	-0.011	0.006	5.92E-02	1.00E+00	0.010
Asp	354	0.018	0.019	3.30E-01	1.00E+00	0.003
Cit	354	-0.022	0.008	8.16E-03	1.00E+00	0.020
Gln	353	-0.001	0.000	2.78E-03	5.28E-01	0.025
Glu	354	0.001	0.001	2.64E-01	1.00E+00	0.004
Gly	354	-0.004	0.001	1.06E-03	2.01E-01	0.030
His	354	0.000	0.002	9.13E-01	1.00E+00	0.000
Ile	354	0.013	0.006	2.45E-02	1.00E+00	0.014
Leu	354	0.009	0.003	4.62E-03	8.77E-01	0.023
Lys	354	0.001	0.004	6.90E-01	1.00E+00	0.000
Met	354	0.008	0.014	5.66E-01	1.00E+00	0.001
Orn	354	0.004	0.004	3.20E-01	1.00E+00	0.003
Phe	353	0.013	0.006	4.90E-02	1.00E+00	0.011
Pro	354	0.001	0.002	5.34E-01	1.00E+00	0.001
Ser	353	-0.003	0.002	8.71E-02	1.00E+00	0.008
Thr	354	0.002	0.003	4.53E-01	1.00E+00	0.002
Trp	354	0.015	0.006	1.25E-02	1.00E+00	0.018
Tyr	353	0.027	0.005	4.16E-09	7.90E-07	0.094
Val	354	0.006	0.002	1.83E-04	3.47E-02	0.039
H1	354	0.000	0.000	6.10E-01	1.00E+00	0.001
Carn	354	0.007	0.003	8.78E-03	1.00E+00	0.019
Carn C2	354	-0.029	0.023	2.04E-01	1.00E+00	0.005
Carn C3	351	0.435	0.186	2.01E-02	1.00E+00	0.015
Carn C3:1	286	2.428	2.803	3.87E-01	1.00E+00	0.003
Carn C4:0:OH	237	-1.533	0.761	4.50E-02	1.00E+00	0.017
Carn C5	354	2.141	0.753	4.72E-03	8.97E-01	0.000
Carn C5:0:OH	354	7.843	42.020	8.52E-01	1.00E+00	0.022
Carn C6	354	-0.100	1.215	9.34E-01	1.00E+00	0.000
Carn C6:1	354	-1.112	1.569	4.79E-01	1.00E+00	0.001
Carn C8	354	-0.716	0.344	3.82E-02	1.00E+00	0.012

Carn C8:1	354	0.073	0.833	9.30E-01	1.00E+00	0.000
Carn C9	354	-1.354	3.258	6.78E-01	1.00E+00	0.000
Carn C10	354	-0.550	0.202	6.83E-03	1.00E+00	0.021
Carn C10:1	353	0.284	0.337	3.99E-01	1.00E+00	0.002
Carn C10:2	354	-0.123	2.379	9.59E-01	1.00E+00	0.000
Carn C12	354	-0.933	0.516	7.15E-02	1.00E+00	0.009
Carn C12:1	354	-0.271	0.216	2.12E-01	1.00E+00	0.004
Carn C14	354	-1.535	2.187	4.83E-01	1.00E+00	0.001
Carn C14:1	353	-0.342	0.353	3.34E-01	1.00E+00	0.003
Carn C14:2	354	0.511	1.645	7.56E-01	1.00E+00	0.000
Carn C14:2:OH	352	0.807	5.164	8.76E-01	1.00E+00	0.000
Carn C15	354	-3.169	11.919	7.90E-01	1.00E+00	0.000
Carn C16	354	-1.570	2.671	5.57E-01	1.00E+00	0.001
Carn C16:0:OH	288	15.689	31.513	6.19E-01	1.00E+00	0.001
Carn C16:1	354	-2.983	2.291	1.94E-01	1.00E+00	0.005
Carn C16:2	354	-3.366	5.940	5.71E-01	1.00E+00	0.001
Carn C16:2:OH	304	2.239	12.062	8.53E-01	1.00E+00	0.000
Carn C18	354	-13.733	5.873	1.99E-02	1.00E+00	0.015
Carn C18:1	354	-2.416	1.735	1.65E-01	1.00E+00	0.005
Carn C18:1:OH	354	-15.211	7.964	5.70E-02	1.00E+00	0.010
Carn C18:2	352	2.686	2.878	3.51E-01	1.00E+00	0.002
Carn C18:2:OH	233	11.409	21.417	5.95E-01	1.00E+00	0.001
Carn C20	348	-44.661	11.154	7.62E-05	1.45E-02	0.044
Carn C20:1	353	393.839	814.564	6.29E-01	1.00E+00	0.001
Carn C20:3	354	61.928	30.974	4.63E-02	1.00E+00	0.011
Carn C20:4	304	602.091	412.904	1.46E-01	1.00E+00	0.007
Carn C22	221	-631.698	358.995	7.99E-02	1.00E+00	0.014
Carn C22:5	253	187.401	59.271	1.76E-03	3.35E-01	0.038
Carn C22:6	183	33.562	43.016	4.36E-01	1.00E+00	0.003
LPC C16	354	0.000	0.001	8.81E-01	1.00E+00	0.000
LPC C16:1	354	0.071	0.047	1.31E-01	1.00E+00	0.006
LPC C17	288	0.050	0.070	4.77E-01	1.00E+00	0.002
LPC C18	354	0.000	0.004	9.97E-01	1.00E+00	0.000
LPC C18:1	354	-0.023	0.008	5.11E-03	9.71E-01	0.022
LPC C18:2	354	-0.009	0.005	4.42E-02	1.00E+00	0.011
LPC C18:3	221	-0.156	0.249	5.31E-01	1.00E+00	0.002
LPC C18:6	180	-0.023	0.376	9.52E-01	1.00E+00	0.000
LPC C20:3	287	0.064	0.068	3.51E-01	1.00E+00	0.003
LPC C20:4	354	0.003	0.024	8.95E-01	1.00E+00	0.000
LPC C20:5	170	0.115	0.268	6.67E-01	1.00E+00	0.001

LPC C22:5	154	-0.042	0.310	8.94E-01	1.00E+00	0.000
LPC C22:6	252	-0.154	0.100	1.22E-01	1.00E+00	0.010
PCaa C30:5	179	53.182	26.617	4.72E-02	1.00E+00	0.022
PCaa C32	354	-0.040	0.016	1.13E-02	1.00E+00	0.018
PCaa C32:1	288	0.010	0.010	3.09E-01	1.00E+00	0.004
PCaa C34:1	354	-0.004	0.001	5.02E-04	9.54E-02	0.034
PCaa C34:2	289	-0.002	0.001	1.88E-02	1.00E+00	0.019
PCaa C34:3	354	-0.011	0.011	3.12E-01	1.00E+00	0.003
PCaa C34:4	304	0.263	0.089	3.27E-03	6.21E-01	0.028
PCaa C36:2	354	-0.001	0.001	4.16E-01	1.00E+00	0.002
PCaa C36:3	354	-0.003	0.002	1.19E-01	1.00E+00	0.007
PCaa C36:4	289	-0.003	0.002	1.32E-01	1.00E+00	0.008
PCaa C36:5	285	-0.007	0.011	4.92E-01	1.00E+00	0.002
PCaa C38	165	-0.018	0.047	7.10E-01	1.00E+00	0.001
PCaa C38:3	167	0.007	0.008	4.01E-01	1.00E+00	0.004
PCaa C38:4	183	0.006	0.003	2.50E-02	1.00E+00	0.027
PCaa C38:5	238	-0.007	0.007	2.86E-01	1.00E+00	0.005
PCaa C38:6	354	-0.006	0.003	1.41E-02	1.00E+00	0.017

PCaa C40:4	156	0.089	0.070	2.07E-01	1.00E+00	0.010
PCaa C40:5	171	-0.008	0.025	7.54E-01	1.00E+00	0.001
PCaa C40:6	353	-0.003	0.007	6.71E-01	1.00E+00	0.001
PCaa C44:12	173	-0.845	0.257	1.20E-03	2.27E-01	0.060
PCae C34:1	354	-0.041	0.018	2.46E-02	1.00E+00	0.014
PCae C34:2	287	-0.017	0.015	2.36E-01	1.00E+00	0.005
PCae C34:3	354	-0.034	0.016	3.69E-02	1.00E+00	0.012
PCae C36:1	239	-0.037	0.011	1.29E-03	2.46E-01	0.043
PCae C36:2	155	-0.077	0.020	1.69E-04	3.21E-02	0.089
PCae C36:3	354	-0.020	0.018	2.59E-01	1.00E+00	0.004
PCae C36:4	287	-0.001	0.009	9.07E-01	1.00E+00	0.000
PCae C36:5	354	-0.007	0.014	6.02E-01	1.00E+00	0.001
PCae C38	182	-0.096	0.068	1.59E-01	1.00E+00	0.011
PCae C38:3	182	-0.031	0.015	3.52E-02	1.00E+00	0.024
PCae C38:4	173	-0.054	0.025	3.07E-02	1.00E+00	0.027
PCae C38:5	288	-0.012	0.010	2.37E-01	1.00E+00	0.005

PCae C38:6	238	0.003	0.027	8.98E-01	1.00E+00	0.000
PCae C40	165	-0.034	0.026	1.94E-01	1.00E+00	0.010
PCae C40:4	171	-0.089	0.049	7.02E-02	1.00E+00	0.019
PCae C40:6	171	-0.069	0.058	2.34E-01	1.00E+00	0.008
SM C21:2	181	-4.484	1.323	8.61E-04	1.64E-01	0.060
SM C31:1	181	1.632	0.898	7.08E-02	1.00E+00	0.018
SM C32:1	354	0.028	0.025	2.63E-01	1.00E+00	0.004
SM C32:2	171	1.173	0.275	3.27E-05	6.22E-03	0.097
SM C33:1	354	-0.031	0.035	3.82E-01	1.00E+00	0.002
SM C34:1	354	-0.006	0.002	8.37E-03	1.00E+00	0.020
SM C34:2	354	0.046	0.016	4.04E-03	7.67E-01	0.023
SM C35:1	304	-0.038	0.068	5.71E-01	1.00E+00	0.001
SM C36:1	354	0.010	0.009	2.88E-01	1.00E+00	0.003
SM C36:2	221	0.062	0.027	2.12E-02	1.00E+00	0.024
SM C36:3	287	0.356	0.189	6.03E-02	1.00E+00	0.012

SM C41:1	238	-0.016	0.019	3.93E-01	1.00E+00	0.003
SM C41:2	237	-0.002	0.025	9.33E-01	1.00E+00	0.000
SM C42:1	188	-0.005	0.013	6.86E-01	1.00E+00	0.001
SM C42:2	173	0.001	0.007	8.83E-01	1.00E+00	0.000
NEFA 10:0	338	0.014	0.022	5.37E-01	1.00E+00	0.001
NEFA 11:0	265	-0.492	0.679	4.69E-01	1.00E+00	0.002
NEFA 12:0	337	-0.083	0.031	7.75E-03	1.00E+00	0.021
NEFA 12:1	220	-0.413	0.764	5.89E-01	1.00E+00	0.001
NEFA 14:0	338	-0.014	0.007	3.56E-02	1.00E+00	0.013
NEFA 14:1	337	-0.031	0.037	3.96E-01	1.00E+00	0.002
NEFA 15:0	338	-0.067	0.032	3.75E-02	1.00E+00	0.013
NEFA 15:1	215	-0.819	0.516	1.14E-01	1.00E+00	0.012
NEFA 16:0	338	-0.001	0.001	2.14E-01	1.00E+00	0.005
NEFA 16:1	338	-0.006	0.004	1.19E-01	1.00E+00	0.007
NEFA 16:2	338	0.201	0.261	4.43E-01	1.00E+00	0.002
NEFA 17:0	338	-0.076	0.032	1.76E-02	1.00E+00	0.017
NEFA 17:1	338	-0.083	0.054	1.25E-01	1.00E+00	0.007
NEFA 18:0	283	-0.006	0.002	3.06E-03	5.82E-01	0.031
NEFA 18:1	338	-0.001	0.001	8.66E-02	1.00E+00	0.009
NEFA 18:2	336	-0.001	0.002	6.68E-01	1.00E+00	0.001
NEFA 18:3	338	-0.021	0.009	1.67E-02	1.00E+00	0.017
NEFA 18:4	336	-0.914	0.583	1.18E-01	1.00E+00	0.007
NEFA 19:0	283	-1.317	0.353	2.28E-04	4.33E-02	0.047
NEFA 19:1	338	-0.247	0.112	2.74E-02	1.00E+00	0.014
NEFA 20:0	338	-0.661	0.153	2.09E-05	3.97E-03	0.053
NEFA 20:1	338	-0.118	0.038	1.95E-03	3.70E-01	0.028
NEFA 20:2	337	-0.023	0.066	7.27E-01	1.00E+00	0.000
NEFA 20:3	338	0.028	0.039	4.76E-01	1.00E+00	0.002
NEFA 20:4	338	0.009	0.010	3.72E-01	1.00E+00	0.002
NEFA 20:5	336	-0.047	0.105	6.54E-01	1.00E+00	0.001
NEFA 22:0	287	-0.355	0.574	5.36E-01	1.00E+00	0.001
NEFA 22:1	337	-1.647	0.473	5.62E-04	1.07E-01	0.035

NEFA 22:2	283	-3.271	2.275	1.52E-01	1.00E+00	0.007
NEFA 22:3	232	-0.761	2.055	7.12E-01	1.00E+00	0.001
NEFA 22:4	337	0.077	0.134	5.64E-01	1.00E+00	0.001
NEFA 22:5	338	-0.094	0.070	1.80E-01	1.00E+00	0.005
NEFA 22:6	338	-0.034	0.032	2.78E-01	1.00E+00	0.004
NEFA 24:0	338	-1.333	0.543	1.46E-02	1.00E+00	0.018
NEFA 24:1	338	-1.207	0.452	7.96E-03	1.00E+00	0.021
NEFA 24:3	150	-16.555	13.926	2.36E-01	1.00E+00	0.009
NEFA 24:4	220	7.336	3.165	2.14E-02	1.00E+00	0.024
NEFA 24:5	166	-5.104	2.903	8.06E-02	1.00E+00	0.019
NEFA 24:6	221	-0.879	1.980	6.57E-01	1.00E+00	0.001
NEFA 26:0	287	-3.657	3.589	3.09E-01	1.00E+00	0.004
NEFA 26:1	338	0.452	0.824	5.84E-01	1.00E+00	0.001
NEFA 26:2	288	0.979	1.121	3.84E-01	1.00E+00	0.003
NEFA 26:3	337	1.209	1.870	5.18E-01	1.00E+00	0.001
NEFA 26:4	167	-35.885	10.383	6.97E-04	1.32E-01	0.068
NEFA 26:5	181	-9.922	10.596	3.50E-01	1.00E+00	0.005
Pyruvate	226	0.002	0.001	4.18E-02	1.00E+00	0.018
Lactate	346	0.000	0.000	3.95E-01	1.00E+00	0.002
Succininate	348	0.003	0.013	7.86E-01	1.00E+00	0.000
Taurine	297	0.005	0.002	2.30E-02	1.00E+00	0.017
3-Methyl-2-oxovalerate	348	0.002	0.012	8.68E-01	1.00E+00	0.000
4-Methyl-2-oxovalerate	348	0.001	0.007	8.36E-01	1.00E+00	0.000
Malate	346	-0.007	0.015	6.28E-01	1.00E+00	0.001
Alpha-ketoglutarate	348	0.012	0.009	1.93E-01	1.00E+00	0.005
Alpha-aminoadipate	161	0.117	0.201	5.62E-01	1.00E+00	0.002
Isocitrate	297	0.007	0.012	5.41E-01	1.00E+00	0.001
Citrate	296	-0.009	0.002	1.49E-05	2.83E-03	0.062
Alpha-ketobutyrate	334	0.001	0.003	6.43E-01	1.00E+00	0.001
Acetoacetate	246	0.000	0.001	6.55E-01	1.00E+00	0.001
Sum AA	351	0.000	0.000	8.53E-01	1.00E+00	0.000
Sum Carn	350	0.006	0.003	3.28E-02	1.00E+00	0.013
Sum LPC	53	-0.001	0.001	3.01E-01	1.00E+00	0.021
Sum PCaa	50	-0.001	0.001	2.27E-01	1.00E+00	0.030
Sum PCae	51	-0.004	0.005	3.92E-01	1.00E+00	0.015
Sum SM	54	-0.005	0.005	3.28E-01	1.00E+00	0.018
Sum NEFA	215	0.000	0.000	8.63E-01	1.00E+00	0.000

UBCS 8 years

Metabolite	N	Estimate	Std..Error	P	P.adjusted	PartialRsquared
Ala	401	0.002	0.001	9.93E-04	2.02E-01	0.027
Arg	400	-0.001	0.002	5.51E-01	1.00E+00	0.001
Asn	363	0.002	0.005	7.12E-01	1.00E+00	0.000
Asp	364	0.023	0.010	2.37E-02	1.00E+00	0.014
Cit	402	-0.011	0.005	3.46E-02	1.00E+00	0.011
Gln	401	0.000	0.000	3.09E-01	1.00E+00	0.003
Glu	402	0.007	0.003	5.36E-03	1.00E+00	0.019
Gly	400	-0.001	0.001	3.59E-01	1.00E+00	0.002
His	402	0.006	0.002	9.08E-03	1.00E+00	0.017
Ile	361	0.009	0.004	3.26E-02	1.00E+00	0.013
Leu	402	0.007	0.002	1.47E-03	2.98E-01	0.025
Lys	401	0.003	0.001	4.54E-02	1.00E+00	0.010
Met	402	0.010	0.009	2.42E-01	1.00E+00	0.003
Orn	401	0.000	0.003	9.82E-01	1.00E+00	0.000
Phe	402	0.009	0.004	3.22E-02	1.00E+00	0.011
Pro	398	0.002	0.001	1.75E-02	1.00E+00	0.014
Ser	401	-0.001	0.001	3.34E-01	1.00E+00	0.002
Thr	402	0.003	0.002	6.39E-02	1.00E+00	0.009
Trp	401	0.010	0.004	4.31E-03	8.74E-01	0.020
Tyr	401	0.015	0.003	2.33E-06	4.73E-04	0.054
Val	402	0.004	0.001	1.44E-04	2.92E-02	0.036
Hpro	400	0.003	0.005	4.85E-01	1.00E+00	0.001
H1	400	0.000	0.000	2.55E-02	1.00E+00	0.012
Carn	401	0.029	0.007	2.50E-05	5.07E-03	0.044
Carn C2	401	-0.032	0.018	6.97E-02	1.00E+00	0.008
Carn C3	402	0.976	0.328	3.07E-03	6.24E-01	0.022
Carn C4	402	0.578	0.558	3.01E-01	1.00E+00	0.003
Carn C5	395	1.692	0.897	6.01E-02	1.00E+00	0.009

Carn C8:1	397	0.743	0.311	1.74E-02	1.00E+00		0.014
Carn C10:1	400	-0.225	0.312	4.71E-01	1.00E+00		0.001
Carn C12:1	402	-0.604	0.302	4.61E-02	1.00E+00		0.010
Carn C14:1	401	-0.697	0.428	1.04E-01	1.00E+00		0.007
Carn C16	400	-2.446	1.105	2.75E-02	1.00E+00		0.012
Carn C18	398	-7.479	2.326	1.41E-03	2.86E-01		0.025
Carn C18:1	402	-1.802	0.919	5.06E-02	1.00E+00		0.010
LPC C14	402	0.508	0.098	3.22E-07	6.54E-05		0.063
LPC C15	402	0.376	0.235	1.11E-01	1.00E+00		0.006
LPC C15:1	402	5.381	1.965	6.46E-03	1.00E+00		0.018
LPC C16	402	0.006	0.004	9.53E-02	1.00E+00		0.007
LPC C16:1	401	0.391	0.092	2.73E-05	5.54E-03		0.043
LPC C17	402	-0.228	0.180	2.05E-01	1.00E+00		0.004
LPC C17:1	402	0.618	0.637	3.32E-01	1.00E+00		0.002
LPC C18	402	0.015	0.010	1.30E-01	1.00E+00		0.006
LPC C18:1	402	-0.002	0.013	8.82E-01	1.00E+00		0.000
LPC C18:2	402	0.002	0.006	7.40E-01	1.00E+00		0.000
LPC C18:3	401	0.626	0.249	1.23E-02	1.00E+00		0.016
LPC C18:6	402	1.065	0.667	1.11E-01	1.00E+00		0.006
LPC C20	402	-0.765	0.678	2.60E-01	1.00E+00		0.003
LPC C20:1	402	-0.343	0.598	5.66E-01	1.00E+00		0.001
LPC C20:2	402	0.118	0.562	8.33E-01	1.00E+00		0.000
LPC C20:3	400	0.228	0.106	3.25E-02	1.00E+00		0.011
LPC C20:4	400	0.075	0.045	9.65E-02	1.00E+00		0.007
LPC C20:5	400	0.738	0.302	1.50E-02	1.00E+00		0.015

LPC C22:4	402	0.207	0.397	6.01E-01	1.00E+00	0.001
LPC C22:5	402	-0.007	0.214	9.75E-01	1.00E+00	0.000
LPC C22:6	401	-0.108	0.130	4.07E-01	1.00E+00	0.002
PCaa C18	402	-0.796	1.070	4.57E-01	1.00E+00	0.001
PCaa C18:1	402	-2.765	1.272	3.03E-02	1.00E+00	0.012
PCaa C30	402	0.046	0.030	1.29E-01	1.00E+00	0.006
PCaa C30:2	402	0.032	0.103	7.58E-01	1.00E+00	0.000
PCaa C32	402	-0.008	0.007	2.36E-01	1.00E+00	0.004
PCaa C32:1	402	0.017	0.007	2.42E-02	1.00E+00	0.013
PCaa C32:2	402	0.088	0.029	2.46E-03	4.99E-01	0.023
PCaa C32:3	402	0.584	0.276	3.49E-02	1.00E+00	0.011
PCaa C34	400	-0.008	0.020	6.89E-01	1.00E+00	0.000
PCaa C34:1	401	0.000	0.001	8.75E-01	1.00E+00	0.000
PCaa C34:2	401	0.000	0.001	6.73E-01	1.00E+00	0.000
PCaa C34:3	402	0.030	0.011	5.47E-03	1.00E+00	0.019
PCaa C34:4	402	0.262	0.061	2.24E-05	4.54E-03	0.044
PCaa C34:5	402	1.176	0.472	1.31E-02	1.00E+00	0.015
PCaa C34:6	388	-0.257	1.027	8.03E-01	1.00E+00	0.000
PCaa C36	401	-0.004	0.049	9.33E-01	1.00E+00	0.000
PCaa C36:1	402	0.000	0.003	8.83E-01	1.00E+00	0.000
PCaa C36:2	401	0.002	0.001	1.13E-01	1.00E+00	0.006
PCaa C36:3	402	0.005	0.002	3.47E-02	1.00E+00	0.011
PCaa C36:4	402	0.003	0.001	3.32E-02	1.00E+00	0.011
PCaa C36:5	402	0.020	0.008	1.68E-02	1.00E+00	0.014
PCaa C36:6	402	0.622	0.186	9.32E-04	1.89E-01	0.027
PCaa C38	402	0.017	0.070	8.10E-01	1.00E+00	0.000
PCaa C38:1	398	0.030	0.064	6.38E-01	1.00E+00	0.001
PCaa C38:2	402	0.026	0.028	3.48E-01	1.00E+00	0.002
PCaa C38:3	401	0.021	0.005	1.33E-04	2.71E-02	0.036
PCaa C38:4	402	0.004	0.002	1.65E-02	1.00E+00	0.014
PCaa C38:5	402	0.004	0.004	2.61E-01	1.00E+00	0.003
PCaa C38:6	402	-0.001	0.003	8.39E-01	1.00E+00	0.000
PCaa C40	401	-0.183	0.341	5.92E-01	1.00E+00	0.001

PCaa C40:1	395	-0.088	0.645	8.91E-01	1.00E+00	0.000
PCaa C40:2	398	0.644	0.571	2.60E-01	1.00E+00	0.003
PCaa C40:3	394	-0.302	0.336	3.70E-01	1.00E+00	0.002
PCaa C40:4	402	0.073	0.037	4.97E-02	1.00E+00	0.010
PCaa C40:5	401	0.031	0.017	6.36E-02	1.00E+00	0.009
PCaa C40:6	402	0.021	0.009	2.42E-02	1.00E+00	0.013
PCaa C42	402	-0.221	0.303	4.65E-01	1.00E+00	0.001
PCaa C42:1	400	-0.267	0.612	6.63E-01	1.00E+00	0.000
PCaa C42:2	396	0.050	1.002	9.60E-01	1.00E+00	0.000
PCaa C42:4	400	0.178	0.681	7.94E-01	1.00E+00	0.000
PCaa C42:5	399	0.142	0.420	7.36E-01	1.00E+00	0.000
PCaa C42:6	402	0.402	0.356	2.60E-01	1.00E+00	0.003
PCaa C43:6	402	-0.027	0.138	8.47E-01	1.00E+00	0.000
PCae C30	402	-0.093	0.313	7.65E-01	1.00E+00	0.000
PCae C32	399	-0.063	0.050	2.05E-01	1.00E+00	0.004
PCae C32:1	402	-0.036	0.066	5.87E-01	1.00E+00	0.001
PCae C32:2	402	0.030	0.363	9.34E-01	1.00E+00	0.000
PCae C34	400	-0.004	0.067	9.54E-01	1.00E+00	0.000
PCae C34:1	402	-0.033	0.020	1.09E-01	1.00E+00	0.006
PCae C34:2	402	0.008	0.015	6.00E-01	1.00E+00	0.001
PCae C34:3	402	0.017	0.017	3.24E-01	1.00E+00	0.002
PCae C34:4	402	0.930	0.463	4.51E-02	1.00E+00	0.010
PCae C36	401	-0.037	0.166	8.22E-01	1.00E+00	0.000
PCae C36:1	402	-0.069	0.037	6.27E-02	1.00E+00	0.009
PCae C36:2	402	-0.036	0.018	4.86E-02	1.00E+00	0.010
PCae C36:3	402	0.025	0.026	3.41E-01	1.00E+00	0.002
PCae C36:4	401	0.021	0.010	3.14E-02	1.00E+00	0.012
PCae C36:5	402	0.028	0.014	5.07E-02	1.00E+00	0.010
PCae C36:6	397	0.465	0.208	2.61E-02	1.00E+00	0.012
PCae C38	401	0.080	0.092	3.87E-01	1.00E+00	0.002
PCae C38:2	401	-0.056	0.083	4.98E-01	1.00E+00	0.001
PCae C38:3	402	-0.034	0.046	4.65E-01	1.00E+00	0.001
PCae C38:4	400	-0.006	0.012	6.32E-01	1.00E+00	0.001
PCae C38:5	401	0.005	0.009	5.60E-01	1.00E+00	0.001

PCae C38:6	402	0.023	0.020	2.45E-01	1.00E+00	0.003
PCae C40	402	-0.017	0.037	6.45E-01	1.00E+00	0.001
PCae C40:1	397	0.039	0.085	6.41E-01	1.00E+00	0.001
PCae C40:2	402	-0.064	0.229	7.80E-01	1.00E+00	0.000
PCae C40:3	401	-0.207	0.229	3.67E-01	1.00E+00	0.002
PCae C40:4	402	-0.089	0.074	2.26E-01	1.00E+00	0.004
PCae C40:5	402	-0.093	0.055	9.01E-02	1.00E+00	0.007
PCae C40:6	402	-0.055	0.049	2.58E-01	1.00E+00	0.003
PCae C42	402	-0.276	0.438	5.28E-01	1.00E+00	0.001
PCae C42:1	400	-0.019	0.238	9.38E-01	1.00E+00	0.000
PCae C42:2	402	-0.238	0.246	3.35E-01	1.00E+00	0.002
PCae C42:3	401	-0.192	0.238	4.21E-01	1.00E+00	0.002
PCae C42:4	402	-0.146	0.166	3.80E-01	1.00E+00	0.002
PCae C42:5	402	-0.091	0.095	3.39E-01	1.00E+00	0.002
PCae C42:6	402	-0.170	0.144	2.37E-01	1.00E+00	0.003
SM C32:2	326	2.847	0.286	1.50E-20	3.05E-18	0.234
SM C35	401	-0.127	0.140	3.66E-01	1.00E+00	0.002
SM C35:1	402	-0.029	0.037	4.26E-01	1.00E+00	0.002
SM C36	401	-0.007	0.049	8.91E-01	1.00E+00	0.000
SM C36:1	402	-0.002	0.006	7.66E-01	1.00E+00	0.000
SM C36:2	402	0.005	0.011	6.76E-01	1.00E+00	0.000
SM C37:1	401	-0.040	0.067	5.50E-01	1.00E+00	0.001
SM C38:1	402	-0.001	0.005	8.44E-01	1.00E+00	0.000
SM C38:2	402	-0.001	0.007	8.73E-01	1.00E+00	0.000
SM C38:3	402	0.205	0.166	2.18E-01	1.00E+00	0.004
SM C39:1	402	0.027	0.033	4.06E-01	1.00E+00	0.002
SM C39:2	400	0.134	0.101	1.87E-01	1.00E+00	0.004
SM C39:5	402	0.179	0.168	2.89E-01	1.00E+00	0.003
SM C40:1	399	0.001	0.005	9.01E-01	1.00E+00	0.000
SM C40:2	402	0.010	0.007	1.46E-01	1.00E+00	0.005
SM C40:3	399	0.019	0.014	1.87E-01	1.00E+00	0.004
SM C40:4	402	0.004	0.018	8.29E-01	1.00E+00	0.000

SM C41:1	402	0.007	0.017	6.66E-01	1.00E+00	0.000
SM C41:2	402	0.033	0.025	2.01E-01	1.00E+00	0.004
SM C41:3	394	0.178	0.113	1.15E-01	1.00E+00	0.006
SM C42:1	401	0.006	0.008	4.71E-01	1.00E+00	0.001
SM C42:2	402	0.003	0.004	4.86E-01	1.00E+00	0.001
SM C42:3	402	0.033	0.010	9.34E-04	1.90E-01	0.027
SM C42:4	401	0.036	0.025	1.58E-01	1.00E+00	0.005
SM C42:6	402	-0.039	0.053	4.64E-01	1.00E+00	0.001
SM C43	400	-0.226	0.244	3.53E-01	1.00E+00	0.002
SM C43:1	402	0.039	0.102	7.01E-01	1.00E+00	0.000
SM C43:2	402	0.026	0.066	6.92E-01	1.00E+00	0.000
SM C43:3	400	0.100	0.278	7.20E-01	1.00E+00	0.000
SM C44:2	398	0.194	0.352	5.82E-01	1.00E+00	0.001
SM C44:6	400	0.038	0.112	7.37E-01	1.00E+00	0.000
NEFA 10:0	323	-0.163	0.051	1.68E-03	3.41E-01	0.030
NEFA 12:0	398	-0.019	0.011	8.73E-02	1.00E+00	0.007
NEFA 12:1	395	-0.010	0.051	8.46E-01	1.00E+00	0.000
NEFA 14:0	401	-0.010	0.006	1.01E-01	1.00E+00	0.007
NEFA 14:1	399	-0.069	0.040	8.33E-02	1.00E+00	0.008
NEFA 15:0	394	-0.042	0.032	1.88E-01	1.00E+00	0.004
NEFA 15:1	335	-0.390	0.440	3.76E-01	1.00E+00	0.002
NEFA 16:0	401	-0.001	0.001	1.11E-01	1.00E+00	0.006
NEFA 16:1	400	-0.007	0.003	5.23E-02	1.00E+00	0.009
NEFA 16:2	398	0.137	0.325	6.73E-01	1.00E+00	0.000
NEFA 17:0	399	-0.029	0.032	3.74E-01	1.00E+00	0.002
NEFA 17:1	399	-0.036	0.047	4.41E-01	1.00E+00	0.001
NEFA 18:0	398	-0.003	0.002	2.30E-01	1.00E+00	0.004
NEFA 18:1	402	-0.001	0.001	1.29E-01	1.00E+00	0.006
NEFA 18:2	401	-0.002	0.002	3.01E-01	1.00E+00	0.003
NEFA 18:3	401	-0.002	0.017	9.25E-01	1.00E+00	0.000
NEFA 19:1	402	-0.109	0.104	2.95E-01	1.00E+00	0.003
NEFA 20:0	360	-0.123	0.185	5.04E-01	1.00E+00	0.001
NEFA 20:2	351	0.042	0.086	6.26E-01	1.00E+00	0.001
NEFA 20:3	285	0.164	0.109	1.32E-01	1.00E+00	0.008
NEFA 20:4	402	0.030	0.038	4.31E-01	1.00E+00	0.002
NEFA 20:5	387	0.112	0.251	6.55E-01	1.00E+00	0.001

NEFA 22:4	402	0.242	0.196	2.18E-01	1.00E+00	0.004
NEFA 22:5	402	0.072	0.101	4.72E-01	1.00E+00	0.001
NEFA 22:6	402	0.025	0.057	6.60E-01	1.00E+00	0.000
NEFA 24:0	399	0.025	0.640	9.69E-01	1.00E+00	0.000
NEFA 24:2	355	4.028	2.677	1.33E-01	1.00E+00	0.006
NEFA 24:4	396	3.218	3.175	3.12E-01	1.00E+00	0.003
NEFA 24:5	401	6.252	2.953	3.49E-02	1.00E+00	0.011
NEFA 26:1	402	3.256	1.112	3.60E-03	7.30E-01	0.021

Sum AA	334	0.000	0.000	5.63E-03	1.00E+00	0.023
Sum Carn	381	0.023	0.006	4.27E-04	8.67E-02	0.032
Sum LPC	397	0.002	0.002	3.40E-01	1.00E+00	0.002
Sum PCaa	399	0.000	0.000	3.40E-01	1.00E+00	0.002
Sum PCae	399	0.001	0.002	4.27E-01	1.00E+00	0.002
Sum SM	325	0.005	0.003	8.23E-02	1.00E+00	0.009
Sum NEFA	274	0.000	0.000	6.61E-01	1.00E+00	0.001

GINI-LISA 10 years

Metabolite	N	Estimate	Std..Error	P	P.adjusted	PartialRsquared
Ala	231	0.000	0.001	6.58E-01	1.00E+00	0.001
Arg	231	0.001	0.003	6.10E-01	1.00E+00	0.001
Asn	231	-0.008	0.005	9.48E-02	1.00E+00	0.012
Asp	230	0.008	0.005	1.14E-01	1.00E+00	0.011
Cit	231	0.000	0.007	9.59E-01	1.00E+00	0.000
Gln	203	0.000	0.001	4.85E-01	1.00E+00	0.002
Glu	231	0.001	0.001	4.68E-01	1.00E+00	0.002
Gly	231	-0.001	0.001	2.70E-01	1.00E+00	0.005
His	231	0.000	0.003	9.57E-01	1.00E+00	0.000
Ile	231	0.003	0.004	5.08E-01	1.00E+00	0.002
Leu	231	0.005	0.002	2.23E-02	1.00E+00	0.023
Lys	231	0.001	0.002	3.73E-01	1.00E+00	0.003
Met	231	-0.015	0.012	2.18E-01	1.00E+00	0.007
Orn	231	0.001	0.002	5.99E-01	1.00E+00	0.001
Phe	231	0.008	0.003	2.67E-02	1.00E+00	0.021
Pro	231	0.000	0.001	8.15E-01	1.00E+00	0.000
Ser	231	0.000	0.002	8.63E-01	1.00E+00	0.000
Thr	231	-0.003	0.002	7.49E-02	1.00E+00	0.014
Trp	231	0.003	0.005	5.70E-01	1.00E+00	0.001
Tyr	231	0.007	0.004	5.56E-02	1.00E+00	0.016
Val	231	0.003	0.001	4.96E-03	1.00E+00	0.034
H1	232	0.000	0.000	4.35E-01	1.00E+00	0.003
Carn	173	0.018	0.011	1.09E-01	1.00E+00	0.015
Carn C2	176	0.013	0.034	7.14E-01	1.00E+00	0.001
Carn C3	188	1.808	0.800	2.50E-02	1.00E+00	0.027
Carn C4	149	1.811	0.888	4.31E-02	1.00E+00	0.028
Carn C5	141	2.453	2.042	2.32E-01	1.00E+00	0.010
Carn C6	231	0.028	1.452	9.85E-01	1.00E+00	0.000
Carn C8	232	-0.057	0.219	7.96E-01	1.00E+00	0.000

Carn C8:1	231	1.782	0.914	5.23E-02	1.00E+00	0.016
Carn C9	215	-3.018	2.369	2.04E-01	1.00E+00	0.008
Carn C10	231	-0.180	0.159	2.60E-01	1.00E+00	0.006
Carn C10:1	232	-0.035	0.253	8.90E-01	1.00E+00	0.000
Carn C10:2	145	-0.592	1.421	6.78E-01	1.00E+00	0.001
Carn C12	231	-0.288	0.364	4.31E-01	1.00E+00	0.003
Carn C12:1	204	-0.047	0.168	7.81E-01	1.00E+00	0.000
Carn C14	153	-4.226	2.881	1.44E-01	1.00E+00	0.014
Carn C14:1	231	-0.132	0.544	8.08E-01	1.00E+00	0.000
Carn C14:2	151	0.166	2.142	9.38E-01	1.00E+00	0.000
Carn C16	213	1.960	2.263	3.87E-01	1.00E+00	0.004
Carn C16:1	183	2.683	3.625	4.60E-01	1.00E+00	0.003
Carn C18	176	-0.898	2.716	7.41E-01	1.00E+00	0.001
Carn C18:1	232	1.732	1.356	2.03E-01	1.00E+00	0.007
Carn C18:2	166	6.004	3.180	6.08E-02	1.00E+00	0.021
LPC C14	232	0.135	0.067	4.49E-02	1.00E+00	0.017
LPC C15	218	0.007	0.166	9.67E-01	1.00E+00	0.000
LPC C16	232	0.003	0.002	1.52E-01	1.00E+00	0.009
LPC C16:1	232	0.278	0.074	2.11E-04	5.12E-02	0.058
LPC C17	232	-0.089	0.105	3.96E-01	1.00E+00	0.003
LPC C17:1	218	-0.123	0.493	8.03E-01	1.00E+00	0.000
LPC C18	232	0.003	0.007	6.50E-01	1.00E+00	0.001
LPC C18:1	232	-0.014	0.013	3.18E-01	1.00E+00	0.004
LPC C18:2	232	-0.026	0.007	3.12E-04	7.57E-02	0.055
LPC C18:3	218	-0.392	0.241	1.05E-01	1.00E+00	0.012
LPC C18:6	143	-0.216	0.619	7.27E-01	1.00E+00	0.001
LPC C20:2	126	0.115	0.587	8.44E-01	1.00E+00	0.000
LPC C20:3	232	0.135	0.091	1.39E-01	1.00E+00	0.009
LPC C20:4	232	0.011	0.032	7.35E-01	1.00E+00	0.000
LPC C20:5	229	0.547	0.294	6.43E-02	1.00E+00	0.015

LPC C22:5	172	-0.430	0.372	2.49E-01	1.00E+00	0.008
LPC C22:6	218	-0.021	0.105	8.39E-01	1.00E+00	0.000
PCaa C30	232	-0.004	0.049	9.42E-01	1.00E+00	0.000
PCaa C30:2	161	-0.392	0.306	2.01E-01	1.00E+00	0.010
PCaa C30:3	159	0.169	0.855	8.44E-01	1.00E+00	0.000
PCaa C30:4	117	1.019	1.417	4.73E-01	1.00E+00	0.004
PCaa C32	232	-0.009	0.022	6.65E-01	1.00E+00	0.001
PCaa C32:1	232	0.040	0.011	6.36E-04	1.55E-01	0.050
PCaa C32:2	232	0.016	0.052	7.55E-01	1.00E+00	0.000
PCaa C32:3	182	0.076	0.358	8.32E-01	1.00E+00	0.000
PCaa C34:1	232	0.002	0.001	2.65E-01	1.00E+00	0.005
PCaa C34:2	232	-0.001	0.001	2.08E-01	1.00E+00	0.007
PCaa C34:3	232	0.008	0.014	5.74E-01	1.00E+00	0.001
PCaa C34:4	232	0.181	0.093	5.39E-02	1.00E+00	0.016
PCaa C34:5	152	1.109	0.881	2.10E-01	1.00E+00	0.010
PCaa C36	179	-0.050	0.033	1.37E-01	1.00E+00	0.012
PCaa C36:1	232	0.003	0.005	5.44E-01	1.00E+00	0.002
PCaa C36:2	232	-0.001	0.001	3.65E-01	1.00E+00	0.004
PCaa C36:3	232	0.002	0.002	3.03E-01	1.00E+00	0.005
PCaa C36:4	232	0.003	0.002	7.08E-02	1.00E+00	0.014
PCaa C36:5	231	0.030	0.012	1.14E-02	1.00E+00	0.028
PCaa C36:6	211	0.172	0.143	2.31E-01	1.00E+00	0.007
PCaa C38	232	-0.035	0.054	5.21E-01	1.00E+00	0.002
PCaa C38:2	109	0.046	0.046	3.22E-01	1.00E+00	0.009
PCaa C38:3	232	0.017	0.005	4.10E-04	9.95E-02	0.053
PCaa C38:4	232	0.006	0.002	1.47E-02	1.00E+00	0.026
PCaa C38:5	232	0.010	0.005	3.47E-02	1.00E+00	0.019
PCaa C38:6	232	0.003	0.003	2.84E-01	1.00E+00	0.005
PCaa C40	174	-0.225	0.142	1.16E-01	1.00E+00	0.014

PCaa C40:1	183	0.040	0.113	7.27E-01	1.00E+00	0.001
PCaa C40:3	128	-0.054	0.102	5.96E-01	1.00E+00	0.002
PCaa C40:4	232	0.053	0.046	2.44E-01	1.00E+00	0.006
PCaa C40:5	232	0.041	0.018	2.84E-02	1.00E+00	0.021
PCaa C40:6	232	0.015	0.008	6.05E-02	1.00E+00	0.015
PCaa C42	218	-0.279	0.208	1.82E-01	1.00E+00	0.008
PCaa C42:1	115	-0.836	0.391	3.47E-02	1.00E+00	0.039
PCaa C42:4	116	-0.308	0.260	2.38E-01	1.00E+00	0.012
PCaa C42:5	146	-0.654	0.328	4.83E-02	1.00E+00	0.027
PCaa C42:6	128	-0.353	0.190	6.55E-02	1.00E+00	0.027
PCaa C43:6	229	-0.446	0.180	1.42E-02	1.00E+00	0.026
PCaa C44:12	203	-0.344	0.237	1.48E-01	1.00E+00	0.010
PCae C32	232	-0.107	0.077	1.69E-01	1.00E+00	0.008
PCae C32:1	232	-0.095	0.073	1.95E-01	1.00E+00	0.007
PCae C32:2	199	0.032	0.198	8.72E-01	1.00E+00	0.000
PCae C34	216	0.103	0.137	4.52E-01	1.00E+00	0.003
PCae C34:1	232	-0.022	0.030	4.63E-01	1.00E+00	0.002
PCae C34:2	232	-0.032	0.021	1.37E-01	1.00E+00	0.010
PCae C34:3	232	-0.055	0.024	2.61E-02	1.00E+00	0.021
PCae C34:4	139	-0.340	0.660	6.07E-01	1.00E+00	0.002
PCae C36	170	-0.410	0.259	1.16E-01	1.00E+00	0.015
PCae C36:1	232	-0.022	0.018	2.22E-01	1.00E+00	0.006
PCae C36:2	232	-0.039	0.017	2.64E-02	1.00E+00	0.021
PCae C36:3	232	-0.018	0.028	5.26E-01	1.00E+00	0.002
PCae C36:4	232	0.003	0.012	7.71E-01	1.00E+00	0.000
PCae C36:5	232	0.002	0.017	8.87E-01	1.00E+00	0.000
PCae C36:6	117	-0.129	0.430	7.65E-01	1.00E+00	0.001
PCae C38	231	-0.018	0.063	7.76E-01	1.00E+00	0.000
PCae C38:2	232	-0.038	0.029	1.92E-01	1.00E+00	0.007
PCae C38:3	232	-0.009	0.020	6.48E-01	1.00E+00	0.001
PCae C38:4	232	-0.019	0.016	2.33E-01	1.00E+00	0.006
PCae C38:5	232	-0.002	0.013	9.02E-01	1.00E+00	0.000

PCae C38:6	232	0.007	0.027	8.04E-01	1.00E+00	0.000
PCae C40	204	0.005	0.021	8.08E-01	1.00E+00	0.000
PCae C40:1	218	-0.085	0.069	2.19E-01	1.00E+00	0.007
PCae C40:2	154	-0.002	0.072	9.80E-01	1.00E+00	0.000
PCae C40:3	177	0.018	0.035	6.03E-01	1.00E+00	0.002
PCae C40:4	204	-0.100	0.052	5.52E-02	1.00E+00	0.018
PCae C40:5	232	-0.060	0.037	1.11E-01	1.00E+00	0.011
PCae C40:6	232	-0.058	0.047	2.21E-01	1.00E+00	0.006
PCae C42	181	-0.261	0.178	1.45E-01	1.00E+00	0.012
PCae C42:1	160	-0.175	0.100	8.17E-02	1.00E+00	0.019
PCae C42:2	186	-0.166	0.155	2.86E-01	1.00E+00	0.006
PCae C42:3	199	-0.126	0.096	1.89E-01	1.00E+00	0.009
PCae C42:4	172	-0.203	0.196	3.02E-01	1.00E+00	0.006
PCae C42:5	216	-0.099	0.072	1.73E-01	1.00E+00	0.009
PCae C42:6	232	-0.184	0.133	1.67E-01	1.00E+00	0.008
SM C30:1	197	0.747	0.369	4.46E-02	1.00E+00	0.021
SM C31:1	117	1.358	0.968	1.63E-01	1.00E+00	0.017
SM C32:1	232	0.051	0.028	6.59E-02	1.00E+00	0.015
SM C32:2	232	1.347	0.241	6.57E-08	1.60E-05	0.119
SM C33:1	232	-0.009	0.044	8.42E-01	1.00E+00	0.000
SM C33:2	169	2.958	1.014	4.02E-03	9.76E-01	0.048
SM C33:3	170	-1.366	1.802	4.49E-01	1.00E+00	0.003
SM C34:1	232	-0.003	0.003	2.90E-01	1.00E+00	0.005
SM C34:2	232	0.040	0.019	4.07E-02	1.00E+00	0.018
SM C35	155	-0.300	0.401	4.55E-01	1.00E+00	0.004
SM C35:1	232	0.053	0.086	5.34E-01	1.00E+00	0.002
SM C35:2	188	0.209	0.512	6.84E-01	1.00E+00	0.001
SM C36:1	232	0.025	0.013	6.66E-02	1.00E+00	0.015
SM C36:2	232	0.068	0.024	4.95E-03	1.00E+00	0.034
SM C36:3	232	0.599	0.301	4.79E-02	1.00E+00	0.017
SM C37:1	115	-0.049	0.136	7.22E-01	1.00E+00	0.001
SM C37:3	156	0.024	0.525	9.64E-01	1.00E+00	0.000
SM C38:1	181	0.009	0.008	2.78E-01	1.00E+00	0.007
SM C38:2	232	-0.002	0.016	8.81E-01	1.00E+00	0.000
SM C38:3	144	0.627	0.500	2.13E-01	1.00E+00	0.011
SM C39:1	232	0.047	0.038	2.16E-01	1.00E+00	0.007
SM C39:2	142	-0.095	0.141	4.98E-01	1.00E+00	0.003
SM C39:5	118	0.606	0.454	1.84E-01	1.00E+00	0.015
SM C40:2	232	0.010	0.009	2.69E-01	1.00E+00	0.005
SM C40:4	220	0.181	0.115	1.16E-01	1.00E+00	0.011

SM C40:5	201	0.537	0.244	2.88E-02	1.00E+00	0.024
SM C41:1	232	0.017	0.020	4.10E-01	1.00E+00	0.003
SM C41:2	232	0.041	0.029	1.63E-01	1.00E+00	0.008
SM C42:1	232	0.003	0.012	8.28E-01	1.00E+00	0.000
SM C42:2	232	0.010	0.007	1.63E-01	1.00E+00	0.008
SM C42:3	218	0.025	0.011	2.12E-02	1.00E+00	0.024
SM C42:4	196	0.047	0.031	1.27E-01	1.00E+00	0.012
SM C42:6	220	0.074	0.076	3.28E-01	1.00E+00	0.004
SM C43	137	-0.191	0.374	6.11E-01	1.00E+00	0.002
SM C43:1	229	0.121	0.165	4.65E-01	1.00E+00	0.002
SM C43:2	232	0.256	0.095	7.40E-03	1.00E+00	0.031
SM C44:6	190	0.124	0.146	3.95E-01	1.00E+00	0.004
NEFA 10:0	220	-0.214	0.064	1.06E-03	2.57E-01	0.048
NEFA 12:0	192	-0.027	0.010	1.05E-02	1.00E+00	0.034
NEFA 12:1	134	-0.077	0.092	4.03E-01	1.00E+00	0.005
NEFA 13:0	93	-0.004	0.210	9.86E-01	1.00E+00	0.000
NEFA 13:1	179	-0.639	0.951	5.03E-01	1.00E+00	0.003
NEFA 14:0	231	-0.011	0.009	2.17E-01	1.00E+00	0.007
NEFA 14:1	231	0.027	0.055	6.24E-01	1.00E+00	0.001
NEFA 14:2	104	0.013	0.013	3.03E-01	1.00E+00	0.010
NEFA 16:0	232	-0.001	0.001	6.28E-01	1.00E+00	0.001
NEFA 16:1	204	-0.001	0.004	8.24E-01	1.00E+00	0.000
NEFA 16:2	211	0.016	0.232	9.47E-01	1.00E+00	0.000
NEFA 17:0	217	-0.061	0.048	2.05E-01	1.00E+00	0.007
NEFA 17:1	204	-0.030	0.070	6.67E-01	1.00E+00	0.001
NEFA 17:2	136	-2.761	1.524	7.23E-02	1.00E+00	0.024
NEFA 18:0	148	-0.006	0.004	8.21E-02	1.00E+00	0.021
NEFA 18:1	232	0.000	0.001	7.06E-01	1.00E+00	0.001
NEFA 18:2	232	-0.003	0.003	3.11E-01	1.00E+00	0.004
NEFA 18:3	232	0.000	0.009	9.64E-01	1.00E+00	0.000
NEFA 18:4	212	0.494	0.550	3.70E-01	1.00E+00	0.004
NEFA 19:0	216	-0.823	0.392	3.69E-02	1.00E+00	0.020
NEFA 19:1	219	-0.054	0.125	6.67E-01	1.00E+00	0.001
NEFA 20:0	123	-0.270	0.276	3.30E-01	1.00E+00	0.008
NEFA 20:1	232	-0.034	0.048	4.79E-01	1.00E+00	0.002
NEFA 20:2	183	-0.108	0.076	1.56E-01	1.00E+00	0.011
NEFA 20:3	213	0.118	0.062	5.93E-02	1.00E+00	0.017
NEFA 20:4	204	0.024	0.032	4.50E-01	1.00E+00	0.003
NEFA 20:5	168	0.071	0.114	5.35E-01	1.00E+00	0.002
NEFA 22:0	81	0.699	0.639	2.77E-01	1.00E+00	0.015
NEFA 22:1	229	-0.443	0.334	1.86E-01	1.00E+00	0.008

NEFA 22:2	124	-1.646	2.224	4.61E-01	1.00E+00	0.004
NEFA 22:3	204	1.251	1.527	4.14E-01	1.00E+00	0.003
NEFA 22:4	204	0.156	0.170	3.60E-01	1.00E+00	0.004
NEFA 22:5	232	0.059	0.090	5.13E-01	1.00E+00	0.002
NEFA 22:6	232	0.012	0.039	7.48E-01	1.00E+00	0.000
NEFA 24:0	188	-1.987	0.678	3.80E-03	9.23E-01	0.044
NEFA 24:1	199	-0.461	0.505	3.62E-01	1.00E+00	0.004
NEFA 24:2	149	2.147	4.099	6.01E-01	1.00E+00	0.002
NEFA 24:3	143	8.557	12.662	5.00E-01	1.00E+00	0.003
NEFA 24:4	204	2.455	3.293	4.57E-01	1.00E+00	0.003
NEFA 24:5	217	4.126	2.128	5.39E-02	1.00E+00	0.017
NEFA 24:6	174	5.436	3.445	1.16E-01	1.00E+00	0.014
NEFA 26:0	148	-6.889	2.644	1.01E-02	1.00E+00	0.044
NEFA 26:1	187	-1.167	2.534	6.46E-01	1.00E+00	0.001
NEFA 26:2	181	-0.942	3.437	7.84E-01	1.00E+00	0.000
NEFA 26:3	151	-0.809	3.924	8.37E-01	1.00E+00	0.000
NEFA 26:4	189	12.369	13.157	3.48E-01	1.00E+00	0.005
NEFA 26:5	176	37.551	18.271	4.14E-02	1.00E+00	0.024
NEFA 26:6	120	-10.638	7.696	1.69E-01	1.00E+00	0.016
Pyruvate	232	0.000	0.001	7.00E-01	1.00E+00	0.001
Lactate	232	0.000	0.000	7.61E-01	1.00E+00	0.000
Fumarate	232	-0.070	0.187	7.07E-01	1.00E+00	0.001
3-Methyl-2-oxobutanoate	232	0.021	0.019	2.79E-01	1.00E+00	0.005
Succininate	232	-0.021	0.026	4.36E-01	1.00E+00	0.003
Methylmalonate	232	-0.029	0.291	9.21E-01	1.00E+00	0.000
Taurine	232	0.002	0.003	3.30E-01	1.00E+00	0.004
3-Methyl-2-oxovalerate	232	0.003	0.018	8.48E-01	1.00E+00	0.000
4-Methyl-2-oxovalerate	232	0.006	0.011	5.67E-01	1.00E+00	0.001
Malate	232	-0.057	0.102	5.73E-01	1.00E+00	0.001
Alpha-ketoglutarate	231	0.069	0.046	1.33E-01	1.00E+00	0.010
Alpha-aminoadipate	232	1.57495327	0.588044898	0.007933885	1	0.0302
Isocitrate	219	0.147388242	0.342404861	0.66729539	1	0.0009
Citrate	232	-0.015389558	0.007590684	0.043772197	1	0.0176
Alpha-ketobutyrate	213	-0.016	0.018	3.66E-01	1.00E+00	0.004
Acetoacetate	228	-0.004	0.006	4.26E-01	1.00E+00	0.003
Sum AA	202	3.85E-06	0.000122199	0.97488105	1	0.0000
Sum Carn	122	0.008376627	0.010481906	0.42578171	1	0.0053
Sum LPC	215	-0.000144602	0.00135467	0.915092794	1	0.0001
Sum PCaa	231	0.000150318	0.000216817	0.488827004	1	0.0021
Sum PCae	203	-0.001987177	0.001796522	0.269994788	1	0.0061
Sum SM	232	0.003807366	0.002541004	0.135408264	1	0.0097
Sum NEFA	75	-0.000887122	0.00070936	0.21507582	1	0.020975125

Meta Analyses

Metabolite	N	Estimate	Std..Error	P	P.adjusted	PartialRsquared	Q	Qp
Ala	986	0.001	0.000	1.49E-02	1.00E+00	0.0058	7.45	0.024
Arg	985	-0.001	0.001	4.34E-01	1.00E+00	0.0006	2.16	0.34
Asn	948	-0.006	0.003	5.47E-02	1.00E+00	0.0038	3.25	0.2
Asp	948	0.012	0.005	1.04E-02	1.00E+00	0.0067	1.64	0.44
Cit	987	-0.010	0.004	7.64E-03	8.79E-01	0.0069	4.39	0.11
Gln	957	-0.001	0.000	2.35E-03	2.71E-01	0.0094	2.34	0.31
Glu	987	0.001	0.001	6.13E-02	1.00E+00	0.0035	6.1	0.047
Gly	985	-0.002	0.001	4.23E-03	4.87E-01	0.0080	5.68	0.06
His	987	0.002	0.001	1.30E-01	1.00E+00	0.0023	4.44	0.11
Ile	946	0.008	0.003	4.77E-03	5.48E-01	0.0082	2.14	0.34
Leu	987	0.007	0.001	2.45E-06	2.82E-04	0.0215	1.07	0.59
Lys	986	0.002	0.001	3.51E-02	1.00E+00	0.0043	0.763	0.68
Met	987	0.003	0.006	6.40E-01	1.00E+00	0.0002	3.04	0.22
Orn	986	0.001	0.002	3.79E-01	1.00E+00	0.0008	0.472	0.79
Phe	986	0.009	0.003	2.96E-04	3.41E-02	0.0129	0.369	0.83
Pro	983	0.001	0.001	6.82E-02	1.00E+00	0.0033	2.27	0.32
Ser	985	-0.002	0.001	1.02E-01	1.00E+00	0.0026	1.66	0.44
Thr	987	0.001	0.001	6.47E-01	1.00E+00	0.0002	7.06	0.029
Trp	986	0.009	0.003	6.15E-04	7.07E-02	0.0114	2.77	0.25
Tyr	985	0.015	0.002	1.26E-12	1.45E-10	0.0479	11.9	0.003
Val	987	0.004	0.001	3.34E-09	3.84E-07	0.0334	1.62	0.44
H1	986	0.000	0.000	1.74E-01	1.00E+00	0.0018	3.35	0.19
Carn	928	0.010	0.002	2.69E-05	3.09E-03	0.0183	8.53	0.014
Carn C2	931	-0.025	0.013	5.85E-02	1.00E+00	0.0037	1.56	0.46
Carn C3	941	0.588	0.153	1.33E-04	1.53E-02	0.0150	4.29	0.12
Carn C5	890	2.013	0.547	2.47E-04	2.84E-02	0.0147	0.203	0.9

Carn C8:1	982	0.751	0.290	9.73E-03	1.00E+00	0.0065	1.94	0.38
Carn C10:1	985	0.009	0.170	9.56E-01	1.00E+00	0.0000	1.2	0.55
Carn C12:1	960	-0.201	0.121	9.67E-02	1.00E+00	0.0028	2.81	0.25
Carn C14:1	985	-0.399	0.242	1.00E-01	1.00E+00	0.0027	0.731	0.69
Carn C16	967	-1.505	0.965	1.19E-01	1.00E+00	0.0025	3.15	0.21
Carn C18	928	-5.444	1.749	1.91E-03	2.19E-01	0.0100	5.52	0.06
Carn C18:1	988	-0.947	0.716	1.86E-01	1.00E+00	0.0017	5.9	0.052
LPC C16	988	0.001	0.001	2.48E-01	1.00E+00	0.0013	3.75	0.15
LPC C16:1	987	0.158	0.035	9.13E-06	1.05E-03	0.0196	12.9	0.002
LPC C17	922	-0.008	0.053	8.77E-01	1.00E+00	0.0000	2.58	0.28
LPC C18	988	0.002	0.003	5.66E-01	1.00E+00	0.0003	2.43	0.3
LPC C18:1	988	-0.017	0.006	4.66E-03	5.36E-01	0.0078	2.27	0.32
LPC C18:2	988	-0.010	0.003	2.18E-03	2.51E-01	0.0091	9.02	0.011
LPC C18:3	840	-0.016	0.142	9.08E-01	1.00E+00	0.0000	9.38	0.009
LPC C20:3	919	0.113	0.048	1.80E-02	1.00E+00	0.0060	1.71	0.43
LPC C20:4	986	0.014	0.017	4.10E-01	1.00E+00	0.0007	2.14	0.34
LPC C20:5	799	0.403	0.162	1.28E-02	1.00E+00	0.0075	2.54	0.28

LPC C22:6	871	-0.099	0.062	1.12E-01	1.00E+00	0.0028	0.919	0.63
PCaa C32	988	-0.014	0.006	2.76E-02	1.00E+00	0.0048	3.46	0.18
PCaa C32:1	922	0.019	0.005	2.82E-04	3.24E-02	0.0137	4.07	0.13
PCaa C34:1	987	-0.001	0.001	4.55E-02	1.00E+00	0.0040	10.3	0.006
PCaa C34:2	922	-0.001	0.000	2.49E-02	1.00E+00	0.0053	2.84	0.24
PCaa C34:3	988	0.007	0.007	2.71E-01	1.00E+00	0.0012	7.3	0.026
PCaa C34:4	938	0.242	0.045	8.30E-08	9.54E-06	0.0291	0.579	0.75
PCaa C36:2	987	0.000	0.001	7.11E-01	1.00E+00	0.0001	4.19	0.12
PCaa C36:3	988	0.001	0.001	5.56E-01	1.00E+00	0.0003	7.21	0.027
PCaa C36:4	923	0.001	0.001	2.44E-01	1.00E+00	0.0014	7.36	0.025
PCaa C36:5	918	0.013	0.006	1.99E-02	1.00E+00	0.0057	6.02	0.049
PCaa C38	799	-0.017	0.031	5.84E-01	1.00E+00	0.0004	0.33	0.85
PCaa C38:3	800	0.016	0.003	7.31E-07	8.41E-05	0.0299	2.23	0.33
PCaa C38:4	817	0.005	0.001	4.84E-05	5.56E-03	0.0193	0.254	0.88
PCaa C38:5	872	0.004	0.003	1.67E-01	1.00E+00	0.0021	4.64	0.1
PCaa C38:6	988	-0.002	0.002	1.89E-01	1.00E+00	0.0017	5.88	0.053

PCaa C40:4	790	0.068	0.027	1.17E-02	1.00E+00	0.0078	0.172	0.92
PCaa C40:5	804	0.026	0.011	2.23E-02	1.00E+00	0.0063	2.71	0.26
PCaa C40:6	987	0.007	0.004	9.70E-02	1.00E+00	0.0027	5.24	0.07

PCae C34:1	988	-0.035	0.012	3.97E-03	4.57E-01	0.0081	0.264	0.88
PCae C34:2	921	-0.011	0.009	2.26E-01	1.00E+00	0.0015	2.8	0.25
PCae C34:3	988	-0.020	0.011	5.47E-02	1.00E+00	0.0036	7.58	0.023
PCae C36:1	873	-0.035	0.009	9.66E-05	1.11E-02	0.0164	1.36	0.51
PCae C36:2	789	-0.050	0.011	2.61E-06	3.00E-04	0.0262	2.82	0.24
PCae C36:3	988	-0.010	0.013	4.19E-01	1.00E+00	0.0006	2.12	0.35
PCae C36:4	920	0.007	0.006	2.37E-01	1.00E+00	0.0015	2.66	0.26
PCae C36:5	988	0.006	0.009	4.54E-01	1.00E+00	0.0006	3.06	0.22
PCae C38	814	-0.031	0.041	4.45E-01	1.00E+00	0.0007	1.94	0.38
PCae C38:3	816	-0.024	0.011	2.73E-02	1.00E+00	0.0058	0.634	0.73
PCae C38:4	805	-0.019	0.009	3.35E-02	1.00E+00	0.0055	4.22	0.12
PCae C38:5	921	-0.003	0.006	5.77E-01	1.00E+00	0.0003	1.49	0.47

PCae C38:6	872	0.012	0.014	3.77E-01	1.00E+00	0.0009	0.465	0.79
PCae C40	771	-0.012	0.015	4.17E-01	1.00E+00	0.0008	1.24	0.54
PCae C40:4	777	-0.095	0.031	2.56E-03	2.94E-01	0.0111	0.1	0.95
PCae C40:6	805	-0.063	0.029	2.99E-02	1.00E+00	0.0056	0.0243	0.99
SM C32:2	729	1.632	0.152	4.07E-25	4.68E-23	0.1331	24.8	<0.0001
SM C35:1	938	-0.020	0.031	5.20E-01	1.00E+00	0.0004	0.86	0.65
SM C36:1	988	0.005	0.005	2.87E-01	1.00E+00	0.0011	3.51	0.17
SM C36:2	855	0.023	0.010	1.58E-02	1.00E+00	0.0066	8.24	0.016

SM C41:1	872	0.002	0.011	8.83E-01	1.00E+00	0.0000	1.42	0.49
SM C41:2	871	0.021	0.015	1.57E-01	1.00E+00	0.0022	1.56	0.46
SM C42:1	821	0.002	0.006	7.59E-01	1.00E+00	0.0001	0.467	0.79
SM C42:2	807	0.004	0.003	2.44E-01	1.00E+00	0.0016	0.988	0.61
NEFA 12:0	927	-0.027	0.008	3.31E-04	3.80E-02	0.0135	3.71	0.16
NEFA 14:0	970	-0.012	0.004	3.22E-03	3.70E-01	0.0086	0.235	0.89
NEFA 14:1	967	-0.033	0.024	1.74E-01	1.00E+00	0.0019	2.29	0.32
NEFA 16:0	971	-0.001	0.001	4.28E-02	1.00E+00	0.0041	0.23	0.89
NEFA 16:1	942	-0.005	0.002	2.67E-02	1.00E+00	0.0050	1.62	0.44
NEFA 16:2	947	0.111	0.153	4.68E-01	1.00E+00	0.0005	0.167	0.92
NEFA 17:0	954	-0.056	0.020	6.00E-03	6.90E-01	0.0076	1.07	0.59
NEFA 17:1	941	-0.053	0.032	9.54E-02	1.00E+00	0.0029	0.668	0.72
NEFA 18:1	972	-0.001	0.000	2.37E-02	1.00E+00	0.0051	0.516	0.77
NEFA 18:2	969	-0.002	0.001	1.76E-01	1.00E+00	0.0018	0.236	0.89
NEFA 18:3	971	-0.010	0.006	7.43E-02	1.00E+00	0.0032	3.2	0.2
NEFA 19:1	959	-0.147	0.065	2.39E-02	1.00E+00	0.0051	1.46	0.48
NEFA 20:2	871	-0.033	0.043	4.35E-01	1.00E+00	0.0007	1.57	0.46
NEFA 20:3	836	0.060	0.031	5.28E-02	1.00E+00	0.0045	2.3	0.32
NEFA 20:4	944	0.011	0.009	2.05E-01	1.00E+00	0.0017	0.393	0.82
NEFA 20:5	891	0.010	0.073	8.90E-01	1.00E+00	0.0000	0.835	0.66

NEFA 22:4	943	0.131	0.091	1.52E-01	1.00E+00	0.0021	0.455	0.8
NEFA 22:5	972	-0.018	0.048	7.08E-01	1.00E+00	0.0001	2.75	0.25
NEFA 22:6	972	-0.011	0.022	6.08E-01	1.00E+00	0.0003	1.43	0.49
NEFA 24:0	925	-1.130	0.351	1.32E-03	1.52E-01	0.0107	4.77	0.09
NEFA 24:4	820	4.526	1.835	1.39E-02	1.00E+00	0.0073	1.41	0.49
NEFA 26:1	927	1.144	0.628	6.89E-02	1.00E+00	0.0035	4.77	0.09
Sum AA	887	0.000	0.000	1.18E-01	1.00E+00	0.0027	4.77	0.09
Sum Carn	853	0.008	0.002	6.84E-04	7.87E-02	0.0131	5.21	0.07
Sum LPC	665	0.000	0.001	7.08E-01	1.00E+00	0.0002	2.15	0.34
Sum PCaa	680	0.000	0.000	3.76E-01	1.00E+00	0.0011	2.21	0.33
Sum PCae	653	-0.001	0.001	6.25E-01	1.00E+00	0.0004	2.41	0.3
Sum SM	611	0.003	0.002	7.35E-02	1.00E+00	0.0050	2.82	0.24
Sum NEFA	564	0.000	0.000	6.18E-01	1.00E+00	0.0004	1.51	0.47

Supplemental Table 3 Results of the adjusted linear models and individual participant meta-analysis of the association of metabolites with weight. Statistical significance was evaluated using Bonferroni corrected p-values (p.adjust) < 0.05 (with dark grey background). AA, amino acids; Carn, Acylcarnitines; lyso.PC, lyso-phosphatidylcholine; NEFA, nonesterified fatty acid; PCaa, Diacyl-phosphatidylcholine; PCae, Alkyl-acyl-phosphatidylcholine; SM, sphingomyelin.

CHOP 5.5 years

Metabolite	N	Estimate	Std..Error	P	P.adjusted	PartialRsquared
Ala	388	0.001	0.002	5.99E-01	1.00E+00	0.001
Arg	386	0.011	0.010	2.40E-01	1.00E+00	0.004
Asn	388	-0.034	0.016	3.57E-02	1.00E+00	0.011
Asp	386	0.120	0.052	2.07E-02	1.00E+00	0.014
Cit	388	-0.030	0.022	1.75E-01	1.00E+00	0.005
Gln	388	-0.003	0.001	3.75E-02	1.00E+00	0.011
Glu	388	0.001	0.002	6.97E-01	1.00E+00	0.000
Gly	388	-0.004	0.004	3.18E-01	1.00E+00	0.003
His	388	-0.006	0.009	4.93E-01	1.00E+00	0.001
Ile	388	0.014	0.010	1.57E-01	1.00E+00	0.005
Leu	388	0.007	0.006	2.82E-01	1.00E+00	0.003
Lys	388	0.001	0.005	8.93E-01	1.00E+00	0.000
Met	388	0.028	0.036	4.41E-01	1.00E+00	0.002
Orn	388	0.001	0.009	9.41E-01	1.00E+00	0.000
Phe	388	0.009	0.015	5.40E-01	1.00E+00	0.001
Pro	388	-0.002	0.003	5.50E-01	1.00E+00	0.001
Ser	387	0.002	0.008	8.14E-01	1.00E+00	0.000
Thr	388	0.013	0.006	2.38E-02	1.00E+00	0.013
Trp	387	0.019	0.015	2.10E-01	1.00E+00	0.004
Tyr	388	0.021	0.011	5.03E-02	1.00E+00	0.010
Val	388	0.005	0.003	1.22E-01	1.00E+00	0.006
H1	387	0.000	0.000	9.77E-02	1.00E+00	0.007
Carn	393	0.071	0.019	1.44E-04	3.35E-02	0.036
Carn C2	393	-0.096	0.048	4.44E-02	1.00E+00	0.010
Carn C3	393	4.313	1.780	1.58E-02	1.00E+00	0.015
Carn C3:1	276	47.904	30.019	1.12E-01	1.00E+00	0.009
Carn C4	393	-0.427	1.626	7.93E-01	1.00E+00	0.000
Carn C4:0:OH	276	-3.599	2.156	9.62E-02	1.00E+00	0.010
Carn C4:1	351	4.226	5.444	4.38E-01	1.00E+00	0.002
Carn C5	393	13.412	4.651	4.15E-03	9.66E-01	0.021
Carn C5:0:OH	317	806.765	377.048	3.32E-02	1.00E+00	0.014
Carn C6	318	-6.608	5.913	2.65E-01	1.00E+00	0.004
Carn C6:1	208	33.106	55.884	5.54E-01	1.00E+00	0.002
Carn C8	325	-1.051	1.210	3.86E-01	1.00E+00	0.002
Carn C8:1	393	4.545	3.173	1.53E-01	1.00E+00	0.005
Carn C9	393	11.976	12.047	3.21E-01	1.00E+00	0.003

Carn C10	392	-0.319	0.757	6.74E-01	1.00E+00	0.000
Carn C10:1	393	1.459	1.005	1.47E-01	1.00E+00	0.005
Carn C10:2	393	4.982	9.043	5.82E-01	1.00E+00	0.001
Carn C12::DC	262	1.181	2.524	6.40E-01	1.00E+00	0.001
Carn C12	392	-1.912	2.050	3.52E-01	1.00E+00	0.002
Carn C12:1	393	0.101	0.752	8.93E-01	1.00E+00	0.000
Carn C14	393	-2.904	5.770	6.15E-01	1.00E+00	0.001
Carn C14:1	391	-0.989	1.463	4.99E-01	1.00E+00	0.001
Carn C14:2	393	-1.268	6.654	8.49E-01	1.00E+00	0.000
Carn C14:2:OH	251	5.211	20.144	7.96E-01	1.00E+00	0.000
Carn C15	393	-3.255	16.182	8.41E-01	1.00E+00	0.000
Carn C16	392	-0.101	6.219	9.87E-01	1.00E+00	0.000
Carn C16:OH	392	-1.682	6.728	8.03E-01	1.00E+00	0.000
Carn C16:1	290	7.640	37.321	8.38E-01	1.00E+00	0.000
Carn C16:2	276	7.253	22.541	7.48E-01	1.00E+00	0.000
Carn C16:2:OH	256	-31.803	28.175	2.60E-01	1.00E+00	0.005
Carn C18	393	-27.424	12.054	2.34E-02	1.00E+00	0.013
Carn C18:1	393	-1.865	3.917	6.34E-01	1.00E+00	0.001
Carn C18:1:OH	324	-0.750	26.679	9.78E-01	1.00E+00	0.000
Carn C18:2	393	4.201	5.460	4.42E-01	1.00E+00	0.002
Carn C18:2:OH	250	27.237	76.945	7.24E-01	1.00E+00	0.001
Carn C20	391	-27.494	39.050	4.82E-01	1.00E+00	0.001
Carn C20:1	316	-345.418	1015.561	7.34E-01	1.00E+00	0.000
Carn C20:3	393	11.778	25.619	6.46E-01	1.00E+00	0.001
Carn C20:4	393	46.823	290.355	8.72E-01	1.00E+00	0.000
Carn C22	291	-277.097	425.577	5.15E-01	1.00E+00	0.001
Carn C22:5	277	0.783	60.775	9.90E-01	1.00E+00	0.000
Carn C22:6	255	-67.910	249.067	7.85E-01	1.00E+00	0.000
LPC C14	245	0.688	0.281	1.52E-02	1.00E+00	0.024
LPC C15	248	-0.761	0.583	1.93E-01	1.00E+00	0.007
LPC C16	392	0.010	0.006	9.92E-02	1.00E+00	0.007
LPC C16:1	393	0.413	0.170	1.55E-02	1.00E+00	0.015
LPC C17	391	-0.040	0.262	8.80E-01	1.00E+00	0.000
LPC C18	393	0.022	0.015	1.45E-01	1.00E+00	0.005
LPC C18:1	393	0.000	0.033	9.99E-01	1.00E+00	0.000
LPC C18:2	393	0.004	0.017	8.18E-01	1.00E+00	0.000
LPC C18:3	352	0.120	0.579	8.36E-01	1.00E+00	0.000
LPC C20:1	202	-1.542	0.901	8.83E-02	1.00E+00	0.014
LPC C20:3	391	0.344	0.175	4.96E-02	1.00E+00	0.010
LPC C20:4	393	0.168	0.079	3.42E-02	1.00E+00	0.011
LPC C20:5	322	0.782	0.822	3.42E-01	1.00E+00	0.003

LPC C22:6	318	-0.034	0.257	8.94E-01	1.00E+00	0.000
PCaa C20:4	214	0.383	4.173	9.27E-01	1.00E+00	0.000
PCaa C28:2	322	6.493	3.876	9.49E-02	1.00E+00	0.009
PCaa C30	393	0.199	0.108	6.69E-02	1.00E+00	0.009
PCaa C30:2	392	0.558	0.739	4.50E-01	1.00E+00	0.001
PCaa C30:3	392	8.781	2.316	1.74E-04	4.05E-02	0.036
PCaa C30:4	168	10.085	7.322	1.70E-01	1.00E+00	0.011
PCaa C32	393	0.098	0.046	3.37E-02	1.00E+00	0.012
PCaa C32:1	393	0.102	0.032	1.69E-03	3.93E-01	0.025
PCaa C32:2	393	0.416	0.163	1.12E-02	1.00E+00	0.016
PCaa C34:1	393	0.002	0.003	6.15E-01	1.00E+00	0.001
PCaa C34:2	393	0.000	0.002	8.52E-01	1.00E+00	0.000
PCaa C34:3	391	0.112	0.036	2.19E-03	5.09E-01	0.024
PCaa C34:4	393	1.089	0.288	1.82E-04	4.23E-02	0.035
PCaa C36	275	0.312	0.079	9.28E-05	2.16E-02	0.055
PCaa C36:1	393	0.008	0.012	4.98E-01	1.00E+00	0.001
PCaa C36:2	393	0.000	0.003	9.38E-01	1.00E+00	0.000
PCaa C36:3	393	0.004	0.006	5.31E-01	1.00E+00	0.001
PCaa C36:4	393	0.002	0.004	6.43E-01	1.00E+00	0.001
PCaa C36:5	391	0.115	0.047	1.58E-02	1.00E+00	0.015

PCaa C36:6	323	0.333	0.256	1.95E-01	1.00E+00	0.005
PCaa C38	316	0.182	0.148	2.20E-01	1.00E+00	0.005
PCaa C38:3	393	0.029	0.016	6.03E-02	1.00E+00	0.009
PCaa C38:4	393	0.010	0.006	9.09E-02	1.00E+00	0.007
PCaa C38:5	393	0.016	0.017	3.26E-01	1.00E+00	0.002
PCaa C38:6	393	-0.007	0.010	4.87E-01	1.00E+00	0.001
PCaa C40	282	0.350	0.298	2.42E-01	1.00E+00	0.005
PCaa C40:4	392	0.221	0.141	1.18E-01	1.00E+00	0.006
PCaa C40:5	393	0.069	0.051	1.74E-01	1.00E+00	0.005
PCaa C40:6	393	-0.002	0.023	9.31E-01	1.00E+00	0.000
PCaa C42	245	0.608	0.558	2.77E-01	1.00E+00	0.005
PCaa C42:1	211	3.185	1.155	6.34E-03	1.00E+00	0.035
PCaa C42:6	202	-0.105	0.660	8.74E-01	1.00E+00	0.000
PCaa C43:6	251	0.138	0.418	7.42E-01	1.00E+00	0.000
PCaa C44:12	393	0.097	0.298	7.44E-01	1.00E+00	0.000
PCae C30:2	282	0.426	1.647	7.96E-01	1.00E+00	0.000
PCae C32	393	0.299	0.217	1.69E-01	1.00E+00	0.005

PCae C32:1	393	0.223	0.176	2.07E-01	1.00E+00	0.004
PCae C32:2	208	1.274	0.642	4.85E-02	1.00E+00	0.019
PCae C34	393	0.025	0.298	9.34E-01	1.00E+00	0.000
PCae C34:1	393	0.073	0.064	2.53E-01	1.00E+00	0.003
PCae C34:2	393	0.025	0.054	6.45E-01	1.00E+00	0.001
PCae C34:3	393	0.068	0.063	2.87E-01	1.00E+00	0.003
PCae C34:4	248	3.756	1.550	1.61E-02	1.00E+00	0.023
PCae C36	255	-0.097	0.347	7.81E-01	1.00E+00	0.000
PCae C36:1	392	0.063	0.029	3.40E-02	1.00E+00	0.011
PCae C36:2	392	0.006	0.036	8.70E-01	1.00E+00	0.000
PCae C36:3	393	0.068	0.064	2.90E-01	1.00E+00	0.003
PCae C36:4	392	0.028	0.033	3.98E-01	1.00E+00	0.002
PCae C36:5	393	0.035	0.042	4.04E-01	1.00E+00	0.002
PCae C36:6	178	-0.514	0.613	4.03E-01	1.00E+00	0.004
PCae C38	317	0.310	0.129	1.73E-02	1.00E+00	0.018
PCae C38:2	312	0.081	0.042	5.58E-02	1.00E+00	0.012
PCae C38:3	391	0.115	0.040	4.27E-03	9.95E-01	0.021
PCae C38:4	393	-0.033	0.043	4.43E-01	1.00E+00	0.002
PCae C38:5	393	0.007	0.034	8.47E-01	1.00E+00	0.000
PCae C38:6	393	0.061	0.073	4.06E-01	1.00E+00	0.002
PCae C40	393	0.040	0.058	4.87E-01	1.00E+00	0.001
PCae C40:1	237	0.532	0.221	1.68E-02	1.00E+00	0.024
PCae C40:3	322	0.080	0.055	1.48E-01	1.00E+00	0.007
PCae C40:4	353	0.306	0.090	7.99E-04	1.86E-01	0.032

PCae C40:5	393	0.128	0.098	1.90E-01	1.00E+00	0.004
PCae C40:6	393	-0.142	0.125	2.55E-01	1.00E+00	0.003
PCae C42:2	325	0.761	0.323	1.93E-02	1.00E+00	0.017
PCae C42:3	282	-0.112	0.256	6.63E-01	1.00E+00	0.001
PCae C42:4	198	1.328	0.538	1.45E-02	1.00E+00	0.030
PCae C42:5	318	0.327	0.205	1.11E-01	1.00E+00	0.008
PCae C42:6	392	0.403	0.329	2.21E-01	1.00E+00	0.004
SM C21:2	353	6.849	7.337	3.51E-01	1.00E+00	0.002
SM C32:1	393	0.201	0.073	6.03E-03	1.00E+00	0.019
SM C32:2	353	6.511	0.921	8.39E-12	1.95E-09	0.124
SM C33:1	393	0.200	0.130	1.24E-01	1.00E+00	0.006
SM C33:3	185	-2.240	3.427	5.14E-01	1.00E+00	0.002
SM C34:1	392	0.006	0.008	4.58E-01	1.00E+00	0.001
SM C34:2	392	0.204	0.052	1.16E-04	2.71E-02	0.037
SM C34:4	217	-6.449	11.922	5.89E-01	1.00E+00	0.001
SM C35:1	392	0.386	0.218	7.74E-02	1.00E+00	0.008
SM C35:2	203	-1.468	1.605	3.61E-01	1.00E+00	0.004
SM C36:1	391	0.058	0.037	1.22E-01	1.00E+00	0.006
SM C36:2	392	0.254	0.069	2.40E-04	5.59E-02	0.034
SM C36:3	187	2.703	0.728	2.71E-04	6.31E-02	0.070
SM C37:1	203	0.185	0.190	3.31E-01	1.00E+00	0.005
SM C37:3	212	-0.334	1.398	8.11E-01	1.00E+00	0.000
SM C38:2	393	0.032	0.045	4.75E-01	1.00E+00	0.001
SM C39:1	318	0.298	0.111	7.89E-03	1.00E+00	0.022
SM C40:2	243	0.023	0.027	3.93E-01	1.00E+00	0.003

SM C40:4	313	0.506	0.300	9.26E-02	1.00E+00	0.009
SM C40:5	353	0.885	0.542	1.03E-01	1.00E+00	0.008
SM C41:1	392	0.037	0.040	3.59E-01	1.00E+00	0.002
SM C41:2	392	0.085	0.058	1.42E-01	1.00E+00	0.006
SM C42:1	393	0.021	0.027	4.36E-01	1.00E+00	0.002
SM C42:2	392	0.027	0.016	9.70E-02	1.00E+00	0.007
SM C42:3	323	0.050	0.033	1.23E-01	1.00E+00	0.007
SM C42:6	393	-0.041	0.195	8.35E-01	1.00E+00	0.000
SM C43	176	-1.343	1.080	2.15E-01	1.00E+00	0.009
SM C43:1	205	0.752	0.417	7.29E-02	1.00E+00	0.016
SM C43:2	322	0.092	0.221	6.77E-01	1.00E+00	0.001
SM C44:6	238	0.434	0.515	4.00E-01	1.00E+00	0.003
SM C47:6	325	8.262	32.602	8.00E-01	1.00E+00	0.000
NEFA 12:0	180	-0.055	0.066	4.11E-01	1.00E+00	0.004
NEFA 14:0	323	-0.055	0.022	1.11E-02	1.00E+00	0.020
NEFA 14:1	390	-0.022	0.033	5.15E-01	1.00E+00	0.001
NEFA 15:0	180	-0.589	0.273	3.28E-02	1.00E+00	0.025
NEFA 16:0	247	-0.007	0.003	4.03E-02	1.00E+00	0.017
NEFA 16:1	390	-0.018	0.010	6.37E-02	1.00E+00	0.009
NEFA 16:2	310	0.169	0.359	6.39E-01	1.00E+00	0.001
NEFA 17:0	168	0.048	0.118	6.85E-01	1.00E+00	0.001
NEFA 17:1	390	-0.114	0.088	1.98E-01	1.00E+00	0.004
NEFA 17:2	168	-1.474	4.245	7.29E-01	1.00E+00	0.001
NEFA 18:1	390	-0.005	0.002	4.11E-03	9.57E-01	0.021
NEFA 18:2	390	-0.009	0.004	2.19E-02	1.00E+00	0.013
NEFA 18:3	390	-0.123	0.052	1.83E-02	1.00E+00	0.014
NEFA 18:4	168	-5.186	2.416	3.33E-02	1.00E+00	0.027
NEFA 19:1	308	-0.860	0.464	6.47E-02	1.00E+00	0.011
NEFA 20:1	390	-0.328	0.110	2.93E-03	6.82E-01	0.023
NEFA 20:2	390	-0.062	0.060	3.02E-01	1.00E+00	0.003
NEFA 20:3	390	0.060	0.119	6.18E-01	1.00E+00	0.001
NEFA 20:4	389	-0.004	0.040	9.28E-01	1.00E+00	0.000

NEFA 20:5	307	-0.082	0.236	7.30E-01	1.00E+00	0.000
NEFA 22:3	235	-6.277	6.855	3.61E-01	1.00E+00	0.004
NEFA 22:4	390	-0.367	0.511	4.73E-01	1.00E+00	0.001
NEFA 22:5	387	-0.284	0.262	2.80E-01	1.00E+00	0.003
NEFA 22:6	390	-0.052	0.104	6.19E-01	1.00E+00	0.001
NEFA 24:0	168	-3.142	1.804	8.35E-02	1.00E+00	0.018
NEFA 24:1	390	-1.203	0.714	9.27E-02	1.00E+00	0.007
NEFA 24:2	168	31.066	14.568	3.45E-02	1.00E+00	0.027
NEFA 24:3	242	-1.235	10.554	9.07E-01	1.00E+00	0.000
NEFA 24:4	390	-2.790	8.934	7.55E-01	1.00E+00	0.000
NEFA 24:6	167	-38.637	24.693	1.20E-01	1.00E+00	0.015
NEFA 26:0	335	1.279	1.079	2.37E-01	1.00E+00	0.004
NEFA 26:1	315	0.352	1.193	7.68E-01	1.00E+00	0.000
NEFA 26:2	390	0.707	1.487	6.35E-01	1.00E+00	0.001
NEFA 26:3	309	3.624	5.199	4.86E-01	1.00E+00	0.002
NEFA 26:4	390	-17.560	19.207	3.61E-01	1.00E+00	0.002
Pyruvate	385	0.000	0.001	8.48E-01	1.00E+00	0.000
Lactic_acid	390	0.000	0.000	4.33E-01	1.00E+00	0.002
Fumarate	383	-0.191	0.601	7.51E-01	1.00E+00	0.000
3-Methyl-2-oxobutanoate	385	-0.009	0.013	4.95E-01	1.00E+00	0.001
Succininate	388	0.066	0.052	2.05E-01	1.00E+00	0.004
Methylmalonate	390	4.704	2.490	5.96E-02	1.00E+00	0.009
Taurine	388	0.003	0.004	4.44E-01	1.00E+00	0.002
3-Methyl-2-oxovalerate	389	-0.004	0.014	7.77E-01	1.00E+00	0.000
4-Methyl-2-oxovalerate	384	0.002	0.007	7.21E-01	1.00E+00	0.000
Malate	386	0.108	0.102	2.90E-01	1.00E+00	0.003
Alpha-ketoglutarate	379	-0.044	0.032	1.73E-01	1.00E+00	0.005
Alpha-aminoadipate	388	0.669	1.098	5.42E-01	1.00E+00	0.001
Isocitrate	390	0.026	0.032	4.16E-01	1.00E+00	0.002
Citrate	390	0.003	0.002	1.91E-01	1.00E+00	0.004
Beta-hydroxybutyrate	151	0.005	0.007	4.73E-01	1.00E+00	0.003

CHOP 8 years

Metabolite	N	Estimate	Std..Error	P	P.adjusted	PartialRsquared
Ala	355	0.009	0.005	5.56E-02	1.00E+00	0.010
Arg	355	-0.032	0.021	1.34E-01	1.00E+00	0.006
Asn	355	-0.083	0.035	1.90E-02	1.00E+00	0.016
Asp	355	0.098	0.110	3.75E-01	1.00E+00	0.002
Cit	355	-0.163	0.048	8.41E-04	1.60E-01	0.031
Gln	354	-0.007	0.002	1.70E-03	3.23E-01	0.028
Glu	355	0.005	0.004	1.65E-01	1.00E+00	0.005
Gly	355	-0.021	0.008	7.74E-03	1.00E+00	0.020
His	355	-0.002	0.013	8.72E-01	1.00E+00	0.000
Ile	355	0.089	0.033	8.21E-03	1.00E+00	0.020
Leu	355	0.059	0.019	1.99E-03	3.78E-01	0.027
Lys	355	0.021	0.022	3.49E-01	1.00E+00	0.002
Met	355	0.016	0.080	8.41E-01	1.00E+00	0.000
Orn	355	0.040	0.023	8.36E-02	1.00E+00	0.008
Phe	354	0.070	0.037	5.99E-02	1.00E+00	0.010
Pro	355	0.001	0.009	8.98E-01	1.00E+00	0.000
Ser	354	-0.026	0.011	2.50E-02	1.00E+00	0.014
Thr	355	0.003	0.015	8.23E-01	1.00E+00	0.000
Trp	355	0.058	0.035	1.06E-01	1.00E+00	0.007
Tyr	354	0.142	0.027	1.63E-07	3.10E-05	0.075
Val	355	0.031	0.009	6.88E-04	1.31E-01	0.032
H1	355	0.000	0.001	4.09E-01	1.00E+00	0.002
Carn	355	0.054	0.016	9.59E-04	1.82E-01	0.030
Carn C2	355	-0.133	0.131	3.12E-01	1.00E+00	0.003
Carn C3	352	1.480	1.075	1.70E-01	1.00E+00	0.005
Carn C3:1	287	9.366	16.535	5.72E-01	1.00E+00	0.001
Carn C4:0:OH	238	-7.088	4.514	1.18E-01	1.00E+00	0.010
Carn C5	355	75.005	243.526	7.58E-01	1.00E+00	0.000
Carn C5:0:OH	355	9.644	4.356	2.75E-02	1.00E+00	0.014
Carn C6	355	0.551	7.029	9.38E-01	1.00E+00	0.000
Carn C6:1	355	-3.042	9.070	7.38E-01	1.00E+00	0.000
Carn C8	355	-3.035	1.987	1.28E-01	1.00E+00	0.007
Carn C8:1	355	2.491	4.830	6.06E-01	1.00E+00	0.001
Carn C9	355	-10.765	18.762	5.67E-01	1.00E+00	0.001

Carn C10	355	-2.405	1.169	4.04E-02	1.00E+00	0.012
Carn C10:1	354	0.044	1.935	9.82E-01	1.00E+00	0.000
Carn C10:2	355	7.650	13.833	5.81E-01	1.00E+00	0.001
Carn C12	355	-4.060	3.010	1.78E-01	1.00E+00	0.005
Carn C12:1	355	-0.400	1.261	7.52E-01	1.00E+00	0.000
Carn C14	355	-7.312	12.684	5.65E-01	1.00E+00	0.001
Carn C14:1	354	-1.456	2.076	4.83E-01	1.00E+00	0.001
Carn C14:2	355	-2.924	9.629	7.62E-01	1.00E+00	0.000
Carn C14:2:OH	353	-11.033	29.870	7.12E-01	1.00E+00	0.000
Carn C15	355	7.008	68.889	9.19E-01	1.00E+00	0.000
Carn C16	355	-5.175	15.504	7.39E-01	1.00E+00	0.000
Carn C16:0:OH	289	161.487	186.347	3.87E-01	1.00E+00	0.003
Carn C16:1	355	-8.786	13.383	5.12E-01	1.00E+00	0.001
Carn C16:2	355	-35.784	34.637	3.02E-01	1.00E+00	0.003
Carn C16:2:OH	305	20.304	69.064	7.69E-01	1.00E+00	0.000
Carn C18	355	-83.371	33.821	1.42E-02	1.00E+00	0.017
Carn C18:1	355	-11.099	10.207	2.78E-01	1.00E+00	0.003
Carn C18:1:OH	355	-78.817	46.113	8.83E-02	1.00E+00	0.008
Carn C18:2	353	-3.991	16.681	8.11E-01	1.00E+00	0.000
Carn C18:2:OH	233	25.269	125.334	8.40E-01	1.00E+00	0.000
Carn C20	349	-183.214	64.434	4.73E-03	8.99E-01	0.023
Carn C20:1	354	666.021	4735.474	8.88E-01	1.00E+00	0.000
Carn C20:3	355	359.235	178.841	4.53E-02	1.00E+00	0.011
Carn C20:4	305	3143.425	2368.305	1.85E-01	1.00E+00	0.006
Carn C22	222	-5614.830	2122.330	8.75E-03	1.00E+00	0.031
Carn C22:5	254	1070.093	347.980	2.34E-03	4.44E-01	0.036
Carn C22:6	184	255.572	243.994	2.96E-01	1.00E+00	0.006
LPC C16	355	-0.001	0.008	8.79E-01	1.00E+00	0.000
LPC C16:1	355	0.404	0.273	1.40E-01	1.00E+00	0.006
LPC C17	289	0.088	0.429	8.39E-01	1.00E+00	0.000
LPC C18	355	-0.010	0.021	6.33E-01	1.00E+00	0.001
LPC C18:1	355	-0.148	0.048	2.11E-03	4.01E-01	0.026
LPC C18:2	355	-0.083	0.026	1.75E-03	3.32E-01	0.027
LPC C18:3	221	-0.968	1.514	5.23E-01	1.00E+00	0.002
LPC C18:6	181	-0.506	2.204	8.19E-01	1.00E+00	0.000
LPC C20:3	288	0.288	0.391	4.61E-01	1.00E+00	0.002
LPC C20:4	355	-0.016	0.137	9.08E-01	1.00E+00	0.000
LPC C20:5	170	0.849	1.544	5.83E-01	1.00E+00	0.002

LPC C22:5	154	0.440	1.892	8.17E-01	1.00E+00	0.000
LPC C22:6	253	-0.514	0.585	3.81E-01	1.00E+00	0.003
PCaa C30:5	180	368.058	163.495	2.56E-02	1.00E+00	0.028
PCaa C32	355	-0.243	0.091	7.99E-03	1.00E+00	0.020
PCaa C32:1	289	0.080	0.059	1.75E-01	1.00E+00	0.006
PCaa C34:1	355	-0.017	0.006	3.74E-03	7.11E-01	0.024
PCaa C34:2	289	-0.012	0.004	5.31E-03	1.00E+00	0.027
PCaa C34:3	355	-0.049	0.063	4.37E-01	1.00E+00	0.002
PCaa C34:4	305	1.112	0.512	3.06E-02	1.00E+00	0.015
PCaa C36:2	355	-0.008	0.004	6.16E-02	1.00E+00	0.010
PCaa C36:3	355	-0.022	0.012	6.42E-02	1.00E+00	0.010
PCaa C36:4	289	-0.014	0.010	1.56E-01	1.00E+00	0.007
PCaa C36:5	286	-0.018	0.062	7.72E-01	1.00E+00	0.000

PCaa C38	166	-0.321	0.260	2.18E-01	1.00E+00	0.009
PCaa C38:3	167	0.027	0.044	5.39E-01	1.00E+00	0.002
PCaa C38:4	184	0.024	0.014	7.96E-02	1.00E+00	0.017
PCaa C38:5	239	-0.044	0.040	2.66E-01	1.00E+00	0.005
PCaa C38:6	355	-0.030	0.015	4.29E-02	1.00E+00	0.012
PCaa C40:4	156	0.290	0.434	5.04E-01	1.00E+00	0.003
PCaa C40:5	172	-0.123	0.147	4.04E-01	1.00E+00	0.004
PCaa C40:6	354	-0.012	0.038	7.44E-01	1.00E+00	0.000
PCaa C44:12	173	-4.248	1.601	8.71E-03	1.00E+00	0.040

PCae C34:1	355	-0.296	0.106	5.37E-03	1.00E+00	0.022
PCae C34:2	288	-0.157	0.083	5.76E-02	1.00E+00	0.013
PCae C34:3	355	-0.253	0.093	7.07E-03	1.00E+00	0.020
PCae C36:1	239	-0.164	0.067	1.61E-02	1.00E+00	0.024
PCae C36:2	155	-0.411	0.124	1.18E-03	2.25E-01	0.067
PCae C36:3	355	-0.190	0.101	6.02E-02	1.00E+00	0.010
PCae C36:4	288	-0.034	0.051	5.04E-01	1.00E+00	0.002
PCae C36:5	355	-0.051	0.082	5.36E-01	1.00E+00	0.001
PCae C38	183	-0.418	0.387	2.82E-01	1.00E+00	0.006
PCae C38:3	183	-0.166	0.084	4.85E-02	1.00E+00	0.022
PCae C38:4	173	-0.362	0.151	1.80E-02	1.00E+00	0.032
PCae C38:5	289	-0.067	0.059	2.53E-01	1.00E+00	0.005
PCae C38:6	239	0.010	0.157	9.47E-01	1.00E+00	0.000
PCae C40	166	-0.208	0.145	1.53E-01	1.00E+00	0.013
PCae C40:4	172	-0.496	0.290	8.84E-02	1.00E+00	0.017

PCae C40:6	172	-0.616	0.338	7.04E-02	1.00E+00	0.019
SM C21:2	181	-22.526	8.139	6.25E-03	1.00E+00	0.041
SM C31:1	182	12.185	5.141	1.88E-02	1.00E+00	0.030
SM C32:1	355	0.161	0.148	2.77E-01	1.00E+00	0.003
SM C32:2	171	5.820	1.750	1.09E-03	2.06E-01	0.060
SM C33:1	355	-0.224	0.204	2.72E-01	1.00E+00	0.003
SM C34:1	355	-0.042	0.014	3.15E-03	5.99E-01	0.024
SM C34:2	355	0.284	0.094	2.60E-03	4.94E-01	0.025
SM C35:1	305	-0.117	0.390	7.63E-01	1.00E+00	0.000
SM C36:1	355	0.088	0.054	1.04E-01	1.00E+00	0.007
SM C36:2	222	0.351	0.159	2.77E-02	1.00E+00	0.022
SM C36:3	287	1.835	1.113	1.00E-01	1.00E+00	0.009

SM C41:1	239	-0.136	0.113	2.31E-01	1.00E+00	0.006
SM C41:2	238	0.058	0.144	6.89E-01	1.00E+00	0.001
SM C42:1	188	-0.055	0.076	4.67E-01	1.00E+00	0.003
SM C42:2	173	0.029	0.040	4.63E-01	1.00E+00	0.003
NEFA 10:0	339	-0.003	0.130	9.82E-01	1.00E+00	0.000
NEFA 11:0	266	-0.921	4.031	8.19E-01	1.00E+00	0.000
NEFA 12:0	338	-0.467	0.178	9.19E-03	1.00E+00	0.020
NEFA 12:1	221	-0.194	4.526	9.66E-01	1.00E+00	0.000
NEFA 14:0	339	-0.066	0.040	9.35E-02	1.00E+00	0.008
NEFA 14:1	338	-0.021	0.214	9.22E-01	1.00E+00	0.000
NEFA 15:0	339	-0.277	0.186	1.37E-01	1.00E+00	0.007
NEFA 15:1	216	-3.229	2.789	2.48E-01	1.00E+00	0.006
NEFA 16:0	339	-0.005	0.005	3.79E-01	1.00E+00	0.002
NEFA 16:1	339	-0.011	0.022	6.30E-01	1.00E+00	0.001
NEFA 16:2	339	1.780	1.512	2.40E-01	1.00E+00	0.004
NEFA 17:0	339	-0.334	0.185	7.22E-02	1.00E+00	0.010
NEFA 17:1	339	-0.192	0.312	5.38E-01	1.00E+00	0.001
NEFA 18:0	284	-0.031	0.012	9.04E-03	1.00E+00	0.024
NEFA 18:1	339	-0.005	0.004	1.62E-01	1.00E+00	0.006
NEFA 18:2	337	-0.007	0.013	5.78E-01	1.00E+00	0.001
NEFA 18:3	339	-0.062	0.052	2.29E-01	1.00E+00	0.004
NEFA 18:4	337	-1.866	3.360	5.79E-01	1.00E+00	0.001
NEFA 19:0	284	-5.426	1.985	6.66E-03	1.00E+00	0.026
NEFA 19:1	339	-0.804	0.648	2.16E-01	1.00E+00	0.005
NEFA 20:0	339	-3.306	0.893	2.51E-04	4.76E-02	0.039
NEFA 20:1	339	-0.495	0.219	2.46E-02	1.00E+00	0.015
NEFA 20:2	338	0.010	0.379	9.79E-01	1.00E+00	0.000
NEFA 20:3	339	0.283	0.232	2.23E-01	1.00E+00	0.004
NEFA 20:4	339	0.080	0.061	1.88E-01	1.00E+00	0.005

NEFA 20:5	337	0.135	0.615	8.26E-01	1.00E+00	0.000
NEFA 22:0	288	-2.321	3.367	4.91E-01	1.00E+00	0.002
NEFA 22:1	338	-6.119	2.746	2.65E-02	1.00E+00	0.015
NEFA 22:2	284	-12.212	12.568	3.32E-01	1.00E+00	0.003
NEFA 22:3	233	8.242	11.485	4.74E-01	1.00E+00	0.002
NEFA 22:4	338	0.950	0.772	2.19E-01	1.00E+00	0.005
NEFA 22:5	339	-0.080	0.407	8.44E-01	1.00E+00	0.000
NEFA 22:6	339	-0.035	0.185	8.51E-01	1.00E+00	0.000
NEFA 24:0	339	-7.749	3.124	1.36E-02	1.00E+00	0.018
NEFA 24:1	339	-3.351	2.628	2.03E-01	1.00E+00	0.005
NEFA 24:3	150	-53.480	75.569	4.80E-01	1.00E+00	0.003
NEFA 24:4	221	48.800	18.527	9.04E-03	1.00E+00	0.031
NEFA 24:5	167	-25.352	16.358	1.23E-01	1.00E+00	0.014
NEFA 24:6	222	-1.719	11.666	8.83E-01	1.00E+00	0.000
NEFA 26:0	288	-30.214	21.189	1.55E-01	1.00E+00	0.007
NEFA 26:1	339	4.101	4.793	3.93E-01	1.00E+00	0.002
NEFA 26:2	289	2.628	6.401	6.82E-01	1.00E+00	0.001
NEFA 26:3	338	-8.734	10.757	4.17E-01	1.00E+00	0.002
NEFA 26:4	167	-131.581	60.696	3.16E-02	1.00E+00	0.028
NEFA 26:5	182	-25.936	58.406	6.58E-01	1.00E+00	0.001
Pyruvate	349	0.030	0.067	6.55E-01	1.00E+00	0.001
Lactate	349	0.016	0.039	6.91E-01	1.00E+00	0.000
Succininate	247	-0.004	0.003	2.03E-01	1.00E+00	0.007
Taurine	161	0.166	1.152	8.86E-01	1.00E+00	0.000
3-Methyl-2-oxovalerate	335	-0.004	0.014	7.65E-01	1.00E+00	0.000
4-Methyl-2-oxovalerate	297	-0.056	0.012	3.69E-06	7.01E-04	0.070
Malate	298	0.009	0.067	8.92E-01	1.00E+00	0.000
Alpha-ketoglutarate	347	0.000	0.000	8.59E-01	1.00E+00	0.000
Alpha-aminoadipate	347	-0.047	0.083	5.75E-01	1.00E+00	0.001
Isocitrate	349	0.115	0.054	3.26E-02	1.00E+00	0.013
Citrate	226	0.003	0.005	6.49E-01	1.00E+00	0.001
Alpha-ketobutyrate	349	0.040	0.074	5.90E-01	1.00E+00	0.001
Acetoacetate	298	0.024	0.012	5.45E-02	1.00E+00	0.012

UBCS 8 years

Metabolite	N	Estimate	Std..Error	P	P.adjusted	PartialRsquared
Ala	401	0.012	0.003	1.21E-04	2.46E-02	0.036
Arg	400	0.002	0.009	7.84E-01	1.00E+00	0.000
Asn	363	0.027	0.027	3.14E-01	1.00E+00	0.003
Asp	364	0.153	0.051	2.74E-03	5.56E-01	0.024
Cit	402	-0.061	0.027	2.26E-02	1.00E+00	0.013
Gln	401	-0.001	0.003	8.07E-01	1.00E+00	0.000
Glu	402	0.038	0.013	2.61E-03	5.29E-01	0.022
Gly	400	0.000	0.005	9.53E-01	1.00E+00	0.000
His	402	0.033	0.012	5.97E-03	1.00E+00	0.018
Ile	361	0.045	0.021	3.43E-02	1.00E+00	0.012
Leu	402	0.032	0.011	3.63E-03	7.37E-01	0.021
Lys	401	0.021	0.007	5.01E-03	1.00E+00	0.019
Met	402	0.064	0.045	1.51E-01	1.00E+00	0.005
Orn	401	0.012	0.015	4.52E-01	1.00E+00	0.001
Phe	402	0.048	0.021	2.49E-02	1.00E+00	0.012
Pro	398	0.006	0.003	4.99E-02	1.00E+00	0.010
Ser	401	-0.009	0.007	1.76E-01	1.00E+00	0.005
Thr	402	0.020	0.009	2.25E-02	1.00E+00	0.013
Trp	401	0.055	0.018	2.57E-03	5.21E-01	0.022
Tyr	401	0.088	0.015	1.61E-08	3.26E-06	0.076
Val	402	0.019	0.005	3.11E-04	6.31E-02	0.032
Hpro	400	0.034	0.024	1.68E-01	1.00E+00	0.005
H1	400	0.001	0.000	9.19E-04	1.86E-01	0.027
Carn	401	0.154	0.034	1.02E-05	2.07E-03	0.047
Carn C2	401	-0.227	0.090	1.20E-02	1.00E+00	0.015
Carn C3	402	4.847	1.667	3.84E-03	7.80E-01	0.020
Carn C4	402	1.386	2.861	6.28E-01	1.00E+00	0.001
Carn C5	395	4.798	4.603	2.98E-01	1.00E+00	0.003
Carn C8:1	397	3.666	1.580	2.09E-02	1.00E+00	0.013

Carn C10:1	400	-0.568	1.591	7.21E-01	1.00E+00	0.000
Carn C12:1	402	-3.299	1.538	3.25E-02	1.00E+00	0.011
Carn C14:1	401	-4.409	2.170	4.29E-02	1.00E+00	0.010
Carn C16	400	-12.330	5.637	2.93E-02	1.00E+00	0.012
Carn C18	398	-31.883	11.537	5.99E-03	1.00E+00	0.019
Carn C18:1	402	-10.993	4.662	1.89E-02	1.00E+00	0.014
LPC C14	402	3.047	0.490	1.26E-09	2.56E-07	0.087
LPC C15	402	3.199	1.194	7.68E-03	1.00E+00	0.017
LPC C15:1	402	27.167	9.994	6.85E-03	1.00E+00	0.018
LPC C16	402	0.063	0.020	1.50E-03	3.04E-01	0.025
LPC C16:1	401	2.440	0.465	2.52E-07	5.11E-05	0.064
LPC C17	402	-0.449	0.918	6.25E-01	1.00E+00	0.001
LPC C17:1	402	4.970	3.238	1.26E-01	1.00E+00	0.006
LPC C18	402	0.139	0.052	7.45E-03	1.00E+00	0.018
LPC C18:1	402	0.044	0.069	5.20E-01	1.00E+00	0.001
LPC C18:2	402	0.023	0.033	4.97E-01	1.00E+00	0.001
LPC C18:3	401	3.454	1.268	6.71E-03	1.00E+00	0.018
LPC C18:6	402	8.887	3.400	9.29E-03	1.00E+00	0.017
LPC C20	402	-1.721	3.460	6.19E-01	1.00E+00	0.001
LPC C20:1	402	0.314	3.044	9.18E-01	1.00E+00	0.000
LPC C20:2	402	2.592	2.859	3.65E-01	1.00E+00	0.002
LPC C20:3	400	1.251	0.541	2.12E-02	1.00E+00	0.013
LPC C20:4	400	0.569	0.231	1.43E-02	1.00E+00	0.015
LPC C20:5	400	4.960	1.530	1.29E-03	2.61E-01	0.025

LPC C22:4	402	2.256	2.038	2.69E-01	1.00E+00	0.003
LPC C22:5	402	0.103	1.099	9.25E-01	1.00E+00	0.000
LPC C22:6	401	0.282	0.664	6.72E-01	1.00E+00	0.000
PCaa C18	402	-0.215	5.452	9.69E-01	1.00E+00	0.000
PCaa C18:1	402	-12.241	6.473	5.93E-02	1.00E+00	0.009
PCaa C30	402	0.370	0.154	1.70E-02	1.00E+00	0.014
PCaa C30:2	402	0.370	0.524	4.81E-01	1.00E+00	0.001
PCaa C32	402	-0.004	0.034	9.10E-01	1.00E+00	0.000
PCaa C32:1	402	0.100	0.037	7.12E-03	1.00E+00	0.018
PCaa C32:2	402	0.506	0.147	6.12E-04	1.24E-01	0.029
PCaa C32:3	402	3.640	1.407	1.01E-02	1.00E+00	0.016
PCaa C34	400	-0.049	0.102	6.30E-01	1.00E+00	0.001
PCaa C34:1	401	0.000	0.005	9.86E-01	1.00E+00	0.000
PCaa C34:2	401	0.000	0.003	9.01E-01	1.00E+00	0.000
PCaa C34:3	402	0.158	0.055	4.23E-03	8.59E-01	0.020
PCaa C34:4	402	1.520	0.308	1.20E-06	2.44E-04	0.057
PCaa C34:5	402	6.662	2.397	5.71E-03	1.00E+00	0.019
PCaa C34:6	388	2.198	5.210	6.73E-01	1.00E+00	0.000
PCaa C36	401	0.022	0.250	9.30E-01	1.00E+00	0.000
PCaa C36:1	402	0.004	0.014	7.65E-01	1.00E+00	0.000
PCaa C36:2	401	0.007	0.005	2.21E-01	1.00E+00	0.004
PCaa C36:3	402	0.022	0.011	4.77E-02	1.00E+00	0.010
PCaa C36:4	402	0.017	0.007	1.79E-02	1.00E+00	0.014
PCaa C36:5	402	0.106	0.042	1.15E-02	1.00E+00	0.016

PCaa C36:6	402	3.838	0.942	5.58E-05	1.13E-02	0.039
PCaa C38	402	0.268	0.356	4.53E-01	1.00E+00	0.001
PCaa C38:1	398	0.205	0.327	5.31E-01	1.00E+00	0.001
PCaa C38:2	402	0.132	0.141	3.51E-01	1.00E+00	0.002
PCaa C38:3	401	0.110	0.027	5.87E-05	1.19E-02	0.039
PCaa C38:4	402	0.027	0.009	2.42E-03	4.91E-01	0.023
PCaa C38:5	402	0.027	0.019	1.44E-01	1.00E+00	0.005
PCaa C38:6	402	0.015	0.016	3.42E-01	1.00E+00	0.002
PCaa C40	401	-0.092	1.741	9.58E-01	1.00E+00	0.000
PCaa C40:1	395	0.125	3.294	9.70E-01	1.00E+00	0.000
PCaa C40:2	398	4.802	2.913	1.00E-01	1.00E+00	0.007
PCaa C40:3	394	-0.154	1.719	9.29E-01	1.00E+00	0.000
PCaa C40:4	402	0.385	0.189	4.22E-02	1.00E+00	0.010
PCaa C40:5	401	0.162	0.086	5.97E-02	1.00E+00	0.009
PCaa C40:6	402	0.145	0.046	1.81E-03	3.67E-01	0.024
PCaa C42	402	-1.117	1.548	4.71E-01	1.00E+00	0.001
PCaa C42:1	400	-1.415	3.142	6.53E-01	1.00E+00	0.001
PCaa C42:2	396	2.359	5.151	6.47E-01	1.00E+00	0.001
PCaa C42:4	400	1.096	3.469	7.52E-01	1.00E+00	0.000
PCaa C42:5	399	0.123	2.143	9.54E-01	1.00E+00	0.000
PCaa C42:6	402	1.746	1.826	3.40E-01	1.00E+00	0.002
PCaa C43:6	402	-0.414	0.704	5.57E-01	1.00E+00	0.001
PCae C30	402	0.943	1.589	5.53E-01	1.00E+00	0.001
PCae C32	399	-0.088	0.254	7.30E-01	1.00E+00	0.000

PCae C32:1	402	0.053	0.335	8.73E-01	1.00E+00	0.000
PCae C32:2	402	0.961	1.848	6.03E-01	1.00E+00	0.001
PCae C34	400	0.005	0.340	9.88E-01	1.00E+00	0.000
PCae C34:1	402	-0.111	0.103	2.84E-01	1.00E+00	0.003
PCae C34:2	402	0.026	0.077	7.36E-01	1.00E+00	0.000
PCae C34:3	402	0.032	0.087	7.08E-01	1.00E+00	0.000
PCae C34:4	402	4.575	2.355	5.28E-02	1.00E+00	0.009
PCae C36	401	-0.099	0.847	9.07E-01	1.00E+00	0.000
PCae C36:1	402	-0.354	0.187	5.94E-02	1.00E+00	0.009
PCae C36:2	402	-0.217	0.093	1.97E-02	1.00E+00	0.013
PCae C36:3	402	0.068	0.134	6.12E-01	1.00E+00	0.001
PCae C36:4	401	0.112	0.050	2.55E-02	1.00E+00	0.012
PCae C36:5	402	0.136	0.072	5.79E-02	1.00E+00	0.009
PCae C36:6	397	2.467	1.060	2.05E-02	1.00E+00	0.013
PCae C38	401	0.766	0.467	1.01E-01	1.00E+00	0.007
PCae C38:2	401	-0.466	0.421	2.69E-01	1.00E+00	0.003
PCae C38:3	402	-0.141	0.234	5.47E-01	1.00E+00	0.001
PCae C38:4	400	-0.019	0.060	7.44E-01	1.00E+00	0.000
PCae C38:5	401	0.047	0.046	3.16E-01	1.00E+00	0.002
PCae C38:6	402	0.158	0.102	1.22E-01	1.00E+00	0.006
PCae C40	402	0.043	0.191	8.22E-01	1.00E+00	0.000
PCae C40:1	397	0.440	0.432	3.09E-01	1.00E+00	0.003
PCae C40:2	402	-0.351	1.165	7.64E-01	1.00E+00	0.000
PCae C40:3	401	-1.240	1.166	2.88E-01	1.00E+00	0.003
PCae C40:4	402	-0.473	0.376	2.09E-01	1.00E+00	0.004

PCae C40:5	402	-0.534	0.280	5.68E-02	1.00E+00	0.009
PCae C40:6	402	-0.250	0.250	3.19E-01	1.00E+00	0.002
PCae C42	402	-0.712	2.232	7.50E-01	1.00E+00	0.000
PCae C42:1	400	0.646	1.215	5.95E-01	1.00E+00	0.001
PCae C42:2	402	-0.707	1.255	5.74E-01	1.00E+00	0.001
PCae C42:3	401	-1.053	1.214	3.86E-01	1.00E+00	0.002
PCae C42:4	402	-1.257	0.846	1.38E-01	1.00E+00	0.005
PCae C42:5	402	-0.718	0.485	1.40E-01	1.00E+00	0.005
PCae C42:6	402	-1.140	0.735	1.22E-01	1.00E+00	0.006
SM C32:2	326	15.022	1.499	9.39E-21	1.91E-18	0.228
SM C35	401	0.026	0.716	9.71E-01	1.00E+00	0.000
SM C35:1	402	-0.024	0.187	9.00E-01	1.00E+00	0.000
SM C36	401	0.216	0.248	3.83E-01	1.00E+00	0.002
SM C36:1	402	0.012	0.030	6.99E-01	1.00E+00	0.000
SM C36:2	402	0.071	0.057	2.12E-01	1.00E+00	0.004
SM C37:1	401	-0.026	0.341	9.40E-01	1.00E+00	0.000
SM C38:1	402	0.007	0.023	7.50E-01	1.00E+00	0.000
SM C38:2	402	0.015	0.034	6.61E-01	1.00E+00	0.000
SM C38:3	402	1.491	0.843	7.78E-02	1.00E+00	0.008
SM C39:1	402	0.091	0.168	5.88E-01	1.00E+00	0.001
SM C39:2	400	0.572	0.516	2.68E-01	1.00E+00	0.003
SM C39:5	402	1.322	0.854	1.23E-01	1.00E+00	0.006
SM C40:1	399	-0.003	0.024	8.94E-01	1.00E+00	0.000
SM C40:2	402	0.061	0.035	8.34E-02	1.00E+00	0.007
SM C40:3	399	0.110	0.072	1.27E-01	1.00E+00	0.006

SM C40:4	402	0.091	0.091	3.16E-01	1.00E+00	0.002
SM C41:1	402	-0.042	0.085	6.21E-01	1.00E+00	0.001
SM C41:2	402	0.128	0.129	3.23E-01	1.00E+00	0.002
SM C41:3	394	1.167	0.573	4.24E-02	1.00E+00	0.010
SM C42:1	401	-0.002	0.043	9.71E-01	1.00E+00	0.000
SM C42:2	402	0.016	0.021	4.62E-01	1.00E+00	0.001
SM C42:3	402	0.178	0.050	4.39E-04	8.91E-02	0.030
SM C42:4	401	0.279	0.129	3.07E-02	1.00E+00	0.012
SM C42:6	402	0.033	0.271	9.02E-01	1.00E+00	0.000
SM C43	400	-1.169	1.243	3.48E-01	1.00E+00	0.002
SM C43:1	402	-0.280	0.521	5.91E-01	1.00E+00	0.001
SM C43:2	402	-0.012	0.336	9.71E-01	1.00E+00	0.000
SM C43:3	400	-0.345	1.420	8.08E-01	1.00E+00	0.000
SM C44:2	398	-0.357	1.807	8.43E-01	1.00E+00	0.000
SM C44:6	400	0.540	0.567	3.42E-01	1.00E+00	0.002
NEFA 10:0	323	-0.989	0.264	2.11E-04	4.28E-02	0.041
NEFA 12:0	398	-0.170	0.056	2.56E-03	5.20E-01	0.022
NEFA 12:1	395	-0.435	0.256	9.03E-02	1.00E+00	0.007
NEFA 14:0	401	-0.104	0.032	1.07E-03	2.18E-01	0.026
NEFA 14:1	399	-0.630	0.202	1.90E-03	3.86E-01	0.024
NEFA 15:0	394	-0.389	0.160	1.57E-02	1.00E+00	0.015
NEFA 15:1	335	-3.679	2.220	9.85E-02	1.00E+00	0.008
NEFA 16:0	401	-0.015	0.004	4.89E-04	9.92E-02	0.030
NEFA 16:1	400	-0.065	0.017	1.98E-04	4.03E-02	0.034
NEFA 16:2	398	-1.699	1.656	3.05E-01	1.00E+00	0.003
NEFA 17:0	399	-0.359	0.164	2.94E-02	1.00E+00	0.012
NEFA 17:1	399	-0.521	0.238	2.90E-02	1.00E+00	0.012
NEFA 18:0	398	-0.033	0.011	3.54E-03	7.19E-01	0.021
NEFA 18:1	402	-0.009	0.003	9.39E-04	1.91E-01	0.027
NEFA 18:2	401	-0.031	0.011	3.14E-03	6.37E-01	0.021
NEFA 18:3	401	-0.159	0.085	6.13E-02	1.00E+00	0.009
NEFA 19:1	402	-1.331	0.527	1.20E-02	1.00E+00	0.015
NEFA 20:0	360	-2.137	0.938	2.34E-02	1.00E+00	0.014
NEFA 20:2	351	-0.574	0.439	1.91E-01	1.00E+00	0.005
NEFA 20:3	285	0.065	0.560	9.08E-01	1.00E+00	0.000
NEFA 20:4	402	0.024	0.193	9.00E-01	1.00E+00	0.000

NEFA 20:5	387	-0.305	1.281	8.12E-01	1.00E+00	0.000
NEFA 22:4	402	-0.202	1.001	8.40E-01	1.00E+00	0.000
NEFA 22:5	402	-0.254	0.515	6.22E-01	1.00E+00	0.001
NEFA 22:6	402	-0.057	0.291	8.44E-01	1.00E+00	0.000
NEFA 24:0	399	-0.884	3.252	7.86E-01	1.00E+00	0.000
						0.003
NEFA 24:2	355	14.563	13.673	2.88E-01	1.00E+00	
						0.000
NEFA 24:4	396	3.671	16.184	8.21E-01	1.00E+00	0.003
NEFA 24:5	401	15.713	15.117	2.99E-01	1.00E+00	0.011
NEFA 26:1	402	11.851	5.691	3.79E-02	1.00E+00	0.010

GINI-LISA 10 years

Metabolite	N	Estimate	Std..Error	P	P.adjusted	PartialRsquared
Ala	249	-0.001	0.005	9.01E-01	1.00E+00	0.000
Arg	249	-0.001	0.022	9.80E-01	1.00E+00	0.000
Asn	249	-0.081	0.040	4.24E-02	1.00E+00	0.016
Asp	248	0.110	0.041	8.28E-03	1.00E+00	0.027
Cit	249	-0.090	0.052	8.67E-02	1.00E+00	0.012
Gln	220	-0.009	0.004	3.43E-02	1.00E+00	0.020
Glu	249	0.018	0.009	3.33E-02	1.00E+00	0.018
Gly	249	-0.007	0.007	3.15E-01	1.00E+00	0.004
His	249	-0.009	0.022	6.85E-01	1.00E+00	0.001
Ile	249	0.016	0.033	6.15E-01	1.00E+00	0.001
Leu	249	0.031	0.018	8.18E-02	1.00E+00	0.012
Lys	249	0.016	0.012	1.79E-01	1.00E+00	0.007
Met	249	-0.142	0.095	1.35E-01	1.00E+00	0.009
Orn	249	0.017	0.016	3.10E-01	1.00E+00	0.004
Phe	249	0.056	0.028	4.48E-02	1.00E+00	0.016
Pro	249	0.000	0.007	9.57E-01	1.00E+00	0.000
Ser	249	-0.008	0.015	6.23E-01	1.00E+00	0.001
Thr	249	-0.007	0.015	6.58E-01	1.00E+00	0.001
Trp	249	0.006	0.038	8.70E-01	1.00E+00	0.000
Tyr	249	0.059	0.029	4.12E-02	1.00E+00	0.016
Val	249	0.017	0.009	6.98E-02	1.00E+00	0.013
H1	251	0.000	0.001	7.75E-01	1.00E+00	0.000
Carn	190	0.224	0.097	2.27E-02	1.00E+00	0.026
Carn C2	194	-0.012	0.291	9.67E-01	1.00E+00	0.000
Carn C3	206	13.383	6.531	4.17E-02	1.00E+00	0.020
Carn C4	163	10.600	7.744	1.73E-01	1.00E+00	0.011
Carn C5	152	28.811	17.736	1.06E-01	1.00E+00	0.017
Carn C6	250	-2.643	11.606	8.20E-01	1.00E+00	0.000
Carn C8	251	-0.683	1.566	6.63E-01	1.00E+00	0.001
Carn C8:1	250	6.543	7.317	3.72E-01	1.00E+00	0.003
Carn C9	229	-17.123	18.188	3.47E-01	1.00E+00	0.004

Carn C10	250	-1.997	1.213	1.01E-01	1.00E+00	0.011
Carn C10:1	251	-1.565	1.984	4.31E-01	1.00E+00	0.002
Carn C10:2	153	-16.250	11.402	1.56E-01	1.00E+00	0.013
Carn C12	250	-4.375	2.770	1.16E-01	1.00E+00	0.010
Carn C12:1	222	-0.923	1.362	4.99E-01	1.00E+00	0.002
Carn C14	161	-26.077	23.949	2.78E-01	1.00E+00	0.007
Carn C14:1	250	-5.577	4.265	1.92E-01	1.00E+00	0.007
Carn C14:2	163	-12.822	17.249	4.58E-01	1.00E+00	0.003
Carn C16	229	0.731	18.015	9.68E-01	1.00E+00	0.000
Carn C16:1	200	23.570	29.433	4.24E-01	1.00E+00	0.003
Carn C18	188	-15.426	21.787	4.80E-01	1.00E+00	0.003
Carn C18:1	251	8.939	10.920	4.14E-01	1.00E+00	0.003
Carn C18:2	178	40.252	26.268	1.27E-01	1.00E+00	0.013

LPC C14	251	0.697	0.517	1.79E-01	1.00E+00	0.007
LPC C15	233	-0.038	1.263	9.76E-01	1.00E+00	0.000
LPC C16	251	0.017	0.019	3.59E-01	1.00E+00	0.003
LPC C16:1	251	1.618	0.569	4.80E-03	1.00E+00	0.031
LPC C17	251	-0.589	0.809	4.68E-01	1.00E+00	0.002
LPC C17:1	233	-1.688	3.791	6.57E-01	1.00E+00	0.001
LPC C18	251	0.023	0.055	6.76E-01	1.00E+00	0.001
LPC C18:1	251	-0.171	0.107	1.12E-01	1.00E+00	0.010
LPC C18:2	251	-0.206	0.056	2.71E-04	6.59E-02	0.051
LPC C18:3	233	-3.756	1.927	5.25E-02	1.00E+00	0.016
LPC C18:6	151	3.688	4.971	4.59E-01	1.00E+00	0.003
LPC C20:2	135	2.747	4.295	5.24E-01	1.00E+00	0.003
LPC C20:3	251	0.344	0.736	6.41E-01	1.00E+00	0.001
LPC C20:4	251	0.062	0.267	8.17E-01	1.00E+00	0.000
LPC C20:5	248	0.679	2.406	7.78E-01	1.00E+00	0.000

LPC C22:5	186	-1.227	3.101	6.93E-01	1.00E+00	0.001
LPC C22:6	233	-0.479	0.834	5.67E-01	1.00E+00	0.001
PCaa C30	251	-0.468	0.378	2.18E-01	1.00E+00	0.006
PCaa C30:2	177	-3.587	2.578	1.66E-01	1.00E+00	0.011
PCaa C30:3	172	1.142	7.316	8.76E-01	1.00E+00	0.000
PCaa C30:4	126	2.959	12.530	8.14E-01	1.00E+00	0.000
PCaa C32	251	-0.263	0.168	1.18E-01	1.00E+00	0.010
PCaa C32:1	251	0.177	0.088	4.50E-02	1.00E+00	0.016
PCaa C32:2	251	-0.373	0.401	3.53E-01	1.00E+00	0.003
PCaa C32:3	196	0.366	2.869	8.99E-01	1.00E+00	0.000
PCaa C34:1	251	-0.005	0.011	6.63E-01	1.00E+00	0.001
PCaa C34:2	251	-0.014	0.006	2.57E-02	1.00E+00	0.019
PCaa C34:3	251	-0.065	0.107	5.43E-01	1.00E+00	0.001
PCaa C34:4	251	0.627	0.717	3.83E-01	1.00E+00	0.003
PCaa C34:5	166	3.676	6.550	5.75E-01	1.00E+00	0.002
PCaa C36	191	-0.473	0.285	9.88E-02	1.00E+00	0.014
PCaa C36:1	251	-0.011	0.041	7.81E-01	1.00E+00	0.000
PCaa C36:2	251	-0.015	0.009	9.75E-02	1.00E+00	0.011
PCaa C36:3	251	-0.006	0.017	7.27E-01	1.00E+00	0.000
PCaa C36:4	251	0.014	0.014	3.08E-01	1.00E+00	0.004
PCaa C36:5	250	0.074	0.096	4.43E-01	1.00E+00	0.002

PCaa C36:6	230	0.658	1.129	5.61E-01	1.00E+00	0.001
PCaa C38	251	-0.454	0.444	3.08E-01	1.00E+00	0.004
PCaa C38:2	116	-0.127	0.379	7.39E-01	1.00E+00	0.001
PCaa C38:3	251	0.094	0.038	1.42E-02	1.00E+00	0.023
PCaa C38:4	251	0.034	0.018	5.93E-02	1.00E+00	0.014
PCaa C38:5	251	0.028	0.039	4.70E-01	1.00E+00	0.002
PCaa C38:6	251	0.001	0.025	9.55E-01	1.00E+00	0.000
PCaa C40	183	-2.124	1.194	7.70E-02	1.00E+00	0.017
PCaa C40:1	199	0.726	0.893	4.17E-01	1.00E+00	0.003
PCaa C40:3	138	-0.819	0.775	2.93E-01	1.00E+00	0.008
PCaa C40:4	251	0.110	0.373	7.69E-01	1.00E+00	0.000
PCaa C40:5	251	0.114	0.146	4.37E-01	1.00E+00	0.002
PCaa C40:6	251	0.074	0.062	2.33E-01	1.00E+00	0.006
PCaa C42	237	-2.601	1.672	1.21E-01	1.00E+00	0.010
PCaa C42:1	123	-6.110	3.160	5.55E-02	1.00E+00	0.030
PCaa C42:4	121	-2.439	2.225	2.75E-01	1.00E+00	0.010
PCaa C42:5	160	-7.675	2.673	4.65E-03	1.00E+00	0.049
PCaa C42:6	137	-3.327	1.595	3.89E-02	1.00E+00	0.031
PCaa C43:6	248	-3.752	1.401	7.92E-03	1.00E+00	0.028
PCaa C44:12	221	-2.827	1.938	1.46E-01	1.00E+00	0.009
PCae C32	251	-1.145	0.609	6.14E-02	1.00E+00	0.014

PCae C32:1	251	-1.218	0.571	3.37E-02	1.00E+00	0.018
PCae C32:2	217	-0.910	1.555	5.59E-01	1.00E+00	0.002
PCae C34	230	-0.344	1.082	7.51E-01	1.00E+00	0.000
PCae C34:1	251	-0.486	0.226	3.22E-02	1.00E+00	0.018
PCae C34:2	251	-0.387	0.168	2.24E-02	1.00E+00	0.020
PCae C34:3	251	-0.620	0.187	1.08E-03	2.61E-01	0.041
PCae C34:4	144	-3.678	5.019	4.65E-01	1.00E+00	0.004
PCae C36	181	-3.532	2.182	1.07E-01	1.00E+00	0.014
PCae C36:1	251	-0.172	0.145	2.38E-01	1.00E+00	0.005
PCae C36:2	251	-0.318	0.132	1.70E-02	1.00E+00	0.022
PCae C36:3	251	-0.387	0.222	8.19E-02	1.00E+00	0.012
PCae C36:4	251	-0.068	0.095	4.78E-01	1.00E+00	0.002
PCae C36:5	251	-0.105	0.140	4.52E-01	1.00E+00	0.002
PCae C36:6	128	-3.806	3.584	2.90E-01	1.00E+00	0.009
PCae C38	250	-0.591	0.508	2.45E-01	1.00E+00	0.005
PCae C38:2	251	-0.233	0.237	3.27E-01	1.00E+00	0.004
PCae C38:3	251	-0.050	0.165	7.63E-01	1.00E+00	0.000
PCae C38:4	251	-0.201	0.129	1.20E-01	1.00E+00	0.009
PCae C38:5	251	-0.088	0.109	4.19E-01	1.00E+00	0.003
PCae C38:6	251	-0.131	0.217	5.47E-01	1.00E+00	0.001
PCae C40	222	-0.086	0.175	6.26E-01	1.00E+00	0.001
PCae C40:1	233	-0.946	0.561	9.29E-02	1.00E+00	0.012
PCae C40:2	170	0.105	0.583	8.58E-01	1.00E+00	0.000
PCae C40:3	188	0.214	0.276	4.39E-01	1.00E+00	0.003
PCae C40:4	222	-0.817	0.445	6.77E-02	1.00E+00	0.015

PCae C40:5	251	-0.534	0.308	8.37E-02	1.00E+00	0.012
PCae C40:6	251	-0.740	0.378	5.12E-02	1.00E+00	0.015
PCae C42	193	-0.172	1.483	9.08E-01	1.00E+00	0.000
PCae C42:1	171	-1.186	0.810	1.45E-01	1.00E+00	0.012
PCae C42:2	204	-1.669	1.326	2.10E-01	1.00E+00	0.008
PCae C42:3	211	-1.584	0.778	4.30E-02	1.00E+00	0.019
PCae C42:4	189	-1.487	1.647	3.68E-01	1.00E+00	0.004
PCae C42:5	231	-1.156	0.585	4.96E-02	1.00E+00	0.016
PCae C42:6	251	-2.537	1.057	1.72E-02	1.00E+00	0.022
SM C30:1	209	3.397	2.914	2.45E-01	1.00E+00	0.006
SM C31:1	125	4.927	7.411	5.07E-01	1.00E+00	0.003
SM C32:1	251	0.124	0.218	5.70E-01	1.00E+00	0.001
SM C32:2	251	10.062	1.951	5.15E-07	1.25E-04	0.094
SM C33:1	251	-0.457	0.339	1.78E-01	1.00E+00	0.007
SM C33:2	183	21.642	8.023	7.66E-03	1.00E+00	0.038
SM C33:3	180	-1.787	14.467	9.02E-01	1.00E+00	0.000
SM C34:1	251	-0.049	0.022	3.12E-02	1.00E+00	0.018
SM C34:2	251	0.230	0.153	1.35E-01	1.00E+00	0.009
SM C35	167	-1.647	3.249	6.13E-01	1.00E+00	0.002
SM C35:1	251	0.022	0.682	9.75E-01	1.00E+00	0.000
SM C35:2	202	2.368	4.199	5.73E-01	1.00E+00	0.002
SM C36:1	251	0.107	0.108	3.24E-01	1.00E+00	0.004
SM C36:2	251	0.549	0.195	5.24E-03	1.00E+00	0.030
SM C36:3	251	4.193	2.347	7.52E-02	1.00E+00	0.012
SM C37:1	127	-0.338	1.161	7.71E-01	1.00E+00	0.001
SM C37:3	170	-3.739	4.385	3.95E-01	1.00E+00	0.004
SM C38:1	194	0.017	0.060	7.78E-01	1.00E+00	0.000
SM C38:2	251	-0.110	0.123	3.72E-01	1.00E+00	0.003
SM C38:3	150	5.479	3.673	1.38E-01	1.00E+00	0.014
SM C39:1	251	0.084	0.298	7.77E-01	1.00E+00	0.000
SM C39:2	151	-1.178	1.179	3.20E-01	1.00E+00	0.007
SM C39:5	123	4.564	3.700	2.20E-01	1.00E+00	0.012
SM C40:2	251	-0.016	0.068	8.15E-01	1.00E+00	0.000

SM C40:4	239	0.724	0.909	4.27E-01	1.00E+00	0.003
SM C40:5	215	0.616	1.923	7.49E-01	1.00E+00	0.000
SM C41:1	251	-0.035	0.164	8.32E-01	1.00E+00	0.000
SM C41:2	251	0.115	0.230	6.17E-01	1.00E+00	0.001
SM C42:1	251	-0.066	0.100	5.11E-01	1.00E+00	0.002
SM C42:2	251	0.025	0.056	6.48E-01	1.00E+00	0.001
SM C42:3	233	0.100	0.086	2.48E-01	1.00E+00	0.006
SM C42:4	215	0.361	0.245	1.42E-01	1.00E+00	0.010
SM C42:6	239	-0.068	0.595	9.09E-01	1.00E+00	0.000
SM C43	143	-1.687	2.884	5.60E-01	1.00E+00	0.002
SM C43:1	247	0.067	1.320	9.59E-01	1.00E+00	0.000
SM C43:2	251	2.093	0.759	6.25E-03	1.00E+00	0.029
SM C44:6	209	-0.579	1.171	6.22E-01	1.00E+00	0.001
NEFA 10:0	239	-1.931	0.513	2.12E-04	5.14E-02	0.055
NEFA 12:0	207	-0.251	0.088	4.71E-03	1.00E+00	0.037
NEFA 12:1	151	-0.521	0.783	5.07E-01	1.00E+00	0.003
NEFA 13:0	97	-1.141	1.783	5.24E-01	1.00E+00	0.004
NEFA 13:1	192	-5.360	7.561	4.79E-01	1.00E+00	0.003
NEFA 14:0	250	-0.080	0.074	2.81E-01	1.00E+00	0.005
NEFA 14:1	250	0.138	0.437	7.52E-01	1.00E+00	0.000
NEFA 14:2	113	0.008	0.105	9.40E-01	1.00E+00	0.000
NEFA 16:0	251	-0.008	0.012	4.74E-01	1.00E+00	0.002
NEFA 16:1	222	0.014	0.036	7.04E-01	1.00E+00	0.001
NEFA 16:2	224	1.113	1.894	5.57E-01	1.00E+00	0.001
NEFA 17:0	234	-0.556	0.386	1.51E-01	1.00E+00	0.009
NEFA 17:1	222	0.046	0.592	9.38E-01	1.00E+00	0.000
NEFA 17:2	152	-16.140	13.449	2.32E-01	1.00E+00	0.009
NEFA 18:0	163	-0.051	0.032	1.09E-01	1.00E+00	0.016
NEFA 18:1	251	-0.003	0.008	7.02E-01	1.00E+00	0.001
NEFA 18:2	251	-0.021	0.024	3.68E-01	1.00E+00	0.003
NEFA 18:3	251	-0.047	0.073	5.20E-01	1.00E+00	0.002
NEFA 18:4	229	3.170	4.500	4.82E-01	1.00E+00	0.002
NEFA 19:0	235	-7.029	3.189	2.85E-02	1.00E+00	0.020
NEFA 19:1	238	-0.457	1.036	6.59E-01	1.00E+00	0.001
NEFA 20:0	131	-3.614	2.300	1.19E-01	1.00E+00	0.018
NEFA 20:1	251	-0.319	0.397	4.22E-01	1.00E+00	0.003
NEFA 20:2	202	-0.821	0.627	1.92E-01	1.00E+00	0.008
NEFA 20:3	229	0.753	0.502	1.35E-01	1.00E+00	0.010
NEFA 20:4	222	0.278	0.263	2.91E-01	1.00E+00	0.005

NEFA 20:5	187	-0.057	0.947	9.52E-01	1.00E+00	0.000
NEFA 22:0	93	5.479	4.747	2.52E-01	1.00E+00	0.015
NEFA 22:1	248	-2.825	2.666	2.90E-01	1.00E+00	0.004
NEFA 22:2	137	-2.685	20.102	8.94E-01	1.00E+00	0.000
NEFA 22:3	223	4.112	12.394	7.40E-01	1.00E+00	0.000
NEFA 22:4	223	1.159	1.420	4.16E-01	1.00E+00	0.003
NEFA 22:5	251	-0.169	0.728	8.16E-01	1.00E+00	0.000
NEFA 22:6	251	-0.169	0.310	5.86E-01	1.00E+00	0.001
NEFA 24:0	206	-16.935	5.650	3.06E-03	7.44E-01	0.041
NEFA 24:1	216	-4.804	4.203	2.54E-01	1.00E+00	0.006
NEFA 24:2	164	22.059	34.439	5.23E-01	1.00E+00	0.003
NEFA 24:3	154	-17.524	108.330	8.72E-01	1.00E+00	0.000
NEFA 24:4	222	17.046	27.403	5.35E-01	1.00E+00	0.002
NEFA 24:5	231	18.855	17.436	2.81E-01	1.00E+00	0.005
NEFA 24:6	192	24.567	29.174	4.01E-01	1.00E+00	0.004
NEFA 26:0	162	-48.629	22.428	3.16E-02	1.00E+00	0.027
NEFA 26:1	205	-28.682	21.120	1.76E-01	1.00E+00	0.009
NEFA 26:2	195	-17.631	28.892	5.42E-01	1.00E+00	0.002
NEFA 26:3	169	-2.434	33.916	9.43E-01	1.00E+00	0.000
NEFA 26:4	206	24.622	107.736	8.19E-01	1.00E+00	0.000
NEFA 26:5	194	200.510	153.535	1.93E-01	1.00E+00	0.009
NEFA 26:6	133	-80.570	66.352	2.27E-01	1.00E+00	0.011
Pyruvate	251	0.002	0.008	7.87E-01	1.00E+00	0.000
Lactate	251	0.000	0.000	4.78E-01	1.00E+00	0.002
Fumarate	251	0.458	1.455	7.53E-01	1.00E+00	0.000
3-Methyl-2-oxobutanoate	251	-0.053	0.156	7.33E-01	1.00E+00	0.000
Succininate	251	-0.073	0.214	7.34E-01	1.00E+00	0.000
Methylmalonate	251	1.155	2.353	6.24E-01	1.00E+00	0.001
Taurine	251	0.037	0.020	6.02E-02	1.00E+00	0.014
3-Methyl-2-oxovalerate	251	-0.152	0.145	2.96E-01	1.00E+00	0.004
4-Methyl-2-oxovalerate	251	-0.090	0.086	2.96E-01	1.00E+00	0.004
Malate	251	-0.036	0.813	9.65E-01	1.00E+00	0.000
Alpha-ketoglutarate	250	0.573	0.370	1.23E-01	1.00E+00	0.009
Alpha-aminoadipate	251	14.899	4.687	1.67E-03	4.06E-01	0.038
Isocitrate	238	1.512	2.757	5.84E-01	1.00E+00	0.001
Citrate	251	-0.085	0.061	1.63E-01	1.00E+00	0.008
Alpha-ketobutyrate	232	-0.127	0.146	3.82E-01	1.00E+00	0.003
Acetoacetate	247	-0.049	0.045	2.76E-01	1.00E+00	0.005

Meta Analyses

Metabolite	N	Estimate	Std..Error	P	P.adjusted	PartialRsquared	Q	Qp
Ala	1005	0.006	0.002	9.29E-03	1.00E+00	0.005	4.82	0.09
Arg	1004	-0.002	0.009	8.52E-01	1.00E+00	0.000	2.23	0.33
Asn	967	-0.046	0.020	1.75E-02	1.00E+00	0.004	8.45	0.015
Asp	967	0.118	0.028	2.66E-05	3.06E-03	0.016	0.51	0.78
Cit	1006	-0.098	0.023	2.74E-05	3.15E-03	0.011	3.4	0.18
Gln	975	-0.006	0.002	1.80E-04	2.07E-02	0.009	4.69	0.1
Glu	1006	0.010	0.003	2.21E-03	2.54E-01	0.006	7.72	0.021
Gly	1004	-0.008	0.004	4.41E-02	1.00E+00	0.003	4.99	0.08
His	1006	0.007	0.009	4.04E-01	1.00E+00	0.000	5.19	0.07
Ile	965	0.045	0.016	5.92E-03	6.81E-01	0.006	2.45	0.29
Leu	1006	0.038	0.009	1.72E-05	1.98E-03	0.013	1.76	0.41
Lys	1005	0.019	0.006	3.62E-03	4.16E-01	0.006	0.1	0.95
Met	1006	-0.005	0.040	8.94E-01	1.00E+00	0.000	3.89	0.14
Orn	1005	0.019	0.010	5.55E-02	1.00E+00	0.003	1.08	0.58
Phe	1005	0.057	0.015	2.18E-04	2.51E-02	0.011	0.288	0.87
Pro	1002	0.004	0.003	2.04E-01	1.00E+00	0.001	0.759	0.68
Ser	1004	-0.012	0.006	5.33E-02	1.00E+00	0.002	1.65	0.44
Thr	1006	0.009	0.007	2.32E-01	1.00E+00	0.001	2.69	0.26
Trp	1005	0.040	0.016	1.63E-02	1.00E+00	0.004	1.44	0.49
Tyr	1004	0.091	0.013	8.20E-12	9.43E-10	0.032	4.92	0.09
Val	1006	0.022	0.004	7.42E-07	8.53E-05	0.017	1.47	0.48
H1	1006	0.000	0.000	2.50E-01	1.00E+00	0.001	7.84	0.02
Carn	946	0.068	0.015	7.57E-06	8.70E-04	0.014	9.18	0.01
Carn C2	950	-0.167	0.081	3.95E-02	1.00E+00	0.003	0.734	0.69
Carn C3	960	2.555	0.961	7.98E-03	9.18E-01	0.005	5.62	0.06
Carn C5	902	9.269	3.345	5.71E-03	6.57E-01	0.006	2	0.37
Carn C8:1	1002	3.673	1.815	4.33E-02	1.00E+00	0.003	0.214	0.9

Carn C10:1	1005	-1.230	1.038	2.37E-01	1.00E+00	0.001	0.344	0.84
Carn C12:1	979	-1.227	0.737	9.64E-02	1.00E+00	0.002	2.27	0.32
Carn C14:1	1005	-3.165	1.502	3.53E-02	1.00E+00	0.003	1.33	0.52
Carn C16	984	-9.007	5.964	1.31E-01	1.00E+00	0.002	0.612	0.74
Carn C18	941	-27.756	10.513	8.42E-03	9.69E-01	0.005	2.88	0.24
Carn C18:1	1008	-6.168	4.465	1.67E-01	1.00E+00	0.001	2.9	0.23
LPC C16	1008	0.008	0.007	2.41E-01	1.00E+00	0.001	9.46	0.009
LPC C16:1	1007	0.965	0.219	1.15E-05	1.32E-03	0.013	15.5	<0.0001
LPC C17	942	-0.058	0.334	8.62E-01	1.00E+00	0.000	0.697	0.71
LPC C18	1008	0.009	0.018	6.06E-01	1.00E+00	0.000	7.18	0.028
LPC C18:1	1008	-0.116	0.038	2.34E-03	2.69E-01	0.006	5.85	0.054
LPC C18:2	1008	-0.090	0.020	8.71E-06	1.00E-03	0.012	13.8	0.001
LPC C18:3	855	-0.865	0.895	3.34E-01	1.00E+00	0.001	11.2	0.004
LPC C20:3	939	0.408	0.298	1.71E-01	1.00E+00	0.001	2.2	0.33
LPC C20:4	1006	0.055	0.108	6.13E-01	1.00E+00	0.000	4.8	0.09
LPC C20:5	818	1.936	1.032	6.10E-02	1.00E+00	0.003	4.3	0.12

LPC C22:6	887	-0.395	0.388	3.10E-01	1.00E+00	0.001	0.918	0.63
PCaa C32	1008	-0.071	0.039	6.47E-02	1.00E+00	0.002	7.84	0.02
PCaa C32:1	942	0.109	0.033	1.06E-03	1.21E-01	0.007	0.866	0.65
PCaa C34:1	1007	-0.009	0.004	2.61E-02	1.00E+00	0.003	5.12	0.08
PCaa C34:2	941	-0.007	0.002	1.83E-03	2.10E-01	0.007	7.88	0.019
PCaa C34:3	1008	0.007	0.042	8.76E-01	1.00E+00	0.000	7.49	0.024
PCaa C34:4	958	1.141	0.279	4.59E-05	5.28E-03	0.011	1.52	0.47
PCaa C36:2	1007	-0.007	0.003	2.45E-02	1.00E+00	0.003	6.12	0.047
PCaa C36:3	1008	-0.006	0.008	4.32E-01	1.00E+00	0.000	7.51	0.023
PCaa C36:4	942	0.005	0.006	3.67E-01	1.00E+00	0.001	6.73	0.035
PCaa C36:5	938	0.060	0.036	9.86E-02	1.00E+00	0.002	2.73	0.26

PCaa C38	819	-0.341	0.197	8.31E-02	1.00E+00	0.002	2.25	0.32
PCaa C38:3	819	0.081	0.020	7.92E-05	9.11E-03	0.012	2.55	0.28
PCaa C38:4	837	0.028	0.008	2.82E-04	3.24E-02	0.009	0.175	0.92
PCaa C38:5	892	0.011	0.017	5.36E-01	1.00E+00	0.000	2.77	0.25
PCaa C38:6	1008	-0.010	0.010	3.28E-01	1.00E+00	0.001	4.53	0.1
PCaa C40:4	809	0.252	0.174	1.48E-01	1.00E+00	0.002	0.44	0.8
PCaa C40:5	824	0.071	0.071	3.15E-01	1.00E+00	0.001	2.82	0.24
PCaa C40:6	1007	0.044	0.027	1.06E-01	1.00E+00	0.002	7.07	0.029

PCae C34:1	1008	-0.289	0.076	1.45E-04	1.67E-02		0.009	3.01	0.22
PCae C34:2	941	-0.154	0.058	8.37E-03	9.63E-01		0.005	6.07	0.048
PCae C34:3	1008	-0.238	0.065	2.81E-04	3.23E-02		0.008	11.9	0.003
PCae C36:1	892	-0.187	0.056	9.61E-04	1.11E-01		0.008	0.924	0.63
PCae C36:2	808	-0.328	0.067	9.85E-07	1.13E-04		0.018	1.62	0.45
PCae C36:3	1008	-0.198	0.079	1.22E-02	1.00E+00		0.004	3.89	0.14
PCae C36:4	940	-0.007	0.036	8.37E-01	1.00E+00		0.000	5.31	0.07
PCae C36:5	1008	-0.010	0.054	8.47E-01	1.00E+00		0.000	4.11	0.13
PCae C38	834	-0.380	0.255	1.36E-01	1.00E+00		0.002	5.06	0.08
PCae C38:3	836	-0.158	0.069	2.34E-02	1.00E+00		0.004	0.395	0.82
PCae C38:4	824	-0.139	0.058	1.59E-02	1.00E+00		0.005	5.36	0.07
PCae C38:5	941	-0.041	0.038	2.77E-01	1.00E+00		0.001	2.94	0.23
PCae C38:6	892	0.016	0.088	8.57E-01	1.00E+00		0.000	1.73	0.42
PCae C40	790	-0.137	0.093	1.41E-01	1.00E+00		0.002	1.12	0.57
PCae C40:4	796	-0.688	0.200	6.36E-04	7.31E-02		0.009	0.434	0.8

PCae C40:6	825	-0.586	0.185	1.61E-03	1.85E-01	0.007	1.46	0.48
SM C32:2	748	9.746	1.017	1.39E-20	1.60E-18	0.081	16.2	<0.0001
SM C35:1	958	-0.017	0.193	9.30E-01	1.00E+00	0.000	0.055	0.97
SM C36:1	1008	0.050	0.030	9.77E-02	1.00E+00	0.002	1.99	0.37
SM C36:2	875	0.196	0.062	1.50E-03	1.72E-01	0.007	7.67	0.022

SM C41:1	892	-0.080	0.068	2.36E-01	1.00E+00	0.001	0.494	0.78
SM C41:2	891	0.091	0.094	3.37E-01	1.00E+00	0.001	0.136	0.93
SM C42:1	840	-0.042	0.039	2.90E-01	1.00E+00	0.001	0.606	0.74
SM C42:2	826	0.019	0.020	3.47E-01	1.00E+00	0.001	0.105	0.95
NEFA 12:0	943	-0.239	0.047	3.80E-07	4.38E-05	0.017	2.8	0.25
NEFA 14:0	990	-0.083	0.026	1.22E-03	1.41E-01	0.007	0.564	0.75
NEFA 14:1	987	-0.179	0.150	2.33E-01	1.00E+00	0.001	5.39	0.07
NEFA 16:0	991	-0.009	0.003	1.35E-02	1.00E+00	0.004	2.54	0.28
NEFA 16:1	961	-0.024	0.014	8.60E-02	1.00E+00	0.002	5.96	0.051
NEFA 16:2	961	0.790	0.944	4.02E-01	1.00E+00	0.001	2.58	0.28
NEFA 17:0	972	-0.350	0.127	6.14E-03	7.06E-01	0.005	0.274	0.87
NEFA 17:1	960	-0.251	0.198	2.05E-01	1.00E+00	0.001	1.22	0.54
NEFA 18:1	992	-0.007	0.002	6.12E-03	7.04E-01	0.005	0.949	0.62
NEFA 18:2	989	-0.019	0.008	2.59E-02	1.00E+00	0.003	2.22	0.33
NEFA 18:3	991	-0.068	0.036	5.97E-02	1.00E+00	0.002	1.19	0.55
NEFA 19:1	979	-0.824	0.407	4.33E-02	1.00E+00	0.003	0.753	0.69
NEFA 20:2	891	-0.386	0.265	1.46E-01	1.00E+00	0.002	1.72	0.42
NEFA 20:3	853	0.420	0.196	3.23E-02	1.00E+00	0.004	0.976	0.61
NEFA 20:4	963	0.101	0.057	7.56E-02	1.00E+00	0.002	0.645	0.72

NEFA 20:5	911	0.011	0.446	9.80E-01	1.00E+00	0.000	0.106	0.95
NEFA 22:4	963	0.870	0.568	1.26E-01	1.00E+00	0.002	1	0.61
NEFA 22:5	992	-0.098	0.299	7.42E-01	1.00E+00	0.000	0.071	0.97
NEFA 22:6	992	-0.062	0.137	6.54E-01	1.00E+00	0.000	0.14	0.93
NEFA 24:0	944	-8.122	2.183	2.11E-04	2.43E-02	0.009	6.53	0.038
NEFA 24:4	839	24.212	11.706	3.89E-02	1.00E+00	0.003	3.41	0.18
NEFA 26:1	946	3.643	3.937	3.55E-01	1.00E+00	0.001	3.91	0.14

Supplemental Table 4 Results of the adjusted linear models and individual participant meta-analysis of the association of metabolites with height. Statistical significance was evaluated using Bonferroni corrected p-values (p.adjust) < 0.05 (with dark grey background). AA, amino acids; Carn, Acylcarnitines; lyso.PC, lyso-phosphatidylcholine; NEFA, nonesterified fatty acid; PCaa, Diacyl-phosphatidylcholine; PCae, Alkyl-acyl-phosphatidylcholine; SM,

CHOP 5.5 years

Metabolite	N	Estimate	Std..Error	P	P.adjusted	PartialRsquared
Ala	387	-0.002	0.003	5.54E-01	1.00E+00	0.001
Arg	385	0.016	0.012	1.84E-01	1.00E+00	0.005
Asn	387	-0.014	0.021	5.02E-01	1.00E+00	0.001
Asp	385	0.089	0.067	1.84E-01	1.00E+00	0.005
Cit	387	-0.039	0.028	1.74E-01	1.00E+00	0.005
Gln	387	-0.001	0.002	4.08E-01	1.00E+00	0.002
Glu	387	0.000	0.003	8.70E-01	1.00E+00	0.000
Gly	387	0.000	0.005	9.54E-01	1.00E+00	0.000
His	387	-0.010	0.011	3.56E-01	1.00E+00	0.002
Ile	387	0.008	0.013	5.46E-01	1.00E+00	0.001
Leu	387	0.005	0.008	5.11E-01	1.00E+00	0.001
Lys	387	0.001	0.007	9.33E-01	1.00E+00	0.000
Met	387	-0.022	0.047	6.38E-01	1.00E+00	0.001
Orn	387	0.002	0.011	8.91E-01	1.00E+00	0.000
Phe	387	0.007	0.020	7.23E-01	1.00E+00	0.000
Pro	387	0.000	0.004	9.90E-01	1.00E+00	0.000
Ser	386	0.006	0.010	5.49E-01	1.00E+00	0.001
Thr	387	0.012	0.007	1.07E-01	1.00E+00	0.007
Trp	386	0.006	0.020	7.72E-01	1.00E+00	0.000
Tyr	387	-0.006	0.014	6.40E-01	1.00E+00	0.001
Val	387	0.003	0.004	5.17E-01	1.00E+00	0.001
H1	386	0.000	0.000	9.58E-01	1.00E+00	0.000
Carn	392	0.032	0.024	1.89E-01	1.00E+00	0.004
Carn C2	392	-0.075	0.062	2.28E-01	1.00E+00	0.004
Carn C3	392	2.123	2.311	3.59E-01	1.00E+00	0.002
Carn C3:1	274	46.130	39.952	2.49E-01	1.00E+00	0.005
Carn C4	392	-2.419	2.095	2.49E-01	1.00E+00	0.003
Carn C4:0:OH	274	-1.037	2.878	7.19E-01	1.00E+00	0.000
Carn C4:1	350	9.212	7.120	1.97E-01	1.00E+00	0.005
Carn C5	392	11.483	6.043	5.82E-02	1.00E+00	0.009
Carn C5:0:OH	315	897.784	490.229	6.80E-02	1.00E+00	0.011
Carn C6	316	-5.863	7.667	4.45E-01	1.00E+00	0.002
Carn C6:1	206	87.598	71.871	2.24E-01	1.00E+00	0.007
Carn C8	324	0.961	1.520	5.27E-01	1.00E+00	0.001
Carn C8:1	392	5.937	4.115	1.50E-01	1.00E+00	0.005
Carn C9	392	28.770	15.517	6.45E-02	1.00E+00	0.009
Carn C10	391	1.226	0.970	2.07E-01	1.00E+00	0.004

Carn C10:1	392	1.487	1.300	2.54E-01	1.00E+00	0.003
Carn C10:2	392	4.317	11.733	7.13E-01	1.00E+00	0.000
Carn C12::DC	261	4.383	3.158	1.66E-01	1.00E+00	0.007
Carn C12	391	0.090	2.659	9.73E-01	1.00E+00	0.000
Carn C12:1	392	0.982	0.973	3.13E-01	1.00E+00	0.003
Carn C14	392	8.124	7.472	2.78E-01	1.00E+00	0.003
Carn C14:1	390	0.811	1.902	6.70E-01	1.00E+00	0.000
Carn C14:2	392	-0.296	8.614	9.73E-01	1.00E+00	0.000
Carn C14:2:OH	249	-7.705	25.469	7.63E-01	1.00E+00	0.000
Carn C15	392	-8.737	20.971	6.77E-01	1.00E+00	0.000
Carn C16	391	-0.340	8.052	9.66E-01	1.00E+00	0.000
Carn C16:OH	391	5.077	8.723	5.61E-01	1.00E+00	0.001
Carn C16:1	289	-36.170	48.745	4.59E-01	1.00E+00	0.002
Carn C16:2	274	-27.012	29.776	3.65E-01	1.00E+00	0.003
Carn C16:2:OH	254	-54.281	36.404	1.37E-01	1.00E+00	0.009
Carn C18	392	-31.811	15.613	4.23E-02	1.00E+00	0.011
Carn C18:1	392	-2.611	5.069	6.07E-01	1.00E+00	0.001
Carn C18:1:OH	323	16.288	33.776	6.30E-01	1.00E+00	0.001
Carn C18:2	392	-3.248	7.075	6.46E-01	1.00E+00	0.001
Carn C18:2:OH	248	-20.607	97.224	8.32E-01	1.00E+00	0.000
Carn C20	390	-48.710	50.290	3.33E-01	1.00E+00	0.002
Carn C20:1	314	-1667.610	1309.132	2.04E-01	1.00E+00	0.005
Carn C20:3	392	-34.104	33.118	3.04E-01	1.00E+00	0.003
Carn C20:4	392	-309.639	375.469	4.10E-01	1.00E+00	0.002
Carn C22	290	-497.250	557.662	3.73E-01	1.00E+00	0.003
Carn C22:5	275	-88.756	80.262	2.70E-01	1.00E+00	0.004
Carn C22:6	253	-548.099	318.603	8.66E-02	1.00E+00	0.012
LPC C14	246	0.719	0.366	5.06E-02	1.00E+00	0.016
LPC C15	246	0.044	0.740	9.53E-01	1.00E+00	0.000
LPC C16	391	0.009	0.008	2.47E-01	1.00E+00	0.003
LPC C16:1	392	0.577	0.219	8.85E-03	1.00E+00	0.017
LPC C17	390	0.111	0.339	7.43E-01	1.00E+00	0.000
LPC C18	392	0.006	0.020	7.48E-01	1.00E+00	0.000
LPC C18:1	392	0.020	0.043	6.37E-01	1.00E+00	0.001
LPC C18:2	392	0.007	0.022	7.42E-01	1.00E+00	0.000
LPC C18:3	351	0.322	0.760	6.72E-01	1.00E+00	0.001
LPC C20:1	203	-0.669	1.229	5.86E-01	1.00E+00	0.001
LPC C20:3	390	0.375	0.227	9.99E-02	1.00E+00	0.007
LPC C20:4	392	0.211	0.103	4.06E-02	1.00E+00	0.011
LPC C20:5	321	1.186	1.044	2.57E-01	1.00E+00	0.004

LPC C22:6	316	0.211	0.333	5.27E-01	1.00E+00	0.001
PCaa C20:4	213	-7.219	5.223	1.68E-01	1.00E+00	0.009
PCaa C28:2	321	-1.146	4.952	8.17E-01	1.00E+00	0.000
PCaa C30	392	0.077	0.140	5.83E-01	1.00E+00	0.001
PCaa C30:2	391	-1.191	0.958	2.15E-01	1.00E+00	0.004
PCaa C30:3	391	6.058	3.045	4.73E-02	1.00E+00	0.010
PCaa C30:4	168	-1.804	9.747	8.53E-01	1.00E+00	0.000
PCaa C32	392	0.033	0.060	5.85E-01	1.00E+00	0.001
PCaa C32:1	392	0.028	0.042	5.06E-01	1.00E+00	0.001
PCaa C32:2	392	0.030	0.214	8.87E-01	1.00E+00	0.000
PCaa C34:1	392	-0.006	0.004	1.72E-01	1.00E+00	0.005
PCaa C34:2	392	-0.005	0.003	6.51E-02	1.00E+00	0.009
PCaa C34:3	390	0.050	0.048	2.91E-01	1.00E+00	0.003
PCaa C34:4	392	0.764	0.377	4.36E-02	1.00E+00	0.010
PCaa C36	273	0.225	0.106	3.50E-02	1.00E+00	0.016
PCaa C36:1	392	-0.023	0.015	1.36E-01	1.00E+00	0.006
PCaa C36:2	392	-0.008	0.004	3.40E-02	1.00E+00	0.011
PCaa C36:3	392	-0.005	0.008	5.18E-01	1.00E+00	0.001
PCaa C36:4	392	-0.004	0.006	4.67E-01	1.00E+00	0.001
PCaa C36:5	390	0.108	0.061	7.99E-02	1.00E+00	0.008

PCaa C36:6	322	0.270	0.326	4.08E-01	1.00E+00	0.002
PCaa C38	314	-0.050	0.200	8.03E-01	1.00E+00	0.000
PCaa C38:3	392	0.001	0.020	9.53E-01	1.00E+00	0.000
PCaa C38:4	392	0.003	0.008	7.05E-01	1.00E+00	0.000
PCaa C38:5	392	0.036	0.022	1.00E-01	1.00E+00	0.007
PCaa C38:6	392	0.002	0.013	8.62E-01	1.00E+00	0.000
PCaa C40	281	-0.179	0.387	6.44E-01	1.00E+00	0.001
PCaa C40:4	391	-0.071	0.185	7.01E-01	1.00E+00	0.000
PCaa C40:5	392	0.018	0.066	7.83E-01	1.00E+00	0.000
PCaa C40:6	392	0.001	0.030	9.76E-01	1.00E+00	0.000
PCaa C42	246	-0.285	0.728	6.96E-01	1.00E+00	0.001
PCaa C42:1	209	2.395	1.502	1.12E-01	1.00E+00	0.012
PCaa C42:6	203	-0.556	0.898	5.37E-01	1.00E+00	0.002
PCaa C43:6	249	-0.283	0.528	5.92E-01	1.00E+00	0.001
PCaa C44:12	392	-0.381	0.385	3.23E-01	1.00E+00	0.003
PCae C30:2	281	-2.025	2.133	3.43E-01	1.00E+00	0.003
PCae C32	392	-0.004	0.281	9.89E-01	1.00E+00	0.000

PCae C32:1	392	-0.127	0.229	5.79E-01	1.00E+00	0.001
PCae C32:2	206	0.485	0.843	5.66E-01	1.00E+00	0.002
PCae C34	392	0.048	0.388	9.02E-01	1.00E+00	0.000
PCae C34:1	392	0.004	0.083	9.62E-01	1.00E+00	0.000
PCae C34:2	392	-0.083	0.071	2.43E-01	1.00E+00	0.004
PCae C34:3	392	-0.071	0.082	3.92E-01	1.00E+00	0.002
PCae C34:4	246	1.022	1.990	6.08E-01	1.00E+00	0.001
PCae C36	254	-0.704	0.425	9.87E-02	1.00E+00	0.011
PCae C36:1	391	0.035	0.038	3.55E-01	1.00E+00	0.002
PCae C36:2	391	-0.054	0.047	2.50E-01	1.00E+00	0.003
PCae C36:3	392	-0.045	0.083	5.92E-01	1.00E+00	0.001
PCae C36:4	391	-0.011	0.043	7.97E-01	1.00E+00	0.000
PCae C36:5	392	-0.019	0.055	7.34E-01	1.00E+00	0.000
PCae C36:6	179	-0.628	0.794	4.31E-01	1.00E+00	0.003
PCae C38	315	0.343	0.168	4.21E-02	1.00E+00	0.013
PCae C38:2	313	-0.004	0.057	9.47E-01	1.00E+00	0.000
PCae C38:3	390	0.050	0.052	3.37E-01	1.00E+00	0.002
PCae C38:4	392	-0.087	0.056	1.17E-01	1.00E+00	0.006
PCae C38:5	392	-0.038	0.044	3.90E-01	1.00E+00	0.002
PCae C38:6	392	-0.047	0.095	6.20E-01	1.00E+00	0.001
PCae C40	392	0.069	0.075	3.55E-01	1.00E+00	0.002
PCae C40:1	237	0.200	0.303	5.09E-01	1.00E+00	0.002
PCae C40:3	321	0.010	0.070	8.92E-01	1.00E+00	0.000
PCae C40:4	352	0.189	0.121	1.18E-01	1.00E+00	0.007

PCae C40:5	392	0.080	0.127	5.30E-01	1.00E+00	0.001
PCae C40:6	392	-0.215	0.161	1.84E-01	1.00E+00	0.005
PCae C42:2	324	0.322	0.413	4.36E-01	1.00E+00	0.002
PCae C42:3	281	-0.523	0.330	1.15E-01	1.00E+00	0.009
PCae C42:4	198	0.651	0.764	3.95E-01	1.00E+00	0.004
PCae C42:5	316	0.393	0.266	1.40E-01	1.00E+00	0.007
PCae C42:6	391	0.160	0.427	7.09E-01	1.00E+00	0.000
SM C21:2	352	9.206	9.640	3.40E-01	1.00E+00	0.003
SM C32:1	392	0.086	0.096	3.70E-01	1.00E+00	0.002
SM C32:2	352	2.757	1.286	3.27E-02	1.00E+00	0.013
SM C33:1	392	0.224	0.169	1.85E-01	1.00E+00	0.004
SM C33:3	186	-5.439	4.433	2.21E-01	1.00E+00	0.008
SM C34:1	391	-0.012	0.010	2.11E-01	1.00E+00	0.004
SM C34:2	391	0.012	0.069	8.65E-01	1.00E+00	0.000
SM C34:4	215	-20.664	15.693	1.89E-01	1.00E+00	0.008
SM C35:1	391	0.310	0.284	2.76E-01	1.00E+00	0.003
SM C35:2	204	-2.095	2.183	3.38E-01	1.00E+00	0.005
SM C36:1	390	-0.018	0.048	7.17E-01	1.00E+00	0.000
SM C36:2	391	0.098	0.090	2.80E-01	1.00E+00	0.003
SM C36:3	185	1.717	0.928	6.59E-02	1.00E+00	0.018
SM C37:1	204	0.242	0.258	3.51E-01	1.00E+00	0.004
SM C37:3	213	-0.086	1.912	9.64E-01	1.00E+00	0.000
SM C38:2	392	-0.099	0.058	8.89E-02	1.00E+00	0.007
SM C39:1	316	0.268	0.145	6.60E-02	1.00E+00	0.011
SM C40:2	244	-0.015	0.035	6.68E-01	1.00E+00	0.001

SM C40:4	314	0.491	0.400	2.21E-01	1.00E+00	0.005
SM C40:5	352	1.485	0.713	3.81E-02	1.00E+00	0.012
SM C41:1	391	-0.071	0.052	1.68E-01	1.00E+00	0.005
SM C41:2	391	-0.004	0.075	9.58E-01	1.00E+00	0.000
SM C42:1	392	-0.076	0.035	3.04E-02	1.00E+00	0.012
SM C42:2	391	-0.005	0.021	8.04E-01	1.00E+00	0.000
SM C42:3	322	0.048	0.041	2.44E-01	1.00E+00	0.004
SM C42:6	392	-0.063	0.253	8.04E-01	1.00E+00	0.000
SM C43	177	0.230	1.368	8.67E-01	1.00E+00	0.000
SM C43:1	206	-0.040	0.560	9.44E-01	1.00E+00	0.000
SM C43:2	321	-0.120	0.280	6.68E-01	1.00E+00	0.001
SM C44:6	238	1.082	0.690	1.18E-01	1.00E+00	0.010
SM C47:6	324	-85.129	41.660	4.18E-02	1.00E+00	0.013
NEFA 12:0	178	-0.090	0.080	2.63E-01	1.00E+00	0.007
NEFA 14:0	322	-0.032	0.028	2.54E-01	1.00E+00	0.004
NEFA 14:1	389	-0.051	0.043	2.42E-01	1.00E+00	0.004
NEFA 15:0	178	-0.433	0.334	1.96E-01	1.00E+00	0.010
NEFA 16:0	245	-0.006	0.004	1.48E-01	1.00E+00	0.009
NEFA 16:1	389	-0.011	0.013	3.78E-01	1.00E+00	0.002
NEFA 16:2	311	0.254	0.476	5.94E-01	1.00E+00	0.001
NEFA 17:0	168	0.064	0.153	6.77E-01	1.00E+00	0.001
NEFA 17:1	389	-0.133	0.114	2.44E-01	1.00E+00	0.004
NEFA 17:2	168	-0.373	5.521	9.46E-01	1.00E+00	0.000
NEFA 18:1	389	-0.004	0.002	8.80E-02	1.00E+00	0.008
NEFA 18:2	389	-0.010	0.005	3.85E-02	1.00E+00	0.011
NEFA 18:3	389	-0.064	0.067	3.40E-01	1.00E+00	0.002
NEFA 18:4	168	-2.919	3.177	3.60E-01	1.00E+00	0.005
NEFA 19:1	309	-0.523	0.607	3.89E-01	1.00E+00	0.002
NEFA 20:1	389	-0.241	0.143	9.24E-02	1.00E+00	0.007
NEFA 20:2	389	-0.137	0.078	7.75E-02	1.00E+00	0.008

NEFA 20:3	389	-0.079	0.154	6.07E-01	1.00E+00	0.001
NEFA 20:4	388	0.013	0.052	8.03E-01	1.00E+00	0.000
NEFA 20:5	308	-0.003	0.311	9.93E-01	1.00E+00	0.000
NEFA 22:3	235	-10.081	9.205	2.75E-01	1.00E+00	0.005
NEFA 22:4	389	-0.017	0.660	9.80E-01	1.00E+00	0.000
NEFA 22:5	386	-0.161	0.338	6.34E-01	1.00E+00	0.001
NEFA 22:6	389	-0.096	0.134	4.76E-01	1.00E+00	0.001
NEFA 24:0	168	-4.064	2.346	8.50E-02	1.00E+00	0.018
NEFA 24:1	389	-1.355	0.923	1.43E-01	1.00E+00	0.006
NEFA 24:2	168	27.950	19.076	1.45E-01	1.00E+00	0.013
NEFA 24:3	243	-8.633	13.576	5.25E-01	1.00E+00	0.002
NEFA 24:4	389	-5.967	11.503	6.04E-01	1.00E+00	0.001
NEFA 24:6	167	-18.830	32.931	5.68E-01	1.00E+00	0.002
NEFA 26:0	336	-0.037	1.433	9.79E-01	1.00E+00	0.000
NEFA 26:1	313	-2.305	1.540	1.36E-01	1.00E+00	0.007
NEFA 26:2	389	-2.632	1.921	1.71E-01	1.00E+00	0.005
NEFA 26:3	310	-3.122	6.979	6.55E-01	1.00E+00	0.001
NEFA 26:4	389	-49.470	24.708	4.60E-02	1.00E+00	0.010
Pyruvate	384	-0.001	0.001	2.44E-01	1.00E+00	0.004
Lactic_acid	389	0.000	0.000	1.78E-01	1.00E+00	0.005
Fumarate	382	0.891	0.768	2.47E-01	1.00E+00	0.004
3-Methyl-2-oxobutanoate	384	-0.009	0.016	5.97E-01	1.00E+00	0.001
Succininate	387	0.158	0.069	2.22E-02	1.00E+00	0.014
Methylmalonate	389	5.382	3.228	9.63E-02	1.00E+00	0.007
Taurine	387	0.005	0.006	3.95E-01	1.00E+00	0.002
3-Methyl-2-oxovalerate	388	0.020	0.018	2.77E-01	1.00E+00	0.003
4-Methyl-2-oxovalerate	383	0.010	0.009	2.69E-01	1.00E+00	0.003
Malate	385	0.263	0.131	4.49E-02	1.00E+00	0.010
Alpha-ketoglutarate	378	-0.072	0.041	8.09E-02	1.00E+00	0.008
Alpha-aminoadipate	387	0.974	1.430	4.96E-01	1.00E+00	0.001
Isocitrate	389	0.082	0.041	4.75E-02	1.00E+00	0.010
Citrate	389	0.007	0.003	2.91E-02	1.00E+00	0.012
Beta-hydroxybutyrate	150	0.029	0.009	7.68E-04	1.79E-01	0.074

Sum AA	384	0.000	0.001	9.87E-01	1.00E+00	0.000
Sum Carn	389	0.019	0.022	4.04E-01	1.00E+00	0.002
Sum LPC	206	-0.001	0.007	9.33E-01	1.00E+00	0.000
Sum PCaa	311	-0.002	0.001	9.72E-02	1.00E+00	0.009
Sum PCae	274	0.003	0.008	7.33E-01	1.00E+00	0.000
Sum SM	350	-0.005	0.009	5.73E-01	1.00E+00	0.001
Sum NEFA	99	-0.002	0.001	1.07E-01	1.00E+00	0.027

CHOP 8 years

Metabolite	N	Estimate	Std..Error	P	P.adjusted	PartialRsquared
Ala	354	0.005	0.004	2.88E-01	1.00E+00	0.003
Arg	354	-0.003	0.020	8.88E-01	1.00E+00	0.000
Asn	354	-0.012	0.032	7.03E-01	1.00E+00	0.000
Asp	354	0.052	0.100	6.02E-01	1.00E+00	0.001
Cit	354	-0.073	0.044	1.02E-01	1.00E+00	0.008
Gln	353	-0.003	0.002	2.06E-01	1.00E+00	0.005
Glu	354	0.003	0.003	3.05E-01	1.00E+00	0.003
Gly	354	0.001	0.007	9.37E-01	1.00E+00	0.000
His	354	0.009	0.012	4.46E-01	1.00E+00	0.002
Ile	354	0.066	0.030	3.13E-02	1.00E+00	0.013
Leu	354	0.043	0.017	1.41E-02	1.00E+00	0.017
Lys	354	0.043	0.020	3.31E-02	1.00E+00	0.013
Met	354	0.032	0.072	6.61E-01	1.00E+00	0.001
Orn	354	0.043	0.021	3.98E-02	1.00E+00	0.012
Phe	353	0.026	0.035	4.51E-01	1.00E+00	0.002
Pro	354	0.005	0.008	5.40E-01	1.00E+00	0.001
Ser	353	-0.011	0.010	2.88E-01	1.00E+00	0.003
Thr	354	0.020	0.013	1.43E-01	1.00E+00	0.006
Trp	354	0.023	0.032	4.79E-01	1.00E+00	0.001
Tyr	353	0.063	0.025	1.21E-02	1.00E+00	0.018
Val	354	0.017	0.008	4.10E-02	1.00E+00	0.012
H1	354	0.000	0.001	5.04E-01	1.00E+00	0.001
Carn	354	0.037	0.015	1.26E-02	1.00E+00	0.018
Carn C2	354	-0.009	0.119	9.42E-01	1.00E+00	0.000
Carn C3	351	-0.119	0.978	9.03E-01	1.00E+00	0.000
Carn C3:1	286	-1.529	15.066	9.19E-01	1.00E+00	0.000
Carn C4:0:OH	237	-0.941	4.107	8.19E-01	1.00E+00	0.000
Carn C5	354	183.683	220.491	4.05E-01	1.00E+00	0.002
Carn C5:0:OH	354	3.008	3.989	4.51E-01	1.00E+00	0.002
Carn C6	354	0.485	6.399	9.40E-01	1.00E+00	0.000
Carn C6:1	354	-5.002	8.217	5.43E-01	1.00E+00	0.001
Carn C8	354	-0.629	1.822	7.30E-01	1.00E+00	0.000
Carn C8:1	354	3.103	4.375	4.79E-01	1.00E+00	0.001
Carn C9	354	0.041	17.013	9.98E-01	1.00E+00	0.000
Carn C10	354	-0.649	1.075	5.47E-01	1.00E+00	0.001

Carn C10:1	353	-1.835	1.777	3.03E-01	1.00E+00	0.003
Carn C10:2	354	11.028	12.540	3.80E-01	1.00E+00	0.002
Carn C12	354	0.218	2.738	9.36E-01	1.00E+00	0.000
Carn C12:1	354	1.007	1.145	3.80E-01	1.00E+00	0.002
Carn C14	354	6.715	11.495	5.59E-01	1.00E+00	0.001
Carn C14:1	353	0.722	1.879	7.01E-01	1.00E+00	0.000
Carn C14:2	354	-5.925	8.776	5.00E-01	1.00E+00	0.001
Carn C14:2:OH	352	2.246	27.114	9.34E-01	1.00E+00	0.000
Carn C15	354	109.690	62.151	7.85E-02	1.00E+00	0.009
Carn C16	354	8.108	14.045	5.64E-01	1.00E+00	0.001
Carn C16:0:OH	288	285.688	169.458	9.29E-02	1.00E+00	0.010
Carn C16:1	354	5.725	12.147	6.38E-01	1.00E+00	0.001
Carn C16:2	354	-32.074	31.497	3.09E-01	1.00E+00	0.003
Carn C16:2:OH	304	33.543	62.138	5.90E-01	1.00E+00	0.001
Carn C18	354	-22.213	30.899	4.73E-01	1.00E+00	0.001
Carn C18:1	354	0.270	9.265	9.77E-01	1.00E+00	0.000
Carn C18:1:OH	354	-19.157	41.950	6.48E-01	1.00E+00	0.001
Carn C18:2	352	-14.385	15.109	3.42E-01	1.00E+00	0.003
Carn C18:2:OH	233	0.255	110.183	9.98E-01	1.00E+00	0.000
Carn C20	348	-30.828	60.418	6.10E-01	1.00E+00	0.001
Carn C20:1	353	927.262	4279.390	8.29E-01	1.00E+00	0.000
Carn C20:3	354	303.913	162.251	6.19E-02	1.00E+00	0.010
Carn C20:4	304	1690.141	2134.693	4.29E-01	1.00E+00	0.002
Carn C22	221	-3715.298	1988.959	6.31E-02	1.00E+00	0.016
Carn C22:5	253	642.108	314.527	4.23E-02	1.00E+00	0.016
Carn C22:6	183	356.637	223.543	1.12E-01	1.00E+00	0.014
LPC C16	354	-0.006	0.007	3.63E-01	1.00E+00	0.002
LPC C16:1	354	0.132	0.248	5.96E-01	1.00E+00	0.001
LPC C17	288	-0.107	0.392	7.85E-01	1.00E+00	0.000
LPC C18	354	-0.025	0.019	1.95E-01	1.00E+00	0.005
LPC C18:1	354	-0.077	0.044	7.99E-02	1.00E+00	0.009
LPC C18:2	354	-0.053	0.024	2.71E-02	1.00E+00	0.014
LPC C18:3	221	0.330	1.367	8.10E-01	1.00E+00	0.000
LPC C18:6	180	1.027	1.990	6.07E-01	1.00E+00	0.002
LPC C20:3	287	0.037	0.359	9.17E-01	1.00E+00	0.000
LPC C20:4	354	-0.041	0.124	7.43E-01	1.00E+00	0.000
LPC C20:5	170	1.405	1.380	3.10E-01	1.00E+00	0.006

LPC C22:5	154	1.018	1.736	5.59E-01	1.00E+00	0.002
LPC C22:6	252	0.208	0.524	6.92E-01	1.00E+00	0.001
PCaa C30:5	179	306.225	147.070	3.88E-02	1.00E+00	0.024
PCaa C32	354	-0.105	0.083	2.09E-01	1.00E+00	0.004
PCaa C32:1	288	0.087	0.054	1.05E-01	1.00E+00	0.009
PCaa C34:1	354	-0.001	0.005	8.03E-01	1.00E+00	0.000
PCaa C34:2	289	-0.005	0.004	1.78E-01	1.00E+00	0.006
PCaa C34:3	354	0.040	0.057	4.83E-01	1.00E+00	0.001
PCaa C34:4	304	0.426	0.464	3.59E-01	1.00E+00	0.003
PCaa C36:2	354	-0.007	0.004	6.28E-02	1.00E+00	0.010
PCaa C36:3	354	-0.010	0.011	3.73E-01	1.00E+00	0.002
PCaa C36:4	289	0.002	0.009	8.65E-01	1.00E+00	0.000
PCaa C36:5	285	0.038	0.057	5.07E-01	1.00E+00	0.002

PCaa C38	165	-0.400	0.253	1.16E-01	1.00E+00	0.015
PCaa C38:3	167	-0.020	0.041	6.24E-01	1.00E+00	0.001
PCaa C38:4	183	0.006	0.013	6.57E-01	1.00E+00	0.001
PCaa C38:5	238	0.001	0.036	9.77E-01	1.00E+00	0.000
PCaa C38:6	354	-0.002	0.014	8.65E-01	1.00E+00	0.000
PCaa C40:4	156	-0.076	0.398	8.49E-01	1.00E+00	0.000
PCaa C40:5	171	-0.138	0.136	3.12E-01	1.00E+00	0.006
PCaa C40:6	353	0.004	0.034	9.07E-01	1.00E+00	0.000
PCaa C44:12	173	-0.634	1.456	6.64E-01	1.00E+00	0.001

PCae C34:1	354	-0.109	0.097	2.60E-01	1.00E+00	0.004
PCae C34:2	287	-0.088	0.076	2.52E-01	1.00E+00	0.005
PCae C34:3	354	-0.116	0.086	1.77E-01	1.00E+00	0.005
PCae C36:1	239	0.023	0.061	7.01E-01	1.00E+00	0.001
PCae C36:2	155	0.003	0.118	9.80E-01	1.00E+00	0.000
PCae C36:3	354	-0.103	0.092	2.64E-01	1.00E+00	0.004
PCae C36:4	287	-0.037	0.047	4.36E-01	1.00E+00	0.002
PCae C36:5	354	0.013	0.074	8.59E-01	1.00E+00	0.000
PCae C38	182	-0.061	0.357	8.65E-01	1.00E+00	0.000
PCae C38:3	182	-0.078	0.078	3.13E-01	1.00E+00	0.006
PCae C38:4	173	-0.031	0.137	8.22E-01	1.00E+00	0.000
PCae C38:5	288	-0.001	0.054	9.81E-01	1.00E+00	0.000
PCae C38:6	238	0.099	0.142	4.86E-01	1.00E+00	0.002
PCae C40	165	-0.082	0.141	5.60E-01	1.00E+00	0.002
PCae C40:4	171	0.005	0.269	9.85E-01	1.00E+00	0.000

PCae C40:6	171	-0.325	0.314	3.03E-01	1.00E+00	0.006
SM C21:2	181	-6.281	7.222	3.86E-01	1.00E+00	0.004
SM C31:1	181	12.846	4.698	6.89E-03	1.00E+00	0.040
SM C32:1	354	0.220	0.134	1.01E-01	1.00E+00	0.008
SM C32:2	171	1.394	1.631	3.94E-01	1.00E+00	0.004
SM C33:1	354	0.083	0.185	6.52E-01	1.00E+00	0.001
SM C34:1	354	-0.020	0.013	1.26E-01	1.00E+00	0.007
SM C34:2	354	0.209	0.085	1.47E-02	1.00E+00	0.017
SM C35:1	304	0.215	0.351	5.41E-01	1.00E+00	0.001
SM C36:1	354	0.097	0.049	4.63E-02	1.00E+00	0.011
SM C36:2	221	0.264	0.148	7.55E-02	1.00E+00	0.014
SM C36:3	287	1.859	0.992	6.19E-02	1.00E+00	0.012

SM C41:1	238	-0.086	0.103	4.03E-01	1.00E+00	0.003
SM C41:2	237	0.210	0.130	1.07E-01	1.00E+00	0.011
SM C42:1	188	-0.053	0.067	4.30E-01	1.00E+00	0.003
SM C42:2	173	0.063	0.035	7.88E-02	1.00E+00	0.018
NEFA 10:0	338	-0.044	0.119	7.11E-01	1.00E+00	0.000
NEFA 11:0	265	-0.289	3.601	9.36E-01	1.00E+00	0.000
NEFA 12:0	337	-0.313	0.163	5.56E-02	1.00E+00	0.011
NEFA 12:1	220	2.508	4.209	5.52E-01	1.00E+00	0.002
NEFA 14:0	338	-0.020	0.036	5.83E-01	1.00E+00	0.001
NEFA 14:1	337	0.125	0.194	5.19E-01	1.00E+00	0.001
NEFA 15:0	338	0.012	0.170	9.43E-01	1.00E+00	0.000
NEFA 15:1	215	-1.517	2.654	5.68E-01	1.00E+00	0.002
NEFA 16:0	338	-0.001	0.005	7.88E-01	1.00E+00	0.000
NEFA 16:1	338	0.006	0.020	7.71E-01	1.00E+00	0.000
NEFA 16:2	338	0.645	1.380	6.41E-01	1.00E+00	0.001
NEFA 17:0	338	-0.055	0.170	7.48E-01	1.00E+00	0.000
NEFA 17:1	338	0.132	0.284	6.42E-01	1.00E+00	0.001
NEFA 18:0	283	-0.013	0.011	2.29E-01	1.00E+00	0.005
NEFA 18:1	338	-0.003	0.003	3.72E-01	1.00E+00	0.002
NEFA 18:2	336	-0.012	0.011	2.84E-01	1.00E+00	0.003
NEFA 18:3	338	0.012	0.047	7.94E-01	1.00E+00	0.000
NEFA 18:4	336	0.229	3.068	9.41E-01	1.00E+00	0.000
NEFA 19:0	283	-0.648	1.852	7.27E-01	1.00E+00	0.000
NEFA 19:1	338	0.289	0.591	6.25E-01	1.00E+00	0.001
NEFA 20:0	338	-1.479	0.826	7.43E-02	1.00E+00	0.009
NEFA 20:1	338	-0.134	0.201	5.04E-01	1.00E+00	0.001
NEFA 20:2	337	-0.012	0.344	9.73E-01	1.00E+00	0.000

NEFA 20:3	338	0.108	0.212	6.09E-01	1.00E+00	0.001
NEFA 20:4	338	0.027	0.055	6.28E-01	1.00E+00	0.001
NEFA 20:5	336	0.313	0.559	5.75E-01	1.00E+00	0.001
NEFA 22:0	287	-1.775	3.060	5.62E-01	1.00E+00	0.001
NEFA 22:1	337	-0.884	2.521	7.26E-01	1.00E+00	0.000
NEFA 22:2	283	-5.713	11.585	6.22E-01	1.00E+00	0.001
NEFA 22:3	232	11.419	10.480	2.77E-01	1.00E+00	0.005
NEFA 22:4	337	0.370	0.703	5.99E-01	1.00E+00	0.001
NEFA 22:5	338	0.374	0.370	3.14E-01	1.00E+00	0.003
NEFA 22:6	338	0.120	0.168	4.74E-01	1.00E+00	0.002
NEFA 24:0	338	-2.681	2.867	3.50E-01	1.00E+00	0.003
NEFA 24:1	338	1.883	2.396	4.33E-01	1.00E+00	0.002
NEFA 24:3	150	-3.754	71.113	9.58E-01	1.00E+00	0.000
NEFA 24:4	220	32.004	17.248	6.49E-02	1.00E+00	0.016
NEFA 24:5	166	-21.046	15.863	1.86E-01	1.00E+00	0.011
NEFA 24:6	221	7.128	10.879	5.13E-01	1.00E+00	0.002
NEFA 26:0	287	-18.721	19.291	3.33E-01	1.00E+00	0.003
NEFA 26:1	338	6.430	4.363	1.41E-01	1.00E+00	0.006
NEFA 26:2	288	2.231	5.786	7.00E-01	1.00E+00	0.001
NEFA 26:3	337	-20.699	9.741	3.43E-02	1.00E+00	0.013
NEFA 26:4	167	-32.261	55.046	5.59E-01	1.00E+00	0.002
NEFA 26:5	181	-0.968	52.620	9.85E-01	1.00E+00	0.000
3-Methyl-2-oxovalerate	348	0.043	0.062	4.88E-01	1.00E+00	0.001
4-Methyl-2-oxovalerate	348	0.018	0.037	6.32E-01	1.00E+00	0.001
Acetoacetate	246	-0.005	0.003	5.30E-02	1.00E+00	0.015
Alpha-aminoadipate	161	-0.782	1.027	4.47E-01	1.00E+00	0.004
Alpha-ketobutyrate	334	-0.019	0.013	1.48E-01	1.00E+00	0.006
Citrate	296	-0.027	0.011	1.64E-02	1.00E+00	0.019
Isocitrate	297	-0.019	0.062	7.61E-01	1.00E+00	0.000
Lactate	346	0.000	0.000	6.96E-01	1.00E+00	0.000
Malate	346	-0.042	0.077	5.89E-01	1.00E+00	0.001
Alpha-ketoglutarate	348	0.034	0.050	5.00E-01	1.00E+00	0.001
Pyruvate	226	-0.006	0.005	1.99E-01	1.00E+00	0.007
Succininate	348	0.042	0.069	5.44E-01	1.00E+00	0.001
Taurine	297	0.009	0.012	4.24E-01	1.00E+00	0.002

Sum AA	351	0.001	0.001	2.28E-01	1.00E+00	0.004
Sum Carn	350	0.032	0.015	3.50E-02	1.00E+00	0.013
Sum LPC	53	-0.009	0.008	2.59E-01	1.00E+00	0.025
Sum PCaa	50	0.002	0.004	6.81E-01	1.00E+00	0.004
Sum PCae	51	0.003	0.027	9.11E-01	1.00E+00	0.000
Sum SM	54	0.021	0.034	5.34E-01	1.00E+00	0.008
Sum NEFA	215	-0.001	0.002	7.50E-01	1.00E+00	0.000

UBCS 8 years

Metabolite	N	Estimate	Std..Error	P	P.adjusted	PartialRsquared
Ala	401	0.009	0.003	4.49E-03	9.12E-01	0.019
Arg	400	0.016	0.009	9.90E-02	1.00E+00	0.007
Asn	363	0.067	0.029	1.92E-02	1.00E+00	0.014
Asp	364	0.163	0.055	3.11E-03	6.31E-01	0.023
Cit	402	-0.041	0.029	1.57E-01	1.00E+00	0.005
Gln	401	0.004	0.003	1.74E-01	1.00E+00	0.004
Glu	402	0.025	0.014	6.47E-02	1.00E+00	0.008
Gly	400	0.012	0.005	1.80E-02	1.00E+00	0.013
His	402	0.022	0.013	8.84E-02	1.00E+00	0.007
Ile	361	0.017	0.023	4.59E-01	1.00E+00	0.001
Leu	402	0.012	0.012	3.20E-01	1.00E+00	0.002
Lys	401	0.019	0.008	2.00E-02	1.00E+00	0.013
Met	402	0.089	0.048	6.59E-02	1.00E+00	0.008
Orn	401	0.034	0.016	3.75E-02	1.00E+00	0.010
Phe	402	0.037	0.023	1.02E-01	1.00E+00	0.006
Pro	398	0.003	0.004	4.67E-01	1.00E+00	0.001
Ser	401	-0.005	0.007	5.43E-01	1.00E+00	0.001
Thr	402	0.029	0.009	2.05E-03	4.17E-01	0.022
Trp	401	0.048	0.020	1.48E-02	1.00E+00	0.014
Tyr	401	0.070	0.017	3.69E-05	7.49E-03	0.040
Val	402	0.008	0.006	1.50E-01	1.00E+00	0.005
Hpro	400	0.063	0.026	1.57E-02	1.00E+00	0.014
H1	400	0.001	0.000	1.14E-03	2.31E-01	0.025
Carn	401	0.119	0.037	1.65E-03	3.34E-01	0.024
Carn C2	401	-0.235	0.097	1.58E-02	1.00E+00	0.014
Carn C3	402	2.961	1.803	1.01E-01	1.00E+00	0.006
Carn C4	402	-0.892	3.073	7.72E-01	1.00E+00	0.000
Carn C5	395	-0.938	4.944	8.50E-01	1.00E+00	0.000
Carn C8:1	397	2.124	1.726	2.19E-01	1.00E+00	0.004

Carn C10:1	400	1.597	1.701	3.48E-01	1.00E+00	0.002
Carn C12:1	402	-1.957	1.658	2.39E-01	1.00E+00	0.003
Carn C14:1	401	-3.670	2.335	1.17E-01	1.00E+00	0.006
Carn C16	400	-3.251	6.094	5.94E-01	1.00E+00	0.001
Carn C18	398	-14.488	12.761	2.57E-01	1.00E+00	0.003
Carn C18:1	402	-10.498	5.014	3.69E-02	1.00E+00	0.010
LPC C14	402	2.560	0.536	2.51E-06	5.10E-04	0.052
LPC C15	402	4.620	1.273	3.21E-04	6.51E-02	0.031
LPC C15:1	402	24.069	10.765	2.59E-02	1.00E+00	0.012
LPC C16	402	0.092	0.021	1.33E-05	2.69E-03	0.044
LPC C16:1	401	2.164	0.503	2.14E-05	4.35E-03	0.043
LPC C17	402	1.569	0.983	1.11E-01	1.00E+00	0.006
LPC C17:1	402	9.241	3.457	7.82E-03	1.00E+00	0.017
LPC C18	402	0.195	0.055	4.33E-04	8.80E-02	0.029
LPC C18:1	402	0.159	0.073	3.14E-02	1.00E+00	0.011
LPC C18:2	402	0.067	0.035	5.92E-02	1.00E+00	0.008
LPC C18:3	401	3.063	1.368	2.57E-02	1.00E+00	0.012
LPC C18:6	402	14.543	3.610	6.71E-05	1.36E-02	0.037
LPC C20	402	4.699	3.710	2.06E-01	1.00E+00	0.004
LPC C20:1	402	5.441	3.258	9.57E-02	1.00E+00	0.007
LPC C20:2	402	5.942	3.060	5.28E-02	1.00E+00	0.009
LPC C20:3	400	1.013	0.579	8.10E-02	1.00E+00	0.007
LPC C20:4	400	0.791	0.245	1.36E-03	2.77E-01	0.025
LPC C20:5	400	4.569	1.646	5.77E-03	1.00E+00	0.018

LPC C22:4	402	2.818	2.187	1.98E-01	1.00E+00	0.004
LPC C22:5	402	0.524	1.180	6.57E-01	1.00E+00	0.000
LPC C22:6	401	1.813	0.705	1.04E-02	1.00E+00	0.016
PCaa C18	402	10.509	5.832	7.23E-02	1.00E+00	0.008
PCaa C18:1	402	-3.391	6.980	6.27E-01	1.00E+00	0.001
PCaa C30	402	0.462	0.165	5.39E-03	1.00E+00	0.018
PCaa C30:2	402	0.731	0.562	1.94E-01	1.00E+00	0.004
PCaa C32	402	0.057	0.037	1.20E-01	1.00E+00	0.006
PCaa C32:1	402	0.073	0.040	6.91E-02	1.00E+00	0.008
PCaa C32:2	402	0.443	0.158	5.35E-03	1.00E+00	0.018
PCaa C32:3	402	3.900	1.511	1.02E-02	1.00E+00	0.016
PCaa C34	400	-0.001	0.109	9.95E-01	1.00E+00	0.000
PCaa C34:1	401	0.001	0.005	7.81E-01	1.00E+00	0.000
PCaa C34:2	401	0.002	0.003	4.95E-01	1.00E+00	0.001
PCaa C34:3	402	0.115	0.059	5.42E-02	1.00E+00	0.009
PCaa C34:4	402	1.265	0.335	1.84E-04	3.74E-02	0.033
PCaa C34:5	402	5.376	2.585	3.82E-02	1.00E+00	0.010
PCaa C34:6	388	10.056	5.598	7.32E-02	1.00E+00	0.008
PCaa C36	401	0.256	0.268	3.40E-01	1.00E+00	0.002
PCaa C36:1	402	0.012	0.015	4.03E-01	1.00E+00	0.002
PCaa C36:2	401	0.002	0.006	7.81E-01	1.00E+00	0.000
PCaa C36:3	402	0.012	0.012	3.30E-01	1.00E+00	0.002
PCaa C36:4	402	0.016	0.008	4.01E-02	1.00E+00	0.010
PCaa C36:5	402	0.065	0.045	1.52E-01	1.00E+00	0.005

PCaa C36:6	402	3.604	1.017	4.39E-04	8.92E-02	0.029
PCaa C38	402	0.772	0.381	4.35E-02	1.00E+00	0.010
PCaa C38:1	398	0.180	0.352	6.10E-01	1.00E+00	0.001
PCaa C38:2	402	0.061	0.152	6.88E-01	1.00E+00	0.000
PCaa C38:3	401	0.081	0.030	6.51E-03	1.00E+00	0.018
PCaa C38:4	402	0.026	0.010	6.11E-03	1.00E+00	0.018
PCaa C38:5	402	0.028	0.020	1.70E-01	1.00E+00	0.005
PCaa C38:6	402	0.046	0.017	5.82E-03	1.00E+00	0.018
PCaa C40	401	2.567	1.866	1.70E-01	1.00E+00	0.005
PCaa C40:1	395	2.340	3.527	5.07E-01	1.00E+00	0.001
PCaa C40:2	398	4.404	3.143	1.62E-01	1.00E+00	0.005
PCaa C40:3	394	2.649	1.844	1.52E-01	1.00E+00	0.005
PCaa C40:4	402	0.234	0.204	2.52E-01	1.00E+00	0.003
PCaa C40:5	401	0.117	0.094	2.10E-01	1.00E+00	0.004
PCaa C40:6	402	0.156	0.050	1.80E-03	3.64E-01	0.023
PCaa C42	402	0.173	1.664	9.17E-01	1.00E+00	0.000
PCaa C42:1	400	0.697	3.381	8.37E-01	1.00E+00	0.000
PCaa C42:2	396	4.624	5.556	4.06E-01	1.00E+00	0.002
PCaa C42:4	400	1.515	3.724	6.84E-01	1.00E+00	0.000
PCaa C42:5	399	-0.360	2.302	8.76E-01	1.00E+00	0.000
PCaa C42:6	402	1.172	1.962	5.51E-01	1.00E+00	0.001
PCaa C43:6	402	-0.125	0.756	8.69E-01	1.00E+00	0.000
PCae C30	402	3.274	1.700	5.48E-02	1.00E+00	0.009
PCae C32	399	0.485	0.272	7.49E-02	1.00E+00	0.008

PCae C32:1	402	0.641	0.359	7.49E-02	1.00E+00	0.008
PCae C32:2	402	2.734	1.981	1.68E-01	1.00E+00	0.005
PCae C34	400	0.232	0.366	5.27E-01	1.00E+00	0.001
PCae C34:1	402	0.072	0.111	5.19E-01	1.00E+00	0.001
PCae C34:2	402	0.076	0.082	3.52E-01	1.00E+00	0.002
PCae C34:3	402	0.060	0.093	5.18E-01	1.00E+00	0.001
PCae C34:4	402	3.898	2.534	1.25E-01	1.00E+00	0.006
PCae C36	401	0.362	0.910	6.91E-01	1.00E+00	0.000
PCae C36:1	402	-0.135	0.202	5.05E-01	1.00E+00	0.001
PCae C36:2	402	-0.124	0.100	2.14E-01	1.00E+00	0.004
PCae C36:3	402	0.089	0.144	5.34E-01	1.00E+00	0.001
PCae C36:4	401	0.117	0.054	2.96E-02	1.00E+00	0.011
PCae C36:5	402	0.128	0.077	9.75E-02	1.00E+00	0.007
PCae C36:6	397	1.585	1.142	1.66E-01	1.00E+00	0.005
PCae C38	401	1.266	0.499	1.15E-02	1.00E+00	0.015
PCae C38:2	401	-0.319	0.452	4.81E-01	1.00E+00	0.001
PCae C38:3	402	0.081	0.252	7.48E-01	1.00E+00	0.000
PCae C38:4	400	0.059	0.064	3.54E-01	1.00E+00	0.002
PCae C38:5	401	0.093	0.050	6.15E-02	1.00E+00	0.008
PCae C38:6	402	0.228	0.109	3.72E-02	1.00E+00	0.010
PCae C40	402	0.345	0.204	9.17E-02	1.00E+00	0.007
PCae C40:1	397	0.924	0.462	4.62E-02	1.00E+00	0.010
PCae C40:2	402	0.106	1.251	9.33E-01	1.00E+00	0.000
PCae C40:3	401	-0.357	1.256	7.76E-01	1.00E+00	0.000
PCae C40:4	402	0.002	0.405	9.95E-01	1.00E+00	0.000

PCae C40:5	402	-0.156	0.302	6.05E-01	1.00E+00	0.001
PCae C40:6	402	0.186	0.269	4.90E-01	1.00E+00	0.001
PCae C42	402	1.347	2.396	5.74E-01	1.00E+00	0.001
PCae C42:1	400	1.920	1.300	1.40E-01	1.00E+00	0.005
PCae C42:2	402	0.790	1.347	5.58E-01	1.00E+00	0.001
PCae C42:3	401	0.122	1.306	9.25E-01	1.00E+00	0.000
PCae C42:4	402	-0.851	0.910	3.50E-01	1.00E+00	0.002
PCae C42:5	402	-0.591	0.521	2.57E-01	1.00E+00	0.003
PCae C42:6	402	-0.407	0.792	6.08E-01	1.00E+00	0.001
SM C32:2	326	7.819	1.800	1.87E-05	3.80E-03	0.051
SM C35	401	1.112	0.767	1.48E-01	1.00E+00	0.005
SM C35:1	402	0.278	0.200	1.64E-01	1.00E+00	0.005
SM C36	401	0.531	0.265	4.56E-02	1.00E+00	0.010
SM C36:1	402	0.051	0.032	1.18E-01	1.00E+00	0.006
SM C36:2	402	0.138	0.060	2.34E-02	1.00E+00	0.012
SM C37:1	401	0.349	0.366	3.41E-01	1.00E+00	0.002
SM C38:1	402	0.028	0.025	2.63E-01	1.00E+00	0.003
SM C38:2	402	0.056	0.037	1.27E-01	1.00E+00	0.006
SM C38:3	402	1.748	0.905	5.41E-02	1.00E+00	0.009
SM C39:1	402	0.000	0.180	9.99E-01	1.00E+00	0.000
SM C39:2	400	0.170	0.555	7.59E-01	1.00E+00	0.000
SM C39:5	402	2.255	0.913	1.40E-02	1.00E+00	0.014
SM C40:1	399	-0.007	0.026	7.76E-01	1.00E+00	0.000
SM C40:2	402	0.058	0.038	1.28E-01	1.00E+00	0.006
SM C40:3	399	0.069	0.077	3.74E-01	1.00E+00	0.002

SM C40:4	402	0.204	0.097	3.73E-02	1.00E+00	0.010
SM C41:1	402	-0.163	0.091	7.35E-02	1.00E+00	0.008
SM C41:2	402	0.032	0.139	8.19E-01	1.00E+00	0.000
SM C41:3	394	1.240	0.620	4.61E-02	1.00E+00	0.010
SM C42:1	401	-0.048	0.047	3.02E-01	1.00E+00	0.003
SM C42:2	402	0.015	0.023	5.04E-01	1.00E+00	0.001
SM C42:3	402	0.146	0.054	7.32E-03	1.00E+00	0.017
SM C42:4	401	0.303	0.138	2.91E-02	1.00E+00	0.011
SM C42:6	402	0.489	0.289	9.17E-02	1.00E+00	0.007
SM C43	400	-0.389	1.338	7.71E-01	1.00E+00	0.000
SM C43:1	402	-0.967	0.558	8.38E-02	1.00E+00	0.007
SM C43:2	402	-0.263	0.360	4.67E-01	1.00E+00	0.001
SM C43:3	400	-0.716	1.522	6.38E-01	1.00E+00	0.001
SM C44:2	398	-1.976	1.945	3.10E-01	1.00E+00	0.002
SM C44:6	400	0.792	0.607	1.93E-01	1.00E+00	0.004
NEFA 10:0	323	-0.772	0.285	7.14E-03	1.00E+00	0.021
NEFA 12:0	398	-0.217	0.060	3.22E-04	6.54E-02	0.031
NEFA 12:1	395	-0.840	0.271	2.04E-03	4.14E-01	0.023
NEFA 14:0	401	-0.142	0.034	2.87E-05	5.83E-03	0.041
NEFA 14:1	399	-0.917	0.214	2.25E-05	4.57E-03	0.043
NEFA 15:0	394	-0.589	0.168	5.02E-04	1.02E-01	0.029
NEFA 15:1	335	-4.593	2.375	5.39E-02	1.00E+00	0.010
NEFA 16:0	401	-0.022	0.005	1.47E-06	2.98E-04	0.054
NEFA 16:1	400	-0.097	0.018	1.75E-07	3.56E-05	0.064
NEFA 16:2	398	-5.135	1.756	3.66E-03	7.43E-01	0.020
NEFA 17:0	399	-0.561	0.174	1.36E-03	2.76E-01	0.025
NEFA 17:1	399	-0.968	0.251	1.32E-04	2.68E-02	0.035
NEFA 18:0	398	-0.049	0.012	5.92E-05	1.20E-02	0.038
NEFA 18:1	402	-0.013	0.003	7.00E-06	1.42E-03	0.047
NEFA 18:2	401	-0.050	0.011	9.72E-06	1.97E-03	0.046
NEFA 18:3	401	-0.313	0.090	5.46E-04	1.11E-01	0.028
NEFA 19:1	402	-2.178	0.560	1.18E-04	2.39E-02	0.035
NEFA 20:0	360	-3.307	0.988	9.01E-04	1.83E-01	0.029
NEFA 20:2	351	-1.535	0.459	9.16E-04	1.86E-01	0.029

NEFA 20:3	285	-1.091	0.573	5.78E-02	1.00E+00	0.012
NEFA 20:4	402	-0.214	0.207	3.03E-01	1.00E+00	0.003
NEFA 20:5	387	-1.492	1.348	2.69E-01	1.00E+00	0.003
NEFA 22:4	402	-2.486	1.068	2.04E-02	1.00E+00	0.013
NEFA 22:5	402	-1.165	0.550	3.48E-02	1.00E+00	0.011
NEFA 22:6	402	-0.308	0.312	3.23E-01	1.00E+00	0.002
NEFA 24:0	399	-2.299	3.533	5.16E-01	1.00E+00	0.001
NEFA 24:2	355	4.004	14.746	7.86E-01	1.00E+00	0.000
NEFA 24:4	396	-13.488	17.300	4.36E-01	1.00E+00	0.001
NEFA 24:5	401	-15.937	16.235	3.27E-01	1.00E+00	0.002
NEFA 26:1	402	-1.278	6.144	8.35E-01	1.00E+00	0.000

Sum AA	334	0.003	0.001	2.94E-04	5.97E-02	0.037
Sum Carn	381	0.085	0.035	1.63E-02	1.00E+00	0.014
Sum LPC	397	0.032	0.010	1.14E-03	2.32E-01	0.025
Sum PCaa	399	0.002	0.001	7.34E-02	1.00E+00	0.008
Sum PCae	399	0.012	0.008	1.54E-01	1.00E+00	0.005
Sum SM	325	0.013	0.015	3.93E-01	1.00E+00	0.002
Sum NEFA	274	-0.007	0.002	3.46E-05	7.01E-03	0.058

GINI-LISA 10 years

Metabolite	N	Estimate	Std..Error	P	P.adjusted	PartialRsquared
Ala	250	-0.005	0.004	1.77E-01	1.00E+00	0.007
Arg	250	-0.025	0.017	1.48E-01	1.00E+00	0.008
Asn	250	-0.053	0.031	9.25E-02	1.00E+00	0.011
Asp	249	0.039	0.033	2.30E-01	1.00E+00	0.005
Cit	250	-0.089	0.041	2.94E-02	1.00E+00	0.018
Gln	221	-0.006	0.003	4.27E-02	1.00E+00	0.017
Glu	250	0.010	0.007	1.25E-01	1.00E+00	0.009
Gly	250	-0.001	0.006	7.99E-01	1.00E+00	0.000
His	250	-0.015	0.017	3.62E-01	1.00E+00	0.003
Ile	250	-0.020	0.026	4.42E-01	1.00E+00	0.002
Leu	250	-0.002	0.014	8.68E-01	1.00E+00	0.000
Lys	250	0.002	0.010	8.28E-01	1.00E+00	0.000
Met	250	-0.109	0.075	1.45E-01	1.00E+00	0.008
Orn	250	0.009	0.013	5.04E-01	1.00E+00	0.002
Phe	250	0.003	0.022	9.08E-01	1.00E+00	0.000
Pro	250	0.001	0.006	8.38E-01	1.00E+00	0.000
Ser	250	-0.006	0.012	6.49E-01	1.00E+00	0.001
Thr	250	0.012	0.012	3.03E-01	1.00E+00	0.004
Trp	250	-0.036	0.030	2.22E-01	1.00E+00	0.006
Tyr	250	0.002	0.023	9.32E-01	1.00E+00	0.000
Val	250	-0.003	0.007	6.48E-01	1.00E+00	0.001
H1	252	0.000	0.000	5.69E-01	1.00E+00	0.001
Carn	191	0.143	0.074	5.32E-02	1.00E+00	0.018
Carn C2	195	0.129	0.219	5.58E-01	1.00E+00	0.002
Carn C3	207	4.162	4.992	4.05E-01	1.00E+00	0.003
Carn C4	163	1.022	5.824	8.61E-01	1.00E+00	0.000
Carn C5	153	19.591	12.961	1.33E-01	1.00E+00	0.014
Carn C6	251	7.066	9.038	4.35E-01	1.00E+00	0.002
Carn C8	252	-0.114	1.230	9.26E-01	1.00E+00	0.000
Carn C8:1	251	-2.376	5.750	6.80E-01	1.00E+00	0.001
Carn C9	230	-2.770	14.503	8.49E-01	1.00E+00	0.000
Carn C10	251	-0.132	0.959	8.91E-01	1.00E+00	0.000

Carn C10:1	252	0.212	1.559	8.92E-01	1.00E+00	0.000
Carn C10:2	153	-16.421	9.508	8.62E-02	1.00E+00	0.018
Carn C12	251	-0.035	2.187	9.87E-01	1.00E+00	0.000
Carn C12:1	223	0.855	1.037	4.11E-01	1.00E+00	0.003
Carn C14	162	25.781	18.514	1.66E-01	1.00E+00	0.011
Carn C14:1	251	-0.014	3.364	9.97E-01	1.00E+00	0.000
Carn C14:2	163	-10.718	12.477	3.92E-01	1.00E+00	0.004
Carn C16	230	-2.312	14.065	8.70E-01	1.00E+00	0.000
Carn C16:1	200	15.771	21.940	4.73E-01	1.00E+00	0.002
Carn C18	189	-11.635	16.884	4.92E-01	1.00E+00	0.002
Carn C18:1	252	9.945	8.559	2.46E-01	1.00E+00	0.005
Carn C18:2	178	9.787	19.865	6.23E-01	1.00E+00	0.001
LPC C14	252	-0.155	0.407	7.04E-01	1.00E+00	0.001
LPC C15	234	0.005	1.008	9.96E-01	1.00E+00	0.000
LPC C16	252	0.007	0.015	6.37E-01	1.00E+00	0.001
LPC C16:1	252	0.761	0.451	9.28E-02	1.00E+00	0.010
LPC C17	252	0.222	0.636	7.28E-01	1.00E+00	0.000
LPC C17:1	234	-1.166	3.026	7.00E-01	1.00E+00	0.001
LPC C18	252	0.004	0.043	9.20E-01	1.00E+00	0.000
LPC C18:1	252	-0.043	0.085	6.09E-01	1.00E+00	0.001
LPC C18:2	252	-0.048	0.045	2.80E-01	1.00E+00	0.004
LPC C18:3	234	-2.448	1.541	1.13E-01	1.00E+00	0.010
LPC C18:6	152	6.875	4.014	8.88E-02	1.00E+00	0.017
LPC C20:2	135	0.235	3.515	9.47E-01	1.00E+00	0.000
LPC C20:3	252	0.142	0.579	8.07E-01	1.00E+00	0.000
LPC C20:4	252	0.190	0.208	3.62E-01	1.00E+00	0.003
LPC C20:5	249	-3.686	1.834	4.56E-02	1.00E+00	0.015

LPC C22:5	187	3.054	2.357	1.97E-01	1.00E+00	0.008
LPC C22:6	234	0.160	0.663	8.10E-01	1.00E+00	0.000
PCaa C30	252	-0.712	0.295	1.64E-02	1.00E+00	0.021
PCaa C30:2	178	-0.582	1.989	7.70E-01	1.00E+00	0.000
PCaa C30:3	173	2.092	5.605	7.09E-01	1.00E+00	0.001
PCaa C30:4	126	-6.652	8.591	4.40E-01	1.00E+00	0.004
PCaa C32	252	-0.182	0.132	1.68E-01	1.00E+00	0.007
PCaa C32:1	252	-0.088	0.069	2.06E-01	1.00E+00	0.006
PCaa C32:2	252	-0.803	0.310	1.03E-02	1.00E+00	0.024
PCaa C32:3	197	-0.595	2.161	7.83E-01	1.00E+00	0.000
PCaa C34:1	252	-0.016	0.009	6.60E-02	1.00E+00	0.013
PCaa C34:2	252	-0.009	0.005	6.80E-02	1.00E+00	0.012
PCaa C34:3	252	-0.171	0.083	4.04E-02	1.00E+00	0.016
PCaa C34:4	252	-0.765	0.562	1.75E-01	1.00E+00	0.007
PCaa C34:5	167	-9.320	5.049	6.68E-02	1.00E+00	0.019
PCaa C36	192	-0.178	0.217	4.11E-01	1.00E+00	0.003
PCaa C36:1	252	-0.070	0.032	2.92E-02	1.00E+00	0.018
PCaa C36:2	252	-0.013	0.007	6.02E-02	1.00E+00	0.013
PCaa C36:3	252	-0.015	0.014	2.84E-01	1.00E+00	0.004
PCaa C36:4	252	-0.001	0.011	9.57E-01	1.00E+00	0.000
PCaa C36:5	251	-0.137	0.075	6.79E-02	1.00E+00	0.012

PCaa C36:6	231	-1.114	0.880	2.07E-01	1.00E+00	0.007
PCaa C38	252	-0.332	0.349	3.42E-01	1.00E+00	0.003
PCaa C38:2	117	-0.472	0.305	1.25E-01	1.00E+00	0.018
PCaa C38:3	252	-0.006	0.030	8.45E-01	1.00E+00	0.000
PCaa C38:4	252	0.006	0.014	6.91E-01	1.00E+00	0.001
PCaa C38:5	252	-0.019	0.031	5.34E-01	1.00E+00	0.001
PCaa C38:6	252	-0.004	0.020	8.25E-01	1.00E+00	0.000
PCaa C40	184	-0.394	0.980	6.88E-01	1.00E+00	0.001
PCaa C40:1	200	-0.147	0.677	8.28E-01	1.00E+00	0.000
PCaa C40:3	139	-0.590	0.600	3.27E-01	1.00E+00	0.007
PCaa C40:4	252	-0.397	0.292	1.75E-01	1.00E+00	0.007
PCaa C40:5	252	-0.073	0.115	5.27E-01	1.00E+00	0.001
PCaa C40:6	252	0.004	0.049	9.42E-01	1.00E+00	0.000
PCaa C42	238	-0.247	1.292	8.48E-01	1.00E+00	0.000
PCaa C42:1	124	-1.429	2.436	5.58E-01	1.00E+00	0.003
PCaa C42:4	122	-0.778	1.852	6.75E-01	1.00E+00	0.001
PCaa C42:5	161	-4.227	2.059	4.18E-02	1.00E+00	0.023
PCaa C42:6	138	-0.858	1.250	4.93E-01	1.00E+00	0.003
PCaa C43:6	249	-1.340	1.088	2.19E-01	1.00E+00	0.006
PCaa C44:12	222	-0.427	1.481	7.73E-01	1.00E+00	0.000
PCae C32	252	-0.538	0.481	2.64E-01	1.00E+00	0.005

PCae C32:1	252	-0.563	0.451	2.13E-01	1.00E+00	0.006
PCae C32:2	217	-0.018	1.198	9.88E-01	1.00E+00	0.000
PCae C34	231	-1.822	0.878	3.92E-02	1.00E+00	0.017
PCae C34:1	252	-0.442	0.177	1.31E-02	1.00E+00	0.023
PCae C34:2	252	-0.292	0.132	2.82E-02	1.00E+00	0.018
PCae C34:3	252	-0.355	0.149	1.78E-02	1.00E+00	0.021
PCae C34:4	144	-4.703	3.907	2.31E-01	1.00E+00	0.010
PCae C36	182	-1.394	1.674	4.06E-01	1.00E+00	0.004
PCae C36:1	252	-0.065	0.114	5.71E-01	1.00E+00	0.001
PCae C36:2	252	-0.133	0.105	2.08E-01	1.00E+00	0.006
PCae C36:3	252	-0.379	0.174	3.00E-02	1.00E+00	0.018
PCae C36:4	252	-0.076	0.075	3.10E-01	1.00E+00	0.004
PCae C36:5	252	-0.109	0.109	3.20E-01	1.00E+00	0.004
PCae C36:6	129	-3.172	2.715	2.45E-01	1.00E+00	0.009
PCae C38	251	-0.608	0.397	1.27E-01	1.00E+00	0.009
PCae C38:2	252	-0.075	0.187	6.87E-01	1.00E+00	0.001
PCae C38:3	252	-0.011	0.130	9.31E-01	1.00E+00	0.000
PCae C38:4	252	-0.085	0.102	4.03E-01	1.00E+00	0.003
PCae C38:5	252	-0.061	0.086	4.75E-01	1.00E+00	0.002
PCae C38:6	252	-0.125	0.170	4.63E-01	1.00E+00	0.002
PCae C40	223	-0.084	0.134	5.30E-01	1.00E+00	0.002
PCae C40:1	234	-0.463	0.449	3.04E-01	1.00E+00	0.004
PCae C40:2	171	-0.040	0.452	9.30E-01	1.00E+00	0.000
PCae C40:3	189	-0.020	0.219	9.28E-01	1.00E+00	0.000
PCae C40:4	223	-0.125	0.341	7.16E-01	1.00E+00	0.001

PCae C40:5	252	-0.144	0.243	5.55E-01	1.00E+00	0.001
PCae C40:6	252	-0.271	0.298	3.65E-01	1.00E+00	0.003
PCae C42	194	0.700	1.181	5.54E-01	1.00E+00	0.002
PCae C42:1	172	-0.225	0.655	7.32E-01	1.00E+00	0.001
PCae C42:2	205	-0.814	0.985	4.10E-01	1.00E+00	0.003
PCae C42:3	212	-0.707	0.621	2.57E-01	1.00E+00	0.006
PCae C42:4	190	-0.256	1.239	8.36E-01	1.00E+00	0.000
PCae C42:5	232	-0.558	0.460	2.26E-01	1.00E+00	0.006
PCae C42:6	252	-1.421	0.835	9.01E-02	1.00E+00	0.011
SM C30:1	210	-1.955	2.260	3.88E-01	1.00E+00	0.003
SM C31:1	125	-1.496	5.647	7.92E-01	1.00E+00	0.001
SM C32:1	252	-0.152	0.171	3.75E-01	1.00E+00	0.003
SM C32:2	252	2.228	1.604	1.66E-01	1.00E+00	0.007
SM C33:1	252	-0.275	0.266	3.03E-01	1.00E+00	0.004
SM C33:2	184	8.246	6.428	2.01E-01	1.00E+00	0.008
SM C33:3	181	3.359	11.613	7.73E-01	1.00E+00	0.000
SM C34:1	252	-0.028	0.018	1.19E-01	1.00E+00	0.009
SM C34:2	252	0.045	0.121	7.12E-01	1.00E+00	0.001
SM C35	168	0.762	2.455	7.57E-01	1.00E+00	0.001
SM C35:1	252	0.108	0.535	8.40E-01	1.00E+00	0.000
SM C35:2	203	1.881	3.166	5.53E-01	1.00E+00	0.002
SM C36:1	252	0.000	0.084	1.00E+00	1.00E+00	0.000
SM C36:2	252	0.308	0.153	4.61E-02	1.00E+00	0.015
SM C36:3	252	1.943	1.848	2.94E-01	1.00E+00	0.004
SM C37:1	128	0.617	0.894	4.91E-01	1.00E+00	0.003
SM C37:3	171	-3.616	3.290	2.73E-01	1.00E+00	0.007
SM C38:1	195	-0.055	0.047	2.42E-01	1.00E+00	0.007
SM C38:2	252	-0.074	0.097	4.42E-01	1.00E+00	0.002
SM C38:3	151	3.927	3.114	2.09E-01	1.00E+00	0.010
SM C39:1	252	-0.297	0.233	2.03E-01	1.00E+00	0.006
SM C39:2	152	-1.026	0.894	2.53E-01	1.00E+00	0.008
SM C39:5	123	3.133	2.915	2.85E-01	1.00E+00	0.009
SM C40:2	252	-0.073	0.053	1.73E-01	1.00E+00	0.007

SM C40:4	240	-0.194	0.715	7.87E-01	1.00E+00	0.000
SM C40:5	216	-3.298	1.525	3.17E-02	1.00E+00	0.020
SM C41:1	252	-0.223	0.128	8.18E-02	1.00E+00	0.011
SM C41:2	252	-0.020	0.181	9.11E-01	1.00E+00	0.000
SM C42:1	252	-0.169	0.078	3.04E-02	1.00E+00	0.017
SM C42:2	252	-0.024	0.044	5.85E-01	1.00E+00	0.001
SM C42:3	234	0.026	0.069	7.07E-01	1.00E+00	0.001
SM C42:4	216	0.167	0.195	3.93E-01	1.00E+00	0.003
SM C42:6	240	0.014	0.467	9.76E-01	1.00E+00	0.000
SM C43	144	1.951	2.371	4.12E-01	1.00E+00	0.004
SM C43:1	248	-0.644	1.034	5.34E-01	1.00E+00	0.001
SM C43:2	252	1.092	0.601	7.06E-02	1.00E+00	0.012
SM C44:6	210	-0.805	0.897	3.70E-01	1.00E+00	0.004
NEFA 10:0	240	-1.105	0.410	7.55E-03	1.00E+00	0.028
NEFA 12:0	208	-0.134	0.071	5.84E-02	1.00E+00	0.016
NEFA 12:1	151	0.317	0.552	5.66E-01	1.00E+00	0.002
NEFA 13:0	98	-1.326	1.401	3.46E-01	1.00E+00	0.008
NEFA 13:1	193	-1.925	5.903	7.45E-01	1.00E+00	0.001
NEFA 14:0	251	0.016	0.057	7.81E-01	1.00E+00	0.000
NEFA 14:1	251	0.518	0.342	1.31E-01	1.00E+00	0.008
NEFA 14:2	114	-0.058	0.077	4.52E-01	1.00E+00	0.005
NEFA 16:0	252	0.001	0.009	9.32E-01	1.00E+00	0.000
NEFA 16:1	223	0.049	0.027	7.27E-02	1.00E+00	0.013
NEFA 16:2	225	1.894	1.490	2.05E-01	1.00E+00	0.007
NEFA 17:0	235	-0.159	0.308	6.06E-01	1.00E+00	0.001
NEFA 17:1	223	0.586	0.448	1.93E-01	1.00E+00	0.007
NEFA 17:2	153	-1.707	10.360	8.69E-01	1.00E+00	0.000
NEFA 18:0	164	-0.007	0.024	7.64E-01	1.00E+00	0.001
NEFA 18:1	252	0.004	0.007	5.60E-01	1.00E+00	0.001
NEFA 18:2	252	0.006	0.019	7.66E-01	1.00E+00	0.000
NEFA 18:3	252	-0.023	0.057	6.86E-01	1.00E+00	0.001
NEFA 18:4	230	1.793	3.416	6.00E-01	1.00E+00	0.001
NEFA 19:0	236	-3.342	2.512	1.85E-01	1.00E+00	0.007
NEFA 19:1	239	0.578	0.812	4.77E-01	1.00E+00	0.002
NEFA 20:0	132	-3.217	1.842	8.32E-02	1.00E+00	0.020
NEFA 20:1	252	0.170	0.311	5.84E-01	1.00E+00	0.001
NEFA 20:2	203	-0.363	0.500	4.68E-01	1.00E+00	0.002

NEFA 20:3	230	0.347	0.393	3.79E-01	1.00E+00	0.003
NEFA 20:4	223	0.199	0.200	3.23E-01	1.00E+00	0.004
NEFA 20:5	188	-0.259	0.753	7.32E-01	1.00E+00	0.001
NEFA 22:0	93	1.286	3.399	7.06E-01	1.00E+00	0.002
NEFA 22:1	249	-1.288	2.085	5.37E-01	1.00E+00	0.001
NEFA 22:2	138	9.066	13.715	5.10E-01	1.00E+00	0.003
NEFA 22:3	224	4.960	9.735	6.11E-01	1.00E+00	0.001
NEFA 22:4	224	0.737	1.114	5.09E-01	1.00E+00	0.002
NEFA 22:5	252	-0.272	0.571	6.35E-01	1.00E+00	0.001
NEFA 22:6	252	-0.258	0.243	2.89E-01	1.00E+00	0.004
NEFA 24:0	207	-9.381	4.329	3.14E-02	1.00E+00	0.020
NEFA 24:1	217	-1.698	3.178	5.94E-01	1.00E+00	0.001
NEFA 24:2	165	34.280	26.013	1.89E-01	1.00E+00	0.010
NEFA 24:3	155	-38.001	82.752	6.47E-01	1.00E+00	0.001
NEFA 24:4	223	4.997	20.899	8.11E-01	1.00E+00	0.000
NEFA 24:5	232	-0.030	13.695	9.98E-01	1.00E+00	0.000
NEFA 24:6	193	-2.536	21.664	9.07E-01	1.00E+00	0.000
NEFA 26:0	163	-27.275	16.925	1.09E-01	1.00E+00	0.014
NEFA 26:1	206	-26.171	15.960	1.03E-01	1.00E+00	0.012
NEFA 26:2	196	-2.513	21.774	9.08E-01	1.00E+00	0.000
NEFA 26:3	170	-17.497	25.739	4.98E-01	1.00E+00	0.002
NEFA 26:4	207	-41.124	85.931	6.33E-01	1.00E+00	0.001
NEFA 26:5	195	86.510	116.526	4.59E-01	1.00E+00	0.003
NEFA 26:6	134	-52.320	47.480	2.73E-01	1.00E+00	0.009
Pyruvate	252	-0.004	0.006	4.98E-01	1.00E+00	0.002
Lactate	252	0.000	0.000	5.92E-01	1.00E+00	0.001
Acetoacetate	248	-0.022	0.036	5.33E-01	1.00E+00	0.001
Alpha-ketobutyrate	233	-0.201	0.112	7.42E-02	1.00E+00	0.013
Fumarate	252	1.073	1.140	3.48E-01	1.00E+00	0.003
3-Methyl-2-oxobutanoate	252	-0.152	0.122	2.11E-01	1.00E+00	0.006
Succininate	252	0.000	0.168	9.99E-01	1.00E+00	0.000
Methylmalonate	252	1.538	1.846	4.06E-01	1.00E+00	0.003
Taurine	252	0.020	0.016	2.06E-01	1.00E+00	0.006
3-Methyl-2-oxovalerate	252	-0.191	0.113	9.41E-02	1.00E+00	0.010
4-Methyl-2-oxovalerate	252	-0.128	0.067	5.81E-02	1.00E+00	0.013
Malate	252	0.398	0.638	5.34E-01	1.00E+00	0.001
Alpha-ketoglutarate	251	0.220	0.286	4.42E-01	1.00E+00	0.002
Alpha-aminoadipate	252	3.570	3.744	3.41E-01	1.00E+00	0.003
Isocitrate	239	1.774	2.176	4.16E-01	1.00E+00	0.003
Citrate	252	0.013	0.048	7.81E-01	1.00E+00	0.000

Sum AA	220	0.000	0.001	5.70E-01	1.00E+00	0.001
Sum Carn	133	0.139	0.067	3.97E-02	1.00E+00	0.029
Sum LPC	231	-0.006	0.008	4.70E-01	1.00E+00	0.002
Sum PCaa	251	-0.002	0.001	1.42E-01	1.00E+00	0.008
Sum PCae	222	-0.019	0.011	8.74E-02	1.00E+00	0.012
Sum SM	252	-0.010	0.016	5.20E-01	1.00E+00	0.002
Sum NEFA	81	0.004	0.005	3.68E-01	1.00E+00	0.009

Meta Analyses

Metabolite	N	Estimate	Std..Error	P	P.adjusted	PartialRsquared	Q	Qp
Ala	1005	0.002	0.002	2.50E-01	1.00E+00	0.0007	8.53	0.014
Arg	1004	0.005	0.008	5.18E-01	1.00E+00	0.0003	4.46	0.11
Asn	967	-0.001	0.018	9.59E-01	1.00E+00	0.0000	8.45	0.015
Asp	967	0.064	0.026	1.23E-02	1.00E+00	0.0049	3.79	0.15
Cit	1006	-0.071	0.021	8.78E-04	1.01E-01	0.0054	1.04	0.59
Gln	975	-0.002	0.001	1.54E-01	1.00E+00	0.0010	6.39	0.041
Glu	1006	0.006	0.003	3.83E-02	1.00E+00	0.0020	3.03	0.22
Gly	1004	0.004	0.003	2.03E-01	1.00E+00	0.0010	3.64	0.16
His	1006	0.007	0.008	3.76E-01	1.00E+00	0.0004	3.12	0.21
Ile	965	0.016	0.015	2.99E-01	1.00E+00	0.0006	4.62	0.1
Leu	1006	0.013	0.008	1.00E-01	1.00E+00	0.0014	4.18	0.12
Lys	1005	0.013	0.006	2.62E-02	1.00E+00	0.0030	3.92	0.14
Met	1006	0.020	0.037	5.75E-01	1.00E+00	0.0001	4.96	0.08
Orn	1005	0.019	0.009	3.00E-02	1.00E+00	0.0031	2.67	0.26
Phe	1005	0.019	0.014	1.72E-01	1.00E+00	0.0012	1.23	0.54
Pro	1002	0.003	0.003	3.65E-01	1.00E+00	0.0004	0.14	0.93
Ser	1004	-0.005	0.006	3.92E-01	1.00E+00	0.0003	0.276	0.87
Thr	1006	0.024	0.007	3.27E-04	3.76E-02	0.0072	1.24	0.54
Trp	1005	0.015	0.015	3.11E-01	1.00E+00	0.0005	5.61	0.06
Tyr	1004	0.045	0.012	2.29E-04	2.63E-02	0.0070	6.17	0.046
Val	1006	0.007	0.004	9.02E-02	1.00E+00	0.0014	3.49	0.17
H1	1006	0.000	0.000	4.09E-01	1.00E+00	0.0003	9.94	0.007
Carn	946	0.047	0.014	7.18E-04	8.26E-02	0.0063	5.67	0.06
Carn C2	950	-0.104	0.074	1.59E-01	1.00E+00	0.0011	3.56	0.17
Carn C3	960	0.561	0.879	5.23E-01	1.00E+00	0.0002	2.75	0.25
Carn C5	902	2.586	3.088	4.03E-01	1.00E+00	0.0004	2.24	0.33
Carn C8:1	1002	1.801	1.663	2.79E-01	1.00E+00	0.0005	0.645	0.72

Carn C10:1	1005	-0.366	0.952	7.01E-01	1.00E+00	0.0001	1.96	0.37
Carn C12:1	979	0.334	0.672	6.19E-01	1.00E+00	0.0001	2.49	0.29
Carn C14:1	1005	-0.667	1.374	6.27E-01	1.00E+00	0.0001	2.22	0.33
Carn C16	984	-2.104	5.460	7.00E-01	1.00E+00	0.0001	0.554	0.76
Carn C18	941	-11.250	9.750	2.49E-01	1.00E+00	0.0007	0.091	0.96
Carn C18:1	1008	-3.899	4.075	3.39E-01	1.00E+00	0.0004	4.54	0.1
LPC C16	1008	0.003	0.006	5.76E-01	1.00E+00	0.0001	20.1	<0.0001
LPC C16:1	1007	0.544	0.201	6.88E-03	7.91E-01	0.0035	13.3	0.001
LPC C17	942	0.219	0.306	4.75E-01	1.00E+00	0.0003	2.53	0.28
LPC C18	1008	-0.002	0.017	9.03E-01	1.00E+00	0.0000	14.3	0.001
LPC C18:1	1008	-0.029	0.035	4.12E-01	1.00E+00	0.0003	7.68	0.021
LPC C18:2	1008	-0.030	0.019	1.06E-01	1.00E+00	0.0012	8.35	0.015
LPC C18:3	856	-0.032	0.826	9.69E-01	1.00E+00	0.0000	7.19	0.028
LPC C20:3	939	0.164	0.273	5.49E-01	1.00E+00	0.0002	2.12	0.35
LPC C20:4	1006	0.109	0.098	2.67E-01	1.00E+00	0.0006	9.22	0.01
LPC C20:5	819	0.822	0.934	3.79E-01	1.00E+00	0.0004	11.3	0.004

LPC C22:6	887	0.468	0.355	1.88E-01	1.00E+00	0.0009	3.96	0.14
PCaa C32	1008	0.004	0.035	9.13E-01	1.00E+00	0.0000	5.62	0.06
PCaa C32:1	942	0.036	0.030	2.34E-01	1.00E+00	0.0007	4.77	0.09
PCaa C34:1	1007	-0.004	0.004	2.55E-01	1.00E+00	0.0006	3.04	0.22
PCaa C34:2	942	-0.003	0.002	1.59E-01	1.00E+00	0.0010	4.67	0.1
PCaa C34:3	1008	0.001	0.038	9.72E-01	1.00E+00	0.0000	7.92	0.019
PCaa C34:4	958	0.471	0.256	6.57E-02	1.00E+00	0.0016	9.94	0.007
PCaa C36:2	1007	-0.008	0.003	1.04E-02	1.00E+00	0.0030	2.81	0.25
PCaa C36:3	1008	-0.007	0.007	3.06E-01	1.00E+00	0.0005	2.6	0.27
PCaa C36:4	943	0.006	0.005	2.38E-01	1.00E+00	0.0007	2.21	0.33
PCaa C36:5	938	0.009	0.033	7.80E-01	1.00E+00	0.0000	5.5	0.06

PCaa C38	819	-0.271	0.182	1.38E-01	1.00E+00	0.0012	7.07	0.029
PCaa C38:3	820	0.012	0.019	5.12E-01	1.00E+00	0.0002	5.84	0.054
PCaa C38:4	837	0.014	0.007	4.77E-02	1.00E+00	0.0020	2.37	0.31
PCaa C38:5	892	0.004	0.016	8.11E-01	1.00E+00	0.0000	1.72	0.42
PCaa C38:6	1008	0.009	0.009	3.25E-01	1.00E+00	0.0004	5.98	0.0503
PCaa C40:4	810	-0.073	0.159	6.48E-01	1.00E+00	0.0001	3.19	0.2
PCaa C40:5	824	-0.023	0.065	7.28E-01	1.00E+00	0.0001	3	0.22
PCaa C40:6	1007	0.031	0.025	2.09E-01	1.00E+00	0.0007	7.13	0.028

PCae C34:1	1008	-0.131	0.069	5.92E-02	1.00E+00	0.0017	6.12	0.047
PCae C34:2	941	-0.087	0.053	1.04E-01	1.00E+00	0.0013	5.96	0.051
PCae C34:3	1008	-0.114	0.060	5.75E-02	1.00E+00	0.0017	5.86	0.053
PCae C36:1	893	-0.026	0.052	6.14E-01	1.00E+00	0.0001	0.905	0.64
PCae C36:2	809	-0.109	0.062	7.86E-02	1.00E+00	0.0018	0.901	0.64
PCae C36:3	1008	-0.147	0.072	4.14E-02	1.00E+00	0.0019	4.31	0.12
PCae C36:4	940	-0.010	0.033	7.57E-01	1.00E+00	0.0000	6.3	0.043
PCae C36:5	1008	0.013	0.049	7.97E-01	1.00E+00	0.0000	3.28	0.19
PCae C38	834	-0.143	0.233	5.39E-01	1.00E+00	0.0002	8.79	0.012
PCae C38:3	836	-0.075	0.064	2.42E-01	1.00E+00	0.0007	0.494	0.78
PCae C38:4	825	-0.016	0.053	7.66E-01	1.00E+00	0.0000	1.56	0.46
PCae C38:5	941	0.008	0.035	8.21E-01	1.00E+00	0.0000	3.09	0.21
PCae C38:6	892	0.072	0.080	3.70E-01	1.00E+00	0.0004	3.08	0.21
PCae C40	790	-0.066	0.086	4.43E-01	1.00E+00	0.0004	3.59	0.17
PCae C40:4	796	-0.162	0.184	3.79E-01	1.00E+00	0.0004	0.1	0.95

PCae C40:6	825	-0.189	0.171	2.70E-01	1.00E+00	0.0007	1.97	0.37
SM C32:2	749	3.364	0.967	5.34E-04	6.14E-02	0.0081	8.05	0.018
SM C35:1	958	0.272	0.176	1.23E-01	1.00E+00	0.0011	0.1	0.95
SM C36:1	1008	0.061	0.027	2.53E-02	1.00E+00	0.0023	1.17	0.56
SM C36:2	875	0.195	0.056	5.82E-04	6.70E-02	0.0062	1.49	0.47

SM C41:1	892	-0.163	0.062	8.24E-03	9.48E-01	0.0035	0.739	0.69
SM C41:2	891	0.085	0.086	3.24E-01	1.00E+00	0.0005	1.4	0.5
SM C42:1	841	-0.093	0.036	9.30E-03	1.00E+00	0.0036	1.91	0.38
SM C42:2	827	0.016	0.018	3.83E-01	1.00E+00	0.0004	2.5	0.29
NEFA 12:0	943	-0.200	0.043	4.32E-06	4.97E-04	0.0108	1.4	0.5
NEFA 14:0	990	-0.062	0.023	8.66E-03	9.96E-01	0.0032	8.79	0.012
NEFA 14:1	987	-0.143	0.137	3.00E-01	1.00E+00	0.0005	18.4	<0.0001
NEFA 16:0	991	-0.009	0.003	5.55E-03	6.39E-01	0.0036	12.1	0.002
NEFA 16:1	961	-0.021	0.013	1.04E-01	1.00E+00	0.0013	25.1	<0.0001
NEFA 16:2	961	0.001	0.867	9.99E-01	1.00E+00	0.0000	10.2	0.006
NEFA 17:0	972	-0.230	0.117	4.92E-02	1.00E+00	0.0019	4.53	0.1
NEFA 17:1	960	-0.208	0.180	2.47E-01	1.00E+00	0.0007	13.3	0.001
NEFA 18:1	992	-0.007	0.002	1.91E-03	2.19E-01	0.0045	8.51	0.014
NEFA 18:2	989	-0.023	0.008	2.94E-03	3.38E-01	0.0041	9.02	0.011
NEFA 18:3	991	-0.042	0.033	2.04E-01	1.00E+00	0.0008	10.5	0.005
NEFA 19:1	979	-0.455	0.374	2.23E-01	1.00E+00	0.0007	12.2	0.002
NEFA 20:2	891	-0.493	0.245	4.40E-02	1.00E+00	0.0022	7.17	0.028

NEFA 20:3	853	0.110	0.175	5.30E-01	1.00E+00	0.0002	4.61	0.1
NEFA 20:4	963	0.035	0.052	4.99E-01	1.00E+00	0.0002	2.08	0.35
NEFA 20:5	911	-0.117	0.414	7.78E-01	1.00E+00	0.0000	1.64	0.44
NEFA 22:4	963	-0.008	0.522	9.88E-01	1.00E+00	0.0000	5.94	0.051
NEFA 22:5	992	-0.089	0.273	7.46E-01	1.00E+00	0.0000	5.45	0.07
NEFA 22:6	992	-0.049	0.126	6.94E-01	1.00E+00	0.0001	2.45	0.29
NEFA 24:0	944	-4.108	2.009	4.12E-02	1.00E+00	0.0022	1.99	0.37
NEFA 24:4	839	8.292	10.692	4.38E-01	1.00E+00	0.0003	3.5	0.17
NEFA 26:1	946	1.601	3.598	6.56E-01	1.00E+00	0.0001	4.42	0.11

Sum AA	905	0.001	0.000	3.88E-02	1.00E+00	0.0028	8.24	0.016
Sum Carn	864	0.040	0.013	3.30E-03	3.80E-01	0.0056	4.05	0.13
Sum LPC	681	0.000	0.005	9.90E-01	1.00E+00	0.0000	12.3	0.002
Sum PCaa	700	0.000	0.001	9.58E-01	1.00E+00	0.0000	5.18	0.08
Sum PCae	672	-0.005	0.007	4.44E-01	1.00E+00	0.0004	4.98	0.08
Sum SM	631	0.002	0.011	8.22E-01	1.00E+00	0.0000	1.4	0.5
Sum NEFA	570	-0.003	0.001	1.66E-02	1.00E+00	0.0055	9.3	0.01

Supplemental Table 5 Associations of metabolites with HOMA in all studies and follow-ups, and in the meta-analysis. Associations between HOMA levels and metabolites for each study follow-up visit were calculated in linear models adjusted for child age and sex and individual participant meta-analysis. Statistical significance was evaluated using Bonferroni corrected p-values (p.adjust) < 0.05 (with dark grey background). AA, amino acids; Carn, Acylcarnitines; lyso.PC, lyso-phosphatidylcholine; NEFA, nonesterified fatty acid; PCaa, Diacyl-phosphatidylcholine; PCae, Alkyl-acyl-phosphatidylcholine; SM, sphingomyelin.

CHOP 5.5 years

Metabolite	N	Estimate	Std..Error	P	P.adjusted	PartialRsquared
Ala	374	7.95E-04	3.52E-04	2.45E-02	1.00E+00	0.014
Arg	372	1.93E-03	1.62E-03	2.34E-01	1.00E+00	0.004
Asn	374	9.02E-04	2.85E-03	7.52E-01	1.00E+00	0.000
Asp	372	1.87E-03	9.53E-03	8.45E-01	1.00E+00	0.000
Cit	374	-3.94E-03	3.68E-03	2.84E-01	1.00E+00	0.003
Gln	374	-2.17E-04	2.29E-04	3.44E-01	1.00E+00	0.002
Glu	374	-2.63E-04	3.50E-04	4.54E-01	1.00E+00	0.002
Gly	374	-3.17E-04	6.10E-04	6.04E-01	1.00E+00	0.001
His	374	6.37E-04	1.50E-03	6.71E-01	1.00E+00	0.000
Ile	374	-1.63E-03	1.79E-03	3.65E-01	1.00E+00	0.002
Leu	374	-7.91E-04	1.10E-03	4.72E-01	1.00E+00	0.001
Lys	374	7.83E-04	8.61E-04	3.64E-01	1.00E+00	0.002
Met	374	9.97E-03	6.30E-03	1.15E-01	1.00E+00	0.007
Orn	374	2.22E-03	1.54E-03	1.51E-01	1.00E+00	0.006
Phe	374	-5.44E-04	2.62E-03	8.36E-01	1.00E+00	0.000
Pro	374	9.50E-04	5.24E-04	7.07E-02	1.00E+00	0.009
Ser	373	-1.14E-03	1.32E-03	3.86E-01	1.00E+00	0.002
Thr	374	1.64E-03	9.80E-04	9.51E-02	1.00E+00	0.007
Trp	373	2.78E-04	2.63E-03	9.16E-01	1.00E+00	0.000
Tyr	374	4.23E-03	1.89E-03	2.58E-02	1.00E+00	0.013
Val	374	-2.07E-04	5.33E-04	6.99E-01	1.00E+00	0.000
H1	375	9.42E-05	3.63E-05	9.82E-03	1.00E+00	0.018
Carn	379	1.17E-02	3.21E-03	2.97E-04	6.91E-02	0.034
Carn C2	379	-2.22E-02	7.99E-03	5.78E-03	1.00E+00	0.020
Carn C3	379	1.14E+00	2.92E-01	1.07E-04	2.50E-02	0.039
Carn C3:1	268	5.92E+00	5.22E+00	2.58E-01	1.00E+00	0.005
Carn C4	379	5.86E-01	2.70E-01	3.09E-02	1.00E+00	0.012
Carn C4:0:OH	268	-1.90E+00	3.67E-01	4.60E-07	1.07E-04	0.092
Carn C4:1	338	-1.20E+00	9.33E-01	1.98E-01	1.00E+00	0.005
Carn C5	379	1.45E+00	7.82E-01	6.37E-02	1.00E+00	0.009
Carn C5:0:OH	308	3.92E+01	6.41E+01	5.41E-01	1.00E+00	0.001
Carn C6	309	-2.30E+00	9.97E-01	2.15E-02	1.00E+00	0.017
Carn C6:1	203	-4.05E+01	8.52E+00	3.91E-06	9.12E-04	0.102
Carn C8	314	-5.76E-01	1.90E-01	2.64E-03	6.16E-01	0.029
Carn C8:1	379	-1.68E+00	5.24E-01	1.46E-03	3.41E-01	0.027
Carn C9	379	-1.23E+00	2.03E+00	5.44E-01	1.00E+00	0.001

Carn C10	378	-3.51E-01	1.28E-01	6.57E-03	1.00E+00	0.020
Carn C10:1	379	-8.66E-02	1.70E-01	6.11E-01	1.00E+00	0.001
Carn C10:2	379	8.66E-01	1.52E+00	5.68E-01	1.00E+00	0.001
Carn C12::DC	253	-2.51E-01	3.96E-01	5.27E-01	1.00E+00	0.002
Carn C12	378	-1.27E+00	3.44E-01	2.47E-04	5.75E-02	0.035
Carn C12:1	379	-3.51E-01	1.27E-01	6.11E-03	1.00E+00	0.020
Carn C14	379	-2.76E+00	9.63E-01	4.40E-03	1.00E+00	0.021
Carn C14:1	377	-1.01E+00	2.45E-01	4.78E-05	1.11E-02	0.043
Carn C14:2	379	-2.06E+00	1.12E+00	6.63E-02	1.00E+00	0.009
Carn C14:2:OH	245	3.23E+00	3.10E+00	2.98E-01	1.00E+00	0.004
Carn C15	379	-8.06E-01	2.70E+00	7.66E-01	1.00E+00	0.000
Carn C16	378	-1.10E+00	1.05E+00	2.96E-01	1.00E+00	0.003
Carn C16:OH	378	-3.37E+00	1.13E+00	3.02E-03	7.03E-01	0.023
Carn C16:1	279	5.70E-01	6.48E+00	9.30E-01	1.00E+00	0.000
Carn C16:2	268	1.85E+00	3.86E+00	6.32E-01	1.00E+00	0.001
Carn C16:2:OH	249	2.15E+00	4.83E+00	6.57E-01	1.00E+00	0.001
Carn C18	379	-5.26E-01	2.03E+00	7.96E-01	1.00E+00	0.000
Carn C18:1	379	-4.70E-01	6.62E-01	4.79E-01	1.00E+00	0.001
Carn C18:1:OH	313	-8.18E-01	4.16E+00	8.44E-01	1.00E+00	0.000
Carn C18:2	379	9.06E-01	9.05E-01	3.17E-01	1.00E+00	0.003
Carn C18:2:OH	244	8.33E+00	1.20E+01	4.87E-01	1.00E+00	0.002
Carn C20	377	-8.17E+00	6.46E+00	2.07E-01	1.00E+00	0.004
Carn C20:1	307	5.50E+00	1.70E+02	9.74E-01	1.00E+00	0.000
Carn C20:3	379	8.79E+00	4.22E+00	3.76E-02	1.00E+00	0.011
Carn C20:4	379	1.02E+02	4.81E+01	3.41E-02	1.00E+00	0.012
Carn C22	280	1.04E+02	7.36E+01	1.59E-01	1.00E+00	0.007
Carn C22:5	269	1.17E+01	1.05E+01	2.65E-01	1.00E+00	0.005
Carn C22:6	248	5.13E+01	4.20E+01	2.23E-01	1.00E+00	0.006
LPC C14	235	1.09E-01	4.65E-02	1.95E-02	1.00E+00	0.023
LPC C15	241	1.08E-01	9.25E-02	2.45E-01	1.00E+00	0.006
LPC C16	379	2.08E-03	1.03E-03	4.40E-02	1.00E+00	0.011
LPC C16:1	379	1.54E-02	2.95E-02	6.02E-01	1.00E+00	0.001
LPC C17	378	4.66E-02	4.35E-02	2.85E-01	1.00E+00	0.003
LPC C18	379	6.37E-03	2.70E-03	1.90E-02	1.00E+00	0.015
LPC C18:1	379	6.28E-03	5.76E-03	2.77E-01	1.00E+00	0.003
LPC C18:2	379	3.05E-03	2.96E-03	3.04E-01	1.00E+00	0.003
LPC C18:3	339	1.00E-01	1.01E-01	3.23E-01	1.00E+00	0.003
LPC C20:1	192	-8.11E-02	1.75E-01	6.44E-01	1.00E+00	0.001
LPC C20:3	377	3.59E-02	3.00E-02	2.33E-01	1.00E+00	0.004
LPC C20:4	379	1.74E-02	1.40E-02	2.14E-01	1.00E+00	0.004
LPC C20:5	310	-2.84E-01	1.34E-01	3.41E-02	1.00E+00	0.014

LPC C22:6	309	-4.64E-02	4.30E-02	2.81E-01	1.00E+00	0.004
PCaa C20:4	207	1.48E+00	6.22E-01	1.86E-02	1.00E+00	0.027
PCaa C28:2	310	7.87E-01	6.41E-01	2.21E-01	1.00E+00	0.005
PCaa C30	379	1.83E-02	1.84E-02	3.20E-01	1.00E+00	0.003
PCaa C30:2	379	4.79E-02	1.23E-01	6.97E-01	1.00E+00	0.000
PCaa C30:3	378	3.07E-01	4.09E-01	4.54E-01	1.00E+00	0.001
PCaa C30:4	162	1.67E+00	1.28E+00	1.92E-01	1.00E+00	0.010
PCaa C32	379	-7.61E-03	7.87E-03	3.34E-01	1.00E+00	0.002
PCaa C32:1	379	-6.03E-03	5.50E-03	2.74E-01	1.00E+00	0.003
PCaa C32:2	379	8.12E-03	2.77E-02	7.70E-01	1.00E+00	0.000
PCaa C34:1	379	-2.85E-04	5.72E-04	6.19E-01	1.00E+00	0.001
PCaa C34:2	379	-1.39E-04	3.88E-04	7.20E-01	1.00E+00	0.000
PCaa C34:3	378	-9.65E-03	6.12E-03	1.15E-01	1.00E+00	0.007
PCaa C34:4	379	3.04E-02	4.89E-02	5.34E-01	1.00E+00	0.001
PCaa C36	267	-2.11E-03	1.40E-02	8.81E-01	1.00E+00	0.000
PCaa C36:1	379	-1.48E-03	2.01E-03	4.63E-01	1.00E+00	0.001
PCaa C36:2	379	4.39E-05	5.09E-04	9.31E-01	1.00E+00	0.000
PCaa C36:3	379	-6.49E-04	1.00E-03	5.18E-01	1.00E+00	0.001
PCaa C36:4	379	-2.64E-04	7.48E-04	7.24E-01	1.00E+00	0.000
PCaa C36:5	377	-1.86E-02	7.88E-03	1.89E-02	1.00E+00	0.015
PCaa C36:6	311	-8.00E-02	4.28E-02	6.23E-02	1.00E+00	0.011
PCaa C38	307	-6.01E-04	2.51E-02	9.81E-01	1.00E+00	0.000
PCaa C38:3	379	-3.49E-04	2.62E-03	8.94E-01	1.00E+00	0.000
PCaa C38:4	379	-5.12E-04	1.01E-03	6.14E-01	1.00E+00	0.001
PCaa C38:5	379	-9.24E-03	2.76E-03	8.85E-04	2.06E-01	0.029
PCaa C38:6	379	-2.20E-03	1.67E-03	1.89E-01	1.00E+00	0.005
PCaa C40	272	6.28E-03	4.96E-02	8.99E-01	1.00E+00	0.000
PCaa C40:4	378	-2.70E-02	2.41E-02	2.64E-01	1.00E+00	0.003
PCaa C40:5	379	-2.06E-02	8.41E-03	1.48E-02	1.00E+00	0.016
PCaa C40:6	379	-5.98E-03	3.89E-03	1.25E-01	1.00E+00	0.006
PCaa C42	236	-2.43E-02	9.24E-02	7.92E-01	1.00E+00	0.000
PCaa C42:1	206	-4.74E-02	1.88E-01	8.01E-01	1.00E+00	0.000
PCaa C42:6	193	3.02E-02	1.20E-01	8.02E-01	1.00E+00	0.000

PCaa C43:6	245	1.40E-01	6.50E-02	3.25E-02	1.00E+00	0.019
PCaa C44:12	379	-2.22E-03	4.99E-02	9.65E-01	1.00E+00	0.000
PCae C30:2	272	1.32E-01	2.75E-01	6.30E-01	1.00E+00	0.001
PCae C32	379	-3.29E-02	3.65E-02	3.68E-01	1.00E+00	0.002
PCae C32:1	379	-2.82E-02	3.06E-02	3.58E-01	1.00E+00	0.002
PCae C32:2	202	-1.27E-01	1.05E-01	2.28E-01	1.00E+00	0.007
PCae C34	379	-5.88E-02	5.01E-02	2.41E-01	1.00E+00	0.004
PCae C34:1	379	-2.36E-02	1.07E-02	2.85E-02	1.00E+00	0.013
PCae C34:2	379	-8.88E-03	9.09E-03	3.29E-01	1.00E+00	0.003
PCae C34:3	379	-4.94E-03	1.07E-02	6.44E-01	1.00E+00	0.001
PCae C34:4	241	-1.30E-01	2.45E-01	5.98E-01	1.00E+00	0.001
PCae C36	247	-7.16E-02	5.03E-02	1.55E-01	1.00E+00	0.008
PCae C36:1	379	-5.94E-03	4.88E-03	2.24E-01	1.00E+00	0.004
PCae C36:2	379	-4.23E-03	5.97E-03	4.79E-01	1.00E+00	0.001
PCae C36:3	379	-9.20E-04	1.07E-02	9.32E-01	1.00E+00	0.000
PCae C36:4	378	3.40E-03	5.49E-03	5.36E-01	1.00E+00	0.001
PCae C36:5	379	5.34E-03	7.08E-03	4.51E-01	1.00E+00	0.002
PCae C36:6	169	-9.57E-02	1.10E-01	3.86E-01	1.00E+00	0.005
PCae C38	308	-5.32E-02	2.15E-02	1.38E-02	1.00E+00	0.020
PCae C38:2	300	1.36E-03	7.55E-03	8.57E-01	1.00E+00	0.000
PCae C38:3	378	-1.54E-03	6.89E-03	8.23E-01	1.00E+00	0.000
PCae C38:4	379	7.40E-04	7.23E-03	9.19E-01	1.00E+00	0.000
PCae C38:5	379	3.00E-03	5.67E-03	5.97E-01	1.00E+00	0.001
PCae C38:6	379	6.88E-03	1.22E-02	5.74E-01	1.00E+00	0.001
PCae C40	379	-2.43E-02	9.57E-03	1.16E-02	1.00E+00	0.017
PCae C40:1	229	4.55E-03	4.10E-02	9.12E-01	1.00E+00	0.000
PCae C40:3	311	-1.17E-02	8.85E-03	1.88E-01	1.00E+00	0.006
PCae C40:4	340	-3.63E-03	1.63E-02	8.24E-01	1.00E+00	0.000
PCae C40:5	379	-3.19E-02	1.64E-02	5.20E-02	1.00E+00	0.010
PCae C40:6	379	-2.68E-02	2.09E-02	2.01E-01	1.00E+00	0.004
PCae C42:2	315	-3.53E-02	5.12E-02	4.92E-01	1.00E+00	0.002
PCae C42:3	272	-2.68E-02	4.22E-02	5.25E-01	1.00E+00	0.001
PCae C42:4	191	-3.86E-02	1.04E-01	7.11E-01	1.00E+00	0.001
PCae C42:5	309	-8.36E-02	3.39E-02	1.41E-02	1.00E+00	0.019
PCae C42:6	379	-8.03E-02	5.46E-02	1.43E-01	1.00E+00	0.006
SM C21:2	340	2.09E+00	1.28E+00	1.02E-01	1.00E+00	0.008
SM C32:1	379	2.78E-02	1.27E-02	2.90E-02	1.00E+00	0.013
SM C32:2	340	1.57E-01	1.68E-01	3.50E-01	1.00E+00	0.003
SM C33:1	379	-3.96E-03	2.25E-02	8.61E-01	1.00E+00	0.000
SM C33:3	177	-9.06E-01	6.12E-01	1.41E-01	1.00E+00	0.012
SM C34:1	379	-6.55E-04	1.26E-03	6.04E-01	1.00E+00	0.001

SM C34:2	379	2.13E-02	8.97E-03	1.81E-02	1.00E+00	0.015
SM C34:4	211	-1.41E+00	2.09E+00	5.02E-01	1.00E+00	0.002
SM C35:1	379	-6.09E-03	3.64E-02	8.67E-01	1.00E+00	0.000
SM C35:2	193	-2.14E-01	2.95E-01	4.69E-01	1.00E+00	0.003
SM C36:1	378	-1.16E-03	6.21E-03	8.51E-01	1.00E+00	0.000
SM C36:2	379	3.27E-03	1.17E-02	7.79E-01	1.00E+00	0.000
SM C36:3	182	1.17E-01	1.21E-01	3.35E-01	1.00E+00	0.005
SM C37:1	193	2.22E-02	3.56E-02	5.33E-01	1.00E+00	0.002
SM C37:3	202	-2.68E-01	2.61E-01	3.06E-01	1.00E+00	0.005
SM C38:2	379	-2.93E-03	7.67E-03	7.03E-01	1.00E+00	0.000
SM C39:1	309	3.76E-02	1.86E-02	4.42E-02	1.00E+00	0.013
SM C40:2	232	-4.10E-03	4.60E-03	3.74E-01	1.00E+00	0.003
SM C40:4	300	-1.50E-02	5.30E-02	7.77E-01	1.00E+00	0.000
SM C40:5	340	-2.12E-01	9.13E-02	2.09E-02	1.00E+00	0.016
SM C41:1	379	8.13E-04	6.66E-03	9.03E-01	1.00E+00	0.000
SM C41:2	379	8.11E-03	9.66E-03	4.02E-01	1.00E+00	0.002
SM C42:1	379	1.81E-03	4.63E-03	6.97E-01	1.00E+00	0.000
SM C42:2	379	-1.27E-03	2.69E-03	6.38E-01	1.00E+00	0.001
SM C42:3	311	-5.62E-03	5.21E-03	2.82E-01	1.00E+00	0.004
SM C42:6	379	-1.57E-02	3.26E-02	6.30E-01	1.00E+00	0.001
SM C43	168	-1.10E-01	1.68E-01	5.13E-01	1.00E+00	0.003
SM C43:1	197	4.47E-04	7.23E-02	9.95E-01	1.00E+00	0.000
SM C43:2	311	-2.33E-02	3.56E-02	5.14E-01	1.00E+00	0.001
SM C44:6	230	-4.98E-02	9.33E-02	5.94E-01	1.00E+00	0.001
SM C47:6	315	-7.21E+00	5.11E+00	1.59E-01	1.00E+00	0.006
NEFA 12:0	176	8.93E-03	9.24E-03	3.35E-01	1.00E+00	0.005
NEFA 14:0	312	-6.99E-03	3.49E-03	4.62E-02	1.00E+00	0.013
NEFA 14:1	376	-3.55E-04	5.54E-03	9.49E-01	1.00E+00	0.000
NEFA 15:0	176	-3.15E-02	3.92E-02	4.23E-01	1.00E+00	0.004

NEFA 16:0	241	-1.49E-03	5.35E-04	5.83E-03	1.00E+00	0.031
NEFA 16:1	376	-4.05E-03	1.68E-03	1.64E-02	1.00E+00	0.015
NEFA 16:2	297	-7.28E-02	6.58E-02	2.69E-01	1.00E+00	0.004
NEFA 17:0	163	-2.89E-02	1.98E-02	1.46E-01	1.00E+00	0.013
NEFA 17:1	376	-1.35E-02	1.48E-02	3.63E-01	1.00E+00	0.002
NEFA 17:2	163	-1.34E+00	7.12E-01	6.25E-02	1.00E+00	0.022
NEFA 18:1	376	-5.65E-04	2.74E-04	3.99E-02	1.00E+00	0.011
NEFA 18:2	376	-8.80E-04	6.34E-04	1.66E-01	1.00E+00	0.005
NEFA 18:3	376	-2.41E-02	8.70E-03	5.97E-03	1.00E+00	0.020
NEFA 18:4	163	-5.45E-01	4.03E-01	1.78E-01	1.000	0.011
NEFA 19:1	296	-2.16E-01	7.81E-02	6.02E-03	1.00E+00	0.026
NEFA 20:1	376	-5.54E-02	1.84E-02	2.78E-03	6.48E-01	0.024
NEFA 20:2	376	-1.01E-03	1.00E-02	9.20E-01	1.00E+00	0.000
NEFA 20:3	376	2.72E-02	2.08E-02	1.92E-01	1.00E+00	0.005
NEFA 20:4	376	-1.56E-03	6.70E-03	8.17E-01	1.00E+00	0.000
NEFA 20:5	294	-1.25E-02	4.30E-02	7.71E-01	1.00E+00	0.000
NEFA 22:3	227	-2.24E+00	1.22E+00	6.75E-02	1.00E+00	0.015
NEFA 22:4	376	-9.18E-02	8.78E-02	2.97E-01	1.00E+00	0.003
NEFA 22:5	374	-6.79E-02	4.30E-02	1.15E-01	1.00E+00	0.007
NEFA 22:6	376	1.56E-02	1.79E-02	3.84E-01	1.00E+00	0.002
NEFA 24:0	163	-2.11E-02	2.99E-01	9.44E-01	1.00E+00	0.000
NEFA 24:1	376	-4.56E-02	1.21E-01	7.06E-01	1.00E+00	0.000
NEFA 24:2	163	2.64E-01	2.49E+00	9.16E-01	1.00E+00	0.000
NEFA 24:3	233	-8.03E-01	1.73E+00	6.43E-01	1.00E+00	0.001
NEFA 24:4	376	-1.21E-01	1.52E+00	9.37E-01	1.00E+00	0.000
NEFA 24:6	161	-4.13E+00	4.37E+00	3.46E-01	1.00E+00	0.005
NEFA 26:0	321	2.48E-01	1.95E-01	2.05E-01	1.00E+00	0.005
NEFA 26:1	306	3.99E-01	2.09E-01	5.74E-02	1.00E+00	0.012
NEFA 26:2	376	4.96E-01	2.53E-01	5.08E-02	1.00E+00	0.010
NEFA 26:3	296	4.36E-02	9.40E-01	9.63E-01	1.00E+00	0.000
NEFA 26:4	376	2.00E+00	3.30E+00	5.45E-01	1.00E+00	0.001
Pyruvate	372	4.43E-04	1.53E-04	3.98E-03	9.28E-01	0.022
Lactic_acid	376	8.29E-06	1.50E-05	5.80E-01	1.00E+00	0.001
Fumarate	370	-1.01E-01	1.09E-01	3.52E-01	1.00E+00	0.002
3-Methyl-2-oxobutanoate	372	2.64E-03	2.11E-03	2.12E-01	1.00E+00	0.004
Succininate	374	-8.42E-03	8.94E-03	3.47E-01	1.00E+00	0.002
Methylmalonate	376	6.12E-01	4.18E-01	1.44E-01	1.00E+00	0.006
Taurine	374	-6.57E-04	7.81E-04	4.01E-01	1.00E+00	0.002

3-Methyl-2-oxovalerate	375	-9.86E-04	2.41E-03	6.82E-01	1.00E+00	0.000
4-Methyl-2-oxovalerate	371	3.09E-04	1.17E-03	7.92E-01	1.00E+00	0.000
Malate	373	-4.91E-03	1.71E-02	7.75E-01	1.00E+00	0.000
Alpha-ketoglutarate	366	6.30E-03	5.38E-03	2.42E-01	1.00E+00	0.004
Alpha-aminoadipate	375	1.85E-01	1.87E-01	3.24E-01	1.00E+00	0.003
Isocitrate	376	-3.97E-03	5.36E-03	4.59E-01	1.00E+00	0.001
Citrate	376	1.07E-05	4.03E-04	9.79E-01	1.00E+00	0.000
Beta-hydroxybutyrate	148	-2.88E-03	1.13E-03	1.21E-02	1.00E+00	0.043
	371	3.95E-05	7.02E-05	5.74E-01	1.00E+00	0.001
Sum AA	376	6.19E-03	2.90E-03	3.36E-02	1.00E+00	0.012
Sum Carn	202	2.55E-04	8.51E-04	7.65E-01	1.00E+00	0.000
Sum LPC	304	-8.22E-05	1.20E-04	4.94E-01	1.00E+00	0.002
Sum PCaa	268	-6.46E-04	1.04E-03	5.36E-01	1.00E+00	0.001
Sum PCae	339	3.48E-05	1.11E-03	9.75E-01	1.00E+00	0.000
Sum SM	96	-3.68E-04	1.70E-04	3.29E-02	1.00E+00	0.046
Sum NEFA						

CHOP 8 years

Metabolite	N	Estimate	Std..Error	P	P.adjusted	PartialRsquared
Ala	349	3.98E-03	5.04E-04	4.03E-14	7.66E-12	0.152
Arg	349	1.10E-02	2.40E-03	5.87E-06	1.12E-03	0.058
Asn	349	1.65E-02	3.98E-03	4.49E-05	8.53E-03	0.047
Asp	349	-6.92E-02	1.21E-02	2.22E-08	4.22E-06	0.086
Cit	349	8.70E-03	5.57E-03	1.19E-01	1.00E+00	0.007
Gln	348	1.15E-03	2.49E-04	5.25E-06	9.97E-04	0.058
Glu	349	-2.41E-03	4.00E-04	4.19E-09	7.97E-07	0.095
Gly	349	1.64E-03	8.78E-04	6.28E-02	1.00E+00	0.010
His	349	5.44E-04	1.60E-03	7.34E-01	1.00E+00	0.000
Ile	349	-1.73E-03	3.87E-03	6.55E-01	1.00E+00	0.001
Leu	349	-1.35E-05	2.21E-03	9.95E-01	1.00E+00	0.000
Lys	349	6.21E-03	2.49E-03	1.32E-02	1.00E+00	0.018
Met	349	2.45E-02	8.99E-03	6.81E-03	1.00E+00	0.021
Orn	349	2.61E-03	2.62E-03	3.20E-01	1.00E+00	0.003
Phe	348	3.17E-03	4.30E-03	4.61E-01	1.00E+00	0.002
Pro	349	6.64E-04	1.00E-03	5.08E-01	1.00E+00	0.001
Ser	348	-1.65E-03	1.31E-03	2.10E-01	1.00E+00	0.005
Thr	349	7.09E-03	1.63E-03	1.81E-05	3.44E-03	0.052
Trp	349	9.62E-03	4.01E-03	1.70E-02	1.00E+00	0.016
Tyr	348	1.95E-02	2.96E-03	1.67E-10	3.17E-08	0.112
Val	349	1.64E-03	1.04E-03	1.17E-01	1.00E+00	0.007
H1	349	3.20E-04	6.21E-05	4.18E-07	7.95E-05	0.071
Carn	349	8.25E-03	1.84E-03	1.02E-05	1.94E-03	0.054
Carn C2	349	-6.45E-02	1.46E-02	1.28E-05	2.42E-03	0.053
Carn C3	346	-9.55E-02	1.23E-01	4.38E-01	1.00E+00	0.002
Carn C3:1	284	4.34E+00	1.88E+00	2.18E-02	1.00E+00	0.018
Carn C4:0:OH	236	-2.40E+00	4.97E-01	2.51E-06	4.76E-04	0.090
Carn C5	349	-2.94E+01	2.76E+01	2.88E-01	1.00E+00	0.003
Carn C5:0:OH	349	-9.46E-01	4.97E-01	5.77E-02	1.00E+00	0.010
Carn C6	349	-1.37E+00	7.93E-01	8.62E-02	1.00E+00	0.008
Carn C6:1	349	-1.34E+00	1.03E+00	1.96E-01	1.00E+00	0.005
Carn C8	349	-4.59E-01	2.25E-01	4.20E-02	1.00E+00	0.012
Carn C8:1	349	7.85E-01	5.75E-01	1.73E-01	1.00E+00	0.005
Carn C9	349	-1.44E+00	2.13E+00	5.01E-01	1.00E+00	0.001

Carn C10	349	-3.81E-01	1.32E-01	4.15E-03	7.89E-01	0.023
Carn C10:1	348	7.13E-02	2.20E-01	7.46E-01	1.00E+00	0.000
Carn C10:2	349	2.74E+00	1.60E+00	8.70E-02	1.00E+00	0.008
Carn C12	349	-1.30E+00	3.35E-01	1.25E-04	2.38E-02	0.041
Carn C12:1	349	-4.92E-01	1.41E-01	5.34E-04	1.01E-01	0.034
Carn C14	349	-8.48E+00	1.37E+00	1.55E-09	2.94E-07	0.099
Carn C14:1	348	-1.08E+00	2.28E-01	3.31E-06	6.29E-04	0.060
Carn C14:2	349	-2.47E+00	1.09E+00	2.40E-02	1.00E+00	0.014
Carn C14:2:OH	347	-1.20E+01	3.33E+00	3.68E-04	6.99E-02	0.036
Carn C15	349	-3.37E+01	7.65E+00	1.45E-05	2.75E-03	0.053
Carn C16	349	-8.89E+00	1.70E+00	2.75E-07	5.22E-05	0.073
Carn C16:0:OH	286	-8.83E+01	2.08E+01	3.09E-05	5.88E-03	0.059
Carn C16:1	349	-7.68E+00	1.46E+00	2.67E-07	5.07E-05	0.073
Carn C16:2	349	-1.26E+01	3.89E+00	1.29E-03	2.46E-01	0.029
Carn C16:2:OH	300	-4.24E+01	7.46E+00	3.11E-08	5.92E-06	0.098
Carn C18	349	-1.19E+01	3.83E+00	2.10E-03	3.98E-01	0.027
Carn C18:1	349	-6.62E+00	1.10E+00	5.12E-09	9.72E-07	0.093
Carn C18:1:OH	349	-2.65E+01	5.08E+00	3.12E-07	5.93E-05	0.072
Carn C18:2	347	1.57E-01	1.89E+00	9.34E-01	1.00E+00	0.000
Carn C18:2:OH	229	5.18E+00	1.36E+01	7.03E-01	1.00E+00	0.001
Carn C20	343	-7.02E+00	7.41E+00	3.44E-01	1.00E+00	0.003
Carn C20:1	348	1.03E+03	5.38E+02	5.57E-02	1.00E+00	0.011
Carn C20:3	349	3.20E+00	2.05E+01	8.76E-01	1.00E+00	0.000
Carn C20:4	300	4.43E+02	2.69E+02	1.00E-01	1.00E+00	0.009
Carn C22	219	3.62E+02	2.33E+02	1.21E-01	1.00E+00	0.011
Carn C22:5	251	1.57E+01	4.07E+01	6.99E-01	1.00E+00	0.001
Carn C22:6	182	-2.29E+01	3.17E+01	4.71E-01	1.00E+00	0.003
LPC C16	349	-4.73E-03	8.36E-04	3.24E-08	6.16E-06	0.084
LPC C16:1	349	-9.94E-02	3.07E-02	1.34E-03	2.55E-01	0.029
LPC C17	286	-2.51E-01	4.72E-02	2.12E-07	4.02E-05	0.091
LPC C18	349	-1.16E-02	2.32E-03	8.85E-07	1.68E-04	0.067
LPC C18:1	349	-2.59E-02	5.37E-03	2.14E-06	4.07E-04	0.063
LPC C18:2	349	7.02E-03	3.01E-03	2.01E-02	1.00E+00	0.015
LPC C18:3	218	1.28E-01	1.69E-01	4.51E-01	1.00E+00	0.003
LPC C18:6	178	2.51E-01	2.30E-01	2.78E-01	1.00E+00	0.007
LPC C20:3	282	8.78E-03	4.23E-02	8.36E-01	1.00E+00	0.000
LPC C20:4	349	-3.93E-03	1.56E-02	8.01E-01	1.00E+00	0.000
LPC C20:5	165	-1.12E-01	1.52E-01	4.62E-01	1.00E+00	0.003
LPC C22:5	151	1.96E-01	1.82E-01	2.84E-01	1.00E+00	0.008

LPC C22:6	250	-2.57E-01	6.45E-02	8.82E-05	1.67E-02	0.060
PCaa C30:5	180	1.17E+01	1.92E+01	5.44E-01	1.00E+00	0.002
PCaa C32	349	-6.89E-03	1.05E-02	5.11E-01	1.00E+00	0.001
PCaa C32:1	286	1.50E-02	6.74E-03	2.65E-02	1.00E+00	0.017
PCaa C34:1	349	-8.11E-04	6.77E-04	2.32E-01	1.00E+00	0.004
PCaa C34:2	283	1.39E-03	4.71E-04	3.40E-03	6.46E-01	0.030
PCaa C34:3	349	1.62E-02	7.16E-03	2.42E-02	1.00E+00	0.015
PCaa C34:4	300	2.57E-01	5.70E-02	9.79E-06	1.86E-03	0.064
PCaa C36:2	349	1.84E-03	4.65E-04	9.16E-05	1.74E-02	0.043
PCaa C36:3	349	2.31E-03	1.38E-03	9.46E-02	1.00E+00	0.008
PCaa C36:4	283	2.22E-03	1.10E-03	4.51E-02	1.00E+00	0.014
PCaa C36:5	280	4.94E-03	6.79E-03	4.68E-01	1.00E+00	0.002
PCaa C38	163	-4.94E-02	3.21E-02	1.25E-01	1.00E+00	0.014
PCaa C38:3	161	1.37E-03	5.04E-03	7.87E-01	1.00E+00	0.000
PCaa C38:4	182	2.38E-03	1.81E-03	1.90E-01	1.00E+00	0.010
PCaa C38:5	237	-8.70E-03	4.58E-03	5.86E-02	1.00E+00	0.015
PCaa C38:6	349	-1.66E-03	1.73E-03	3.37E-01	1.00E+00	0.003
PCaa C40:4	153	1.50E-02	4.25E-02	7.25E-01	1.00E+00	0.001
PCaa C40:5	170	1.03E-03	1.56E-02	9.47E-01	1.00E+00	0.000
PCaa C40:6	348	1.26E-03	4.36E-03	7.73E-01	1.00E+00	0.000

PCaa C44:12	171	-1.52E-01	1.79E-01	3.97E-01	1.00E+00	0.004
PCae C34:1	349	-9.51E-03	1.21E-02	4.33E-01	1.00E+00	0.002
PCae C34:2	282	2.63E-02	8.81E-03	3.04E-03	5.78E-01	0.030
PCae C34:3	349	-4.40E-03	1.07E-02	6.81E-01	1.00E+00	0.000
PCae C36:1	234	-2.07E-02	7.32E-03	5.13E-03	9.75E-01	0.033
PCae C36:2	152	3.23E-03	1.23E-02	7.94E-01	1.00E+00	0.000
PCae C36:3	349	1.38E-02	1.15E-02	2.31E-01	1.00E+00	0.004
PCae C36:4	282	1.16E-02	5.51E-03	3.61E-02	1.00E+00	0.015
PCae C36:5	349	1.20E-02	9.25E-03	1.95E-01	1.00E+00	0.005
PCae C38	181	-8.86E-02	4.98E-02	7.67E-02	1.00E+00	0.018
PCae C38:3	181	-2.40E-02	1.08E-02	2.78E-02	1.00E+00	0.027
PCae C38:4	171	-1.09E-02	1.69E-02	5.19E-01	1.00E+00	0.002
PCae C38:5	286	1.29E-02	6.72E-03	5.53E-02	1.00E+00	0.013
PCae C38:6	237	9.99E-03	1.81E-02	5.81E-01	1.00E+00	0.001
PCae C40	163	-3.57E-02	1.82E-02	5.15E-02	1.00E+00	0.023
PCae C40:4	170	-6.54E-02	3.03E-02	3.23E-02	1.00E+00	0.027
PCae C40:6	170	-1.59E-02	3.59E-02	6.59E-01	1.00E+00	0.001
SM C21:2	178	2.75E-02	9.00E-01	9.76E-01	1.00E+00	0.000
SM C31:1	181	1.47E-01	6.52E-01	8.22E-01	1.00E+00	0.000
SM C32:1	349	-3.34E-02	1.68E-02	4.78E-02	1.00E+00	0.011
SM C32:2	170	-4.17E-02	2.03E-01	8.38E-01	1.00E+00	0.000
SM C33:1	349	-1.03E-01	2.25E-02	7.52E-06	1.43E-03	0.056
SM C34:1	349	-4.61E-03	1.60E-03	4.10E-03	7.79E-01	0.023

SM C34:2	349	-2.11E-02	1.08E-02	5.08E-02	1.00E+00	0.011
SM C35:1	300	-1.35E-01	4.36E-02	2.12E-03	4.02E-01	0.031
SM C36:1	349	-1.89E-02	6.06E-03	1.94E-03	3.68E-01	0.027
SM C36:2	219	-1.52E-02	1.73E-02	3.80E-01	1.00E+00	0.003
SM C36:3	281	-2.78E-01	1.22E-01	2.33E-02	1.00E+00	0.018
SM C41:1	237	-2.68E-02	1.30E-02	4.00E-02	1.00E+00	0.018
SM C41:2	236	-2.37E-02	1.66E-02	1.55E-01	1.00E+00	0.009
SM C42:1	185	-8.23E-03	8.26E-03	3.20E-01	1.00E+00	0.005
SM C42:2	171	-6.82E-03	4.38E-03	1.21E-01	1.00E+00	0.014
NEFA 10:0	334	-6.90E-02	1.45E-02	2.86E-06	5.43E-04	0.064
NEFA 11:0	261	-2.70E-01	4.37E-01	5.37E-01	1.00E+00	0.001
NEFA 12:0	333	-3.79E-02	2.05E-02	6.49E-02	1.00E+00	0.010
NEFA 12:1	218	-1.14E+00	4.82E-01	1.90E-02	1.00E+00	0.025
NEFA 14:0	334	-2.58E-02	4.33E-03	6.21E-09	1.18E-06	0.096
NEFA 14:1	333	-1.00E-01	2.39E-02	3.32E-05	6.31E-03	0.050
NEFA 15:0	334	-1.39E-01	1.99E-02	1.97E-11	3.73E-09	0.126
NEFA 15:1	211	-1.28E+00	3.22E-01	9.45E-05	1.80E-02	0.069

NEFA 16:0	334	-3.76E-03	5.50E-04	4.09E-11	7.78E-09	0.122
NEFA 16:1	334	-1.29E-02	2.48E-03	3.40E-07	6.45E-05	0.075
NEFA 16:2	334	-4.47E-01	1.72E-01	9.83E-03	1.00E+00	0.020
NEFA 17:0	334	-1.32E-01	2.01E-02	1.68E-10	3.19E-08	0.115
NEFA 17:1	334	-2.05E-01	3.40E-02	4.75E-09	9.03E-07	0.098
NEFA 18:0	279	-7.29E-03	1.42E-03	5.02E-07	9.53E-05	0.086
NEFA 18:1	334	-2.38E-03	4.18E-04	2.58E-08	4.90E-06	0.089
NEFA 18:2	332	-6.31E-03	1.42E-03	1.15E-05	2.19E-03	0.056
NEFA 18:3	334	-1.60E-02	5.97E-03	7.74E-03	1.00E+00	0.021
NEFA 18:4	332	-7.20E-01	3.90E-01	6.57E-02	1.00E+00	0.010
NEFA 19:0	279	-1.25E+00	2.38E-01	3.24E-07	6.16E-05	0.090
NEFA 19:1	334	-3.93E-01	7.19E-02	9.40E-08	1.79E-05	0.082
NEFA 20:0	334	-7.05E-01	9.83E-02	5.03E-12	9.56E-10	0.133
NEFA 20:1	334	-1.38E-01	2.45E-02	3.59E-08	6.82E-06	0.087
NEFA 20:2	333	-1.67E-01	4.30E-02	1.21E-04	2.31E-02	0.044
NEFA 20:3	334	-1.64E-01	2.51E-02	2.43E-10	4.62E-08	0.113
NEFA 20:4	334	-4.62E-02	6.55E-03	9.95E-12	1.89E-09	0.130
NEFA 20:5	332	-4.26E-01	6.65E-02	5.16E-10	9.81E-08	0.110
NEFA 22:0	285	-9.60E-01	3.81E-01	1.23E-02	1.00E+00	0.022
NEFA 22:1	333	-1.60E+00	3.10E-01	4.10E-07	7.78E-05	0.074
NEFA 22:2	279	-4.88E+00	1.54E+00	1.75E-03	3.33E-01	0.035
NEFA 22:3	230	-3.35E+00	1.39E+00	1.65E-02	1.00E+00	0.025
NEFA 22:4	333	-4.40E-01	8.58E-02	5.02E-07	9.53E-05	0.073
NEFA 22:5	334	-2.36E-01	4.48E-02	2.46E-07	4.67E-05	0.077
NEFA 22:6	334	-1.11E-01	2.02E-02	8.66E-08	1.65E-05	0.082
NEFA 24:0	334	-6.48E-01	3.70E-01	8.11E-02	1.00E+00	0.009
NEFA 24:1	334	-1.71E+00	2.96E-01	1.80E-08	3.42E-06	0.091
NEFA 24:3	145	-1.39E+01	8.57E+00	1.07E-01	1.00E+00	0.018
NEFA 24:4	218	-4.84E+00	1.99E+00	1.57E-02	1.00E+00	0.026
NEFA 24:5	164	-4.73E+00	1.99E+00	1.88E-02	1.00E+00	0.033
NEFA 24:6	219	-2.32E+00	1.25E+00	6.45E-02	1.00E+00	0.015
NEFA 26:0	285	-3.14E+00	2.42E+00	1.96E-01	1.00E+00	0.006
NEFA 26:1	334	5.02E-01	5.52E-01	3.64E-01	1.00E+00	0.002
NEFA 26:2	285	8.55E-01	7.40E-01	2.49E-01	1.00E+00	0.005
NEFA 26:3	333	1.87E+00	1.24E+00	1.30E-01	1.00E+00	0.007
NEFA 26:4	164	-7.67E+00	7.22E+00	2.90E-01	1.00E+00	0.007
NEFA 26:5	180	-9.73E+00	7.30E+00	1.84E-01	1.00E+00	0.010
3-Methyl-2-oxovalerate	343	-4.66E-03	7.75E-03	5.48E-01	1.00E+00	0.001
4-Methyl-2-oxovalerate	343	-2.49E-03	4.54E-03	5.84E-01	1.00E+00	0.001
Acetoacetate	243	-7.13E-04	3.40E-04	3.72E-02	1.00E+00	0.018
Alpha-aminoadipate	158	3.08E-01	1.25E-01	1.49E-02	1.00E+00	0.038
Alpha-ketobutyrate	329	-2.03E-03	1.66E-03	2.22E-01	1.00E+00	0.005
Citrate	293	-2.26E-03	1.41E-03	1.12E-01	1.00E+00	0.009
Isocitrate	294	-8.68E-03	7.73E-03	2.62E-01	1.00E+00	0.004

Lactate	341	9.27E-05	5.43E-05	8.87E-02	1.00E+00	0.008
Malate	341	-4.79E-02	9.28E-03	4.11E-07	7.82E-05	0.073
Alpha-ketoglutarate	343	-4.56E-03	6.73E-03	4.98E-01	1.00E+00	0.001
Pyruvate	222	2.71E-03	5.77E-04	4.65E-06	8.84E-04	0.091
Succininate	343	-4.25E-02	8.25E-03	4.53E-07	8.61E-05	0.072
Taurine	294	-1.33E-03	1.43E-03	3.51E-01	1.00E+00	0.003
Sum AA	346	6.45E-04	1.43E-04	9.35E-06	1.78E-03	0.056
Sum Carn	345	6.67E-03	1.86E-03	3.85E-04	7.32E-02	0.036
Sum LPC	53	-2.16E-03	5.47E-04	2.53E-04	4.81E-02	0.240
Sum PCaa	48	2.62E-04	4.42E-04	5.57E-01	1.00E+00	0.008
Sum PCae	49	-4.38E-05	3.15E-03	9.89E-01	1.00E+00	0.000
Sum SM	54	-3.24E-03	2.75E-03	2.45E-01	1.00E+00	0.026
Sum NEFA	213	-8.19E-04	2.14E-04	1.68E-04	0.032	0.064

UBCS 8 years

Metabolite	N	Estimate	Std..Error	P	P.adjusted	PartialRsquared
Ala	379	1.33E-03	1.96E-04	5.55E-11	1.13E-08	0.107
Arg	378	2.52E-04	6.04E-04	6.76E-01	1.00E+00	0.000
Asn	343	1.68E-03	1.81E-03	3.53E-01	1.00E+00	0.002
Asp	344	6.94E-03	3.48E-03	4.68E-02	1.00E+00	0.011
Cit	380	-1.61E-03	1.82E-03	3.76E-01	1.00E+00	0.002
Gln	379	6.93E-05	1.71E-04	6.86E-01	1.00E+00	0.000
Glu	380	1.48E-03	8.76E-04	9.24E-02	1.00E+00	0.007
Gly	378	9.26E-04	3.27E-04	4.90E-03	9.94E-01	0.021
His	380	7.91E-04	8.29E-04	3.40E-01	1.00E+00	0.002
Ile	341	1.37E-03	1.49E-03	3.56E-01	1.00E+00	0.002
Leu	380	2.44E-04	7.78E-04	7.54E-01	1.00E+00	0.000
Lys	379	1.08E-03	5.14E-04	3.62E-02	1.00E+00	0.011
Met	380	8.19E-03	3.01E-03	6.89E-03	1.00E+00	0.019
Orn	379	2.28E-03	1.05E-03	3.05E-02	1.00E+00	0.012
Phe	380	2.12E-03	1.50E-03	1.60E-01	1.00E+00	0.005
Pro	376	9.80E-04	2.15E-04	7.32E-06	1.49E-03	0.051
Ser	379	-3.12E-04	4.72E-04	5.09E-01	1.00E+00	0.001
Thr	380	1.60E-03	5.97E-04	7.76E-03	1.00E+00	0.018
Trp	379	3.29E-03	1.25E-03	9.14E-03	1.00E+00	0.018
Tyr	379	6.50E-03	1.04E-03	1.10E-09	2.24E-07	0.093
Val	380	5.36E-04	3.71E-04	1.49E-01	1.00E+00	0.005
Hpro	378	2.03E-03	1.64E-03	2.15E-01	1.00E+00	0.004
H1	379	1.52E-04	2.96E-05	4.16E-07	8.43E-05	0.064
Carn	379	1.10E-02	2.32E-03	3.26E-06	6.62E-04	0.055
Carn C2	379	-3.60E-02	5.95E-03	3.29E-09	6.69E-07	0.087
Carn C3	380	2.48E-01	1.13E-01	2.83E-02	1.00E+00	0.012
Carn C4	380	1.58E-01	1.93E-01	4.12E-01	1.00E+00	0.002
Carn C5	374	-1.43E-01	3.10E-01	6.46E-01	1.00E+00	0.001
Carn C8:1	376	-2.01E-02	1.09E-01	8.54E-01	1.00E+00	0.000

Carn C10:1	378	-2.80E-01	1.08E-01	9.49E-03	1.00E+00	0.017
Carn C12:1	380	-5.15E-01	1.03E-01	9.28E-07	1.88E-04	0.060
Carn C14:1	379	-7.08E-01	1.44E-01	1.31E-06	2.67E-04	0.059
Carn C16	378	-2.12E+00	3.72E-01	2.39E-08	4.86E-06	0.078
Carn C18	376	-3.37E+00	7.86E-01	2.39E-05	4.86E-03	0.046
Carn C18:1	380	-1.57E+00	3.09E-01	5.80E-07	1.18E-04	0.063
LPC C14	380	1.30E-01	3.52E-02	2.69E-04	5.47E-02	0.034
LPC C15	380	3.10E-02	8.28E-02	7.08E-01	1.00E+00	0.000
LPC C15:1	380	3.71E-01	6.88E-01	5.90E-01	1.00E+00	0.001
LPC C16	380	4.53E-03	1.41E-03	1.40E-03	2.85E-01	0.026
LPC C16:1	379	7.76E-02	3.33E-02	2.04E-02	1.00E+00	0.014
LPC C17	380	-3.37E-02	6.28E-02	5.91E-01	1.00E+00	0.001
LPC C17:1	380	2.41E-02	2.19E-01	9.13E-01	1.00E+00	0.000
LPC C18	380	1.18E-02	3.58E-03	1.09E-03	2.21E-01	0.027
LPC C18:1	380	1.07E-02	4.70E-03	2.39E-02	1.00E+00	0.013
LPC C18:2	380	3.62E-03	2.24E-03	1.07E-01	1.00E+00	0.007
LPC C18:3	379	2.50E-01	8.66E-02	4.19E-03	8.50E-01	0.021
LPC C18:6	380	7.07E-01	2.37E-01	3.03E-03	6.16E-01	0.023
LPC C20	380	-1.38E-01	2.34E-01	5.56E-01	1.00E+00	0.001
LPC C20:1	380	4.75E-02	2.04E-01	8.16E-01	1.00E+00	0.000
LPC C20:2	380	1.58E-01	1.95E-01	4.17E-01	1.00E+00	0.002
LPC C20:3	379	4.10E-02	3.69E-02	2.67E-01	1.00E+00	0.003
LPC C20:4	379	1.54E-02	1.56E-02	3.26E-01	1.00E+00	0.003
LPC C20:5	378	7.61E-02	1.05E-01	4.70E-01	1.00E+00	0.001
LPC C22:4	380	-1.58E-01	1.38E-01	2.55E-01	1.00E+00	0.003
LPC C22:5	380	-6.58E-02	7.59E-02	3.86E-01	1.00E+00	0.002

LPC C22:6	379	-3.21E-02	4.48E-02	4.74E-01	1.00E+00	0.001
PCaa C18	380	-9.26E-02	3.69E-01	8.02E-01	1.00E+00	0.000
PCaa C18:1	380	-9.18E-01	4.39E-01	3.72E-02	1.00E+00	0.011
PCaa C30	380	3.34E-03	1.05E-02	7.51E-01	1.00E+00	0.000
PCaa C30:2	380	-6.93E-02	3.57E-02	5.30E-02	1.00E+00	0.010
PCaa C32	380	-3.65E-03	2.28E-03	1.10E-01	1.00E+00	0.007
PCaa C32:1	380	1.52E-03	2.56E-03	5.52E-01	1.00E+00	0.001
PCaa C32:2	380	1.41E-02	1.02E-02	1.70E-01	1.00E+00	0.005
PCaa C32:3	380	3.64E-02	9.69E-02	7.08E-01	1.00E+00	0.000
PCaa C34	378	-1.04E-02	6.79E-03	1.26E-01	1.00E+00	0.006
PCaa C34:1	379	-4.05E-04	3.28E-04	2.17E-01	1.00E+00	0.004
PCaa C34:2	379	-1.63E-04	1.83E-04	3.75E-01	1.00E+00	0.002
PCaa C34:3	380	3.39E-03	3.81E-03	3.75E-01	1.00E+00	0.002
PCaa C34:4	380	4.07E-02	2.19E-02	6.35E-02	1.00E+00	0.009
PCaa C34:5	380	2.08E-01	1.63E-01	2.03E-01	1.00E+00	0.004
PCaa C34:6	366	-2.24E-01	3.54E-01	5.27E-01	1.00E+00	0.001
PCaa C36	379	-2.90E-02	1.67E-02	8.35E-02	1.00E+00	0.008
PCaa C36:1	380	-2.13E-04	9.18E-04	8.17E-01	1.00E+00	0.000
PCaa C36:2	379	4.18E-04	3.70E-04	2.60E-01	1.00E+00	0.003
PCaa C36:3	380	-2.44E-04	7.59E-04	7.48E-01	1.00E+00	0.000
PCaa C36:4	380	-8.63E-04	5.02E-04	8.66E-02	1.00E+00	0.008
PCaa C36:5	380	1.80E-03	2.85E-03	5.29E-01	1.00E+00	0.001
PCaa C36:6	380	1.02E-01	6.49E-02	1.16E-01	1.00E+00	0.006
PCaa C38	380	-3.04E-02	2.41E-02	2.08E-01	1.00E+00	0.004
PCaa C38:1	376	-2.02E-02	2.20E-02	3.60E-01	1.00E+00	0.002
PCaa C38:2	380	3.07E-03	9.45E-03	7.45E-01	1.00E+00	0.000
PCaa C38:3	379	1.64E-03	1.92E-03	3.95E-01	1.00E+00	0.002
PCaa C38:4	380	-6.53E-04	6.16E-04	2.90E-01	1.00E+00	0.003
PCaa C38:5	380	-2.33E-03	1.26E-03	6.49E-02	1.00E+00	0.009
PCaa C38:6	380	-1.97E-03	1.04E-03	5.85E-02	1.00E+00	0.009
PCaa C40	379	-1.79E-01	1.17E-01	1.28E-01	1.00E+00	0.006
PCaa C40:1	374	-9.76E-02	2.20E-01	6.57E-01	1.00E+00	0.001
PCaa C40:2	376	-3.89E-02	1.97E-01	8.44E-01	1.00E+00	0.000
PCaa C40:3	374	-5.69E-02	1.15E-01	6.20E-01	1.00E+00	0.001
PCaa C40:4	380	-9.05E-03	1.28E-02	4.80E-01	1.00E+00	0.001
PCaa C40:5	379	-9.58E-03	5.78E-03	9.84E-02	1.00E+00	0.007
PCaa C40:6	380	7.09E-05	3.18E-03	9.82E-01	1.00E+00	0.000
PCaa C42	380	-1.22E-01	1.05E-01	2.46E-01	1.00E+00	0.003
PCaa C42:1	378	-2.93E-01	2.13E-01	1.69E-01	1.00E+00	0.005
PCaa C42:2	374	4.38E-01	3.44E-01	2.03E-01	1.00E+00	0.004
PCaa C42:4	378	-3.44E-01	2.32E-01	1.39E-01	1.00E+00	0.006
PCaa C42:5	377	-1.43E-01	1.45E-01	3.24E-01	1.00E+00	0.003
PCaa C42:6	380	-1.42E-01	1.23E-01	2.49E-01	1.00E+00	0.003

PCaa C43:6	380	-5.66E-02	4.75E-02	2.34E-01	1.00E+00	0.004
PCae C30	380	-1.36E-01	1.07E-01	2.05E-01	1.00E+00	0.004
PCae C32	377	-3.27E-02	1.69E-02	5.40E-02	1.00E+00	0.010
PCae C32:1	380	-5.93E-02	2.23E-02	8.24E-03	1.00E+00	0.018
PCae C32:2	380	-2.64E-01	1.24E-01	3.41E-02	1.00E+00	0.012
PCae C34	378	-5.15E-02	2.26E-02	2.29E-02	1.00E+00	0.013
PCae C34:1	380	-1.70E-02	6.83E-03	1.31E-02	1.00E+00	0.016
PCae C34:2	380	-3.70E-03	5.29E-03	4.85E-01	1.00E+00	0.001
PCae C34:3	380	-9.79E-03	5.98E-03	1.03E-01	1.00E+00	0.007
PCae C34:4	380	-1.87E-02	1.66E-01	9.11E-01	1.00E+00	0.000
PCae C36	379	-1.01E-01	5.63E-02	7.24E-02	1.00E+00	0.008
PCae C36:1	380	-3.90E-02	1.24E-02	1.82E-03	3.70E-01	0.025
PCae C36:2	380	-1.94E-02	6.19E-03	1.83E-03	3.71E-01	0.025
PCae C36:3	380	-3.62E-03	9.13E-03	6.92E-01	1.00E+00	0.000
PCae C36:4	379	-2.14E-03	3.44E-03	5.33E-01	1.00E+00	0.001
PCae C36:5	380	-6.80E-03	4.92E-03	1.68E-01	1.00E+00	0.005
PCae C36:6	375	4.06E-02	7.26E-02	5.76E-01	1.00E+00	0.001
PCae C38	379	-2.98E-02	3.16E-02	3.48E-01	1.00E+00	0.002
PCae C38:2	379	-6.84E-02	2.79E-02	1.49E-02	1.00E+00	0.015
PCae C38:3	380	-4.71E-02	1.56E-02	2.79E-03	5.65E-01	0.023
PCae C38:4	378	-1.14E-02	3.96E-03	4.36E-03	8.85E-01	0.021
PCae C38:5	379	-2.93E-03	3.16E-03	3.54E-01	1.00E+00	0.002
PCae C38:6	380	-6.66E-03	7.01E-03	3.42E-01	1.00E+00	0.002
PCae C40	380	-3.12E-02	1.26E-02	1.41E-02	1.00E+00	0.016
PCae C40:1	375	-3.12E-02	2.95E-02	2.90E-01	1.00E+00	0.003
PCae C40:2	380	-2.36E-01	7.80E-02	2.63E-03	5.33E-01	0.023
PCae C40:3	379	-1.87E-01	7.76E-02	1.62E-02	1.00E+00	0.015
PCae C40:4	380	-7.71E-02	2.49E-02	2.13E-03	4.31E-01	0.024
PCae C40:5	380	-6.60E-02	1.85E-02	4.05E-04	8.23E-02	0.032
PCae C40:6	380	-4.92E-02	1.67E-02	3.39E-03	6.89E-01	0.022
PCae C42	380	-3.77E-01	1.48E-01	1.13E-02	1.00E+00	0.017
PCae C42:1	378	-1.42E-01	8.28E-02	8.81E-02	1.00E+00	0.008
PCae C42:2	380	-2.30E-01	8.34E-02	6.02E-03	1.00E+00	0.019
PCae C42:3	379	-2.10E-01	8.05E-02	9.32E-03	1.00E+00	0.017
PCae C42:4	380	-1.26E-01	5.67E-02	2.69E-02	1.00E+00	0.013
PCae C42:5	380	-6.86E-02	3.26E-02	3.60E-02	1.00E+00	0.011
PCae C42:6	380	-1.18E-01	4.91E-02	1.68E-02	1.00E+00	0.015
SM C32:2	308	2.55E-01	1.14E-01	2.64E-02	1.00E+00	0.016

SM C35	379	-8.63E-02	4.80E-02	7.26E-02	1.00E+00	0.008
SM C35:1	380	-2.15E-02	1.26E-02	8.87E-02	1.00E+00	0.007
SM C36	379	-1.80E-02	1.67E-02	2.82E-01	1.00E+00	0.003
SM C36:1	380	-3.97E-03	2.04E-03	5.19E-02	1.00E+00	0.010
SM C36:2	380	-3.97E-03	3.87E-03	3.05E-01	1.00E+00	0.003
SM C37:1	379	-4.92E-02	2.29E-02	3.21E-02	1.00E+00	0.012
SM C38:1	380	-2.05E-03	1.56E-03	1.91E-01	1.00E+00	0.004
SM C38:2	380	-1.93E-03	2.33E-03	4.08E-01	1.00E+00	0.002
SM C38:3	380	-3.07E-02	5.88E-02	6.02E-01	1.00E+00	0.001
SM C39:1	380	-2.90E-02	1.12E-02	9.78E-03	1.00E+00	0.017
SM C39:2	378	-2.87E-02	3.48E-02	4.10E-01	1.00E+00	0.002
SM C39:5	380	-4.07E-02	6.06E-02	5.02E-01	1.00E+00	0.001
SM C40:1	377	-3.22E-03	1.65E-03	5.17E-02	1.00E+00	0.010
SM C40:2	380	-1.61E-03	2.36E-03	4.96E-01	1.00E+00	0.001
SM C40:3	377	-5.58E-03	4.94E-03	2.59E-01	1.00E+00	0.003
SM C40:4	380	-7.50E-03	6.22E-03	2.29E-01	1.00E+00	0.004
SM C41:1	380	-1.61E-02	5.69E-03	4.99E-03	1.00E+00	0.020
SM C41:2	380	-2.13E-02	8.77E-03	1.56E-02	1.00E+00	0.015
SM C41:3	373	-4.03E-03	3.93E-02	9.18E-01	1.00E+00	0.000
SM C42:1	379	-5.10E-03	2.92E-03	8.11E-02	1.00E+00	0.008
SM C42:2	380	-2.09E-03	1.43E-03	1.46E-01	1.00E+00	0.005
SM C42:3	380	-1.17E-03	3.42E-03	7.32E-01	1.00E+00	0.000
SM C42:4	379	-1.33E-02	8.74E-03	1.30E-01	1.00E+00	0.006
SM C42:6	380	-2.28E-02	1.81E-02	2.11E-01	1.00E+00	0.004
SM C43	378	-1.46E-01	8.38E-02	8.26E-02	1.00E+00	0.008
SM C43:1	380	-5.99E-02	3.53E-02	9.09E-02	1.00E+00	0.007
SM C43:2	380	-4.61E-02	2.28E-02	4.37E-02	1.00E+00	0.011
SM C43:3	378	-2.22E-01	9.52E-02	2.00E-02	1.00E+00	0.014
SM C44:2	376	-1.47E-01	1.22E-01	2.26E-01	1.00E+00	0.004
SM C44:6	378	-1.17E-02	3.86E-02	7.62E-01	1.00E+00	0.000
NEFA 10:0	307	-9.57E-02	1.81E-02	2.30E-07	4.67E-05	0.083
NEFA 12:0	377	-1.61E-02	3.73E-03	1.92E-05	3.89E-03	0.047
NEFA 12:1	374	-7.80E-02	1.73E-02	8.81E-06	1.79E-03	0.051
NEFA 14:0	379	-1.03E-02	2.16E-03	2.70E-06	5.49E-04	0.056
NEFA 14:1	378	-4.54E-02	1.36E-02	8.92E-04	1.81E-01	0.028
NEFA 15:0	374	-4.24E-02	1.06E-02	7.75E-05	1.57E-02	0.041
NEFA 15:1	317	-7.25E-01	1.50E-01	2.04E-06	4.15E-04	0.067

NEFA 16:0	380	-1.40E-03	2.84E-04	1.18E-06	2.40E-04	0.060
NEFA 16:1	379	-6.09E-03	1.15E-03	2.01E-07	4.07E-05	0.068
NEFA 16:2	376	-2.71E-01	1.14E-01	1.76E-02	1.00E+00	0.015
NEFA 17:0	377	-3.79E-02	1.13E-02	8.42E-04	1.71E-01	0.029
NEFA 17:1	377	-6.77E-02	1.66E-02	5.43E-05	1.10E-02	0.042
NEFA 18:0	376	-2.78E-03	7.64E-04	3.07E-04	6.24E-02	0.034
NEFA 18:1	380	-8.47E-04	1.90E-04	1.14E-05	2.30E-03	0.049
NEFA 18:2	379	-3.10E-03	7.34E-04	3.02E-05	6.13E-03	0.044
NEFA 18:3	379	-2.18E-02	5.79E-03	1.95E-04	3.97E-02	0.036
NEFA 19:1	380	-1.53E-01	3.64E-02	3.49E-05	7.09E-03	0.044
NEFA 20:0	340	-2.07E-01	6.31E-02	1.17E-03	2.37E-01	0.030
NEFA 20:2	331	-8.99E-02	2.96E-02	2.57E-03	5.21E-01	0.027
NEFA 20:3	266	-7.89E-02	3.77E-02	3.75E-02	1.00E+00	0.016
NEFA 20:4	380	-3.40E-02	1.29E-02	8.79E-03	1.00E+00	0.018
NEFA 20:5	365	-2.04E-01	8.47E-02	1.63E-02	1.00E+00	0.015
NEFA 22:4	380	-2.24E-01	6.78E-02	1.07E-03	2.17E-01	0.027
NEFA 22:5	380	-1.15E-01	3.47E-02	1.03E-03	2.08E-01	0.028
NEFA 22:6	380	-4.29E-02	1.96E-02	2.92E-02	1.00E+00	0.012
NEFA 24:0	377	-2.36E-01	2.15E-01	2.73E-01	1.00E+00	0.003
NEFA 24:2	336	-6.72E-01	9.09E-01	4.60E-01	1.00E+00	0.002
NEFA 24:4	374	-2.71E+00	1.08E+00	1.26E-02	1.00E+00	0.016
NEFA 24:5	379	-1.12E+00	1.03E+00	2.77E-01	1.00E+00	0.003
NEFA 26:1	380	4.58E-01	3.88E-01	2.39E-01	1.00E+00	0.004

Sum AA	314	1.79E-04	4.45E-05	7.39E-05	1.50E-02	0.048
Sum Carn	361	5.03E-03	2.25E-03	2.59E-02	1.00E+00	0.013
Sum LPC	376	1.53E-03	6.33E-04	1.64E-02	1.00E+00	0.015
Sum PCaa	377	-8.11E-05	6.17E-05	1.89E-01	1.00E+00	0.004
Sum PCae	377	-9.51E-04	5.33E-04	7.54E-02	1.00E+00	0.008
Sum SM	307	-1.79E-03	9.15E-04	5.11E-02	1.00E+00	0.012
Sum NEFA	255	-0.000363934	0.00010808	0.000878428	0.17832087	0.041288364

GINI/LISA 10 years

Metabolite	N	Estimate	Std..Error	P	P.adjusted	PartialRsquared
Ala	237	1.76E-03	5.66E-04	2.04E-03	4.97E-01	0.038
Arg	237	9.34E-04	2.73E-03	7.33E-01	1.00E+00	0.000
Asn	237	6.65E-04	4.86E-03	8.91E-01	1.00E+00	0.000
Asp	236	2.44E-03	5.22E-03	6.40E-01	1.00E+00	0.001
Cit	237	-2.10E-03	6.45E-03	7.45E-01	1.00E+00	0.000
Gln	209	3.70E-04	5.01E-04	4.61E-01	1.00E+00	0.002
Glu	237	7.71E-04	1.09E-03	4.81E-01	1.00E+00	0.002
Gly	237	6.70E-04	9.10E-04	4.62E-01	1.00E+00	0.002
His	237	-3.65E-04	2.65E-03	8.91E-01	1.00E+00	0.000
Ile	237	7.43E-03	4.08E-03	7.02E-02	1.00E+00	0.013
Leu	237	2.68E-03	2.21E-03	2.27E-01	1.00E+00	0.006
Lys	237	8.75E-04	1.51E-03	5.64E-01	1.00E+00	0.001
Met	237	9.75E-03	1.17E-02	4.05E-01	1.00E+00	0.003
Orn	237	9.05E-04	2.04E-03	6.58E-01	1.00E+00	0.001
Phe	237	6.66E-03	3.45E-03	5.45E-02	1.00E+00	0.015
Pro	237	3.20E-03	8.86E-04	3.76E-04	9.13E-02	0.050
Ser	237	-1.15E-04	1.88E-03	9.51E-01	1.00E+00	0.000
Thr	237	1.67E-03	1.88E-03	3.75E-01	1.00E+00	0.003
Trp	237	9.57E-03	4.64E-03	4.05E-02	1.00E+00	0.017
Tyr	237	1.13E-02	3.47E-03	1.29E-03	3.14E-01	0.041
Val	237	2.52E-03	1.14E-03	2.83E-02	1.00E+00	0.019
H1	239	6.62E-05	6.79E-05	3.31E-01	1.00E+00	0.004
Carn	181	1.98E-02	1.16E-02	9.01E-02	1.00E+00	0.014
Carn C2	183	-9.48E-02	3.39E-02	5.76E-03	1.00E+00	0.038
Carn C3	195	6.37E-01	7.66E-01	4.07E-01	1.00E+00	0.003
Carn C4	153	5.53E-01	8.26E-01	5.04E-01	1.00E+00	0.003
Carn C5	143	2.70E+00	2.16E+00	2.13E-01	1.00E+00	0.010
Carn C6	238	-4.55E+00	1.37E+00	1.05E-03	2.55E-01	0.042
Carn C8	239	-6.05E-01	1.84E-01	1.18E-03	2.87E-01	0.041
Carn C8:1	238	-7.29E-01	9.07E-01	4.22E-01	1.00E+00	0.003
Carn C9	220	8.07E-01	2.26E+00	7.21E-01	1.00E+00	0.001

Carn C10	238	-5.66E-01	1.43E-01	1.06E-04	2.59E-02	0.058
Carn C10:1	239	-9.28E-01	2.34E-01	9.94E-05	2.42E-02	0.059
Carn C10:2	146	-3.50E+00	1.22E+00	4.72E-03	1.00E+00	0.053
Carn C12	238	-1.36E+00	3.30E-01	5.54E-05	1.35E-02	0.063
Carn C12:1	211	-6.33E-01	1.56E-01	6.79E-05	1.65E-02	0.068
Carn C14	153	-9.72E+00	2.95E+00	1.22E-03	2.97E-01	0.062
Carn C14:1	238	-2.73E+00	4.91E-01	7.38E-08	1.79E-05	0.107
Carn C14:2	155	-6.42E+00	2.00E+00	1.66E-03	4.04E-01	0.060
Carn C16	217	-7.06E+00	2.33E+00	2.78E-03	6.76E-01	0.039
Carn C16:1	188	-9.86E+00	3.18E+00	2.20E-03	5.34E-01	0.047
Carn C18	180	-5.70E+00	2.72E+00	3.76E-02	1.00E+00	0.022
Carn C18:1	239	-3.95E+00	1.37E+00	4.44E-03	1.00E+00	0.032
Carn C18:2	169	-6.14E+00	2.89E+00	3.52E-02	1.00E+00	0.025

LPC C14	239	1.53E-01	6.42E-02	1.78E-02	1.00E+00	0.022
LPC C15	222	1.06E-01	1.68E-01	5.27E-01	1.00E+00	0.002
LPC C16	239	2.03E-03	2.49E-03	4.17E-01	1.00E+00	0.003
LPC C16:1	239	1.43E-01	7.33E-02	5.27E-02	1.00E+00	0.015
LPC C17	239	1.78E-03	1.09E-01	9.87E-01	1.00E+00	0.000
LPC C17:1	222	2.25E-01	4.99E-01	6.52E-01	1.00E+00	0.001
LPC C18	239	6.82E-03	7.08E-03	3.37E-01	1.00E+00	0.004
LPC C18:1	239	-3.42E-04	1.37E-02	9.80E-01	1.00E+00	0.000
LPC C18:2	239	-3.46E-03	6.99E-03	6.21E-01	1.00E+00	0.001
LPC C18:3	222	2.69E-01	2.40E-01	2.63E-01	1.00E+00	0.005
LPC C18:6	144	-1.16E-01	6.70E-01	8.62E-01	1.00E+00	0.000
LPC C20:2	128	1.80E-01	5.15E-01	7.28E-01	1.00E+00	0.001
LPC C20:3	239	1.50E-01	9.40E-02	1.11E-01	1.00E+00	0.010
LPC C20:4	239	2.26E-03	3.30E-02	9.45E-01	1.00E+00	0.000
LPC C20:5	237	3.40E-01	2.90E-01	2.41E-01	1.00E+00	0.006
LPC C22:5	177	-1.54E-01	4.15E-01	7.10E-01	1.00E+00	0.001

LPC C22:6	222	-6.32E-02	1.04E-01	5.45E-01	1.00E+00	0.002
PCaa C30	239	5.08E-02	4.58E-02	2.68E-01	1.00E+00	0.005
PCaa C30:2	166	-4.97E-01	3.13E-01	1.14E-01	1.00E+00	0.014
PCaa C30:3	164	-8.41E-01	8.76E-01	3.38E-01	1.00E+00	0.005
PCaa C30:4	118	-1.91E+00	1.45E+00	1.91E-01	1.00E+00	0.014
PCaa C32	239	-1.90E-02	2.06E-02	3.57E-01	1.00E+00	0.003
PCaa C32:1	239	3.09E-02	1.05E-02	3.62E-03	8.79E-01	0.033
PCaa C32:2	239	6.48E-02	4.84E-02	1.81E-01	1.00E+00	0.007
PCaa C32:3	187	-9.38E-02	3.47E-01	7.87E-01	1.00E+00	0.000
PCaa C34:1	239	-2.53E-04	1.35E-03	8.51E-01	1.00E+00	0.000
PCaa C34:2	239	-5.29E-04	7.86E-04	5.01E-01	1.00E+00	0.002
PCaa C34:3	239	2.22E-02	1.29E-02	8.60E-02	1.00E+00	0.012
PCaa C34:4	239	1.63E-01	8.65E-02	6.13E-02	1.00E+00	0.014
PCaa C34:5	158	5.21E-01	8.19E-01	5.26E-01	1.00E+00	0.002
PCaa C36	186	-4.11E-02	3.48E-02	2.39E-01	1.00E+00	0.007
PCaa C36:1	239	1.29E-02	4.89E-03	8.71E-03	1.00E+00	0.027
PCaa C36:2	239	7.34E-04	1.07E-03	4.92E-01	1.00E+00	0.002
PCaa C36:3	239	2.61E-03	2.16E-03	2.26E-01	1.00E+00	0.006
PCaa C36:4	239	5.34E-04	1.69E-03	7.53E-01	1.00E+00	0.000
PCaa C36:5	238	1.38E-02	1.15E-02	2.32E-01	1.00E+00	0.006
PCaa C36:6	218	8.54E-02	1.39E-01	5.41E-01	1.00E+00	0.002
PCaa C38	239	-8.64E-02	5.54E-02	1.20E-01	1.00E+00	0.010
PCaa C38:2	110	8.91E-03	5.21E-02	8.65E-01	1.00E+00	0.000
PCaa C38:3	239	1.51E-02	4.66E-03	1.39E-03	3.37E-01	0.040
PCaa C38:4	239	1.52E-03	2.21E-03	4.93E-01	1.00E+00	0.002
PCaa C38:5	239	7.37E-04	4.84E-03	8.79E-01	1.00E+00	0.000
PCaa C38:6	239	-2.05E-03	3.05E-03	5.01E-01	1.00E+00	0.002
PCaa C40	174	-1.22E-01	1.61E-01	4.49E-01	1.00E+00	0.003
PCaa C40:1	188	-1.22E-01	1.22E-01	3.21E-01	1.00E+00	0.005
PCaa C40:3	131	-1.24E-01	1.12E-01	2.73E-01	1.00E+00	0.009
PCaa C40:4	239	6.93E-02	4.52E-02	1.27E-01	1.00E+00	0.009
PCaa C40:5	239	2.27E-02	1.79E-02	2.06E-01	1.00E+00	0.006
PCaa C40:6	239	8.17E-03	7.52E-03	2.78E-01	1.00E+00	0.005
PCaa C42	226	-4.59E-01	2.16E-01	3.49E-02	1.00E+00	0.019
PCaa C42:1	116	-3.32E-01	4.16E-01	4.26E-01	1.00E+00	0.005
PCaa C42:4	114	1.77E-01	3.35E-01	5.98E-01	1.00E+00	0.002
PCaa C42:5	150	-1.42E-01	3.45E-01	6.82E-01	1.00E+00	0.001
PCaa C42:6	127	6.72E-05	2.53E-01	1.00E+00	1.00E+00	0.000

PCaa C43:6	238	-4.75E-01	1.72E-01	6.29E-03	1.00E+00	0.030
PCaa C44:12	211	-5.42E-01	2.25E-01	1.70E-02	1.00E+00	0.026
PCae C32	239	-1.33E-01	7.48E-02	7.65E-02	1.00E+00	0.013
PCae C32:1	239	-1.55E-01	6.99E-02	2.78E-02	1.00E+00	0.019
PCae C32:2	204	-5.51E-01	1.83E-01	2.97E-03	7.22E-01	0.041
PCae C34	219	-8.13E-02	1.38E-01	5.57E-01	1.00E+00	0.002
PCae C34:1	239	-2.87E-02	2.77E-02	3.00E-01	1.00E+00	0.004
PCae C34:2	239	-2.62E-02	2.09E-02	2.10E-01	1.00E+00	0.006
PCae C34:3	239	-6.63E-02	2.29E-02	4.12E-03	1.00E+00	0.033
PCae C34:4	137	-4.16E-01	6.58E-01	5.28E-01	1.00E+00	0.003
PCae C36	174	-2.70E-01	2.60E-01	3.02E-01	1.00E+00	0.006
PCae C36:1	239	-1.91E-02	1.88E-02	3.12E-01	1.00E+00	0.004
PCae C36:2	239	-9.62E-03	1.66E-02	5.62E-01	1.00E+00	0.001
PCae C36:3	239	-3.01E-02	2.85E-02	2.92E-01	1.00E+00	0.004
PCae C36:4	239	-1.39E-02	1.20E-02	2.48E-01	1.00E+00	0.005
PCae C36:5	239	-2.90E-02	1.74E-02	9.62E-02	1.00E+00	0.011
PCae C36:6	121	-3.56E-01	4.60E-01	4.40E-01	1.00E+00	0.005
PCae C38	238	-1.27E-02	6.37E-02	8.42E-01	1.00E+00	0.000
PCae C38:2	239	-1.26E-02	3.07E-02	6.82E-01	1.00E+00	0.001
PCae C38:3	239	-8.61E-03	2.10E-02	6.82E-01	1.00E+00	0.001
PCae C38:4	239	-2.60E-02	1.64E-02	1.15E-01	1.00E+00	0.010
PCae C38:5	239	-1.85E-02	1.38E-02	1.82E-01	1.00E+00	0.007
PCae C38:6	239	-3.60E-02	2.70E-02	1.85E-01	1.00E+00	0.007
PCae C40	211	-1.75E-03	2.09E-02	9.34E-01	1.00E+00	0.000
PCae C40:1	222	-2.94E-02	7.44E-02	6.94E-01	1.00E+00	0.001
PCae C40:2	162	-4.22E-02	7.38E-02	5.68E-01	1.00E+00	0.002
PCae C40:3	179	2.97E-04	3.75E-02	9.94E-01	1.00E+00	0.000
PCae C40:4	211	-6.99E-02	5.56E-02	2.10E-01	1.00E+00	0.007
PCae C40:5	239	-7.53E-02	3.89E-02	5.44E-02	1.00E+00	0.015
PCae C40:6	239	-7.01E-02	4.70E-02	1.37E-01	1.00E+00	0.009
PCae C42	187	-8.64E-02	1.94E-01	6.56E-01	1.00E+00	0.001
PCae C42:1	162	-7.91E-03	1.21E-01	9.48E-01	1.00E+00	0.000
PCae C42:2	194	-2.53E-01	1.73E-01	1.47E-01	1.00E+00	0.010
PCae C42:3	200	-1.16E-01	1.06E-01	2.78E-01	1.00E+00	0.006
PCae C42:4	180	-2.86E-01	2.02E-01	1.59E-01	1.00E+00	0.010
PCae C42:5	221	-1.77E-01	7.58E-02	2.04E-02	1.00E+00	0.023
PCae C42:6	239	-3.00E-01	1.33E-01	2.49E-02	1.00E+00	0.020
SM C30:1	199	4.87E-01	3.75E-01	1.96E-01	1.00E+00	0.008
SM C31:1	116	-3.36E-01	9.08E-01	7.12E-01	1.00E+00	0.001
SM C32:1	239	-6.40E-03	2.67E-02	8.11E-01	1.00E+00	0.000
SM C32:2	239	5.78E-01	2.52E-01	2.29E-02	1.00E+00	0.021
SM C33:1	239	-3.94E-02	4.24E-02	3.53E-01	1.00E+00	0.003
SM C33:2	175	1.43E-01	1.06E+00	8.93E-01	1.00E+00	0.000
SM C33:3	172	-3.20E+00	1.76E+00	7.12E-02	1.00E+00	0.018
SM C34:1	239	-8.06E-03	2.75E-03	3.77E-03	9.16E-01	0.033

SM C34:2	239	-1.69E-02	1.88E-02	3.67E-01	1.00E+00	0.003
SM C35	159	-4.29E-01	3.96E-01	2.80E-01	1.00E+00	0.007
SM C35:1	239	-6.64E-02	8.47E-02	4.34E-01	1.00E+00	0.002
SM C35:2	193	-6.93E-01	5.03E-01	1.70E-01	1.00E+00	0.009
SM C36:1	239	-1.94E-02	1.31E-02	1.39E-01	1.00E+00	0.009
SM C36:2	239	-1.89E-02	2.39E-02	4.30E-01	1.00E+00	0.002
SM C36:3	239	-2.83E-01	2.87E-01	3.26E-01	1.00E+00	0.004
SM C37:1	117	-3.02E-01	1.42E-01	3.52E-02	1.00E+00	0.032
SM C37:3	163	-1.25E+00	5.26E-01	1.84E-02	1.00E+00	0.031
SM C38:1	184	1.12E-02	7.31E-03	1.27E-01	1.00E+00	0.012
SM C38:2	239	-1.25E-02	1.49E-02	4.03E-01	1.00E+00	0.003
SM C38:3	142	7.65E-03	4.94E-01	9.88E-01	1.00E+00	0.000
SM C39:1	239	9.06E-04	3.64E-02	9.80E-01	1.00E+00	0.000
SM C39:2	142	-3.31E-02	1.45E-01	8.20E-01	1.00E+00	0.000
SM C39:5	113	-4.78E-01	4.52E-01	2.92E-01	1.00E+00	0.009
SM C40:2	239	4.77E-03	8.23E-03	5.63E-01	1.00E+00	0.001
SM C40:4	227	-4.17E-02	1.15E-01	7.16E-01	1.00E+00	0.001
SM C40:5	205	2.03E-01	2.46E-01	4.11E-01	1.00E+00	0.003
SM C41:1	239	-3.67E-02	2.01E-02	6.84E-02	1.00E+00	0.013
SM C41:2	239	-3.23E-02	2.84E-02	2.58E-01	1.00E+00	0.005
SM C42:1	239	-2.64E-02	1.20E-02	2.92E-02	1.00E+00	0.019
SM C42:2	239	-1.62E-02	6.80E-03	1.77E-02	1.00E+00	0.022
SM C42:3	222	-1.47E-02	1.07E-02	1.68E-01	1.00E+00	0.008
SM C42:4	203	1.31E-02	2.98E-02	6.61E-01	1.00E+00	0.001
SM C42:6	227	-8.44E-02	7.36E-02	2.53E-01	1.00E+00	0.005
SM C43	136	1.13E-01	4.16E-01	7.85E-01	1.00E+00	0.001
SM C43:1	237	-3.86E-01	1.59E-01	1.60E-02	1.00E+00	0.023
SM C43:2	239	-6.59E-02	9.66E-02	4.96E-01	1.00E+00	0.002
SM C44:6	198	-3.38E-02	1.40E-01	8.10E-01	1.00E+00	0.000
NEFA 10:0	227	-1.15E-01	6.22E-02	6.47E-02	1.00E+00	0.014
NEFA 12:0	199	-3.05E-02	1.05E-02	3.99E-03	9.70E-01	0.038
NEFA 12:1	141	-1.64E-01	8.08E-02	4.45E-02	1.00E+00	0.027
NEFA 13:0	96	-3.09E-01	2.11E-01	1.46E-01	1.00E+00	0.019
NEFA 13:1	186	-1.13E+00	8.87E-01	2.06E-01	1.00E+00	0.008
NEFA 14:0	238	-2.41E-02	8.68E-03	5.86E-03	1.00E+00	0.030
NEFA 14:1	238	-1.10E-01	5.30E-02	3.88E-02	1.00E+00	0.017
NEFA 14:2	110	-1.84E-02	1.40E-02	1.93E-01	1.00E+00	0.014

NEFA 16:0	239	-4.25E-03	1.39E-03	2.50E-03	6.08E-01	0.035
NEFA 16:1	211	-1.42E-02	4.13E-03	6.99E-04	1.70E-01	0.049
NEFA 16:2	216	-2.65E-01	2.30E-01	2.50E-01	1.00E+00	0.006
NEFA 17:0	222	-1.22E-01	4.70E-02	1.02E-02	1.00E+00	0.028
NEFA 17:1	211	-2.35E-01	6.80E-02	6.67E-04	1.62E-01	0.050
NEFA 17:2	141	-5.47E+00	1.57E+00	6.56E-04	1.59E-01	0.072
NEFA 18:0	152	-6.00E-03	3.76E-03	1.13E-01	1.00E+00	0.016
NEFA 18:1	239	-3.42E-03	9.86E-04	6.21E-04	1.51E-01	0.045
NEFA 18:2	239	-9.04E-03	2.82E-03	1.53E-03	3.71E-01	0.039
NEFA 18:3	239	-2.03E-02	8.71E-03	2.06E-02	1.00E+00	0.021
NEFA 18:4	223	-5.20E-01	5.36E-01	3.33E-01	1.00E+00	0.004
NEFA 19:0	223	-1.08E+00	3.81E-01	4.95E-03	1.00E+00	0.032
NEFA 19:1	226	-2.58E-01	1.22E-01	3.58E-02	1.00E+00	0.018
NEFA 20:0	128	-6.11E-01	2.92E-01	3.86E-02	1.00E+00	0.032
NEFA 20:1	239	-1.80E-01	4.67E-02	1.46E-04	3.54E-02	0.055
NEFA 20:2	190	-1.71E-01	7.74E-02	2.81E-02	1.00E+00	0.024
NEFA 20:3	217	-6.86E-02	6.36E-02	2.82E-01	1.00E+00	0.005
NEFA 20:4	211	-8.69E-02	3.10E-02	5.53E-03	1.00E+00	0.034
NEFA 20:5	175	-1.13E-01	1.16E-01	3.31E-01	1.00E+00	0.005
NEFA 22:0	86	3.55E-01	5.55E-01	5.23E-01	1.00E+00	0.005
NEFA 22:1	236	-6.74E-01	3.19E-01	3.56E-02	1.00E+00	0.018
NEFA 22:2	131	-5.52E+00	2.23E+00	1.44E-02	1.00E+00	0.040
NEFA 22:3	211	-3.47E+00	1.45E+00	1.80E-02	1.00E+00	0.025
NEFA 22:4	211	-3.48E-01	1.65E-01	3.60E-02	1.00E+00	0.019
NEFA 22:5	239	-2.02E-01	8.75E-02	2.20E-02	1.00E+00	0.021
NEFA 22:6	239	-6.92E-02	3.73E-02	6.50E-02	1.00E+00	0.013
NEFA 24:0	195	-1.23E+00	6.61E-01	6.37E-02	1.00E+00	0.016
NEFA 24:1	205	-1.31E+00	5.03E-01	9.72E-03	1.00E+00	0.030
NEFA 24:2	153	-1.24E+01	4.10E+00	2.84E-03	6.91E-01	0.054
NEFA 24:3	144	-2.81E+01	1.27E+01	2.85E-02	1.00E+00	0.030
NEFA 24:4	211	-8.85E+00	3.18E+00	5.93E-03	1.00E+00	0.033
NEFA 24:5	223	-3.49E+00	2.11E+00	1.01E-01	1.00E+00	0.011
NEFA 24:6	182	-3.97E+00	3.37E+00	2.40E-01	1.00E+00	0.007
NEFA 26:0	153	2.37E+00	2.70E+00	3.81E-01	1.00E+00	0.005
NEFA 26:1	194	-7.82E+00	2.46E+00	1.69E-03	4.11E-01	0.046
NEFA 26:2	186	-6.01E+00	3.48E+00	8.58E-02	1.00E+00	0.015
NEFA 26:3	158	-5.89E+00	3.83E+00	1.26E-01	1.00E+00	0.014
NEFA 26:4	194	-1.54E+01	1.27E+01	2.27E-01	1.00E+00	0.007
NEFA 26:5	183	-2.93E+01	1.81E+01	1.07E-01	1.00E+00	0.013
NEFA 26:6	127	-1.66E+01	8.17E+00	4.41E-02	1.00E+00	0.028
Pyruvate	239	2.09E-03	1.01E-03	3.96E-02	1.00E+00	0.017
Lactate	239	6.30E-05	5.43E-05	2.47E-01	1.00E+00	0.005
Acetoacetate	236	-1.69E-02	5.33E-03	1.76E-03	4.27E-01	0.039
Alpha-ketobutyrate	222	-2.48E-02	1.76E-02	1.60E-01	1.00E+00	0.008
Fumarate	239	-1.70E-01	1.93E-01	3.79E-01	1.00E+00	0.003
3-Methyl-2-oxobutanoate	239	-4.05E-03	1.98E-02	8.38E-01	1.00E+00	0.000
Succininate	239	-2.44E-02	2.74E-02	3.74E-01	1.00E+00	0.003

Methylmalonate	239	-1.96E-01	3.00E-01	5.14E-01	1.00E+00	0.002
Taurine	239	2.21E-03	2.44E-03	3.64E-01	1.00E+00	0.003
3-Methyl-2-oxovalerate	239	-6.99E-03	1.83E-02	7.03E-01	1.00E+00	0.001
4-Methyl-2-oxovalerate	239	-1.02E-02	1.09E-02	3.52E-01	1.00E+00	0.003
Malate	239	-9.49E-02	1.10E-01	3.89E-01	1.00E+00	0.003
Alpha-ketoglutarate	239	1.18E-01	4.45E-02	8.37E-03	1.00E+00	0.028
Alpha-aminoadipate	239	1.49E+00	5.69E-01	9.42E-03	1.00E+00	0.027
Isocitrate	226	-7.73E-01	3.29E-01	1.97E-02	1.00E+00	0.023
Citrate	239	-1.25E-02	7.32E-03	8.96E-02	1.00E+00	0.011
Sum AA	208	2.68E-04	1.20E-04	2.71E-02	1.00E+00	0.022
Sum Carn	127	3.58E-03	1.12E-02	7.49E-01	1.00E+00	0.001
Sum LPC	220	8.26E-04	1.40E-03	5.54E-01	1.00E+00	0.002
Sum PCaa	238	9.26E-05	2.06E-04	6.54E-01	1.00E+00	0.001
Sum PCae	210	-2.19E-03	1.83E-03	2.31E-01	1.00E+00	0.007
Sum SM	239	-5.04E-03	2.49E-03	4.43E-02	1.00E+00	0.016
Sum NEFA	77	-2.18E-03	7.82E-04	6.74E-03	1.000	0.088

Meta-analyses

Metabolite	N	Estimate	Std..Error	P	P.adjusted	PartialRsquared	Q	Qp
Ala	967	2.20E-03	2.51E-04	8.49E-18	9.77E-16	0.042	24	<0.0001
Arg	966	2.73E-03	9.64E-04	4.73E-03	5.44E-01	0.005	19	<0.0001
Asn	931	6.34E-03	2.16E-03	3.40E-03	3.90E-01	0.005	11.9	0.003
Asp	931	-9.52E-04	3.20E-03	7.67E-01	1.00E+00	0.000	36.7	<0.0001
Cit	968	-1.50E-04	2.58E-03	9.54E-01	1.00E+00	0.000	3.15	0.21
Gln	938	7.22E-04	1.72E-04	3.01E-05	3.46E-03	0.010	12.8	0.002
Glu	968	-1.56E-03	3.46E-04	6.88E-06	7.91E-04	0.012	21.1	<0.0001
Gly	966	1.06E-03	4.12E-04	1.01E-02	1.00E+00	0.004	0.706	0.7
His	968	6.30E-04	9.89E-04	5.25E-01	1.00E+00	0.000	0.18	0.91
Ile	929	3.28E-03	1.87E-03	8.01E-02	1.00E+00	0.002	2.8	0.25
Leu	968	1.33E-03	1.00E-03	1.87E-01	1.00E+00	0.001	1.13	0.57
Lys	967	1.69E-03	7.24E-04	1.98E-02	1.00E+00	0.003	4.13	0.13
Met	968	1.35E-02	4.39E-03	2.17E-03	2.49E-01	0.006	2.95	0.23
Orn	967	1.39E-03	1.09E-03	2.02E-01	1.00E+00	0.001	0.405	0.82
Phe	967	4.99E-03	1.75E-03	4.38E-03	5.04E-01	0.005	1.47	0.48
Pro	964	1.67E-03	3.48E-04	2.00E-06	2.31E-04	0.014	6.1	0.047
Ser	966	-3.35E-04	6.79E-04	6.22E-01	1.00E+00	0.000	0.951	0.62
Thr	968	3.42E-03	7.89E-04	1.63E-05	1.88E-03	0.011	10.1	0.006
Trp	967	6.76E-03	1.83E-03	2.25E-04	2.59E-02	0.008	3.69	0.16
Tyr	966	1.16E-02	1.44E-03	1.64E-15	1.88E-13	0.036	18.1	<0.0001
Val	968	1.59E-03	4.92E-04	1.28E-03	1.48E-01	0.006	3.42	0.18
H1	968	1.66E-04	3.18E-05	2.16E-07	2.48E-05	0.016	8.54	0.014
Carn	911	9.07E-03	1.62E-03	2.91E-08	3.34E-06	0.021	1.65	0.44
Carn C2	913	-5.41E-02	8.71E-03	8.01E-10	9.21E-08	0.023	5.81	0.055
Carn C3	923	-9.49E-03	1.03E-01	9.27E-01	1.00E+00	0.000	4.74	0.09
Carn C5	868	-4.70E-01	3.60E-01	1.92E-01	1.00E+00	0.001	3.87	0.14
Carn C8:1	965	-3.65E-02	2.02E-01	8.57E-01	1.00E+00	0.000	2.55	0.28

Carn C10:1	967	-5.09E-01	1.13E-01	7.77E-06	8.94E-04	0.012	10	0.007
Carn C12:1	942	-5.66E-01	7.81E-02	9.07E-13	1.04E-10	0.030	0.529	0.77
Carn C14:1	967	-1.28E+00	1.60E-01	3.07E-15	3.53E-13	0.036	16.2	<0.0001
Carn C16	946	-4.17E+00	6.64E-01	5.18E-10	5.96E-08	0.024	19.1	<0.0001
Carn C18	907	-4.67E+00	1.17E+00	7.60E-05	8.74E-03	0.010	5.23	0.07
Carn C18:1	970	-3.18E+00	4.89E-01	1.29E-10	1.48E-08	0.027	21.4	<0.0001
LPC C16	970	-3.07E-03	7.20E-04	2.28E-05	2.62E-03	0.011	34.8	<0.0001
LPC C16:1	969	-2.67E-02	2.45E-02	2.76E-01	1.00E+00	0.001	19.6	<0.0001
LPC C17	907	-1.80E-01	3.71E-02	1.54E-06	1.77E-04	0.015	9.94	0.007
LPC C18	970	-7.37E-03	1.99E-03	2.22E-04	2.56E-02	0.008	32.5	<0.0001
LPC C18:1	970	-1.58E-02	4.20E-03	1.83E-04	2.10E-02	0.008	26.3	<0.0001
LPC C18:2	970	3.22E-03	2.23E-03	1.49E-01	1.00E+00	0.001	2.14	0.34
LPC C18:3	821	1.75E-01	9.79E-02	7.38E-02	1.00E+00	0.002	0.442	0.8
LPC C20:3	902	3.85E-02	3.24E-02	2.36E-01	1.00E+00	0.001	1.91	0.38
LPC C20:4	969	-3.12E-03	1.18E-02	7.92E-01	1.00E+00	0.000	0.775	0.68
LPC C20:5	782	6.37E-02	1.10E-01	5.61E-01	1.00E+00	0.000	2.2	0.33

LPC C22:6	853	-1.53E-01	4.23E-02	3.01E-04	3.46E-02	0.009	8.36	0.015
PCaa C32	970	-5.95E-03	4.21E-03	1.57E-01	1.00E+00	0.001	0.631	0.73
PCaa C32:1	907	1.31E-02	3.64E-03	3.60E-04	4.14E-02	0.008	10.1	0.006
PCaa C34:1	969	-5.68E-04	4.29E-04	1.86E-01	1.00E+00	0.001	0.318	0.85
PCaa C34:2	903	1.81E-04	2.61E-04	4.88E-01	1.00E+00	0.000	9.96	0.007
PCaa C34:3	970	1.22E-02	4.57E-03	7.46E-03	8.57E-01	0.004	3.94	0.14
PCaa C34:4	921	1.35E-01	3.08E-02	1.36E-05	1.57E-03	0.012	13.6	0.001
PCaa C36:2	969	1.18E-03	3.49E-04	7.36E-04	8.46E-02	0.007	5.78	0.06
PCaa C36:3	970	1.44E-03	8.54E-04	9.22E-02	1.00E+00	0.002	3.64	0.16
PCaa C36:4	904	6.38E-04	6.37E-04	3.17E-01	1.00E+00	0.001	6.71	0.035
PCaa C36:5	900	5.70E-03	3.91E-03	1.45E-01	1.00E+00	0.001	1.13	0.57
PCaa C38	784	-6.62E-02	2.17E-02	2.33E-03	2.68E-01	0.007	0.928	0.63
PCaa C38:3	781	7.52E-03	2.25E-03	8.86E-04	1.02E-01	0.008	7.29	0.026
PCaa C38:4	803	7.11E-04	8.66E-04	4.12E-01	1.00E+00	0.000	3.18	0.2
PCaa C38:5	858	-2.89E-03	1.91E-03	1.29E-01	1.00E+00	0.002	2.3	0.32
PCaa C38:6	970	-1.77E-03	1.14E-03	1.19E-01	1.00E+00	0.001	0.0267	0.99
PCaa C40:4	774	2.16E-02	1.83E-02	2.40E-01	1.00E+00	0.001	2.95	0.23
PCaa C40:5	790	5.21E-03	7.61E-03	4.94E-01	1.00E+00	0.000	3.16	0.21
PCaa C40:6	969	3.36E-03	2.97E-03	2.57E-01	1.00E+00	0.001	0.984	0.61

PCae C34:1	970	-1.58E-02	8.28E-03	5.64E-02	1.00E+00	0.002	0.519	0.77
PCae C34:2	903	4.01E-03	6.31E-03	5.25E-01	1.00E+00	0.000	10.6	0.005
PCae C34:3	970	-2.12E-02	7.21E-03	3.41E-03	3.92E-01	0.005	6.26	0.044
PCae C36:1	855	-2.44E-02	6.11E-03	6.92E-05	7.95E-03	0.011	1.71	0.42
PCae C36:2	773	-1.03E-02	7.11E-03	1.50E-01	1.00E+00	0.002	2.78	0.25
PCae C36:3	970	-1.45E-03	8.76E-03	8.69E-01	1.00E+00	0.000	2.69	0.26
PCae C36:4	902	7.95E-04	3.91E-03	8.39E-01	1.00E+00	0.000	6.08	0.048
PCae C36:5	970	-5.38E-03	5.98E-03	3.68E-01	1.00E+00	0.000	5.39	0.07
PCae C38	800	-6.16E-02	2.87E-02	3.23E-02	1.00E+00	0.003	1.24	0.54
PCae C38:3	802	-2.57E-02	7.80E-03	1.01E-03	1.16E-01	0.008	2.49	0.29
PCae C38:4	790	-1.66E-02	6.32E-03	8.66E-03	9.96E-01	0.006	0.753	0.69
PCae C38:5	906	6.01E-04	4.19E-03	8.86E-01	1.00E+00	0.000	6.27	0.044
PCae C38:6	858	-1.16E-02	9.76E-03	2.34E-01	1.00E+00	0.001	2.02	0.36
PCae C40	756	-2.09E-02	1.01E-02	3.97E-02	1.00E+00	0.004	1.79	0.41
PCae C40:4	763	-8.21E-02	2.12E-02	1.18E-04	1.35E-02	0.011	0.0918	0.96
PCae C40:6	791	-5.08E-02	2.00E-02	1.13E-02	1.00E+00	0.005	1	0.61
SM C32:2	719	2.93E-01	1.19E-01	1.37E-02	1.00E+00	0.005	3.73	0.15

SM C35:1	921	-5.78E-02	2.12E-02	6.53E-03	7.51E-01	0.005	6.46	0.04
SM C36:1	970	-1.07E-02	3.27E-03	1.11E-03	1.28E-01	0.006	6.59	0.037
SM C36:2	840	-8.84E-03	6.71E-03	1.88E-01	1.00E+00	0.001	0.755	0.69
SM C41:1	858	-2.76E-02	7.42E-03	2.16E-04	2.48E-02	0.009	1.41	0.49
SM C41:2	857	-2.62E-02	1.04E-02	1.18E-02	1.00E+00	0.004	0.141	0.93
SM C42:1	805	-1.19E-02	4.23E-03	5.18E-03	5.96E-01	0.005	3.01	0.22
SM C42:2	792	-6.71E-03	2.20E-03	2.36E-03	2.72E-01	0.007	4.96	0.08
NEFA 12:0	911	-2.31E-02	5.09E-03	6.67E-06	7.67E-04	0.013	2.61	0.27
NEFA 14:0	953	-1.90E-02	2.78E-03	1.35E-11	1.55E-09	0.027	11.8	0.003
NEFA 14:1	951	-8.11E-02	1.63E-02	7.39E-07	8.50E-05	0.015	4.91	0.09

NEFA 16:0	955	-2.80E-03	3.73E-04	1.50E-13	1.73E-11	0.032	17.2	<0.0001
NEFA 16:1	926	-1.02E-02	1.46E-03	6.98E-12	8.03E-10	0.028	8.86	0.012
NEFA 16:2	928	-3.18E-01	1.03E-01	2.04E-03	2.34E-01	0.006	0.783	0.68
NEFA 17:0	935	-9.40E-02	1.38E-02	1.78E-11	2.05E-09	0.027	18.5	<0.0001
NEFA 17:1	924	-1.48E-01	2.13E-02	6.05E-12	6.96E-10	0.028	17.2	<0.0001
NEFA 18:1	955	-1.78E-03	2.66E-04	3.63E-11	4.18E-09	0.026	16.5	<0.0001
NEFA 18:2	952	-5.53E-03	9.16E-04	2.19E-09	2.52E-07	0.021	7.35	0.025
NEFA 18:3	954	-1.73E-02	3.96E-03	1.37E-05	1.58E-03	0.012	0.502	0.78
NEFA 19:1	942	-2.59E-01	4.40E-02	5.06E-09	5.82E-07	0.020	9.06	0.011
NEFA 20:2	856	-1.46E-01	2.90E-02	6.21E-07	7.14E-05	0.017	2.68	0.26
NEFA 20:3	819	-1.29E-01	2.19E-02	5.71E-09	6.56E-07	0.026	4.61	0.1
NEFA 20:4	927	-4.78E-02	5.93E-03	2.28E-15	2.62E-13	0.037	2.59	0.27
NEFA 20:5	874	-2.96E-01	4.74E-02	6.25E-10	7.18E-08	0.024	7.44	0.024
NEFA 22:4	926	-3.53E-01	6.02E-02	6.27E-09	7.21E-07	0.020	3.97	0.14
NEFA 22:5	955	-1.94E-01	3.22E-02	2.20E-09	2.53E-07	0.022	4.77	0.09
NEFA 22:6	955	-8.48E-02	1.48E-02	1.30E-08	1.49E-06	0.020	5.82	0.055
NEFA 24:0	908	-6.46E-01	2.38E-01	6.77E-03	7.79E-01	0.005	2.61	0.27
NEFA 24:4	805	-5.37E+00	1.22E+00	1.15E-05	1.32E-03	0.014	3.79	0.15
NEFA 26:1	910	2.08E-02	4.24E-01	9.61E-01	1.00E+00	0.000	11.2	0.004

Sum AA	870	3.12E-04	5.81E-05	1.05E-07	1.21E-05	0.019	9.78	0.008
Sum Carn	835	6.40E-03	1.58E-03	5.43E-05	6.25E-03	0.012	0.363	0.83
Sum LPC	651	-6.66E-04	5.45E-04	2.22E-01	1.00E+00	0.001	20.3	<0.0001
Sum PCaa	665	-1.96E-05	9.41E-05	8.35E-01	1.00E+00	0.000	1.2	0.55
Sum PCae	638	-1.81E-03	7.85E-04	2.15E-02	1.00E+00	0.005	0.525	0.77
Sum SM	602	-3.65E-03	1.24E-03	3.37E-03	3.88E-01	0.008	1.63	0.44
Sum NEFA	547	-7.10E-04	1.42E-04	8.54E-07	9.83E-05	0.022	8.4	0.015

Supplemental Table 6 Associations of metabolites to LDL or ApoAI for each study follow-up visit were calculated in linear models adjusted for child age and sex. Statistical significance was evaluated using Bonferroni corrected p-values (p.adjust) < 0.05 (with dark grey background). AA, amino acids; Carn, Acylcarnitines; lyso.PC, lyso-phosphatidylcholine; NEFA, nonesterified fatty acid; PCaa, Diacyl-phosphatidylcholine; PCae, Alkyl-acyl-
[illegible]

CHOP 5.5 years - LDL

Metabolite	N	Estimate	Std..Error	P	P.adjusted	PartialRsquared
Ala	378	0.011	0.016	4.77E-01	1.00E+00	0.001
Arg	376	0.004	0.073	9.54E-01	1.00E+00	0.000
Asn	378	0.009	0.127	9.42E-01	1.00E+00	0.000
Asp	376	0.394	0.395	3.19E-01	1.00E+00	0.003
Cit	378	0.184	0.165	2.66E-01	1.00E+00	0.003
Gln	378	0.026	0.010	1.18E-02	1.00E+00	0.017
Glu	378	-0.035	0.015	2.48E-02	1.00E+00	0.013
Gly	378	0.017	0.027	5.31E-01	1.00E+00	0.001
His	378	-0.158	0.066	1.78E-02	1.00E+00	0.015
Ile	378	-0.023	0.079	7.75E-01	1.00E+00	0.000
Leu	378	-0.012	0.049	8.04E-01	1.00E+00	0.000
Lys	378	0.021	0.039	5.87E-01	1.00E+00	0.001
Met	378	-0.197	0.279	4.81E-01	1.00E+00	0.001
Orn	378	0.014	0.067	8.34E-01	1.00E+00	0.000
Phe	378	0.039	0.116	7.39E-01	1.00E+00	0.000
Pro	378	0.006	0.023	7.87E-01	1.00E+00	0.000
Ser	377	0.061	0.057	2.88E-01	1.00E+00	0.003
Thr	378	0.008	0.044	8.64E-01	1.00E+00	0.000
Trp	377	0.162	0.116	1.64E-01	1.00E+00	0.005
Tyr	378	0.007	0.082	9.36E-01	1.00E+00	0.000
Val	378	0.028	0.024	2.43E-01	1.00E+00	0.004
H1	377	-0.002	0.002	2.40E-01	1.00E+00	0.004
Carn	383	-0.108	0.143	4.49E-01	1.00E+00	0.001
Carn C2	383	-0.205	0.365	5.75E-01	1.00E+00	0.001
Carn C3	383	-3.357	13.465	8.03E-01	1.00E+00	0.000
Carn C3:1	269	501.178	236.904	3.53E-02	1.00E+00	0.016
Carn C4	383	-21.192	12.225	8.38E-02	1.00E+00	0.008
Carn C4:0:OH	269	0.613	17.075	9.71E-01	1.00E+00	0.000
Carn C4:1	341	28.218	42.007	5.02E-01	1.00E+00	0.001
Carn C5	383	-33.411	35.466	3.47E-01	1.00E+00	0.002
Carn C5:0:OH	310	1636.347	2917.968	5.75E-01	1.00E+00	0.001
Carn C6	311	-67.770	45.893	1.41E-01	1.00E+00	0.007
Carn C6:1	209	1394.960	414.355	9.09E-04	2.12E-01	0.051
Carn C8	323	4.792	8.946	5.93E-01	1.00E+00	0.001
Carn C8:1	383	27.982	24.595	2.56E-01	1.00E+00	0.003
Carn C9	383	-76.514	92.033	4.06E-01	1.00E+00	0.002
Carn C10	382	-3.503	5.787	5.45E-01	1.00E+00	0.001

Carn C10:1	383	-6.261	7.791	4.22E-01	1.00E+00	0.002
Carn C10:2	383	72.137	69.460	3.00E-01	1.00E+00	0.003
Carn C12::DC	260	76.716	17.842	2.43E-05	5.67E-03	0.067
Carn C12	382	-1.927	15.472	9.01E-01	1.00E+00	0.000
Carn C12:1	383	-3.329	5.666	5.57E-01	1.00E+00	0.001
Carn C14	383	23.030	43.463	5.97E-01	1.00E+00	0.001
Carn C14:1	381	8.327	11.040	4.51E-01	1.00E+00	0.001
Carn C14:2	383	31.674	51.212	5.37E-01	1.00E+00	0.001
Carn C14:2:OH	252	-166.547	148.071	2.62E-01	1.00E+00	0.005
Carn C15	383	61.458	124.567	6.22E-01	1.00E+00	0.001
Carn C16	382	86.032	47.523	7.10E-02	1.00E+00	0.009
Carn C16:OH	382	-1.340	50.617	9.79E-01	1.00E+00	0.000
Carn C16:1	280	215.283	287.940	4.55E-01	1.00E+00	0.002
Carn C16:2	269	-89.367	178.189	6.16E-01	1.00E+00	0.001
Carn C16:2:OH	249	-43.973	217.772	8.40E-01	1.00E+00	0.000
Carn C18	383	173.727	91.462	5.83E-02	1.00E+00	0.009
Carn C18:1	383	15.339	30.055	6.10E-01	1.00E+00	0.001
Carn C18:1:OH	322	157.412	196.641	4.24E-01	1.00E+00	0.002
Carn C18:2	383	2.420	43.140	9.55E-01	1.00E+00	0.000
Carn C18:2:OH	251	-537.117	572.558	3.49E-01	1.00E+00	0.003
Carn C20	381	268.683	294.167	3.62E-01	1.00E+00	0.002
Carn C20:1	309	2677.887	7930.640	7.36E-01	1.00E+00	0.000
Carn C20:3	383	-43.957	196.063	8.23E-01	1.00E+00	0.000
Carn C20:4	383	1679.996	2254.374	4.57E-01	1.00E+00	0.001
Carn C22	281	3038.971	3363.686	3.67E-01	1.00E+00	0.003
Carn C22:5	270	-230.613	482.280	6.33E-01	1.00E+00	0.001
Carn C22:6	248	81.677	1927.416	9.66E-01	1.00E+00	0.000
LPC C14	242	4.502	1.840	1.51E-02	1.00E+00	0.024
LPC C15	241	-1.968	4.787	6.81E-01	1.00E+00	0.001
LPC C16	382	0.028	0.047	5.49E-01	1.00E+00	0.001
LPC C16:1	383	0.127	1.301	9.22E-01	1.00E+00	0.000
LPC C17	381	1.934	1.991	3.32E-01	1.00E+00	0.002
LPC C18	383	0.047	0.116	6.85E-01	1.00E+00	0.000
LPC C18:1	383	0.330	0.251	1.90E-01	1.00E+00	0.004
LPC C18:2	383	0.198	0.131	1.32E-01	1.00E+00	0.006
LPC C18:3	342	-0.543	4.439	9.03E-01	1.00E+00	0.000
LPC C20:1	191	-9.972	6.934	1.52E-01	1.00E+00	0.011
LPC C20:3	381	1.216	1.342	3.66E-01	1.00E+00	0.002
LPC C20:4	383	-0.546	0.601	3.65E-01	1.00E+00	0.002
LPC C20:5	312	18.482	6.405	4.19E-03	9.75E-01	0.026

LPC C22:6	311	7.375	1.937	1.70E-04	3.96E-02	0.044
PCaa C20:4	212	-109.761	32.621	9.12E-04	2.13E-01	0.051
PCaa C28:2	312	95.203	30.198	1.78E-03	4.14E-01	0.031
PCaa C30	383	2.505	0.810	2.14E-03	4.99E-01	0.024
PCaa C30:2	382	26.732	5.447	1.37E-06	3.20E-04	0.060
PCaa C30:3	382	40.720	17.954	2.39E-02	1.00E+00	0.013
PCaa C30:4	160	68.778	56.409	2.25E-01	1.00E+00	0.009
PCaa C32	383	1.753	0.338	3.59E-07	8.36E-05	0.066
PCaa C32:1	383	0.933	0.244	1.56E-04	3.63E-02	0.037
PCaa C32:2	383	6.918	1.199	1.66E-08	3.88E-06	0.080
PCaa C34:1	383	0.073	0.025	3.66E-03	8.54E-01	0.022
PCaa C34:2	383	0.088	0.017	4.76E-07	1.11E-04	0.064
PCaa C34:3	381	1.379	0.270	5.23E-07	1.22E-04	0.064
PCaa C34:4	383	8.779	2.169	6.28E-05	1.46E-02	0.041
PCaa C36	268	0.780	0.608	2.01E-01	1.00E+00	0.006
PCaa C36:1	383	0.482	0.085	3.09E-08	7.19E-06	0.077
PCaa C36:2	383	0.107	0.022	2.55E-06	5.94E-04	0.056
PCaa C36:3	383	0.234	0.044	1.46E-07	3.40E-05	0.070
PCaa C36:4	383	0.078	0.034	2.25E-02	1.00E+00	0.014
PCaa C36:5	381	1.585	0.350	8.07E-06	1.88E-03	0.051
PCaa C36:6	313	4.450	2.002	2.70E-02	1.00E+00	0.015
PCaa C38	309	1.883	1.181	1.12E-01	1.00E+00	0.008
PCaa C38:3	383	0.652	0.114	2.08E-08	4.84E-06	0.079
PCaa C38:4	383	0.185	0.045	5.07E-05	1.18E-02	0.042
PCaa C38:5	383	0.868	0.119	1.66E-12	3.86E-10	0.122
PCaa C38:6	383	0.285	0.075	1.72E-04	4.01E-02	0.036
PCaa C40	272	-1.675	2.502	5.04E-01	1.00E+00	0.002
PCaa C40:4	382	5.668	1.039	8.94E-08	2.08E-05	0.072
PCaa C40:5	383	2.307	0.368	1.01E-09	2.35E-07	0.093
PCaa C40:6	383	1.004	0.169	6.26E-09	1.46E-06	0.085
PCaa C42	242	6.860	3.714	6.60E-02	1.00E+00	0.014
PCaa C42:1	212	13.228	8.912	1.39E-01	1.00E+00	0.010

PCaa C42:6	191	8.122	4.977	1.04E-01	1.00E+00	0.014
PCaa C43:6	252	-5.622	3.106	7.15E-02	1.00E+00	0.013
PCaa C44:12	383	-2.568	2.258	2.56E-01	1.00E+00	0.003
PCae C30:2	272	13.809	13.873	3.20E-01	1.00E+00	0.004
PCae C32	383	3.762	1.629	2.15E-02	1.00E+00	0.014
PCae C32:1	383	1.066	1.345	4.28E-01	1.00E+00	0.002
PCae C32:2	201	4.976	5.440	3.61E-01	1.00E+00	0.004
PCae C34	383	9.843	2.230	1.32E-05	3.08E-03	0.049
PCae C34:1	383	1.790	0.471	1.68E-04	3.90E-02	0.036
PCae C34:2	383	1.833	0.399	5.97E-06	1.39E-03	0.052
PCae C34:3	383	0.765	0.479	1.11E-01	1.00E+00	0.007
PCae C34:4	241	3.594	12.673	7.77E-01	1.00E+00	0.000
PCae C36	253	5.850	2.663	2.89E-02	1.00E+00	0.019
PCae C36:1	382	0.500	0.221	2.44E-02	1.00E+00	0.013
PCae C36:2	382	0.960	0.269	4.13E-04	9.63E-02	0.032
PCae C36:3	383	1.685	0.479	4.89E-04	1.14E-01	0.031
PCae C36:4	382	0.498	0.249	4.65E-02	1.00E+00	0.010
PCae C36:5	383	0.581	0.320	7.06E-02	1.00E+00	0.009
PCae C36:6	167	-1.349	4.539	7.67E-01	1.00E+00	0.001
PCae C38	310	3.894	0.972	7.80E-05	1.82E-02	0.049
PCae C38:2	301	0.277	0.300	3.56E-01	1.00E+00	0.003
PCae C38:3	381	0.494	0.308	1.10E-01	1.00E+00	0.007
PCae C38:4	383	0.278	0.327	3.95E-01	1.00E+00	0.002
PCae C38:5	383	0.412	0.256	1.09E-01	1.00E+00	0.007
PCae C38:6	383	1.453	0.551	8.73E-03	1.00E+00	0.018
PCae C40	383	2.714	0.417	2.43E-10	5.67E-08	0.100
PCae C40:1	229	5.377	1.581	7.91E-04	1.84E-01	0.049
PCae C40:3	312	0.237	0.436	5.87E-01	1.00E+00	0.001
PCae C40:4	343	1.165	0.700	9.70E-02	1.00E+00	0.008
PCae C40:5	383	2.011	0.730	6.18E-03	1.00E+00	0.019
PCae C40:6	383	2.706	0.935	4.01E-03	9.34E-01	0.021
PCae C42:2	323	6.710	2.378	5.08E-03	1.00E+00	0.024
PCae C42:3	272	-0.228	2.118	9.14E-01	1.00E+00	0.000
PCae C42:4	190	7.283	3.862	6.09E-02	1.00E+00	0.019
PCae C42:5	311	4.350	1.562	5.69E-03	1.00E+00	0.024
PCae C42:6	382	7.489	2.458	2.47E-03	5.76E-01	0.024
SM C21:2	343	60.611	56.941	2.88E-01	1.00E+00	0.003
SM C32:1	383	1.956	0.547	3.97E-04	9.26E-02	0.032

SM C32:2	343	44.200	7.131	1.66E-09	3.86E-07	0.101
SM C33:1	383	4.938	0.956	3.90E-07	9.09E-05	0.065
SM C33:3	182	22.908	22.086	3.01E-01	1.00E+00	0.006
SM C34:1	382	0.311	0.055	3.11E-08	7.24E-06	0.078
SM C34:2	382	0.756	0.405	6.29E-02	1.00E+00	0.009
SM C34:4	210	215.221	92.004	2.03E-02	1.00E+00	0.026
SM C35:1	382	7.921	1.605	1.20E-06	2.79E-04	0.060
SM C35:2	192	26.767	12.181	2.92E-02	1.00E+00	0.025
SM C36:1	381	1.267	0.275	5.43E-06	1.27E-03	0.053
SM C36:2	382	2.115	0.515	4.97E-05	1.16E-02	0.043
SM C36:3	180	2.653	6.224	6.71E-01	1.00E+00	0.001
SM C37:1	192	2.614	1.465	7.61E-02	1.00E+00	0.017
SM C37:3	201	16.783	9.628	8.29E-02	1.00E+00	0.015
SM C38:2	383	1.690	0.332	5.78E-07	1.35E-04	0.064
SM C39:1	311	2.300	0.859	7.84E-03	1.00E+00	0.023
SM C40:2	232	0.702	0.192	3.17E-04	7.38E-02	0.055
SM C40:4	302	7.123	2.085	7.23E-04	1.69E-01	0.038
SM C40:5	343	22.364	3.987	4.24E-08	9.88E-06	0.084
SM C41:1	382	1.161	0.301	1.34E-04	3.13E-02	0.038
SM C41:2	382	0.280	0.439	5.25E-01	1.00E+00	0.001
SM C42:1	383	0.706	0.206	6.85E-04	1.60E-01	0.030
SM C42:2	382	0.250	0.123	4.25E-02	1.00E+00	0.011
SM C42:3	313	1.079	0.258	3.72E-05	8.66E-03	0.053
SM C42:6	383	2.547	1.477	8.54E-02	1.00E+00	0.008
SM C43	173	11.925	7.455	1.12E-01	1.00E+00	0.015
SM C43:1	202	9.814	2.666	3.00E-04	6.98E-02	0.064
SM C43:2	312	3.999	1.766	2.42E-02	1.00E+00	0.016
SM C44:6	230	7.544	3.587	3.66E-02	1.00E+00	0.019
SM C47:6	323	1066.189	232.159	6.31E-06	1.47E-03	0.061
NEFA 12:0	181	-1.071	0.518	4.03E-02	1.00E+00	0.023

NEFA 14:0	321	-0.176	0.160	2.73E-01	1.00E+00	0.004
NEFA 14:1	380	-0.243	0.252	3.35E-01	1.00E+00	0.002
NEFA 15:0	181	-5.272	2.158	1.56E-02	1.00E+00	0.032
NEFA 16:0	248	-0.046	0.026	7.11E-02	1.00E+00	0.013
NEFA 16:1	380	-0.206	0.073	4.88E-03	1.00E+00	0.021
NEFA 16:2	299	-0.124	2.486	9.60E-01	1.00E+00	0.000
NEFA 17:0	168	-0.194	0.761	7.99E-01	1.00E+00	0.000
NEFA 17:1	380	-0.823	0.663	2.15E-01	1.00E+00	0.004
NEFA 17:2	168	-28.059	27.299	3.06E-01	1.00E+00	0.006
NEFA 18:1	380	-0.030	0.012	1.44E-02	1.00E+00	0.016
NEFA 18:2	380	-0.056	0.028	4.66E-02	1.00E+00	0.010
NEFA 18:3	380	-0.218	0.394	5.80E-01	1.00E+00	0.001
NEFA 18:4	168	-19.841	15.612	2.06E-01	1.00E+00	0.010
NEFA 19:1	297	-0.600	3.152	8.49E-01	1.00E+00	0.000
NEFA 20:1	380	-0.436	0.839	6.03E-01	1.00E+00	0.001
NEFA 20:2	380	-0.122	0.461	7.91E-01	1.00E+00	0.000
NEFA 20:3	380	-1.693	0.893	5.87E-02	1.00E+00	0.009
NEFA 20:4	379	-0.648	0.299	3.07E-02	1.00E+00	0.012
NEFA 20:5	296	-0.486	1.616	7.64E-01	1.00E+00	0.000
NEFA 22:3	227	28.760	47.652	5.47E-01	1.00E+00	0.002
NEFA 22:4	380	-2.798	3.828	4.65E-01	1.00E+00	0.001
NEFA 22:5	377	-1.624	1.960	4.08E-01	1.00E+00	0.002
NEFA 22:6	380	-0.287	0.791	7.16E-01	1.00E+00	0.000
NEFA 24:0	168	21.323	12.924	1.01E-01	1.00E+00	0.016
NEFA 24:1	380	0.792	5.448	8.84E-01	1.00E+00	0.000
NEFA 24:2	168	200.275	93.916	3.45E-02	1.00E+00	0.027
NEFA 24:3	239	15.272	69.387	8.26E-01	1.00E+00	0.000
NEFA 24:4						
	380	-5.286	67.012	9.37E-01	1.00E+00	0.000
NEFA 24:6	159	235.416	188.603	2.14E-01	1.00E+00	0.010
NEFA 26:0	324	8.514	8.061	2.92E-01	1.00E+00	0.003
NEFA 26:1	308	8.900	9.826	3.66E-01	1.00E+00	0.003
NEFA 26:2	380	-1.163	11.726	9.21E-01	1.00E+00	0.000
NEFA 26:3	298	31.631	36.680	3.89E-01	1.00E+00	0.003
NEFA 26:4	380	-192.793	146.244	1.88E-01	1.00E+00	0.005

Pyruvate	375	-0.001	0.007	8.67E-01	1.00E+00	0.000
Lactic_acid	380	0.001	0.001	2.19E-02	1.00E+00	0.014
Fumarate	373	-0.837	4.682	8.58E-01	1.00E+00	0.000
3-Methyl-2-oxobutanoate	375	0.047	0.095	6.25E-01	1.00E+00	0.001
Succininate	378	0.145	0.405	7.20E-01	1.00E+00	0.000
Methylmalonate	380	35.840	18.777	5.71E-02	1.00E+00	0.010
Taurine	379	0.053	0.035	1.28E-01	1.00E+00	0.006
3-Methyl-2-oxovalerate	379	0.150	0.107	1.63E-01	1.00E+00	0.005
4-Methyl-2-oxovalerate	374	0.019	0.054	7.31E-01	1.00E+00	0.000
Malate	376	0.515	0.769	5.04E-01	1.00E+00	0.001
Alpha-ketoglutarate	369	-0.251	0.244	3.04E-01	1.00E+00	0.003
Alpha-aminoadipate	379	-6.942	8.515	4.15E-01	1.00E+00	0.002
Isocitrate	380	-0.153	0.244	5.32E-01	1.00E+00	0.001
Citrate	380	0.011	0.018	5.54E-01	1.00E+00	0.001
Beta-hydroxybutyrate	147	0.075	0.052	1.48E-01	1.00E+00	0.015
Sum AA	375	0.002	0.003	5.83E-01	1.00E+00	0.001
Sum Carn	380	-0.117	0.131	3.71E-01	1.00E+00	0.002
Sum LPC	201	0.028	0.044	5.17E-01	1.00E+00	0.002
Sum PCaa	306	0.032	0.005	7.42E-09	1.73E-06	0.104
Sum PCae	269	0.199	0.046	2.21E-05	5.14E-03	0.065
Sum SM	341	0.165	0.050	1.00E-03	2.34E-01	0.031
Sum NEFA	99	-0.013	0.008	1.13E-01	1.00E+00	0.026

CHOP 8 years - LDL

Metabolite	N	Estimate	Std..Error	P	P.adjusted	PartialRsquared
Ala	348	0.005	0.019	8.10E-01	1.00E+00	0.000
Arg	348	0.119	0.086	1.69E-01	1.00E+00	0.005
Asn	348	-0.041	0.141	7.72E-01	1.00E+00	0.000
Asp	348	-0.504	0.443	2.56E-01	1.00E+00	0.004
Cit	348	0.247	0.195	2.07E-01	1.00E+00	0.005
Gln	347	-0.001	0.009	8.90E-01	1.00E+00	0.000
Glu	348	-0.017	0.015	2.35E-01	1.00E+00	0.004
Gly	348	-0.063	0.030	3.88E-02	1.00E+00	0.012
His	348	0.081	0.055	1.46E-01	1.00E+00	0.006
Ile	348	0.161	0.134	2.28E-01	1.00E+00	0.004
Leu	348	0.071	0.077	3.54E-01	1.00E+00	0.002
Lys	348	0.036	0.087	6.81E-01	1.00E+00	0.000
Met	348	-0.295	0.318	3.55E-01	1.00E+00	0.002
Orn	348	-0.039	0.091	6.69E-01	1.00E+00	0.001
Phe	347	0.034	0.151	8.20E-01	1.00E+00	0.000
Pro	348	0.008	0.035	8.31E-01	1.00E+00	0.000
Ser	347	-0.043	0.048	3.71E-01	1.00E+00	0.002
Thr	348	0.014	0.058	8.07E-01	1.00E+00	0.000
Trp	348	0.163	0.141	2.48E-01	1.00E+00	0.004
Tyr	347	-0.022	0.111	8.43E-01	1.00E+00	0.000
Val	348	0.092	0.036	1.05E-02	1.00E+00	0.019
H1	348	0.004	0.002	9.79E-02	1.00E+00	0.008
Carn	348	0.074	0.066	2.58E-01	1.00E+00	0.004
Carn C2	348	0.494	0.528	3.51E-01	1.00E+00	0.002
Carn C3	345	3.333	4.231	4.31E-01	1.00E+00	0.002
Carn C3:1	283	-20.261	63.759	7.51E-01	1.00E+00	0.000
Carn C4:0:OH	235	5.393	18.750	7.74E-01	1.00E+00	0.000
Carn C5	348	-1221.383	957.252	2.03E-01	1.00E+00	0.005
Carn C5:0:OH	348	-8.179	17.398	6.39E-01	1.00E+00	0.001
Carn C6	348	2.626	27.650	9.24E-01	1.00E+00	0.000
Carn C6:1	348	8.402	35.873	8.15E-01	1.00E+00	0.000
Carn C8	348	-5.354	7.858	4.96E-01	1.00E+00	0.001
Carn C8:1	348	38.049	19.882	5.65E-02	1.00E+00	0.010
Carn C9	348	-13.089	74.057	8.60E-01	1.00E+00	0.000
Carn C10	348	-3.524	4.633	4.47E-01	1.00E+00	0.002

Carn C10:1	347	0.179	7.692	9.81E-01	1.00E+00	0.000
Carn C10:2	348	99.929	55.390	7.21E-02	1.00E+00	0.009
Carn C12	348	5.725	11.876	6.30E-01	1.00E+00	0.001
Carn C12:1	348	-0.925	4.964	8.52E-01	1.00E+00	0.000
Carn C14	348	32.127	49.969	5.21E-01	1.00E+00	0.001
Carn C14:1	347	7.103	8.128	3.83E-01	1.00E+00	0.002
Carn C14:2	348	27.781	37.981	4.65E-01	1.00E+00	0.002
Carn C14:2:OH	346	206.130	116.504	7.77E-02	1.00E+00	0.009
Carn C15	348	508.808	271.502	6.18E-02	1.00E+00	0.010
Carn C16	348	66.821	61.112	2.75E-01	1.00E+00	0.003
Carn C16:0:OH	285	434.914	725.206	5.49E-01	1.00E+00	0.001
Carn C16:1	348	35.159	52.735	5.05E-01	1.00E+00	0.001
Carn C16:2	348	172.248	136.511	2.08E-01	1.00E+00	0.005
Carn C16:2:OH	299	203.867	272.378	4.55E-01	1.00E+00	0.002
Carn C18	348	399.292	132.940	2.86E-03	5.44E-01	0.025
Carn C18:1	348	-65.834	40.244	1.03E-01	1.00E+00	0.008
Carn C18:1:OH	348	165.129	183.429	3.69E-01	1.00E+00	0.002
Carn C18:2	346	32.159	65.740	6.25E-01	1.00E+00	0.001
Carn C18:2:OH	228	627.065	487.653	2.00E-01	1.00E+00	0.007
Carn C20	342	-215.027	264.694	4.17E-01	1.00E+00	0.002
Carn C20:1	347	69937.297	18298.352	1.57E-04	2.99E-02	0.040
Carn C20:3	348	720.905	709.009	3.10E-01	1.00E+00	0.003
Carn C20:4	299	8640.888	9332.190	3.55E-01	1.00E+00	0.003
Carn C22	218	13487.501	8236.943	1.03E-01	1.00E+00	0.012
Carn C22:5	251	3430.910	1353.262	1.19E-02	1.00E+00	0.025
Carn C22:6	181	2792.275	1008.734	6.24E-03	1.00E+00	0.041
LPC C16	348	0.032	0.030	2.92E-01	1.00E+00	0.003
LPC C16:1	348	1.656	1.079	1.26E-01	1.00E+00	0.007
LPC C17	285	3.614	1.648	2.91E-02	1.00E+00	0.016
LPC C18	348	0.148	0.084	7.72E-02	1.00E+00	0.009
LPC C18:1	348	0.283	0.193	1.44E-01	1.00E+00	0.006
LPC C18:2	348	0.242	0.105	2.16E-02	1.00E+00	0.015
LPC C18:3	217	10.737	5.845	6.76E-02	1.00E+00	0.015
LPC C18:6	178	18.578	8.182	2.44E-02	1.00E+00	0.028
LPC C20:3	281	5.603	1.502	2.33E-04	4.42E-02	0.047
LPC C20:4	348	1.719	0.533	1.39E-03	2.64E-01	0.029
LPC C20:5	164	20.251	5.833	6.65E-04	1.26E-01	0.070

LPC C22:5	150	30.826	6.664	8.15E-06	1.55E-03	0.126
LPC C22:6	250	8.912	2.160	5.05E-05	9.60E-03	0.064
PCaa C30:5	180	531.952	602.552	3.79E-01	1.00E+00	0.004
PCaa C32	348	1.266	0.358	4.63E-04	8.80E-02	0.034
PCaa C32:1	285	0.543	0.227	1.73E-02	1.00E+00	0.020
PCaa C34:1	348	0.111	0.023	1.64E-06	3.12E-04	0.064
PCaa C34:2	282	0.066	0.017	9.55E-05	1.81E-02	0.053
PCaa C34:3	348	1.026	0.245	3.52E-05	6.68E-03	0.048
PCaa C34:4	299	8.330	1.991	3.79E-05	7.21E-03	0.055
PCaa C36:2	348	0.070	0.016	1.58E-05	3.01E-03	0.052
PCaa C36:3	348	0.178	0.047	1.88E-04	3.57E-02	0.039
PCaa C36:4	282	0.179	0.038	4.15E-06	7.89E-04	0.072
PCaa C36:5	279	0.811	0.241	8.56E-04	1.63E-01	0.039
PCaa C38	162	1.217	1.103	2.71E-01	1.00E+00	0.007
PCaa C38:3	160	0.784	0.186	4.07E-05	7.73E-03	0.103
PCaa C38:4	181	0.084	0.058	1.55E-01	1.00E+00	0.011
PCaa C38:5	236	0.455	0.151	2.86E-03	5.44E-01	0.037
PCaa C38:6	348	0.243	0.059	4.36E-05	8.29E-03	0.047
PCaa C40:4	152	4.809	1.598	3.09E-03	5.86E-01	0.057
PCaa C40:5	169	1.609	0.532	2.89E-03	5.49E-01	0.051
PCaa C40:6	347	0.710	0.146	1.71E-06	3.24E-04	0.064

PCaa C44:12	170	8.431	6.199	1.76E-01	1.00E+00	0.011
PCae C34:1	348	1.802	0.409	1.44E-05	2.74E-03	0.052
PCae C34:2	281	0.760	0.322	1.88E-02	1.00E+00	0.019
PCae C34:3	348	0.531	0.370	1.53E-01	1.00E+00	0.006
PCae C36:1	233	-0.024	0.268	9.29E-01	1.00E+00	0.000
PCae C36:2	151	1.085	0.473	2.32E-02	1.00E+00	0.034
PCae C36:3	348	1.631	0.390	3.71E-05	7.06E-03	0.047
PCae C36:4	281	0.584	0.199	3.54E-03	6.73E-01	0.030
PCae C36:5	348	1.193	0.315	1.83E-04	3.47E-02	0.039
PCae C38	180	2.138	1.630	1.91E-01	1.00E+00	0.010
PCae C38:3	180	-0.099	0.357	7.82E-01	1.00E+00	0.000
PCae C38:4	170	1.732	0.571	2.81E-03	5.35E-01	0.052
PCae C38:5	285	0.769	0.223	6.28E-04	1.19E-01	0.040
PCae C38:6	236	2.643	0.577	7.55E-06	1.44E-03	0.081
PCae C40	162	0.667	0.630	2.91E-01	1.00E+00	0.007
PCae C40:4	169	1.390	1.080	2.00E-01	1.00E+00	0.010
PCae C40:6	169	2.318	1.258	6.73E-02	1.00E+00	0.019
SM C21:2	178	54.355	30.752	7.89E-02	1.00E+00	0.018
SM C31:1	181	37.422	20.633	7.14E-02	1.00E+00	0.017
SM C32:1	348	2.843	0.569	9.51E-07	1.81E-04	0.067

SM C32:2	170	16.972	6.610	1.11E-02	1.00E+00	0.037
SM C33:1	348	3.833	0.781	1.41E-06	2.68E-04	0.065
SM C34:1	348	0.350	0.053	1.31E-10	2.49E-08	0.112
SM C34:2	348	1.259	0.369	7.15E-04	1.36E-01	0.033
SM C35:1	299	6.572	1.490	1.44E-05	2.74E-03	0.061
SM C36:1	348	1.030	0.206	9.31E-07	1.77E-04	0.067
SM C36:2	218	2.541	0.585	2.14E-05	4.07E-03	0.079
SM C36:3	280	2.660	4.396	5.46E-01	1.00E+00	0.001
SM C41:1	236	1.961	0.418	4.71E-06	8.95E-04	0.085
SM C41:2	235	2.122	0.538	1.06E-04	2.01E-02	0.063
SM C42:1	185	1.160	0.274	3.73E-05	7.08E-03	0.089
SM C42:2	170	0.426	0.150	4.97E-03	9.45E-01	0.046
NEFA 10:0	333	-0.581	0.516	2.61E-01	1.00E+00	0.004
NEFA 11:0	260	34.067	15.340	2.72E-02	1.00E+00	0.018
NEFA 12:0	332	1.313	0.705	6.36E-02	1.00E+00	0.010
NEFA 12:1	217	-8.184	17.258	6.36E-01	1.00E+00	0.001

NEFA 14:0	333	-0.033	0.158	8.37E-01	1.00E+00	0.000
NEFA 14:1	332	-0.026	0.851	9.76E-01	1.00E+00	0.000
NEFA 15:0	333	-0.242	0.739	7.43E-01	1.00E+00	0.000
NEFA 15:1	210	-8.840	12.067	4.65E-01	1.00E+00	0.003
NEFA 16:0	333	-0.007	0.020	7.44E-01	1.00E+00	0.000
NEFA 16:1	333	-0.054	0.090	5.50E-01	1.00E+00	0.001
NEFA 16:2	333	4.548	5.990	4.48E-01	1.00E+00	0.002
NEFA 17:0	333	0.054	0.739	9.42E-01	1.00E+00	0.000
NEFA 17:1	333	-0.020	1.243	9.87E-01	1.00E+00	0.000
NEFA 18:0	278	-0.046	0.051	3.70E-01	1.00E+00	0.003
NEFA 18:1	333	-0.001	0.015	9.27E-01	1.00E+00	0.000
NEFA 18:2	331	-0.011	0.051	8.30E-01	1.00E+00	0.000
NEFA 18:3	333	0.249	0.207	2.30E-01	1.00E+00	0.004
NEFA 18:4	331	19.783	13.387	1.40E-01	1.00E+00	0.007
NEFA 19:0	278	-2.536	8.578	7.68E-01	1.00E+00	0.000
NEFA 19:1	333	2.550	2.597	3.27E-01	1.00E+00	0.003
NEFA 20:0	333	-1.175	3.649	7.48E-01	1.00E+00	0.000
NEFA 20:1	333	0.611	0.890	4.93E-01	1.00E+00	0.001
NEFA 20:2	332	1.123	1.523	4.62E-01	1.00E+00	0.002
NEFA 20:3	333	0.156	0.927	8.66E-01	1.00E+00	0.000
NEFA 20:4	333	-0.033	0.242	8.91E-01	1.00E+00	0.000
NEFA 20:5	331	2.762	2.458	2.62E-01	1.00E+00	0.004
NEFA 22:0	285	3.791	12.890	7.69E-01	1.00E+00	0.000
NEFA 22:1	332	10.388	11.086	3.49E-01	1.00E+00	0.003
NEFA 22:2	278	46.480	53.839	3.89E-01	1.00E+00	0.003
NEFA 22:3	230	104.835	46.356	2.47E-02	1.00E+00	0.021
NEFA 22:4	332	1.014	3.081	7.42E-01	1.00E+00	0.000
NEFA 22:5	333	1.723	1.608	2.85E-01	1.00E+00	0.003
NEFA 22:6	333	1.229	0.726	9.14E-02	1.00E+00	0.008
NEFA 24:0	333	18.771	12.749	1.42E-01	1.00E+00	0.006
NEFA 24:1	333	17.858	10.657	9.47E-02	1.00E+00	0.008
NEFA 24:3	144	-201.038	332.838	5.47E-01	1.00E+00	0.003
NEFA 24:4	218	59.436	70.500	4.00E-01	1.00E+00	0.003
NEFA 24:5	163	-54.732	69.811	4.34E-01	1.00E+00	0.004
NEFA 24:6	218	5.753	44.572	8.97E-01	1.00E+00	0.000
NEFA 26:0	285	22.808	81.325	7.79E-01	1.00E+00	0.000
NEFA 26:1	333	75.681	18.530	5.56E-05	1.06E-02	0.047
NEFA 26:2	284	22.846	25.367	3.69E-01	1.00E+00	0.003
NEFA 26:3	332	-16.126	42.543	7.05E-01	1.00E+00	0.000
NEFA 26:4	164	254.061	247.747	3.07E-01	1.00E+00	0.006
NEFA 26:5	180	108.568	241.683	6.54E-01	1.00E+00	0.001

3-Methyl-2-oxovalerate	342	0.173	0.273	5.27E-01	1.00E+00	0.001
4-Methyl-2-oxovalerate	342	0.090	0.160	5.74E-01	1.00E+00	0.001
Acetoacetate	243	0.002	0.013	8.53E-01	1.00E+00	0.000
Alpha-aminoadipate	158	3.657	4.556	4.23E-01	1.00E+00	0.004
Alpha-ketobutyrate	328	-0.018	0.059	7.64E-01	1.00E+00	0.000
Citrate	293	0.041	0.049	3.97E-01	1.00E+00	0.002
Isocitrate	294	-0.155	0.266	5.60E-01	1.00E+00	0.001
Lactate	340	0.002	0.002	2.83E-01	1.00E+00	0.003
Malate	340	-0.077	0.339	8.20E-01	1.00E+00	0.000
Alpha-ketoglutarate	342	-0.133	0.228	5.59E-01	1.00E+00	0.001
Pyruvate	222	-0.018	0.022	4.12E-01	1.00E+00	0.003
Succininate	342	0.130	0.301	6.67E-01	1.00E+00	0.001
Taurine	294	-0.021	0.049	6.63E-01	1.00E+00	0.001
Sum AA	345	-0.001	0.005	9.10E-01	1.00E+00	0.000
Sum Carn	344	0.059	0.066	3.72E-01	1.00E+00	0.002
Sum LPC	53	0.052	0.028	6.70E-02	1.00E+00	0.067
Sum PCaa	47	0.023	0.018	2.00E-01	1.00E+00	0.038
Sum PCae	48	0.159	0.134	2.44E-01	1.00E+00	0.031
Sum SM	54	0.359	0.110	1.97E-03	3.75E-01	0.171
Sum NEFA	213	0.005	0.008	4.85E-01	1.00E+00	0.002

GINI-LISA 10 years - LDL

Metabolite	N	Estimate	Std..Error	P	P.adjusted	PartialRsquared
Ala	250	-0.038	0.016	1.60E-02	1.00E+00	0.023
Arg	250	-0.090	0.073	2.22E-01	1.00E+00	0.006
Asn	250	-0.212	0.133	1.13E-01	1.00E+00	0.010
Asp	249	0.093	0.140	5.08E-01	1.00E+00	0.002
Cit	250	-0.353	0.174	4.35E-02	1.00E+00	0.016
Gln	221	-0.037	0.012	2.60E-03	6.31E-01	0.041
Glu	250	-0.012	0.029	6.82E-01	1.00E+00	0.001
Gly	250	-0.070	0.024	4.53E-03	1.00E+00	0.032
His	250	0.030	0.072	6.79E-01	1.00E+00	0.001
Ile	250	-0.044	0.109	6.85E-01	1.00E+00	0.001
Leu	250	0.008	0.059	8.90E-01	1.00E+00	0.000
Lys	250	-0.071	0.041	8.35E-02	1.00E+00	0.012
Met	250	-0.597	0.317	6.08E-02	1.00E+00	0.014
Orn	250	-0.088	0.054	1.05E-01	1.00E+00	0.011
Phe	250	-0.050	0.094	5.99E-01	1.00E+00	0.001
Pro	250	-0.078	0.024	1.56E-03	3.79E-01	0.040
Ser	250	-0.063	0.052	2.25E-01	1.00E+00	0.006
Thr	250	-0.176	0.050	5.10E-04	1.24E-01	0.048
Trp	250	-0.013	0.127	9.17E-01	1.00E+00	0.000
Tyr	250	-0.125	0.096	1.96E-01	1.00E+00	0.007
Val	250	0.033	0.031	2.83E-01	1.00E+00	0.005
H1	252	0.001	0.002	5.37E-01	1.00E+00	0.002
Carn	191	0.122	0.283	6.66E-01	1.00E+00	0.001
Carn C2	195	1.721	0.837	4.11E-02	1.00E+00	0.022
Carn C3	207	24.668	19.901	2.17E-01	1.00E+00	0.008
Carn C4	163	66.587	23.391	5.00E-03	1.00E+00	0.048
Carn C5	153	62.305	56.370	2.71E-01	1.00E+00	0.008
Carn C6	251	43.343	38.566	2.62E-01	1.00E+00	0.005
Carn C8	252	3.538	5.212	4.98E-01	1.00E+00	0.002
Carn C8:1	251	24.067	24.404	3.25E-01	1.00E+00	0.004
Carn C9	230	-21.111	62.424	7.36E-01	1.00E+00	0.001
Carn C10	251	0.377	4.076	9.26E-01	1.00E+00	0.000

Carn C10:1	252	4.583	6.608	4.89E-01	1.00E+00	0.002
Carn C10:2	153	50.199	45.398	2.71E-01	1.00E+00	0.008
Carn C12	251	7.832	9.284	4.00E-01	1.00E+00	0.003
Carn C12:1	223	3.301	4.068	4.18E-01	1.00E+00	0.003
Carn C14	162	31.652	74.215	6.70E-01	1.00E+00	0.001
Carn C14:1	251	17.214	14.257	2.28E-01	1.00E+00	0.006
Carn C14:2	163	143.405	51.589	6.10E-03	1.00E+00	0.046
Carn C16	230	190.945	59.772	1.60E-03	3.89E-01	0.043
Carn C16:1	200	152.195	89.639	9.11E-02	1.00E+00	0.014
Carn C18	189	270.731	75.964	4.65E-04	1.13E-01	0.064
Carn C18:1	252	61.417	36.196	9.10E-02	1.00E+00	0.011
Carn C18:2	178	139.441	82.393	9.24E-02	1.00E+00	0.016

LPC C14	252	4.139	1.707	1.61E-02	1.00E+00	0.023
LPC C15	234	12.624	4.284	3.54E-03	8.60E-01	0.036
LPC C16	252	0.184	0.062	3.49E-03	8.48E-01	0.034
LPC C16:1	252	3.577	1.911	6.24E-02	1.00E+00	0.014
LPC C17	252	6.913	2.664	1.00E-02	1.00E+00	0.026
LPC C17:1	234	15.621	13.065	2.33E-01	1.00E+00	0.006
LPC C18	252	0.572	0.180	1.72E-03	4.19E-01	0.039
LPC C18:1	252	0.606	0.357	9.07E-02	1.00E+00	0.011
LPC C18:2	252	0.233	0.190	2.20E-01	1.00E+00	0.006
LPC C18:3	234	2.608	6.704	6.98E-01	1.00E+00	0.001
LPC C18:6	152	-9.503	15.972	5.53E-01	1.00E+00	0.002
LPC C20:2	135	21.703	15.971	1.77E-01	1.00E+00	0.014
LPC C20:3	252	9.812	2.374	4.90E-05	1.19E-02	0.064
LPC C20:4	252	2.310	0.870	8.42E-03	1.00E+00	0.028
LPC C20:5	249	17.587	7.885	2.66E-02	1.00E+00	0.020

LPC C22:5	187	20.751	9.005	2.23E-02	1.00E+00	0.028
LPC C22:6	234	9.343	2.805	1.01E-03	2.45E-01	0.046
PCaa C30	252	5.149	1.221	3.50E-05	8.50E-03	0.067
PCaa C30:2	178	30.719	7.191	3.18E-05	7.73E-03	0.095
PCaa C30:3	173	80.443	20.913	1.69E-04	4.12E-02	0.080
PCaa C30:4	126	63.299	39.441	1.11E-01	1.00E+00	0.021
PCaa C32	252	2.541	0.538	3.82E-06	9.29E-04	0.082
PCaa C32:1	252	0.542	0.293	6.52E-02	1.00E+00	0.014
PCaa C32:2	252	5.781	1.283	1.02E-05	2.49E-03	0.076
PCaa C32:3	197	45.524	7.888	3.09E-08	7.51E-06	0.147
PCaa C34:1	252	0.154	0.036	2.34E-05	5.68E-03	0.070
PCaa C34:2	252	0.101	0.021	1.54E-06	3.74E-04	0.089
PCaa C34:3	252	1.056	0.349	2.73E-03	6.63E-01	0.036
PCaa C34:4	252	8.864	2.325	1.74E-04	4.23E-02	0.055
PCaa C34:5	167	24.770	23.355	2.90E-01	1.00E+00	0.007
PCaa C36	192	2.087	0.842	1.41E-02	1.00E+00	0.032
PCaa C36:1	252	0.429	0.135	1.64E-03	3.98E-01	0.039
PCaa C36:2	252	0.127	0.028	1.28E-05	3.11E-03	0.074
PCaa C36:3	252	0.325	0.054	7.76E-09	1.89E-06	0.125
PCaa C36:4	252	0.216	0.044	1.39E-06	3.37E-04	0.090
PCaa C36:5	251	1.061	0.309	6.97E-04	1.69E-01	0.045
PCaa C36:6	231	10.088	3.733	7.40E-03	1.00E+00	0.031
PCaa C38	252	3.009	1.470	4.17E-02	1.00E+00	0.017
PCaa C38:2	117	5.125	1.233	6.33E-05	1.54E-02	0.130
PCaa C38:3	252	0.699	0.121	2.28E-08	5.55E-06	0.118
PCaa C38:4	252	0.273	0.057	3.44E-06	8.36E-04	0.083
PCaa C38:5	252	0.646	0.125	4.39E-07	1.07E-04	0.097
PCaa C38:6	252	0.399	0.080	1.02E-06	2.48E-04	0.092
PCaa C40	184	6.666	3.941	9.25E-02	1.00E+00	0.016
PCaa C40:1	200	4.873	3.027	1.09E-01	1.00E+00	0.013
PCaa C40:3	139	4.110	2.730	1.35E-01	1.00E+00	0.016
PCaa C40:4	252	5.262	1.198	1.66E-05	4.03E-03	0.072
PCaa C40:5	252	2.191	0.467	4.59E-06	1.12E-03	0.081
PCaa C40:6	252	0.842	0.199	3.22E-05	7.82E-03	0.067
PCaa C42	238	7.192	5.490	1.91E-01	1.00E+00	0.007
PCaa C42:1	124	3.438	9.484	7.18E-01	1.00E+00	0.001

PCaa C42:4	122	5.522	7.046	4.35E-01	1.00E+00	0.005
PCaa C42:5	161	24.973	8.220	2.79E-03	6.78E-01	0.055
PCaa C42:6	138	12.025	4.872	1.48E-02	1.00E+00	0.043
PCaa C43:6	249	9.335	4.702	4.82E-02	1.00E+00	0.016
PCaa C44:12	222	8.289	5.778	1.53E-01	1.00E+00	0.009
PCae C32	252	8.339	1.974	3.37E-05	8.19E-03	0.067
PCae C32:1	252	6.433	1.875	7.02E-04	1.71E-01	0.045
PCae C32:2	217	19.685	5.051	1.30E-04	3.17E-02	0.066
PCae C34	231	21.468	3.528	4.90E-09	1.19E-06	0.140
PCae C34:1	252	2.216	0.746	3.28E-03	7.97E-01	0.034
PCae C34:2	252	1.525	0.558	6.76E-03	1.00E+00	0.029
PCae C34:3	252	1.896	0.626	2.72E-03	6.62E-01	0.036
PCae C34:4	144	31.681	19.350	1.04E-01	1.00E+00	0.019
PCae C36	182	18.649	6.599	5.25E-03	1.00E+00	0.043
PCae C36:1	252	0.882	0.483	6.89E-02	1.00E+00	0.013
PCae C36:2	252	1.850	0.431	2.50E-05	6.07E-03	0.069
PCae C36:3	252	2.627	0.724	3.47E-04	8.44E-02	0.050
PCae C36:4	252	0.871	0.313	5.81E-03	1.00E+00	0.030
PCae C36:5	252	1.776	0.451	1.08E-04	2.61E-02	0.059
PCae C36:6	129	19.078	10.207	6.40E-02	1.00E+00	0.027
PCae C38	251	4.729	1.652	4.57E-03	1.00E+00	0.032
PCae C38:2	252	1.031	0.789	1.93E-01	1.00E+00	0.007
PCae C38:3	252	1.472	0.541	7.00E-03	1.00E+00	0.029
PCae C38:4	252	1.467	0.422	5.97E-04	1.45E-01	0.046
PCae C38:5	252	0.840	0.360	2.04E-02	1.00E+00	0.021
PCae C38:6	252	2.984	0.696	2.62E-05	6.37E-03	0.069
PCae C40	223	2.129	0.504	3.58E-05	8.70E-03	0.075
PCae C40:1	234	5.979	1.908	1.95E-03	4.74E-01	0.041
PCae C40:2	171	2.425	1.907	2.05E-01	1.00E+00	0.010
PCae C40:3	189	0.867	0.972	3.73E-01	1.00E+00	0.004
PCae C40:4	223	3.783	1.315	4.41E-03	1.00E+00	0.036
PCae C40:5	252	2.743	1.016	7.42E-03	1.00E+00	0.028
PCae C40:6	252	4.965	1.228	7.01E-05	1.70E-02	0.062
PCae C42	194	4.840	4.626	2.97E-01	1.00E+00	0.006
PCae C42:1	172	-1.382	2.805	6.23E-01	1.00E+00	0.001
PCae C42:2	205	13.213	3.940	9.53E-04	2.32E-01	0.053
PCae C42:3	212	7.362	2.725	7.46E-03	1.00E+00	0.034
PCae C42:4	190	7.893	4.616	8.90E-02	1.00E+00	0.015
PCae C42:5	232	4.239	2.026	3.75E-02	1.00E+00	0.019
PCae C42:6	252	10.182	3.505	4.00E-03	9.72E-01	0.033
SM C30:1	210	56.678	9.582	1.36E-08	3.31E-06	0.145
SM C31:1	125	100.883	22.482	1.65E-05	4.02E-03	0.141
SM C32:1	252	5.012	0.652	3.44E-13	8.36E-11	0.192

SM C32:2	252	40.864	6.319	5.29E-10	1.28E-07	0.144
SM C33:1	252	7.181	1.036	3.60E-11	8.75E-09	0.162
SM C33:2	184	107.846	26.011	5.20E-05	1.26E-02	0.087
SM C33:3	181	182.594	42.823	3.26E-05	7.93E-03	0.093
SM C34:1	252	0.518	0.068	5.23E-13	1.27E-10	0.189
SM C34:2	252	3.342	0.467	9.12E-12	2.22E-09	0.170
SM C35	168	27.975	9.379	3.29E-03	8.00E-01	0.051
SM C35:1	252	14.156	2.085	8.21E-11	1.99E-08	0.157
SM C35:2	203	59.767	12.310	2.43E-06	5.90E-04	0.106
SM C36:1	252	2.463	0.323	4.85E-13	1.18E-10	0.190
SM C36:2	252	3.718	0.612	4.73E-09	1.15E-06	0.129
SM C36:3	252	42.733	7.375	2.07E-08	5.04E-06	0.119
SM C37:1	128	7.883	3.656	3.30E-02	1.00E+00	0.036
SM C37:3	171	45.634	12.983	5.66E-04	1.38E-01	0.069
SM C38:1	195	1.022	0.201	8.35E-07	2.03E-04	0.120
SM C38:2	252	1.901	0.392	2.14E-06	5.20E-04	0.086
SM C38:3	151	39.487	14.688	8.01E-03	1.00E+00	0.047
SM C39:1	252	6.796	0.892	5.28E-13	1.28E-10	0.190
SM C39:2	152	15.451	3.504	1.98E-05	4.81E-03	0.116
SM C39:5	123	40.417	13.141	2.61E-03	6.33E-01	0.074
SM C40:2	252	1.439	0.208	3.88E-11	9.43E-09	0.161
SM C40:4	240	12.982	2.853	8.58E-06	2.08E-03	0.080
SM C40:5	216	22.230	6.436	6.67E-04	1.62E-01	0.053
SM C41:1	252	4.721	0.456	4.10E-21	9.96E-19	0.300
SM C41:2	252	5.158	0.693	1.57E-12	3.82E-10	0.182
SM C42:1	252	2.868	0.279	6.95E-21	1.69E-18	0.296
SM C42:2	252	1.144	0.170	1.19E-10	2.90E-08	0.154
SM C42:3	234	1.339	0.285	4.66E-06	1.13E-03	0.087
SM C42:4	216	1.980	0.807	1.50E-02	1.00E+00	0.027
SM C42:6	240	10.421	1.821	3.15E-08	7.66E-06	0.122
SM C43	144	-7.624	11.486	5.08E-01	1.00E+00	0.003
SM C43:1	248	22.328	4.123	1.46E-07	3.55E-05	0.107
SM C43:2	252	12.814	2.435	3.08E-07	7.48E-05	0.100
SM C44:6	210	15.343	3.757	6.33E-05	1.54E-02	0.075
NEFA 10:0	240	0.107	1.761	9.52E-01	1.00E+00	0.000
NEFA 12:0	208	0.182	0.297	5.41E-01	1.00E+00	0.002
NEFA 12:1	151	1.140	2.347	6.28E-01	1.00E+00	0.002

NEFA 13:0	98	6.134	5.637	2.79E-01	1.00E+00	0.012
NEFA 13:1	193	20.348	26.147	4.37E-01	1.00E+00	0.003
NEFA 14:0	251	0.369	0.242	1.28E-01	1.00E+00	0.009
NEFA 14:1	251	2.255	1.450	1.21E-01	1.00E+00	0.010
NEFA 14:2	114	0.102	0.268	7.06E-01	1.00E+00	0.001
NEFA 16:0	252	0.076	0.039	5.04E-02	1.00E+00	0.015
NEFA 16:1	223	0.131	0.108	2.23E-01	1.00E+00	0.007
NEFA 16:2	225	8.621	6.519	1.87E-01	1.00E+00	0.008
NEFA 17:0	235	2.176	1.288	9.25E-02	1.00E+00	0.012
NEFA 17:1	223	3.082	1.752	8.00E-02	1.00E+00	0.014
NEFA 17:2	153	70.756	37.886	6.38E-02	1.00E+00	0.023
NEFA 18:0	164	0.125	0.093	1.82E-01	1.00E+00	0.011
NEFA 18:1	252	0.037	0.028	1.78E-01	1.00E+00	0.007
NEFA 18:2	252	0.169	0.078	3.18E-02	1.00E+00	0.018
NEFA 18:3	252	0.521	0.240	3.09E-02	1.00E+00	0.019
NEFA 18:4	230	16.048	14.571	2.72E-01	1.00E+00	0.005
NEFA 19:0	236	12.894	10.849	2.36E-01	1.00E+00	0.006
NEFA 19:1	239	4.522	3.426	1.88E-01	1.00E+00	0.007
NEFA 20:0	132	4.017	6.547	5.41E-01	1.00E+00	0.003
NEFA 20:1	252	1.437	1.315	2.75E-01	1.00E+00	0.005
NEFA 20:2	203	1.564	2.037	4.43E-01	1.00E+00	0.003
NEFA 20:3	230	5.175	1.675	2.26E-03	5.48E-01	0.040
NEFA 20:4	223	1.944	0.776	1.30E-02	1.00E+00	0.028
NEFA 20:5	188	9.390	3.060	2.48E-03	6.02E-01	0.049
NEFA 22:0	93	24.444	11.333	3.37E-02	1.00E+00	0.049
NEFA 22:1	249	-4.243	8.867	6.33E-01	1.00E+00	0.001
NEFA 22:2	138	102.434	49.248	3.94E-02	1.00E+00	0.031
NEFA 22:3	224	72.302	41.665	8.41E-02	1.00E+00	0.013
NEFA 22:4	224	7.480	4.775	1.19E-01	1.00E+00	0.011
NEFA 22:5	252	6.917	2.383	4.03E-03	9.80E-01	0.033
NEFA 22:6	252	3.508	1.008	5.90E-04	1.43E-01	0.046
NEFA 24:0	207	55.445	17.057	1.35E-03	3.28E-01	0.049
NEFA 24:1	217	15.363	12.414	2.17E-01	1.00E+00	0.007
NEFA 24:2	165	262.363	99.683	9.31E-03	1.00E+00	0.041
NEFA 24:3	155	942.072	337.057	5.86E-03	1.00E+00	0.049
NEFA 24:4	223	143.038	81.396	8.03E-02	1.00E+00	0.014
NEFA 24:5	232	117.978	57.021	3.97E-02	1.00E+00	0.018
NEFA 24:6	193	247.534	86.586	4.73E-03	1.00E+00	0.041
NEFA 26:0	163	83.882	70.896	2.39E-01	1.00E+00	0.009
NEFA 26:1	206	132.994	62.784	3.54E-02	1.00E+00	0.022
NEFA 26:2	196	187.836	84.969	2.82E-02	1.00E+00	0.025
NEFA 26:3	170	52.120	98.597	5.98E-01	1.00E+00	0.002
NEFA 26:4	207	458.455	365.913	2.12E-01	1.00E+00	0.008
NEFA 26:5	195	788.666	446.253	7.88E-02	1.00E+00	0.016

NEFA 26:6	134	234.563	186.328	2.10E-01	1.00E+00	0.012
Pyruvate	252	-0.019	0.027	4.88E-01	1.00E+00	0.002
Lactate	252	-0.001	0.001	5.73E-01	1.00E+00	0.001
Acetoacetate	248	-0.021	0.152	8.91E-01	1.00E+00	0.000
Alpha-ketobutyrate	233	1.293	0.477	7.20E-03	1.00E+00	0.031
Fumarate	252	-2.313	4.843	6.33E-01	1.00E+00	0.001
3-Methyl-2-oxobutanoate	252	0.722	0.515	1.63E-01	1.00E+00	0.008
Succininate	252	-0.258	0.711	7.16E-01	1.00E+00	0.001
Methylmalonate	252	1.542	7.842	8.44E-01	1.00E+00	0.000
Taurine	252	0.065	0.066	3.25E-01	1.00E+00	0.004
3-Methyl-2-oxovalerate	252	0.799	0.481	9.79E-02	1.00E+00	0.011
4-Methyl-2-oxovalerate	252	0.524	0.286	6.79E-02	1.00E+00	0.013
Malate	252	-0.991	2.709	7.15E-01	1.00E+00	0.001
Alpha-ketoglutarate	251	-0.971	1.237	4.33E-01	1.00E+00	0.002
Alpha-aminoadipate	252	19.90611709	15.85881044	0.210584464	1	0.006304908
Isocitrate	239	-0.46631659	9.218630569	0.959699861	1	1.08717E-05
Citrate	252	-0.41071929	0.201773031	0.042858618	1	0.016382982
Sum AA	220	-0.01024548	0.00297492	0.000688503	0.167306186	0.051985037
Sum Carn	133	0.196205515	0.281534483	0.487110175	1	0.003741729
Sum LPC	231	0.093968764	0.036125836	0.009901787	1	0.028834103
Sum PCaa	251	0.028826155	0.005266063	1.08014E-07	2.62475E-05	0.107727762
Sum PCae	222	0.168605306	0.042619755	0.000103074	0.025046939	0.066661235
Sum SM	252	0.537666229	0.059122288	3.17538E-17	7.71618E-15	0.249147072
Sum NEFA	81	0.000113005	0.016588333	0.994582213	1	6.00283E-07

UBCS 8 years - ApoB

Metabolite	N	Estimate	Std..Error	P	P.adjusted	PartialRsquared
Ala	397	0.000127758	8.0248E-05	0.11218019	1	0.006321407
Arg	396	-9.6161E-05	0.0002356	0.68339027	1	0.000418082
Asn	359	-0.00061993	0.00070833	0.38206004	1	0.002117495
Asp	360	0.002062043	0.00136292	0.13117635	1	0.006301444
Cit	398	-0.0005733	0.00071704	0.42445782	1	0.001594429
Gln	397	-4.6431E-05	6.6649E-05	0.48643221	1	0.001212438
Glu	398	0.000320631	0.000341	0.34765894	1	0.002203715
Gly	396	-0.00011287	0.0001298	0.3850744	1	0.00189229
His	398	-1.7094E-05	0.0003239	0.95793745	1	6.95881E-06
Ile	358	-0.00036297	0.00056493	0.5209634	1	0.001145796
Leu	398	9.48741E-05	0.00029428	0.74732846	1	0.000259591
Lys	397	9.52297E-05	0.00019784	0.6305355	1	0.000580745
Met	398	-0.00029025	0.00119121	0.80762234	1	0.000148263
Orn	397	0.000232687	0.000409	0.56973277	1	0.000811318
Phe	398	-0.00018701	0.00058934	0.75116967	1	0.000251437
Pro	394	0.00017436	8.6521E-05	0.04456652	1	0.010150213
Ser	397	-0.00013349	0.00018204	0.46379804	1	0.001341968
Thr	398	-9.8807E-05	0.00023497	0.6743423	1	0.000441447
Trp	397	-0.00039885	0.00048899	0.41518596	1	0.001662847
Tyr	397	0.000438844	0.00042573	0.30326837	1	0.002654307
Val	398	0.000274935	0.00014366	0.05637218	1	0.009076217
Hpro	396	-0.00067854	0.000644	0.29269681	1	0.002777292
H1	396	2.57853E-05	1.128E-05	0.0227883	1	0.012922033
Carn	397	0.001097233	0.00093893	0.24327509	1	0.003406412
Carn C2	397	-0.00363772	0.00240619	0.13138405	1	0.005702327
Carn C3	398	-0.05876549	0.04466588	0.18905038	1	0.004307667
Carn C4	398	-0.10833369	0.07598105	0.15471819	1	0.00506377
Carn C5	391	-0.07474521	0.1229451	0.5435733	1	0.000938347
Carn C8:1	393	-0.03368186	0.04239698	0.42742367	1	0.001591567

Carn C10:1	396	-0.0781617	0.04212297	0.06426668	1	0.008560279
Carn C12:1	398	-0.0903446	0.04086344	0.02761711	1	0.012054823
Carn C14:1	397	0.090529716	0.05764171	0.11708983	1	0.006142421
Carn C16	396	0.170873301	0.15002618	0.25541681	1	0.00324704
Carn C18	394	0.837869369	0.31701153	0.00854805	1	0.017272921
Carn C18:1	398	-0.10371968	0.1244345	0.40505184	1	0.001732659
LPC C14	398	0.045600626	0.01345251	0.0007702	0.15635119	0.027924894
LPC C15	398	0.12638784	0.03137925	6.7574E-05	0.01371745	0.039074877
LPC C15:1	398	0.616610995	0.26532637	0.0206349	1	0.013322039
LPC C16	398	0.002790917	0.00050934	7.6233E-08	1.5475E-05	0.070064567
LPC C16:1	397	0.023110313	0.01282962	0.07241815	1	0.008077153
LPC C17	398	0.113993795	0.02366363	2.0799E-06	0.00042221	0.054764572
LPC C17:1	398	0.196636001	0.08582046	0.02247614	1	0.012947894
LPC C18	398	0.008919616	0.00131062	3.759E-11	7.6307E-09	0.104081007
LPC C18:1	398	0.004736015	0.00182035	0.00962554	1	0.016671104
LPC C18:2	398	0.001395815	0.00088098	0.11390713	1	0.006241393
LPC C18:3	397	0.055906039	0.03399502	0.10086515	1	0.006723228
LPC C18:6	398	0.248649679	0.09006552	0.00603615	1	0.018741722
LPC C20	398	0.244674176	0.09085347	0.00738215	1	0.017768036
LPC C20:1	398	0.173812473	0.08044867	0.0313332	1	0.011511951
LPC C20:2	398	0.178661167	0.07533281	0.01818997	1	0.013854608
LPC C20:3	396	0.030975859	0.01442277	0.03234961	1	0.011456983
LPC C20:4	396	0.01086438	0.00612867	0.07705257	1	0.007842326
LPC C20:5	396	0.131698344	0.04061959	0.00128745	0.26135319	0.025678232
LPC C22:4	398	0.080663466	0.05396354	0.13577311	1	0.005543078

LPC C22:5	398	0.06910986	0.02896625	0.01751008	1	0.014009549
LPC C22:6	397	0.036837729	0.0175403	0.03635019	1	0.01091854
PCaa C18	398	0.375952186	0.14398744	0.0093722	1	0.016728126
PCaa C18:1	398	0.335938839	0.17149635	0.05083312	1	0.009489436
PCaa C30	398	0.018386209	0.00400954	6.0888E-06	0.00123602	0.04986146
PCaa C30:2	398	0.072160302	0.0136899	2.2397E-07	4.5466E-05	0.065039093
PCaa C32	398	0.002617948	0.00089953	0.00381515	0.77447574	0.020725682
PCaa C32:1	398	0.003324506	0.00098462	0.00080734	0.16389073	0.02770045
PCaa C32:2	398	0.016546537	0.00385921	2.2745E-05	0.00461728	0.0439936
PCaa C32:3	398	0.171363812	0.03684685	4.5223E-06	0.00091802	0.051519372
PCaa C34	396	0.008356425	0.0026541	0.0017668	0.35865988	0.024200521
PCaa C34:1	397	0.000527554	0.00012759	4.3447E-05	0.00881978	0.041074108
PCaa C34:2	397	0.000294349	6.9312E-05	2.7101E-05	0.00550159	0.043357708
PCaa C34:3	398	0.005993029	0.00144507	4.124E-05	0.00837169	0.04121964
PCaa C34:4	398	0.038595266	0.00818878	3.388E-06	0.00068777	0.05252522
PCaa C34:5	398	0.233490547	0.06303374	0.00024233	0.0491922	0.033054023
PCaa C34:6	384	0.291376344	0.13901814	0.03674681	1	0.011252938
PCaa C36	397	0.022824944	0.00652649	0.00052373	0.10631728	0.029630222
PCaa C36:1	398	0.001410267	0.00035511	8.4943E-05	0.01724335	0.037939433
PCaa C36:2	397	0.000570566	0.00014278	7.6913E-05	0.01561329	0.03858194
PCaa C36:3	398	0.001562122	0.00028592	8.2902E-08	1.6829E-05	0.069280378
PCaa C36:4	398	0.000946567	0.00018792	7.2161E-07	0.00014649	0.059404415
PCaa C36:5	398	0.00420973	0.00109254	0.00013609	0.02762546	0.035670513
PCaa C36:6	398	0.097628923	0.02496973	0.0001087	0.022067	0.03669763
PCaa C38	398	0.026027217	0.00938058	0.00579031	1	0.018825667
PCaa C38:1	394	0.028800146	0.00844983	0.00072163	0.14649146	0.028311262
PCaa C38:2	398	0.00994254	0.00371274	0.00771728	1	0.017563886
PCaa C38:3	397	0.00407387	0.00071742	2.6453E-08	5.3699E-06	0.074597101
PCaa C38:4	398	0.001045517	0.00023378	1.0136E-05	0.00205763	0.047513483
PCaa C38:5	398	0.002070124	0.00048685	2.6476E-05	0.00537457	0.043112519
PCaa C38:6	398	0.001681407	0.00040257	3.6442E-05	0.00739773	0.041668137
PCaa C40	397	0.069272118	0.04605814	0.13338078	1	0.005624894
PCaa C40:1	391	0.074684233	0.08764259	0.39466042	1	0.001841153
PCaa C40:2	394	0.211390259	0.07732941	0.0065493	1	0.018465791
PCaa C40:3	390	0.05194275	0.04541611	0.25345373	1	0.003321508
PCaa C40:4	398	0.01436995	0.00499932	0.00426763	0.86632807	0.020169155
PCaa C40:5	397	0.00735379	0.00230327	0.00152266	0.30909909	0.024860025
PCaa C40:6	398	0.005415866	0.00121169	1.0254E-05	0.0020816	0.047374963
PCaa C42	398	0.017190317	0.04102535	0.67543212	1	0.000438217
PCaa C42:1	396	0.117365309	0.08251214	0.15570527	1	0.005034605
PCaa C42:2	392	0.155680849	0.13680376	0.25582828	1	0.003264298

PCaa C42:4	396	0.128779662	0.09166942	0.16086467	1	0.00492514
PCaa C42:5	395	0.129844055	0.05657795	0.02226446	1	0.013064394
PCaa C42:6	398	0.124371489	0.04809138	0.0100634	1	0.016361342
PCaa C43:6	398	-0.01069539	0.01862828	0.56619549	1	0.00082351
PCae C30	398	0.094335518	0.04182349	0.02464592	1	0.012539721
PCae C32	395	0.021751962	0.0066391	0.00114553	0.23254346	0.026287705
PCae C32:1	398	0.020520906	0.0088323	0.02066621	1	0.013292673
PCae C32:2	398	0.084369736	0.04885268	0.08494713	1	0.007406376
PCae C34	396	0.032195219	0.00886649	0.00031965	0.06488844	0.031944889
PCae C34:1	398	0.00745358	0.0027081	0.00619073	1	0.018562789
PCae C34:2	398	0.005528991	0.00202063	0.0064952	1	0.01837658
PCae C34:3	398	0.003966201	0.00229787	0.08512513	1	0.007384671
PCae C34:4	398	0.145403541	0.06242784	0.0203562	1	0.013363764
PCae C36	397	0.075674063	0.02210881	0.00068493	0.13904087	0.02843755
PCae C36:1	398	0.014652565	0.0049274	0.0031233	0.63402958	0.021587128
PCae C36:2	398	0.006346168	0.00244858	0.00990312	1	0.016497777
PCae C36:3	398	0.005984318	0.00356274	0.09380949	1	0.006994548
PCae C36:4	397	0.003972261	0.00133238	0.00304852	0.61884997	0.021700004
PCae C36:5	398	0.005697527	0.00189418	0.00279922	0.56824219	0.022049047
PCae C36:6	393	0.074687544	0.02813098	0.00825676	1	0.017474206
PCae C38	397	0.055862738	0.01206886	5.0071E-06	0.00101644	0.050933686
PCae C38:2	397	0.038610666	0.01099906	0.00049937	0.10137267	0.02986206
PCae C38:3	398	0.020709333	0.00612866	0.00079979	0.16235676	0.027611964
PCae C38:4	396	0.004983054	0.0015658	0.00157688	0.32010737	0.024678219
PCae C38:5	397	0.003188742	0.00122476	0.00957547	1	0.01667167
PCae C38:6	398	0.009404302	0.00267479	0.00048924	0.09931633	0.029909514
PCae C40	398	0.017399233	0.0049795	0.00052953	0.10749448	0.029504382
PCae C40:1	393	0.050723422	0.01119412	7.8127E-06	0.00158598	0.049266075
PCae C40:2	398	0.09510773	0.03059076	0.00201333	0.40870556	0.023531386
PCae C40:3	397	0.05154214	0.03086057	0.09568361	1	0.00693204
PCae C40:4	398	0.019954623	0.00993415	0.04525188	1	0.009952702
PCae C40:5	398	0.01374088	0.00740591	0.06428649	1	0.008505684
PCae C40:6	398	0.02077129	0.0065589	0.00166079	0.337141	0.024306039
PCae C42	398	0.064290008	0.05898739	0.27642591	1	0.002955521
PCae C42:1	396	0.065226407	0.03200316	0.04220831	1	0.010308737
PCae C42:2	398	0.094070039	0.03290475	0.00447758	0.90894901	0.019952894
PCae C42:3	397	0.070009317	0.03197633	0.02915447	1	0.011843576
PCae C42:4	398	0.013731211	0.02247368	0.54155807	1	0.000931189
PCae C42:5	398	0.001930962	0.01287239	0.88083509	1	5.62062E-05
PCae C42:6	398	0.014308775	0.01953698	0.46436341	1	0.001337033

SM C32:2	322	0.299562214	0.04342515	2.8498E-11	5.7851E-09	0.129923174
SM C35	397	0.056451233	0.01881947	0.00287529	0.58368322	0.022093041
SM C35:1	398	0.018336376	0.0048721	0.000193	0.03917906	0.034221135
SM C36	397	0.017015254	0.00651661	0.00937172	1	0.016775382
SM C36:1	398	0.003235766	0.00079418	5.5812E-05	0.01132986	0.03992541
SM C36:2	398	0.004845281	0.00149474	0.00128997	0.26186409	0.025667762
SM C37:1	397	0.026649987	0.00899321	0.00322833	0.65535039	0.021558762
SM C38:1	398	0.002278696	0.00060387	0.00018574	0.0377062	0.034397803
SM C38:2	398	0.002913208	0.00089156	0.00118008	0.23955706	0.02605813
SM C38:3	398	0.049283519	0.02226522	0.02743713	1	0.012103302
SM C39:1	398	0.019490681	0.00436605	1.051E-05	0.00213357	0.047542733
SM C39:2	396	0.044171868	0.0135497	0.00121153	0.24594079	0.025996445
SM C39:5	398	0.055268982	0.02251775	0.01454121	1	0.014810465
SM C40:1	395	0.001857956	0.00064148	0.00398732	0.80942583	0.020661241
SM C40:2	398	0.004692618	0.00091295	4.3338E-07	8.7977E-05	0.062183747
SM C40:3	395	0.006012656	0.00187191	0.00142709	0.28969892	0.025282502
SM C40:4	398	0.007784336	0.00238083	0.00117171	0.2378567	0.026011402
SM C41:1	398	0.009007137	0.00223586	6.7371E-05	0.01367625	0.038840415
SM C41:2	398	0.010590025	0.00346066	0.00236391	0.47987327	0.022901813
SM C41:3	390	0.027285106	0.01525477	0.07445895	1	0.00811819
SM C42:1	397	0.004170347	0.00113806	0.00028189	0.05722399	0.032335358
SM C42:2	398	0.001311808	0.00056311	0.02033281	1	0.013352435
SM C42:3	398	0.003071877	0.00135137	0.02355389	1	0.012748203
SM C42:4	397	0.010748468	0.00340088	0.00169705	0.34450035	0.024396844
SM C42:6	398	0.026525381	0.00704456	0.00019164	0.03890393	0.034151264
SM C43	396	0.068556359	0.03294227	0.03807279	1	0.010752358
SM C43:1	398	0.023355341	0.0138492	0.0925098	1	0.007041664
SM C43:2	398	0.006733922	0.00896201	0.45286929	1	0.001407286
SM C43:3	396	0.020450128	0.03779719	0.58878118	1	0.000735137
SM C44:2	394	0.089069142	0.04727401	0.06029454	1	0.00883437
SM C44:6	396	0.030841008	0.01503615	0.04091908	1	0.010447193
NEFA 10:0	321	-0.00123899	0.0072518	0.86444834	1	9.08349E-05
NEFA 12:0	394	-0.00101642	0.00150131	0.49879104	1	0.001158532
NEFA 12:1	391	-0.00993386	0.00674652	0.14171451	1	0.005478679

NEFA 14:0	397	-0.00043933	0.00084697	0.60425382	1	0.000673972
NEFA 14:1	395	0.001328604	0.00541981	0.80647791	1	0.000151391
NEFA 15:0	390	-0.00248802	0.00426892	0.56035285	1	0.000868203
NEFA 15:1	332	-0.08656089	0.05995161	0.14973676	1	0.00623448
NEFA 16:0	397	-0.00010557	0.00011475	0.35816036	1	0.002119181
NEFA 16:1	396	-0.0006177	0.00046778	0.18743981	1	0.00436721
NEFA 16:2	394	-0.05817911	0.04392686	0.18612786	1	0.004411554
NEFA 17:0	395	-0.00356465	0.00438558	0.41682112	1	0.001662491
NEFA 17:1	395	-0.01015887	0.00631811	0.10866515	1	0.006485215
NEFA 18:0	394	-0.00018824	0.00030143	0.532674	1	0.000985165
NEFA 18:1	398	-8.8397E-05	7.3378E-05	0.22904643	1	0.003616497
NEFA 18:2	397	-0.00019153	0.00028176	0.49707147	1	0.001157923
NEFA 18:3	397	-0.00153752	0.00223474	0.49185305	1	0.001186506
NEFA 19:1	398	-0.01807845	0.0140711	0.1996193	1	0.004112332
NEFA 20:0	357	-0.06060694	0.0246651	0.01448306	1	0.016675321
NEFA 20:2	348	-0.01368753	0.011421	0.23156553	1	0.004121224
NEFA 20:3	282	0.008652449	0.01485768	0.56079927	1	0.001199331
NEFA 20:4	398	0.012460226	0.00508471	0.01469856	1	0.014711678
NEFA 20:5	383	-0.00803514	0.03361105	0.81118643	1	0.00014859
NEFA 22:4	398	-0.00093854	0.02654805	0.97181656	1	3.12249E-06
NEFA 22:5	398	-0.00318349	0.01364477	0.81564022	1	0.00013602
NEFA 22:6	398	0.000559448	0.0077065	0.94216582	1	1.31647E-05
NEFA 24:0	395	-0.01329107	0.08751216	0.8793624	1	5.80866E-05
NEFA 24:2	351	-0.35923895	0.36367038	0.32393168	1	0.002758117
NEFA 24:4	392	-0.26468484	0.4298588	0.53842165	1	0.000960487
NEFA 24:5	397	0.207996724	0.40092786	0.6042003	1	0.000673604
NEFA 26:1	398	0.10527793	0.1539072	0.4943551	1	0.001167418

SUM_AA	331	1.17877E-05	1.7736E-05	0.50677152	1	0.001324616
SUM_Carn	377	0.000596567	0.00089957	0.50763345	1	0.001154673
SUM_LPC	393	0.001002123	0.0002403	3.7537E-05	0.00761996	0.042298292
SUM_PCaa	395	0.000132827	2.3785E-05	4.3922E-08	8.9162E-06	0.072842317
SUM_PCae	395	0.00072784	0.00020659	0.00047684	0.09679863	0.030182582
SUM_SM	321	0.003024851	0.00034534	1.2061E-16	2.4484E-14	0.192389403
SUM_NEFA	271	-3.617E-05	4.3314E-05	0.40443525	1	0.002574696

Supplemental Table 7 Associations of metabolites with HDL or ApoB for each study follow-up visit were calculated in linear models adjusted for child age and sex. Statistical significance was evaluated using Bonferroni corrected p-values (p.adjust) < 0.05 (with dark grey background). AA, amino acids; Carn, Acylcarnitines; lyso.PC, lyso-phosphatidylcholine; NEFA, nonesterified fatty acid; PCaa, Diacyl-phosphatidylcholine; PCae, Alkyl-acyl- [illegible]

CHOP 5.5 years - HDL

Metabolite	N	Estimate	Std..Error	P	P.adjusted	PartialRsquared
Ala	383	0.002	0.009	8.48E-01	1.00E+00	0.000
Arg	381	0.083	0.040	3.70E-02	1.00E+00	0.011
Asn	383	0.173	0.069	1.31E-02	1.00E+00	0.016
Asp	381	-0.142	0.218	5.15E-01	1.00E+00	0.001
Cit	383	0.372	0.089	3.68E-05	8.58E-03	0.044
Gln	383	0.002	0.006	7.87E-01	1.00E+00	0.000
Glu	383	0.003	0.008	7.51E-01	1.00E+00	0.000
Gly	383	0.021	0.015	1.66E-01	1.00E+00	0.005
His	383	0.197	0.035	4.89E-08	1.14E-05	0.075
Ile	383	0.030	0.043	4.81E-01	1.00E+00	0.001
Leu	383	0.048	0.027	7.43E-02	1.00E+00	0.008
Lys	383	0.045	0.021	3.34E-02	1.00E+00	0.012
Met	383	0.209	0.153	1.72E-01	1.00E+00	0.005
Orn	383	0.034	0.037	3.57E-01	1.00E+00	0.002
Phe	383	0.111	0.064	8.17E-02	1.00E+00	0.008
Pro	383	0.003	0.013	8.19E-01	1.00E+00	0.000
Ser	382	-0.009	0.032	7.83E-01	1.00E+00	0.000
Thr	383	0.009	0.024	7.13E-01	1.00E+00	0.000
Trp	382	0.157	0.063	1.32E-02	1.00E+00	0.016
Tyr	383	0.092	0.045	3.90E-02	1.00E+00	0.011
Val	383	0.019	0.013	1.41E-01	1.00E+00	0.006
H1	382	-0.002	0.001	8.82E-02	1.00E+00	0.008
Carn	388	0.192	0.078	1.36E-02	1.00E+00	0.016
Carn C2	388	0.596	0.198	2.72E-03	6.34E-01	0.023
Carn C3	388	20.831	7.338	4.77E-03	1.00E+00	0.020
Carn C3:1	272	-3.488	135.557	9.79E-01	1.00E+00	0.000
Carn C4	388	18.615	6.695	5.70E-03	1.00E+00	0.020
Carn C4:0:OH	272	-24.772	9.555	1.00E-02	1.00E+00	0.024
Carn C4:1	346	4.901	22.885	8.31E-01	1.00E+00	0.000
Carn C5	388	55.905	19.404	4.19E-03	9.75E-01	0.021
Carn C5:0:OH	313	4944.973	1635.579	2.71E-03	6.31E-01	0.029
Carn C6	314	57.976	25.901	2.59E-02	1.00E+00	0.016
Carn C6:1	209	-607.983	233.190	9.80E-03	1.00E+00	0.032
Carn C8	325	1.959	4.823	6.85E-01	1.00E+00	0.001
Carn C8:1	388	-23.294	13.303	8.07E-02	1.00E+00	0.008
Carn C9	388	37.740	50.743	4.57E-01	1.00E+00	0.001
Carn C10	387	-0.818	3.174	7.97E-01	1.00E+00	0.000

Carn C10:1	388	13.002	4.151	1.87E-03	4.35E-01	0.025
Carn C10:2	388	73.872	37.692	5.07E-02	1.00E+00	0.010
Carn C12::DC	262	-10.408	9.471	2.73E-01	1.00E+00	0.005
Carn C12	387	23.223	8.396	5.95E-03	1.00E+00	0.020
Carn C12:1	388	8.595	3.095	5.75E-03	1.00E+00	0.020
Carn C14	388	48.538	23.859	4.26E-02	1.00E+00	0.011
Carn C14:1	386	8.724	6.036	1.49E-01	1.00E+00	0.005
Carn C14:2	388	94.033	27.271	6.27E-04	1.46E-01	0.030
Carn C14:2:OH	252	244.288	81.463	2.99E-03	6.96E-01	0.035
Carn C15	388	172.049	67.403	1.11E-02	1.00E+00	0.017
Carn C16	387	59.508	25.953	2.24E-02	1.00E+00	0.014
Carn C16:OH	387	59.612	27.717	3.21E-02	1.00E+00	0.012
Carn C16:1	285	455.836	148.815	2.40E-03	5.60E-01	0.032
Carn C16:2	272	249.747	99.387	1.26E-02	1.00E+00	0.023
Carn C16:2:OH	252	480.548	115.647	4.48E-05	1.04E-02	0.063
Carn C18	388	185.323	49.522	2.10E-04	4.90E-02	0.035
Carn C18:1	388	43.492	16.276	7.86E-03	1.00E+00	0.018
Carn C18:1:OH	324	77.789	106.066	4.64E-01	1.00E+00	0.002
Carn C18:2	388	74.425	22.564	1.06E-03	2.48E-01	0.028
Carn C18:2:OH	251	754.009	316.860	1.81E-02	1.00E+00	0.022
Carn C20	386	430.115	160.512	7.69E-03	1.00E+00	0.018
Carn C20:1	312	16059.920	4391.483	3.00E-04	6.99E-02	0.042
Carn C20:3	388	259.499	106.283	1.51E-02	1.00E+00	0.015
Carn C20:4	388	2915.356	1209.793	1.64E-02	1.00E+00	0.015
Carn C22	286	4731.179	1746.836	7.17E-03	1.00E+00	0.025
Carn C22:5	273	900.745	267.561	8.72E-04	2.03E-01	0.040
Carn C22:6	251	5016.463	1004.253	1.11E-06	2.59E-04	0.088
LPC C14	244	-0.462	1.029	6.54E-01	1.00E+00	0.001
LPC C15	244	0.719	2.763	7.95E-01	1.00E+00	0.000
LPC C16	387	-0.001	0.026	9.75E-01	1.00E+00	0.000
LPC C16:1	388	-0.073	0.713	9.19E-01	1.00E+00	0.000
LPC C17	386	0.762	1.096	4.87E-01	1.00E+00	0.001
LPC C18	388	0.092	0.064	1.49E-01	1.00E+00	0.005
LPC C18:1	388	0.231	0.138	9.42E-02	1.00E+00	0.007
LPC C18:2	388	0.208	0.072	3.85E-03	8.98E-01	0.021
LPC C18:3	347	0.282	2.443	9.08E-01	1.00E+00	0.000
LPC C20:1	196	2.023	3.891	6.04E-01	1.00E+00	0.001
LPC C20:3	386	1.488	0.735	4.37E-02	1.00E+00	0.011
LPC C20:4	388	1.282	0.325	9.30E-05	2.17E-02	0.039
LPC C20:5	317	-5.979	3.595	9.73E-02	1.00E+00	0.009

LPC C22:6	314	-1.954	1.121	8.23E-02	1.00E+00	0.010
PCaa C20:4	214	57.162	17.633	1.38E-03	3.22E-01	0.048
PCaa C28:2	317	6.806	17.040	6.90E-01	1.00E+00	0.001
PCaa C30	388	0.456	0.451	3.12E-01	1.00E+00	0.003
PCaa C30:2	387	11.167	3.044	2.79E-04	6.50E-02	0.034
PCaa C30:3	387	47.581	9.625	1.15E-06	2.68E-04	0.060
PCaa C30:4	163	-6.072	34.416	8.60E-01	1.00E+00	0.000
PCaa C32	388	0.070	0.192	7.16E-01	1.00E+00	0.000
PCaa C32:1	388	-0.050	0.136	7.16E-01	1.00E+00	0.000
PCaa C32:2	388	2.461	0.672	2.86E-04	6.68E-02	0.034
PCaa C34:1	388	0.057	0.013	2.67E-05	6.22E-03	0.045
PCaa C34:2	388	0.070	0.009	7.55E-14	1.76E-11	0.136
PCaa C34:3	386	-0.025	0.153	8.73E-01	1.00E+00	0.000
PCaa C34:4	388	2.072	1.212	8.82E-02	1.00E+00	0.008
PCaa C36	271	-0.270	0.358	4.50E-01	1.00E+00	0.002
PCaa C36:1	388	0.096	0.049	5.05E-02	1.00E+00	0.010
PCaa C36:2	388	0.089	0.012	2.53E-13	5.90E-11	0.130
PCaa C36:3	388	0.144	0.024	3.82E-09	8.91E-07	0.086
PCaa C36:4	388	0.108	0.018	3.60E-09	8.39E-07	0.087
PCaa C36:5	386	0.307	0.197	1.20E-01	1.00E+00	0.006
PCaa C36:6	318	-1.737	1.116	1.21E-01	1.00E+00	0.008
PCaa C38	312	0.802	0.671	2.33E-01	1.00E+00	0.005
PCaa C38:3	388	0.127	0.065	5.01E-02	1.00E+00	0.010
PCaa C38:4	388	0.085	0.025	7.10E-04	1.65E-01	0.029
PCaa C38:5	388	0.127	0.069	6.85E-02	1.00E+00	0.009
PCaa C38:6	388	0.124	0.042	3.24E-03	7.55E-01	0.022
PCaa C40	277	0.952	1.366	4.87E-01	1.00E+00	0.002
PCaa C40:4	387	0.254	0.595	6.69E-01	1.00E+00	0.000
PCaa C40:5	388	0.201	0.211	3.40E-01	1.00E+00	0.002
PCaa C40:6	388	0.051	0.097	5.97E-01	1.00E+00	0.001
PCaa C42	244	1.545	2.048	4.51E-01	1.00E+00	0.002
PCaa C42:1	212	0.658	5.019	8.96E-01	1.00E+00	0.000

PCaa C42:6	196	2.984	2.806	2.89E-01	1.00E+00	0.006
PCaa C43:6	252	2.465	1.739	1.58E-01	1.00E+00	0.008
PCaa C44:12	388	3.547	1.229	4.11E-03	9.58E-01	0.021
PCae C30:2	277	22.996	7.530	2.48E-03	5.78E-01	0.033
PCae C32	388	3.496	0.882	8.83E-05	2.06E-02	0.039
PCae C32:1	388	3.334	0.719	4.85E-06	1.13E-03	0.053
PCae C32:2	204	8.100	3.119	1.01E-02	1.00E+00	0.033
PCae C34	388	3.525	1.244	4.86E-03	1.00E+00	0.020
PCae C34:1	388	0.857	0.260	1.05E-03	2.45E-01	0.028
PCae C34:2	388	1.298	0.216	4.29E-09	1.00E-06	0.086
PCae C34:3	388	1.993	0.244	4.76E-15	1.11E-12	0.147
PCae C34:4	244	13.315	7.269	6.82E-02	1.00E+00	0.014
PCae C36	255	0.781	1.442	5.89E-01	1.00E+00	0.001
PCae C36:1	387	-0.018	0.123	8.83E-01	1.00E+00	0.000
PCae C36:2	387	0.738	0.146	6.48E-07	1.51E-04	0.063
PCae C36:3	388	1.879	0.250	4.45E-13	1.04E-10	0.127
PCae C36:4	387	0.751	0.133	3.09E-08	7.21E-06	0.077
PCae C36:5	388	1.072	0.169	5.89E-10	1.37E-07	0.095
PCae C36:6	172	6.477	2.387	7.36E-03	1.00E+00	0.042
PCae C38	313	-0.882	0.565	1.20E-01	1.00E+00	0.008
PCae C38:2	306	0.085	0.168	6.11E-01	1.00E+00	0.001
PCae C38:3	386	-0.043	0.170	8.01E-01	1.00E+00	0.000
PCae C38:4	388	1.192	0.170	1.08E-11	2.53E-09	0.113
PCae C38:5	388	0.764	0.136	4.14E-08	9.65E-06	0.075
PCae C38:6	388	1.908	0.291	1.74E-10	4.04E-08	0.101
PCae C40	388	0.009	0.243	9.72E-01	1.00E+00	0.000
PCae C40:1	232	2.392	0.932	1.09E-02	1.00E+00	0.028
PCae C40:3	317	-0.067	0.242	7.83E-01	1.00E+00	0.000
PCae C40:4	348	0.060	0.385	8.76E-01	1.00E+00	0.000
PCae C40:5	388	0.305	0.405	4.52E-01	1.00E+00	0.001
PCae C40:6	388	1.710	0.513	9.48E-04	2.21E-01	0.028
PCae C42:2	325	-0.685	1.303	5.99E-01	1.00E+00	0.001
PCae C42:3	277	0.705	1.157	5.43E-01	1.00E+00	0.001
PCae C42:4	193	4.730	2.216	3.41E-02	1.00E+00	0.023
PCae C42:5	314	-0.677	0.896	4.50E-01	1.00E+00	0.002
PCae C42:6	387	-0.856	1.369	5.32E-01	1.00E+00	0.001
SM C21:2	348	138.496	30.383	7.18E-06	1.67E-03	0.057
SM C32:1	388	1.612	0.294	7.68E-08	1.79E-05	0.073

SM C32:2	348	5.125	4.114	2.14E-01	1.00E+00	0.004
SM C33:1	388	2.373	0.529	9.66E-06	2.25E-03	0.050
SM C33:3	184	20.479	11.214	6.95E-02	1.00E+00	0.018
SM C34:1	387	0.103	0.031	1.09E-03	2.54E-01	0.027
SM C34:2	387	1.195	0.215	5.52E-08	1.29E-05	0.074
SM C34:4	213	9.265	50.912	8.56E-01	1.00E+00	0.000
SM C35:1	387	2.152	0.903	1.76E-02	1.00E+00	0.015
SM C35:2	197	10.641	6.787	1.19E-01	1.00E+00	0.013
SM C36:1	386	0.229	0.155	1.42E-01	1.00E+00	0.006
SM C36:2	387	0.680	0.289	1.91E-02	1.00E+00	0.014
SM C36:3	183	-0.082	3.453	9.81E-01	1.00E+00	0.000
SM C37:1	197	2.182	0.807	7.49E-03	1.00E+00	0.036
SM C37:3	206	28.947	4.622	2.23E-09	5.19E-07	0.160
SM C38:2	388	1.032	0.181	2.34E-08	5.46E-06	0.078
SM C39:1	314	2.045	0.479	2.56E-05	5.98E-03	0.056
SM C40:2	237	0.263	0.111	1.84E-02	1.00E+00	0.024
SM C40:4	307	5.767	1.142	7.67E-07	1.79E-04	0.077
SM C40:5	348	-4.395	2.272	5.39E-02	1.00E+00	0.011
SM C41:1	387	0.683	0.165	4.33E-05	1.01E-02	0.043
SM C41:2	387	1.286	0.233	6.08E-08	1.42E-05	0.074
SM C42:1	388	0.505	0.112	8.94E-06	2.08E-03	0.050
SM C42:2	387	0.193	0.067	3.98E-03	9.28E-01	0.021
SM C42:3	318	0.017	0.147	9.06E-01	1.00E+00	0.000
SM C42:6	388	3.179	0.802	8.76E-05	2.04E-02	0.039
SM C43	175	4.621	4.175	2.70E-01	1.00E+00	0.007
SM C43:1	204	1.744	1.505	2.48E-01	1.00E+00	0.007
SM C43:2	317	0.945	0.979	3.35E-01	1.00E+00	0.003
SM C44:6	233	4.018	2.111	5.82E-02	1.00E+00	0.016
SM C47:6	325	359.481	128.098	5.32E-03	1.00E+00	0.024
NEFA 12:0	181	0.280	0.296	3.46E-01	1.00E+00	0.005

NEFA 14:0	323	0.081	0.087	3.48E-01	1.00E+00	0.003
NEFA 14:1	385	0.386	0.138	5.28E-03	1.00E+00	0.020
NEFA 15:0	181	2.800	1.223	2.32E-02	1.00E+00	0.029
NEFA 16:0	248	0.023	0.014	1.11E-01	1.00E+00	0.010
NEFA 16:1	385	0.069	0.040	8.69E-02	1.00E+00	0.008
NEFA 16:2	304	2.554	1.386	6.64E-02	1.00E+00	0.011
NEFA 17:0	168	0.731	0.444	1.02E-01	1.00E+00	0.016
NEFA 17:1	385	1.090	0.362	2.79E-03	6.50E-01	0.023
NEFA 17:2	168	32.395	15.927	4.36E-02	1.00E+00	0.024
NEFA 18:1	385	0.018	0.007	6.10E-03	1.00E+00	0.020
NEFA 18:2	385	0.054	0.015	4.73E-04	1.10E-01	0.032
NEFA 18:3	385	0.312	0.217	1.51E-01	1.00E+00	0.005
NEFA 18:4	168	18.229	9.128	4.75E-02	1.00E+00	0.023
NEFA 19:1	302	0.781	1.759	6.58E-01	1.00E+00	0.001
NEFA 20:1	385	1.027	0.457	2.54E-02	1.00E+00	0.013
NEFA 20:2	385	0.920	0.247	2.29E-04	5.33E-02	0.035
NEFA 20:3	385	1.116	0.492	2.37E-02	1.00E+00	0.013
NEFA 20:4	384	0.379	0.165	2.20E-02	1.00E+00	0.014
NEFA 20:5	301	0.240	0.901	7.90E-01	1.00E+00	0.000
NEFA 22:3	230	67.414	27.683	1.57E-02	1.00E+00	0.025
NEFA 22:4	385	5.318	2.096	1.16E-02	1.00E+00	0.017
NEFA 22:5	382	2.579	1.068	1.62E-02	1.00E+00	0.015
NEFA 22:6	385	1.102	0.431	1.09E-02	1.00E+00	0.017
NEFA 24:0	168	23.734	7.446	1.72E-03	4.00E-01	0.058
NEFA 24:1	385	7.704	2.949	9.34E-03	1.00E+00	0.018
NEFA 24:2	168	-99.511	55.523	7.49E-02	1.00E+00	0.019
NEFA 24:3	241	-8.288	38.348	8.29E-01	1.00E+00	0.000
NEFA 24:4	385	107.675	36.434	3.32E-03	7.73E-01	0.022
NEFA 24:6	162	313.458	113.252	6.32E-03	1.00E+00	0.045
NEFA 26:0	329	2.266	4.605	6.23E-01	1.00E+00	0.001
NEFA 26:1	311	21.442	5.431	9.77E-05	2.28E-02	0.048
NEFA 26:2	385	25.515	6.278	5.85E-05	1.36E-02	0.041
NEFA 26:3	303	31.058	20.582	1.32E-01	1.00E+00	0.008
NEFA 26:4	385	176.783	79.528	2.68E-02	1.00E+00	0.013

Pyruvate	380	-0.001	0.004	7.19E-01	1.00E+00	0.000
Lactic_acid	385	0.000	0.000	3.52E-01	1.00E+00	0.002
Fumarate	378	3.813	2.576	1.40E-01	1.00E+00	0.006
3-Methyl-2-oxobutanoate	380	-0.003	0.053	9.48E-01	1.00E+00	0.000
Succininate	383	-0.420	0.222	5.95E-02	1.00E+00	0.009
Methylmalonate	385	-16.039	10.369	1.23E-01	1.00E+00	0.006
Taurine	383	-0.001	0.019	9.53E-01	1.00E+00	0.000
3-Methyl-2-oxovalerate	384	-0.034	0.059	5.63E-01	1.00E+00	0.001
4-Methyl-2-oxovalerate	379	0.021	0.030	4.74E-01	1.00E+00	0.001
Malate	381	-1.004	0.417	1.65E-02	1.00E+00	0.015
Alpha-ketoglutarate	374	0.130	0.135	3.35E-01	1.00E+00	0.003
Alpha-aminoadipate	384	2.436	4.591	5.96E-01	1.00E+00	0.001
Isocitrate	385	-0.033	0.134	8.04E-01	1.00E+00	0.000
Citrate	385	-0.015	0.010	1.41E-01	1.00E+00	0.006
Beta-hydroxybutyrate	148	-0.022	0.032	4.85E-01	1.00E+00	0.003
Sum AA	380	0.003	0.002	5.12E-02	1.00E+00	0.010
Sum Carn	385	0.246	0.070	5.11E-04	1.19E-01	0.031
Sum LPC	204	0.055	0.025	3.18E-02	1.00E+00	0.023
Sum PCaa	309	0.018	0.003	9.08E-09	2.12E-06	0.103
Sum PCae	272	0.137	0.026	2.18E-07	5.07E-05	0.095
Sum SM	346	0.109	0.027	7.30E-05	1.70E-02	0.045
Sum NEFA	99	0.011	0.005	3.51E-02	1.00E+00	0.042

CHOP 8 years - HDL

Metabolite	N	Estimate	Std..Error	P	P.adjusted	PartialRsquared
Ala	350	-0.040	0.012	7.03E-04	1.34E-01	0.033
Arg	350	-0.059	0.054	2.78E-01	1.00E+00	0.003
Asn	350	0.112	0.089	2.09E-01	1.00E+00	0.005
Asp	350	0.022	0.278	9.37E-01	1.00E+00	0.000
Cit	350	0.460	0.120	1.58E-04	2.99E-02	0.040
Gln	349	-0.004	0.006	4.47E-01	1.00E+00	0.002
Glu	350	0.002	0.009	8.40E-01	1.00E+00	0.000
Gly	350	-0.039	0.019	4.15E-02	1.00E+00	0.012
His	350	0.005	0.035	8.91E-01	1.00E+00	0.000
Ile	350	0.035	0.085	6.77E-01	1.00E+00	0.000
Leu	350	0.068	0.048	1.59E-01	1.00E+00	0.006
Lys	350	0.015	0.055	7.83E-01	1.00E+00	0.000
Met	350	0.200	0.199	3.15E-01	1.00E+00	0.003
Orn	350	0.035	0.058	5.45E-01	1.00E+00	0.001
Phe	349	0.151	0.095	1.13E-01	1.00E+00	0.007
Pro	350	-0.010	0.022	6.53E-01	1.00E+00	0.001
Ser	349	-0.017	0.029	5.59E-01	1.00E+00	0.001
Thr	350	-0.046	0.037	2.12E-01	1.00E+00	0.004
Trp	350	0.042	0.089	6.37E-01	1.00E+00	0.001
Tyr	349	-0.003	0.069	9.70E-01	1.00E+00	0.000
Val	350	0.037	0.023	1.10E-01	1.00E+00	0.007
H1	350	0.000	0.001	9.82E-01	1.00E+00	0.000
Carn	350	-0.031	0.042	4.52E-01	1.00E+00	0.002
Carn C2	350	0.316	0.329	3.36E-01	1.00E+00	0.003
Carn C3	347	5.952	2.697	2.80E-02	1.00E+00	0.014
Carn C3:1	285	78.338	41.847	6.22E-02	1.00E+00	0.012
Carn C4:0:OH	237	14.366	11.268	2.04E-01	1.00E+00	0.007
Carn C5	350	2162.176	596.724	3.34E-04	6.35E-02	0.036
Carn C5:0:OH	350	42.168	10.740	1.04E-04	1.98E-02	0.042
Carn C6	350	17.710	17.485	3.12E-01	1.00E+00	0.003
Carn C6:1	350	-23.586	22.692	2.99E-01	1.00E+00	0.003
Carn C8	350	4.906	4.970	3.24E-01	1.00E+00	0.003
Carn C8:1	350	-42.871	12.449	6.44E-04	1.22E-01	0.033
Carn C9	350	37.344	46.769	4.25E-01	1.00E+00	0.002
Carn C10	350	6.301	2.915	3.13E-02	1.00E+00	0.013

Carn C10:1	349	7.745	4.844	1.11E-01	1.00E+00	0.007
Carn C10:2	350	-119.701	34.620	6.13E-04	1.16E-01	0.033
Carn C12	350	10.043	7.502	1.82E-01	1.00E+00	0.005
Carn C12:1	350	2.627	3.142	4.04E-01	1.00E+00	0.002
Carn C14	350	99.050	31.216	1.64E-03	3.12E-01	0.028
Carn C14:1	349	6.460	5.169	2.12E-01	1.00E+00	0.004
Carn C14:2	350	52.429	23.919	2.91E-02	1.00E+00	0.014
Carn C14:2:OH	348	-33.794	74.430	6.50E-01	1.00E+00	0.001
Carn C15	350	267.085	172.141	1.22E-01	1.00E+00	0.007
Carn C16	350	98.386	38.372	1.08E-02	1.00E+00	0.019
Carn C16:0:OH	287	612.637	474.102	1.97E-01	1.00E+00	0.006
Carn C16:1	350	53.520	33.284	1.09E-01	1.00E+00	0.007
Carn C16:2	350	180.601	86.176	3.68E-02	1.00E+00	0.012
Carn C16:2:OH	301	723.695	165.353	1.67E-05	3.17E-03	0.060
Carn C18	350	236.211	84.410	5.42E-03	1.00E+00	0.022
Carn C18:1	350	46.934	25.376	6.52E-02	1.00E+00	0.010
Carn C18:1:OH	350	2.006	115.836	9.86E-01	1.00E+00	0.000
Carn C18:2	348	125.195	41.257	2.59E-03	4.93E-01	0.026
Carn C18:2:OH	230	416.685	285.629	1.46E-01	1.00E+00	0.009
Carn C20	344	-221.924	166.132	1.82E-01	1.00E+00	0.005
Carn C20:1	349	-17974.584	11767.427	1.28E-01	1.00E+00	0.007
Carn C20:3	350	452.259	449.097	3.15E-01	1.00E+00	0.003
Carn C20:4	301	129.221	5868.868	9.82E-01	1.00E+00	0.000
Carn C22	220	20527.982	5535.082	2.65E-04	5.03E-02	0.060
Carn C22:5	251	-128.094	883.075	8.85E-01	1.00E+00	0.000
Carn C22:6	183	-501.936	640.830	4.35E-01	1.00E+00	0.003
LPC C16	350	0.012	0.019	5.23E-01	1.00E+00	0.001
LPC C16:1	350	0.054	0.685	9.37E-01	1.00E+00	0.000
LPC C17	287	0.239	1.094	8.28E-01	1.00E+00	0.000
LPC C18	350	0.065	0.052	2.14E-01	1.00E+00	0.004
LPC C18:1	350	0.267	0.121	2.82E-02	1.00E+00	0.014
LPC C18:2	350	0.307	0.064	2.73E-06	5.20E-04	0.061
LPC C18:3	219	-0.814	3.643	8.23E-01	1.00E+00	0.000
LPC C18:6	178	-14.968	5.514	7.31E-03	1.00E+00	0.040
LPC C20:3	283	2.292	0.994	2.18E-02	1.00E+00	0.019
LPC C20:4	350	1.280	0.335	1.58E-04	2.99E-02	0.040
LPC C20:5	166	-2.729	3.614	4.51E-01	1.00E+00	0.003

LPC C22:5	152	2.809	4.540	5.37E-01	1.00E+00	0.003
LPC C22:6	250	1.644	1.439	2.54E-01	1.00E+00	0.005
PCaa C30:5	180	-570.257	408.203	1.64E-01	1.00E+00	0.011
PCaa C32	350	1.144	0.222	4.39E-07	8.34E-05	0.071
PCaa C32:1	287	0.226	0.150	1.32E-01	1.00E+00	0.008
PCaa C34:1	350	0.044	0.015	3.29E-03	6.25E-01	0.025
PCaa C34:2	284	0.058	0.009	2.83E-09	5.37E-07	0.116
PCaa C34:3	350	0.246	0.158	1.19E-01	1.00E+00	0.007
PCaa C34:4	301	1.982	1.275	1.21E-01	1.00E+00	0.008
PCaa C36:2	350	0.072	0.010	7.37E-13	1.40E-10	0.136
PCaa C36:3	350	0.175	0.029	3.70E-09	7.02E-07	0.095
PCaa C36:4	284	0.063	0.023	6.34E-03	1.00E+00	0.026
PCaa C36:5	281	0.034	0.161	8.35E-01	1.00E+00	0.000
PCaa C38	164	3.048	0.711	3.14E-05	5.97E-03	0.103
PCaa C38:3	162	0.133	0.104	2.02E-01	1.00E+00	0.010
PCaa C38:4	183	0.107	0.036	3.22E-03	6.12E-01	0.047
PCaa C38:5	238	0.442	0.095	5.87E-06	1.11E-03	0.084
PCaa C38:6	350	0.006	0.038	8.80E-01	1.00E+00	0.000
PCaa C40:4	154	3.389	1.009	9.90E-04	1.88E-01	0.070
PCaa C40:5	171	1.459	0.358	6.96E-05	1.32E-02	0.089
PCaa C40:6	349	-0.064	0.096	5.04E-01	1.00E+00	0.001

PCaa C44:12	172	7.422	3.763	5.02E-02	1.00E+00	0.022
PCae C34:1	350	1.434	0.255	3.72E-08	7.07E-06	0.083
PCae C34:2	283	1.330	0.197	7.78E-11	1.48E-08	0.141
PCae C34:3	350	1.847	0.213	1.60E-16	3.03E-14	0.178
PCae C36:1	235	0.582	0.151	1.54E-04	2.92E-02	0.059
PCae C36:2	153	0.985	0.297	1.16E-03	2.20E-01	0.067
PCae C36:3	350	1.639	0.238	2.47E-11	4.69E-09	0.121
PCae C36:4	283	0.575	0.127	8.76E-06	1.66E-03	0.068
PCae C36:5	350	1.091	0.195	4.77E-08	9.06E-06	0.083
PCae C38	182	0.268	1.018	7.92E-01	1.00E+00	0.000
PCae C38:3	182	0.352	0.220	1.11E-01	1.00E+00	0.014
PCae C38:4	172	1.627	0.336	2.92E-06	5.55E-04	0.122
PCae C38:5	287	0.414	0.148	5.35E-03	1.00E+00	0.027
PCae C38:6	238	1.755	0.373	4.37E-06	8.31E-04	0.086
PCae C40	164	0.000	0.425	1.00E+00	1.00E+00	0.000
PCae C40:4	171	1.595	0.731	3.06E-02	1.00E+00	0.027
PCae C40:6	171	2.504	0.847	3.55E-03	6.75E-01	0.049
SM C21:2	178	3.456	18.502	8.52E-01	1.00E+00	0.000
SM C31:1	181	1.921	14.016	8.91E-01	1.00E+00	0.000
SM C32:1	350	2.557	0.346	1.05E-12	1.99E-10	0.135

SM C32:2	170	9.631	4.214	2.35E-02	1.00E+00	0.030
SM C33:1	350	2.198	0.496	1.25E-05	2.38E-03	0.053
SM C34:1	350	0.230	0.033	2.10E-11	4.00E-09	0.121
SM C34:2	350	1.317	0.227	1.45E-08	2.75E-06	0.088
SM C35:1	301	4.043	0.934	2.04E-05	3.88E-03	0.059
SM C36:1	350	0.588	0.131	1.04E-05	1.98E-03	0.054
SM C36:2	220	1.339	0.412	1.34E-03	2.54E-01	0.046
SM C36:3	282	2.595	2.575	3.14E-01	1.00E+00	0.004
SM C41:1	238	1.153	0.273	3.43E-05	6.51E-03	0.070
SM C41:2	237	1.987	0.336	1.13E-08	2.15E-06	0.129
SM C42:1	185	0.559	0.165	8.37E-04	1.59E-01	0.058
SM C42:2	172	0.112	0.093	2.30E-01	1.00E+00	0.008
NEFA 10:0	335	1.365	0.321	2.69E-05	5.10E-03	0.052
NEFA 11:0	262	-26.008	9.748	8.11E-03	1.00E+00	0.027
NEFA 12:0	334	0.708	0.452	1.18E-01	1.00E+00	0.007
NEFA 12:1	219	26.212	11.720	2.63E-02	1.00E+00	0.023

NEFA 14:0	335	0.230	0.099	2.15E-02	1.00E+00	0.016
NEFA 14:1	334	1.202	0.535	2.54E-02	1.00E+00	0.015
NEFA 15:0	335	1.277	0.464	6.31E-03	1.00E+00	0.022
NEFA 15:1	212	20.921	7.554	6.12E-03	1.00E+00	0.035
NEFA 16:0	335	0.027	0.013	3.42E-02	1.00E+00	0.013
NEFA 16:1	335	0.060	0.057	2.91E-01	1.00E+00	0.003
NEFA 16:2	335	4.408	3.818	2.49E-01	1.00E+00	0.004
NEFA 17:0	335	1.638	0.461	4.36E-04	8.28E-02	0.037
NEFA 17:1	335	1.426	0.785	7.01E-02	1.00E+00	0.010
NEFA 18:0	280	0.053	0.031	9.00E-02	1.00E+00	0.010
NEFA 18:1	335	0.015	0.010	1.21E-01	1.00E+00	0.007
NEFA 18:2	333	0.061	0.032	5.60E-02	1.00E+00	0.011
NEFA 18:3	335	-0.050	0.133	7.07E-01	1.00E+00	0.000
NEFA 18:4	333	-3.453	8.648	6.90E-01	1.00E+00	0.000
NEFA 19:0	280	9.200	5.265	8.17E-02	1.00E+00	0.011
NEFA 19:1	335	1.900	1.649	2.50E-01	1.00E+00	0.004
NEFA 20:0	335	7.441	2.289	1.27E-03	2.41E-01	0.031
NEFA 20:1	335	0.450	0.564	4.26E-01	1.00E+00	0.002
NEFA 20:2	334	1.287	0.965	1.83E-01	1.00E+00	0.005
NEFA 20:3	335	0.722	0.586	2.19E-01	1.00E+00	0.005
NEFA 20:4	335	0.147	0.154	3.42E-01	1.00E+00	0.003
NEFA 20:5	333	0.469	1.557	7.64E-01	1.00E+00	0.000
NEFA 22:0	285	35.690	8.217	1.96E-05	3.73E-03	0.063
NEFA 22:1	334	2.664	7.099	7.08E-01	1.00E+00	0.000
NEFA 22:2	280	9.517	33.383	7.76E-01	1.00E+00	0.000
NEFA 22:3	230	-20.078	30.157	5.06E-01	1.00E+00	0.002
NEFA 22:4	334	2.688	1.925	1.64E-01	1.00E+00	0.006
NEFA 22:5	335	1.637	1.022	1.10E-01	1.00E+00	0.008
NEFA 22:6	335	0.471	0.464	3.10E-01	1.00E+00	0.003
NEFA 24:0	335	34.194	7.968	2.34E-05	4.44E-03	0.053
NEFA 24:1	335	3.702	6.826	5.88E-01	1.00E+00	0.001
NEFA 24:3	146	340.919	179.021	5.89E-02	1.00E+00	0.024
NEFA 24:4	218	100.184	48.327	3.94E-02	1.00E+00	0.020
NEFA 24:5	165	-10.022	47.090	8.32E-01	1.00E+00	0.000
NEFA 24:6	220	-81.486	30.159	7.44E-03	1.00E+00	0.033
NEFA 26:0	285	134.544	52.950	1.16E-02	1.00E+00	0.022
NEFA 26:1	335	-42.115	11.913	4.65E-04	8.84E-02	0.036
NEFA 26:2	286	7.792	16.070	6.28E-01	1.00E+00	0.001
NEFA 26:3	334	78.033	26.937	4.02E-03	7.64E-01	0.025
NEFA 26:4	164	-275.424	138.879	4.91E-02	1.00E+00	0.024
NEFA 26:5	180	-0.156	152.792	9.99E-01	1.00E+00	0.000

3-Methyl-2-oxovalerate	344	-0.024	0.171	8.89E-01	1.00E+00	0.000
4-Methyl-2-oxovalerate	344	0.014	0.100	8.92E-01	1.00E+00	0.000
Acetoacetate	243	0.028	0.008	3.46E-04	6.58E-02	0.052
Alpha-aminoadipate	158	-3.464	2.669	1.96E-01	1.00E+00	0.010
Alpha-ketobutyrate	330	0.142	0.036	8.75E-05	1.66E-02	0.046
Citrate	293	0.008	0.032	7.98E-01	1.00E+00	0.000
Isocitrate	294	0.944	0.162	1.57E-08	2.98E-06	0.104
Lactate	342	-0.002	0.001	1.64E-01	1.00E+00	0.006
Malate	342	0.512	0.207	1.38E-02	1.00E+00	0.018
Alpha-ketoglutarate	344	-0.116	0.141	4.10E-01	1.00E+00	0.002
Pyruvate	222	0.027	0.013	3.59E-02	1.00E+00	0.020
Succininate	344	0.283	0.189	1.35E-01	1.00E+00	0.007
Taurine	294	0.099	0.031	1.67E-03	3.17E-01	0.033
Sum AA	347	-0.004	0.003	2.80E-01	1.00E+00	0.003
Sum Carn	346	-0.019	0.042	6.44E-01	1.00E+00	0.001
Sum LPC	53	0.020	0.021	3.37E-01	1.00E+00	0.017
Sum PCaa	49	0.039	0.008	2.24E-05	4.25E-03	0.309
Sum PCae	50	0.236	0.063	5.13E-04	9.74E-02	0.228
Sum SM	54	0.252	0.080	2.61E-03	4.97E-01	0.158
Sum NEFA	213	0.004	0.005	4.36E-01	1.00E+00	0.003

GINI-LISA 10 years - HDL

Metabolite	N	Estimate	Std..Error	P	P.adjusted	PartialRsquared
Ala	250	-0.005	0.007	4.37E-01	1.00E+00	0.002
Arg	250	-0.004	0.032	8.95E-01	1.00E+00	0.000
Asn	250	0.079	0.057	1.68E-01	1.00E+00	0.007
Asp	249	-0.125	0.060	3.69E-02	1.00E+00	0.017
Cit	250	0.207	0.074	5.90E-03	1.00E+00	0.030
Gln	221	-0.005	0.006	4.28E-01	1.00E+00	0.003
Glu	250	-0.004	0.012	7.20E-01	1.00E+00	0.001
Gly	250	-0.003	0.011	7.95E-01	1.00E+00	0.000
His	250	0.036	0.031	2.44E-01	1.00E+00	0.005
Ile	250	-0.004	0.047	9.24E-01	1.00E+00	0.000
Leu	250	0.006	0.025	8.23E-01	1.00E+00	0.000
Lys	250	-0.012	0.018	5.01E-01	1.00E+00	0.002
Met	250	0.068	0.137	6.23E-01	1.00E+00	0.001
Orn	250	0.006	0.023	8.14E-01	1.00E+00	0.000
Phe	250	-0.020	0.041	6.23E-01	1.00E+00	0.001
Pro	250	-0.012	0.011	2.54E-01	1.00E+00	0.005
Ser	250	-0.036	0.022	1.08E-01	1.00E+00	0.010
Thr	250	0.008	0.022	7.02E-01	1.00E+00	0.001
Trp	250	0.027	0.055	6.19E-01	1.00E+00	0.001
Tyr	250	-0.018	0.042	6.71E-01	1.00E+00	0.001
Val	250	0.003	0.013	8.00E-01	1.00E+00	0.000
H1	252	0.001	0.001	1.02E-01	1.00E+00	0.010
Carn	191	-0.102	0.129	4.31E-01	1.00E+00	0.003
Carn C2	195	0.308	0.378	4.15E-01	1.00E+00	0.003
Carn C3	207	5.932	8.553	4.89E-01	1.00E+00	0.002
Carn C4	163	-12.267	9.998	2.22E-01	1.00E+00	0.009
Carn C5	153	6.298	22.852	7.83E-01	1.00E+00	0.000
Carn C6	251	34.917	16.539	3.58E-02	1.00E+00	0.017
Carn C8	252	5.219	2.229	2.00E-02	1.00E+00	0.021
Carn C8:1	251	7.618	10.504	4.69E-01	1.00E+00	0.002
Carn C9	230	39.297	26.767	1.43E-01	1.00E+00	0.009
Carn C10	251	4.331	1.731	1.30E-02	1.00E+00	0.024

Carn C10:1	252	9.068	2.798	1.36E-03	3.29E-01	0.039
Carn C10:2	153	7.665	17.911	6.69E-01	1.00E+00	0.001
Carn C12	251	6.962	3.974	8.10E-02	1.00E+00	0.012
Carn C12:1	223	2.587	1.870	1.68E-01	1.00E+00	0.008
Carn C14	162	47.059	32.035	1.44E-01	1.00E+00	0.013
Carn C14:1	251	8.824	6.124	1.51E-01	1.00E+00	0.008
Carn C14:2	163	40.608	25.289	1.10E-01	1.00E+00	0.015
Carn C16	230	36.712	25.539	1.52E-01	1.00E+00	0.009
Carn C16:1	200	59.134	41.224	1.53E-01	1.00E+00	0.010
Carn C18	189	4.138	30.277	8.91E-01	1.00E+00	0.000
Carn C18:1	252	13.533	15.700	3.90E-01	1.00E+00	0.003
Carn C18:2	178	63.423	37.845	9.56E-02	1.00E+00	0.015
LPC C14	252	-0.717	0.745	3.37E-01	1.00E+00	0.004
LPC C15	234	-1.268	1.859	4.96E-01	1.00E+00	0.002
LPC C16	252	-0.015	0.027	5.89E-01	1.00E+00	0.001
LPC C16:1	252	-1.143	0.828	1.69E-01	1.00E+00	0.007
LPC C17	252	-0.569	1.165	6.26E-01	1.00E+00	0.001
LPC C17:1	234	0.632	5.589	9.10E-01	1.00E+00	0.000
LPC C18	252	0.006	0.080	9.42E-01	1.00E+00	0.000
LPC C18:1	252	0.356	0.153	2.11E-02	1.00E+00	0.021
LPC C18:2	252	0.350	0.079	1.49E-05	3.63E-03	0.071
LPC C18:3	234	5.058	2.841	7.63E-02	1.00E+00	0.013
LPC C18:6	152	4.291	7.122	5.48E-01	1.00E+00	0.002
LPC C20:2	135	22.655	6.508	6.79E-04	1.65E-01	0.084
LPC C20:3	252	1.345	1.057	2.04E-01	1.00E+00	0.006
LPC C20:4	252	0.608	0.379	1.10E-01	1.00E+00	0.010
LPC C20:5	249	2.423	3.470	4.86E-01	1.00E+00	0.002

LPC C22:5	187	6.313	4.326	1.46E-01	1.00E+00	0.011
LPC C22:6	234	1.983	1.218	1.05E-01	1.00E+00	0.011
PCaa C30	252	0.848	0.543	1.20E-01	1.00E+00	0.009
PCaa C30:2	178	0.360	3.276	9.13E-01	1.00E+00	0.000
PCaa C30:3	173	-1.940	9.806	8.43E-01	1.00E+00	0.000
PCaa C30:4	126	0.685	17.070	9.68E-01	1.00E+00	0.000
PCaa C32	252	0.803	0.237	8.16E-04	1.98E-01	0.043
PCaa C32:1	252	0.014	0.127	9.11E-01	1.00E+00	0.000
PCaa C32:2	252	0.646	0.575	2.62E-01	1.00E+00	0.005
PCaa C32:3	197	0.667	3.891	8.64E-01	1.00E+00	0.000
PCaa C34:1	252	0.040	0.016	1.19E-02	1.00E+00	0.025
PCaa C34:2	252	0.040	0.009	1.23E-05	2.99E-03	0.072
PCaa C34:3	252	0.098	0.153	5.24E-01	1.00E+00	0.002
PCaa C34:4	252	-0.091	1.033	9.30E-01	1.00E+00	0.000
PCaa C34:5	167	11.582	9.130	2.06E-01	1.00E+00	0.010
PCaa C36	192	-0.956	0.401	1.82E-02	1.00E+00	0.028
PCaa C36:1	252	0.158	0.059	7.33E-03	1.00E+00	0.028
PCaa C36:2	252	0.045	0.012	3.36E-04	8.17E-02	0.049
PCaa C36:3	252	0.058	0.025	2.08E-02	1.00E+00	0.021
PCaa C36:4	252	0.056	0.019	3.99E-03	9.71E-01	0.032
PCaa C36:5	251	0.143	0.138	3.00E-01	1.00E+00	0.004
PCaa C36:6	231	-1.971	1.625	2.26E-01	1.00E+00	0.006
PCaa C38	252	0.353	0.640	5.81E-01	1.00E+00	0.001
PCaa C38:2	117	-0.370	0.556	5.07E-01	1.00E+00	0.004
PCaa C38:3	252	0.009	0.056	8.70E-01	1.00E+00	0.000
PCaa C38:4	252	0.052	0.026	4.41E-02	1.00E+00	0.016
PCaa C38:5	252	0.106	0.056	6.11E-02	1.00E+00	0.014
PCaa C38:6	252	0.070	0.036	5.36E-02	1.00E+00	0.015
PCaa C40	184	-2.976	1.788	9.79E-02	1.00E+00	0.014
PCaa C40:1	200	-1.590	1.221	1.94E-01	1.00E+00	0.008
PCaa C40:3	139	-3.543	1.104	1.66E-03	4.04E-01	0.068
PCaa C40:4	252	-0.042	0.537	9.37E-01	1.00E+00	0.000
PCaa C40:5	252	0.279	0.210	1.84E-01	1.00E+00	0.007
PCaa C40:6	252	0.084	0.089	3.44E-01	1.00E+00	0.004
PCaa C42	238	-0.541	2.453	8.26E-01	1.00E+00	0.000
PCaa C42:1	124	-10.018	4.139	1.70E-02	1.00E+00	0.044

PCaa C42:4	122	-10.745	3.200	1.06E-03	2.57E-01	0.085
PCaa C42:5	161	-1.463	3.778	6.99E-01	1.00E+00	0.001
PCaa C42:6	138	0.573	2.022	7.77E-01	1.00E+00	0.001
PCaa C43:6	249	4.889	2.030	1.68E-02	1.00E+00	0.023
PCaa C44:12	222	7.655	2.635	4.05E-03	9.83E-01	0.036
PCae C32	252	2.759	0.865	1.61E-03	3.91E-01	0.039
PCae C32:1	252	3.675	0.795	6.10E-06	1.48E-03	0.078
PCae C32:2	217	6.811	2.271	3.03E-03	7.37E-01	0.040
PCae C34	231	0.822	1.639	6.16E-01	1.00E+00	0.001
PCae C34:1	252	1.192	0.319	2.32E-04	5.65E-02	0.052
PCae C34:2	252	1.132	0.234	2.29E-06	5.56E-04	0.084
PCae C34:3	252	1.798	0.251	8.43E-12	2.05E-09	0.169
PCae C34:4	144	14.226	8.202	8.50E-02	1.00E+00	0.020
PCae C36	182	2.157	3.036	4.78E-01	1.00E+00	0.003
PCae C36:1	252	-0.171	0.210	4.15E-01	1.00E+00	0.003
PCae C36:2	252	0.592	0.189	1.96E-03	4.76E-01	0.037
PCae C36:3	252	1.309	0.310	3.41E-05	8.28E-03	0.066
PCae C36:4	252	0.489	0.134	3.10E-04	7.53E-02	0.050
PCae C36:5	252	0.832	0.194	2.53E-05	6.15E-03	0.068
PCae C36:6	129	5.651	4.488	2.10E-01	1.00E+00	0.011
PCae C38	251	-0.819	0.730	2.63E-01	1.00E+00	0.005
PCae C38:2	252	-0.748	0.339	2.81E-02	1.00E+00	0.019
PCae C38:3	252	-0.525	0.235	2.64E-02	1.00E+00	0.019
PCae C38:4	252	0.507	0.184	6.23E-03	1.00E+00	0.029
PCae C38:5	252	0.496	0.154	1.42E-03	3.46E-01	0.039
PCae C38:6	252	1.104	0.304	3.38E-04	8.22E-02	0.049
PCae C40	223	0.244	0.241	3.13E-01	1.00E+00	0.005
PCae C40:1	234	-1.142	0.827	1.69E-01	1.00E+00	0.008
PCae C40:2	171	-1.026	0.765	1.82E-01	1.00E+00	0.010
PCae C40:3	189	-1.435	0.407	5.36E-04	1.30E-01	0.061
PCae C40:4	223	-0.192	0.617	7.56E-01	1.00E+00	0.000
PCae C40:5	252	0.108	0.445	8.09E-01	1.00E+00	0.000
PCae C40:6	252	1.439	0.540	8.18E-03	1.00E+00	0.027
PCae C42	194	-2.972	2.148	1.68E-01	1.00E+00	0.010
PCae C42:1	172	-5.130	1.132	1.10E-05	2.68E-03	0.107
PCae C42:2	205	0.672	1.737	6.99E-01	1.00E+00	0.001
PCae C42:3	212	-1.017	1.143	3.75E-01	1.00E+00	0.004
PCae C42:4	190	2.957	2.138	1.68E-01	1.00E+00	0.010
PCae C42:5	232	0.648	0.872	4.58E-01	1.00E+00	0.002
PCae C42:6	252	1.975	1.534	1.99E-01	1.00E+00	0.006
SM C30:1	210	5.433	4.271	2.05E-01	1.00E+00	0.008
SM C31:1	125	12.681	10.471	2.28E-01	1.00E+00	0.012
SM C32:1	252	1.008	0.307	1.16E-03	2.82E-01	0.040

SM C32:2	252	4.365	2.937	1.39E-01	1.00E+00	0.009
SM C33:1	252	1.365	0.481	4.95E-03	1.00E+00	0.030
SM C33:2	184	14.660	11.594	2.08E-01	1.00E+00	0.008
SM C33:3	181	71.980	19.795	3.62E-04	8.81E-02	0.065
SM C34:1	252	0.101	0.032	1.83E-03	4.44E-01	0.037
SM C34:2	252	0.697	0.217	1.48E-03	3.61E-01	0.038
SM C35	168	5.303	4.447	2.35E-01	1.00E+00	0.008
SM C35:1	252	1.961	0.973	4.48E-02	1.00E+00	0.016
SM C35:2	203	16.286	5.824	5.68E-03	1.00E+00	0.036
SM C36:1	252	0.263	0.154	8.86E-02	1.00E+00	0.011
SM C36:2	252	0.572	0.281	4.31E-02	1.00E+00	0.016
SM C36:3	252	9.207	3.343	6.32E-03	1.00E+00	0.029
SM C37:1	128	1.088	1.416	4.44E-01	1.00E+00	0.004
SM C37:3	171	18.661	5.717	1.33E-03	3.23E-01	0.056
SM C38:1	195	0.251	0.085	3.60E-03	8.74E-01	0.042
SM C38:2	252	0.555	0.173	1.56E-03	3.79E-01	0.038
SM C38:3	151	5.149	5.564	3.56E-01	1.00E+00	0.006
SM C39:1	252	0.947	0.424	2.62E-02	1.00E+00	0.019
SM C39:2	152	2.687	1.578	9.07E-02	1.00E+00	0.018
SM C39:5	123	0.601	5.362	9.11E-01	1.00E+00	0.000
SM C40:2	252	0.321	0.096	9.37E-04	2.28E-01	0.042
SM C40:4	240	3.185	1.307	1.56E-02	1.00E+00	0.024
SM C40:5	216	4.206	2.958	1.57E-01	1.00E+00	0.009
SM C41:1	252	0.603	0.233	1.01E-02	1.00E+00	0.026
SM C41:2	252	1.297	0.320	6.90E-05	1.68E-02	0.060
SM C42:1	252	0.404	0.142	4.66E-03	1.00E+00	0.031
SM C42:2	252	0.213	0.079	7.38E-03	1.00E+00	0.028
SM C42:3	234	0.288	0.126	2.34E-02	1.00E+00	0.021
SM C42:4	216	0.555	0.349	1.14E-01	1.00E+00	0.011
SM C42:6	240	1.520	0.859	7.79E-02	1.00E+00	0.013
SM C43	144	1.200	4.292	7.80E-01	1.00E+00	0.001
SM C43:1	248	4.404	1.882	2.01E-02	1.00E+00	0.021
SM C43:2	252	1.985	1.102	7.27E-02	1.00E+00	0.013
SM C44:6	210	0.906	1.618	5.76E-01	1.00E+00	0.001
NEFA 10:0	240	1.948	0.751	1.00E-02	1.00E+00	0.027
NEFA 12:0	208	0.102	0.117	3.88E-01	1.00E+00	0.003
NEFA 12:1	151	1.496	1.029	1.48E-01	1.00E+00	0.013

NEFA 13:0	98	-2.098	2.436	3.91E-01	1.00E+00	0.007
NEFA 13:1	193	19.641	10.572	6.47E-02	1.00E+00	0.017
NEFA 14:0	251	0.058	0.105	5.81E-01	1.00E+00	0.001
NEFA 14:1	251	-0.298	0.628	6.35E-01	1.00E+00	0.001
NEFA 14:2	114	0.048	0.148	7.47E-01	1.00E+00	0.001
NEFA 16:0	252	0.014	0.017	4.19E-01	1.00E+00	0.003
NEFA 16:1	223	0.039	0.050	4.33E-01	1.00E+00	0.003
NEFA 16:2	225	2.694	2.845	3.45E-01	1.00E+00	0.004
NEFA 17:0	235	0.079	0.548	8.86E-01	1.00E+00	0.000
NEFA 17:1	223	0.406	0.813	6.18E-01	1.00E+00	0.001
NEFA 17:2	153	7.436	16.471	6.52E-01	1.00E+00	0.001
NEFA 18:0	164	-0.066	0.041	1.11E-01	1.00E+00	0.015
NEFA 18:1	252	0.014	0.012	2.57E-01	1.00E+00	0.005
NEFA 18:2	252	0.044	0.034	1.93E-01	1.00E+00	0.007
NEFA 18:3	252	0.167	0.104	1.10E-01	1.00E+00	0.010
NEFA 18:4	230	5.034	6.454	4.36E-01	1.00E+00	0.003
NEFA 19:0	236	7.974	4.378	6.98E-02	1.00E+00	0.013
NEFA 19:1	239	0.576	1.481	6.98E-01	1.00E+00	0.001
NEFA 20:0	132	-1.129	3.316	7.34E-01	1.00E+00	0.001
NEFA 20:1	252	0.584	0.568	3.05E-01	1.00E+00	0.004
NEFA 20:2	203	3.028	0.883	7.35E-04	1.79E-01	0.054
NEFA 20:3	230	0.495	0.717	4.91E-01	1.00E+00	0.002
NEFA 20:4	223	0.273	0.362	4.53E-01	1.00E+00	0.002
NEFA 20:5	188	0.149	1.278	9.07E-01	1.00E+00	0.000
NEFA 22:0	93	-0.698	6.393	9.13E-01	1.00E+00	0.000
NEFA 22:1	249	3.613	3.842	3.48E-01	1.00E+00	0.003
NEFA 22:2	138	40.888	24.271	9.44E-02	1.00E+00	0.020
NEFA 22:3	224	12.850	16.904	4.48E-01	1.00E+00	0.002
NEFA 22:4	224	0.026	1.937	9.89E-01	1.00E+00	0.000
NEFA 22:5	252	0.876	1.045	4.03E-01	1.00E+00	0.003
NEFA 22:6	252	0.431	0.445	3.33E-01	1.00E+00	0.004
NEFA 24:0	207	18.979	7.380	1.08E-02	1.00E+00	0.030
NEFA 24:1	217	4.529	5.773	4.34E-01	1.00E+00	0.003
NEFA 24:2	165	37.092	45.308	4.14E-01	1.00E+00	0.004
NEFA 24:3	155	295.896	137.335	3.28E-02	1.00E+00	0.028
NEFA 24:4	223	28.066	37.744	4.58E-01	1.00E+00	0.002
NEFA 24:5	232	23.410	25.240	3.55E-01	1.00E+00	0.004
NEFA 24:6	193	33.888	37.765	3.71E-01	1.00E+00	0.004
NEFA 26:0	163	-2.584	29.599	9.31E-01	1.00E+00	0.000
NEFA 26:1	206	26.942	27.475	3.28E-01	1.00E+00	0.005
NEFA 26:2	196	-75.339	38.854	5.40E-02	1.00E+00	0.019
NEFA 26:3	170	52.391	42.204	2.16E-01	1.00E+00	0.009
NEFA 26:4	207	-69.164	143.805	6.31E-01	1.00E+00	0.001
NEFA 26:5	195	-71.096	201.025	7.24E-01	1.00E+00	0.001

NEFA 26:6	134	1.008	83.365	9.90E-01	1.00E+00	0.000
Pyruvate	252	0.032	0.012	7.35E-03	1.00E+00	0.028
Lactate	252	-0.001	0.001	6.73E-02	1.00E+00	0.013
Acetoacetate	248	0.109	0.065	9.13E-02	1.00E+00	0.011
Alpha-ketobutyrate	233	-0.013	0.201	9.48E-01	1.00E+00	0.000
Fumarate	252	2.033	2.089	3.31E-01	1.00E+00	0.004
3-Methyl-2-oxobutanoate	252	0.730	0.219	9.73E-04	2.36E-01	0.042
Succininate	252	-0.899	0.302	3.18E-03	7.72E-01	0.034
Methylmalonate	252	-9.229	3.336	6.10E-03	1.00E+00	0.029
Taurine	252	0.032	0.028	2.64E-01	1.00E+00	0.005
3-Methyl-2-oxovalerate	252	0.602	0.205	3.69E-03	8.98E-01	0.032
4-Methyl-2-oxovalerate	252	0.381	0.122	1.94E-03	4.72E-01	0.037
Malate	252	-0.025	1.170	9.83E-01	1.00E+00	0.000
Alpha-ketoglutarate	251	1.060	0.531	4.70E-02	1.00E+00	0.015
Alpha-aminoadipate	252	-22.987	6.714	7.23E-04	1.76E-01	0.044
Isocitrate	239	5.162	3.927	1.90E-01	1.00E+00	0.007
Citrate	252	0.120	0.088	1.72E-01	1.00E+00	0.007
Sum AA	220	-0.001	0.001	3.31E-01	1.00E+00	0.004
Sum Carn	133	0.034	0.121	7.79E-01	1.00E+00	0.001
Sum LPC	231	0.020	0.016	1.97E-01	1.00E+00	0.007
Sum PCaa	251	0.008	0.002	7.89E-04	1.92E-01	0.043
Sum PCae	222	0.064	0.020	1.62E-03	3.95E-01	0.043
Sum SM	252	0.084	0.029	4.27E-03	1.00E+00	0.031
Sum NEFA	81	0.013	0.007	8.44E-02	1.00E+00	0.035

Ulm 8 years - ApoAI

Metabolite	N	Estimate	Std..Error	P	P.adjusted	PartialRsquared
Ala	397	0.000	0.000	8.11E-01	1.00E+00	0.000
Arg	396	-0.001	0.000	1.07E-01	1.00E+00	0.007
Asn	359	-0.002	0.001	2.58E-02	1.00E+00	0.014
Asp	360	-0.003	0.002	1.41E-01	1.00E+00	0.006
Cit	398	0.002	0.001	1.59E-01	1.00E+00	0.005
Gln	397	0.000	0.000	1.61E-01	1.00E+00	0.005
Glu	398	-0.001	0.001	2.45E-01	1.00E+00	0.003
Gly	396	0.000	0.000	3.70E-01	1.00E+00	0.002
His	398	0.000	0.000	6.64E-01	1.00E+00	0.000
Ile	357	0.001	0.001	4.71E-01	1.00E+00	0.001
Leu	398	0.000	0.000	3.02E-01	1.00E+00	0.003
Lys	397	0.000	0.000	4.18E-01	1.00E+00	0.002
Met	398	-0.003	0.002	1.06E-01	1.00E+00	0.007
Orn	397	0.000	0.001	8.39E-01	1.00E+00	0.000
Phe	398	-0.001	0.001	1.76E-01	1.00E+00	0.005
Pro	394	0.000	0.000	2.74E-01	1.00E+00	0.003
Ser	397	0.000	0.000	8.57E-01	1.00E+00	0.000
Thr	398	-0.001	0.000	1.65E-01	1.00E+00	0.005
Trp	397	0.001	0.001	3.67E-01	1.00E+00	0.002
Tyr	397	-0.001	0.001	3.96E-01	1.00E+00	0.002
Val	398	0.000	0.000	3.73E-01	1.00E+00	0.002
Hpro	396	0.002	0.001	5.56E-02	1.00E+00	0.009
H1	396	0.000	0.000	8.61E-01	1.00E+00	0.000
Carn	397	0.000	0.001	8.61E-01	1.00E+00	0.000
Carn C2	397	0.009	0.004	1.21E-02	1.00E+00	0.016
Carn C3	398	0.033	0.069	6.34E-01	1.00E+00	0.001
Carn C4	398	0.084	0.117	4.72E-01	1.00E+00	0.001
Carn C5	391	0.526	0.185	4.67E-03	9.47E-01	0.020
Carn C8:1	393	0.173	0.065	7.95E-03	1.00E+00	0.018

Carn C10:1	396	0.057	0.065	3.80E-01	1.00E+00	0.002
Carn C12:1	398	0.126	0.063	4.57E-02	1.00E+00	0.010
Carn C14:1	397	0.244	0.088	5.70E-03	1.00E+00	0.019
Carn C16	396	0.699	0.227	2.28E-03	4.62E-01	0.023
Carn C18	394	1.351	0.489	6.05E-03	1.00E+00	0.019
Carn C18:1	398	0.561	0.189	3.10E-03	6.30E-01	0.022
LPC C14	398	0.016	0.021	4.29E-01	1.00E+00	0.002
LPC C15	398	0.049	0.049	3.15E-01	1.00E+00	0.003
LPC C15:1	398	0.832	0.409	4.24E-02	1.00E+00	0.010
LPC C16	398	0.001	0.001	1.55E-01	1.00E+00	0.005
LPC C16:1	397	0.018	0.020	3.64E-01	1.00E+00	0.002
LPC C17	398	0.061	0.037	1.04E-01	1.00E+00	0.007
LPC C17:1	398	0.309	0.131	1.90E-02	1.00E+00	0.014
LPC C18	398	0.005	0.002	2.55E-02	1.00E+00	0.013
LPC C18:1	398	0.009	0.003	1.62E-03	3.29E-01	0.025
LPC C18:2	398	0.005	0.001	3.25E-04	6.60E-02	0.032
LPC C18:3	397	0.132	0.052	1.13E-02	1.00E+00	0.016
LPC C18:6	398	0.131	0.139	3.46E-01	1.00E+00	0.002
LPC C20	398	0.254	0.140	6.99E-02	1.00E+00	0.008
LPC C20:1	398	0.253	0.124	4.17E-02	1.00E+00	0.010
LPC C20:2	398	0.334	0.115	3.94E-03	7.99E-01	0.021
LPC C20:3	396	0.048	0.022	3.14E-02	1.00E+00	0.012
LPC C20:4	396	0.029	0.009	2.41E-03	4.89E-01	0.023
LPC C20:5	396	0.222	0.062	3.77E-04	7.65E-02	0.032
LPC C22:4	398	0.219	0.082	8.16E-03	1.00E+00	0.018

LPC C22:5	398	0.166	0.044	1.75E-04	3.55E-02	0.035
LPC C22:6	397	0.055	0.027	4.18E-02	1.00E+00	0.010
PCaa C18	398	0.257	0.222	2.47E-01	1.00E+00	0.003
PCaa C18:1	398	0.335	0.264	2.04E-01	1.00E+00	0.004
PCaa C30	398	0.014	0.006	2.18E-02	1.00E+00	0.013
PCaa C30:2	398	0.060	0.021	4.65E-03	9.44E-01	0.020
PCaa C32	398	0.003	0.001	4.22E-02	1.00E+00	0.010
PCaa C32:1	398	0.003	0.002	2.19E-02	1.00E+00	0.013
PCaa C32:2	398	0.012	0.006	4.27E-02	1.00E+00	0.010
PCaa C32:3	398	0.236	0.056	3.46E-05	7.02E-03	0.042
PCaa C34	396	0.007	0.004	7.44E-02	1.00E+00	0.008
PCaa C34:1	397	0.001	0.000	2.49E-07	5.05E-05	0.065
PCaa C34:2	397	0.001	0.000	3.66E-13	7.43E-11	0.126
PCaa C34:3	398	0.010	0.002	4.15E-06	8.43E-04	0.052
PCaa C34:4	398	0.031	0.013	1.47E-02	1.00E+00	0.015
PCaa C34:5	398	0.432	0.096	8.61E-06	1.75E-03	0.049
PCaa C34:6	384	0.903	0.207	1.65E-05	3.35E-03	0.048
PCaa C36	397	0.047	0.010	3.07E-06	6.23E-04	0.054
PCaa C36:1	398	0.002	0.001	2.89E-05	5.87E-03	0.043
PCaa C36:2	397	0.002	0.000	9.22E-21	1.87E-18	0.198
PCaa C36:3	398	0.004	0.000	2.17E-16	4.40E-14	0.157
PCaa C36:4	398	0.002	0.000	9.77E-14	1.98E-11	0.131
PCaa C36:5	398	0.008	0.002	4.46E-07	9.05E-05	0.063
PCaa C36:6	398	0.120	0.039	1.97E-03	4.00E-01	0.024
PCaa C38	398	0.093	0.014	4.90E-11	9.94E-09	0.104
PCaa C38:1	394	0.035	0.013	7.57E-03	1.00E+00	0.018
PCaa C38:2	398	0.025	0.006	1.06E-05	2.15E-03	0.048
PCaa C38:3	397	0.005	0.001	6.74E-05	1.37E-02	0.040
PCaa C38:4	398	0.002	0.000	3.31E-08	6.73E-06	0.075
PCaa C38:5	398	0.004	0.001	7.73E-08	1.57E-05	0.071
PCaa C38:6	398	0.003	0.001	1.88E-05	3.82E-03	0.045
PCaa C40	397	0.338	0.069	1.36E-06	2.77E-04	0.058
PCaa C40:1	391	0.552	0.131	3.37E-05	6.83E-03	0.043
PCaa C40:2	394	0.421	0.117	3.52E-04	7.15E-02	0.032
PCaa C40:3	390	0.231	0.069	9.10E-04	1.85E-01	0.028
PCaa C40:4	398	0.029	0.008	1.92E-04	3.90E-02	0.035
PCaa C40:5	397	0.017	0.003	2.04E-06	4.14E-04	0.056
PCaa C40:6	398	0.007	0.002	1.91E-04	3.87E-02	0.035
PCaa C42	398	0.288	0.061	3.84E-06	7.80E-04	0.053
PCaa C42:1	396	0.520	0.125	3.94E-05	7.99E-03	0.042
PCaa C42:2	392	1.162	0.203	2.01E-08	4.07E-06	0.078

PCaa C42:4	396	0.393	0.139	5.05E-03	1.00E+00	0.020
PCaa C42:5	395	0.335	0.086	1.10E-04	2.24E-02	0.037
PCaa C42:6	398	0.228	0.073	2.00E-03	4.06E-01	0.024
PCaa C43:6	398	0.136	0.028	1.50E-06	3.04E-04	0.057
PCae C30	398	0.244	0.063	1.31E-04	2.66E-02	0.036
PCae C32	395	0.047	0.010	4.06E-06	8.23E-04	0.053
PCae C32:1	398	0.082	0.013	7.32E-10	1.49E-07	0.092
PCae C32:2	398	0.480	0.071	5.37E-11	1.09E-08	0.103
PCae C34	396	0.042	0.014	2.45E-03	4.96E-01	0.023
PCae C34:1	398	0.022	0.004	4.61E-08	9.35E-06	0.073
PCae C34:2	398	0.023	0.003	6.29E-15	1.28E-12	0.143
PCae C34:3	398	0.030	0.003	1.24E-18	2.52E-16	0.179
PCae C34:4	398	0.639	0.091	8.38E-12	1.70E-09	0.112
PCae C36	397	0.148	0.034	1.31E-05	2.67E-03	0.047
PCae C36:1	398	0.037	0.007	7.32E-07	1.49E-04	0.060
PCae C36:2	398	0.029	0.003	1.18E-15	2.39E-13	0.150
PCae C36:3	398	0.049	0.005	3.04E-21	6.17E-19	0.203
PCae C36:4	397	0.013	0.002	2.82E-11	5.72E-09	0.107
PCae C36:5	398	0.022	0.003	1.25E-14	2.54E-12	0.140
PCae C36:6	393	0.257	0.041	1.57E-09	3.19E-07	0.089
PCae C38	397	0.094	0.018	5.12E-07	1.04E-04	0.062
PCae C38:2	397	0.099	0.016	3.88E-09	7.88E-07	0.084
PCae C38:3	398	0.057	0.009	7.90E-10	1.60E-07	0.091
PCae C38:4	396	0.014	0.002	1.96E-09	3.97E-07	0.088
PCae C38:5	397	0.012	0.002	2.69E-11	5.46E-09	0.107
PCae C38:6	398	0.028	0.004	1.88E-12	3.81E-10	0.118
PCae C40	398	0.028	0.008	3.41E-04	6.92E-02	0.032
PCae C40:1	393	0.076	0.017	1.39E-05	2.83E-03	0.047
PCae C40:2	398	0.180	0.047	1.23E-04	2.49E-02	0.037
PCae C40:3	397	0.285	0.045	7.78E-10	1.58E-07	0.092
PCae C40:4	398	0.085	0.015	1.49E-08	3.02E-06	0.078
PCae C40:5	398	0.079	0.011	6.19E-13	1.26E-10	0.123
PCae C40:6	398	0.067	0.010	1.71E-11	3.48E-09	0.108
PCae C42	398	0.220	0.090	1.49E-02	1.00E+00	0.015
PCae C42:1	396	0.093	0.049	5.94E-02	1.00E+00	0.009
PCae C42:2	398	0.194	0.050	1.25E-04	2.54E-02	0.037
PCae C42:3	397	0.273	0.047	1.68E-08	3.41E-06	0.078
PCae C42:4	398	0.193	0.033	1.11E-08	2.26E-06	0.079
PCae C42:5	398	0.106	0.019	4.46E-08	9.05E-06	0.073
PCae C42:6	398	0.170	0.029	7.39E-09	1.50E-06	0.081

SM C32:2	322	0.327	0.067	1.84E-06	3.74E-04	0.069
SM C35	397	0.053	0.029	7.03E-02	1.00E+00	0.008
SM C35:1	398	0.016	0.008	3.51E-02	1.00E+00	0.011
SM C36	397	0.017	0.010	9.34E-02	1.00E+00	0.007
SM C36:1	398	0.004	0.001	2.55E-03	5.17E-01	0.023
SM C36:2	398	0.007	0.002	1.93E-03	3.92E-01	0.024
SM C37:1	397	0.051	0.014	2.18E-04	4.42E-02	0.034
SM C38:1	398	0.004	0.001	1.25E-06	2.53E-04	0.058
SM C38:2	398	0.006	0.001	4.68E-05	9.50E-03	0.041
SM C38:3	398	0.103	0.034	2.62E-03	5.31E-01	0.023
SM C39:1	398	0.035	0.007	1.44E-07	2.92E-05	0.068
SM C39:2	396	0.092	0.020	8.23E-06	1.67E-03	0.049
SM C39:5	398	0.146	0.034	2.07E-05	4.21E-03	0.045
SM C40:1	395	0.002	0.001	2.93E-02	1.00E+00	0.012
SM C40:2	398	0.009	0.001	1.69E-10	3.43E-08	0.098
SM C40:3	395	0.013	0.003	1.54E-05	3.13E-03	0.047
SM C40:4	398	0.013	0.004	3.34E-04	6.79E-02	0.032
SM C41:1	398	0.016	0.003	4.51E-06	9.16E-04	0.052
SM C41:2	398	0.032	0.005	8.34E-10	1.69E-07	0.091
SM C41:3	390	0.075	0.023	1.25E-03	2.53E-01	0.027
SM C42:1	397	0.006	0.002	1.48E-03	3.00E-01	0.025
SM C42:2	398	0.003	0.001	6.48E-05	1.32E-02	0.040
SM C42:3	398	0.010	0.002	3.19E-07	6.47E-05	0.064
SM C42:4	397	0.027	0.005	2.30E-07	4.66E-05	0.066
SM C42:6	398	0.034	0.011	2.09E-03	4.24E-01	0.024
SM C43	396	0.184	0.050	2.53E-04	5.14E-02	0.034
SM C43:1	398	0.065	0.021	2.00E-03	4.05E-01	0.024
SM C43:2	398	0.041	0.014	2.65E-03	5.38E-01	0.023
SM C43:3	396	0.166	0.057	3.84E-03	7.80E-01	0.021
SM C44:2	394	0.196	0.073	7.49E-03	1.00E+00	0.018
SM C44:6	396	0.070	0.023	2.48E-03	5.03E-01	0.023
NEFA 10:0	320	0.021	0.011	6.14E-02	1.00E+00	0.011
NEFA 12:0	394	0.002	0.002	3.49E-01	1.00E+00	0.002
NEFA 12:1	391	0.025	0.010	1.43E-02	1.00E+00	0.015

NEFA 14:0	397	0.002	0.001	1.25E-01	1.00E+00	0.006
NEFA 14:1	395	0.013	0.008	1.05E-01	1.00E+00	0.007
NEFA 15:0	390	0.007	0.006	2.68E-01	1.00E+00	0.003
NEFA 15:1	331	0.072	0.090	4.23E-01	1.00E+00	0.002
NEFA 16:0	397	0.000	0.000	9.89E-02	1.00E+00	0.007
NEFA 16:1	396	0.001	0.001	1.10E-01	1.00E+00	0.006
NEFA 16:2	394	0.140	0.067	3.78E-02	1.00E+00	0.011
NEFA 17:0	395	0.008	0.007	2.53E-01	1.00E+00	0.003
NEFA 17:1	395	0.018	0.010	6.59E-02	1.00E+00	0.009
NEFA 18:0	394	0.001	0.000	6.09E-02	1.00E+00	0.009
NEFA 18:1	398	0.000	0.000	6.37E-02	1.00E+00	0.009
NEFA 18:2	397	0.001	0.000	2.45E-01	1.00E+00	0.003
NEFA 18:3	397	0.007	0.003	2.88E-02	1.00E+00	0.012
NEFA 19:1	398	0.049	0.021	2.18E-02	1.00E+00	0.013
NEFA 20:0	356	-0.003	0.038	9.40E-01	1.00E+00	0.000
NEFA 20:2	347	0.001	0.017	9.42E-01	1.00E+00	0.000
NEFA 20:3	281	0.019	0.022	3.80E-01	1.00E+00	0.003
NEFA 20:4	398	0.016	0.008	4.87E-02	1.00E+00	0.010
NEFA 20:5	383	0.108	0.051	3.58E-02	1.00E+00	0.012
NEFA 22:4	398	0.102	0.040	1.17E-02	1.00E+00	0.016
NEFA 22:5	398	0.055	0.021	8.00E-03	1.00E+00	0.018
NEFA 22:6	398	0.022	0.012	5.83E-02	1.00E+00	0.009
NEFA 24:0	395	0.138	0.134	3.04E-01	1.00E+00	0.003
NEFA 24:2	351	0.395	0.561	4.82E-01	1.00E+00	0.001
NEFA 24:4	392	0.175	0.656	7.90E-01	1.00E+00	0.000
NEFA 24:5	397	0.788	0.614	2.00E-01	1.00E+00	0.004
NEFA 26:1	398	0.508	0.233	2.97E-02	1.00E+00	0.012
